# SNOntology: Myriads of novel snornas or just a mirage?

**DOI:** 10.1186/1471-2164-12-543

**Published:** 2011-11-03

**Authors:** Julia A Makarova, Dmitri A Kramerov

**Affiliations:** 1 Engelhardt Institute of Molecular Biology, Russian Academy of Sciences, 32 Vavilov St., Moscow 119991, Russia

## Abstract

**Background:**

Small nucleolar RNAs (snoRNAs) are a large group of non-coding RNAs (ncRNAs) that mainly guide 2'-O-methylation (C/D RNAs) and pseudouridylation (H/ACA RNAs) of ribosomal RNAs. The pattern of rRNA modifications and the set of snoRNAs that guide these modifications are conserved in vertebrates. Nearly all snoRNA genes in vertebrates are localized in introns of other genes and are processed from pre-mRNAs. Thus, the same promoter is used for the transcription of snoRNAs and host genes.

**Results:**

The series of studies by Dahai Zhu and coworkers on snoRNAs and their genes were critically considered. We present evidence that dozens of species-specific snoRNAs that they described in vertebrates are experimental artifacts resulting from the improper use of Northern hybridization. The snoRNA genes with putative intrinsic promoters that were supposed to be transcribed independently proved to contain numerous substitutions and are, most likely, pseudogenes. In some cases, they are localized within introns of overlooked host genes. Finally, an increased number of snoRNA genes in mammalian genomes described by Zhu and coworkers is also an artifact resulting from two mistakes. First, numerous mammalian snoRNA pseudogenes were considered as genes, whereas most of them are localized outside of host genes and contain substitutions that question their functionality. Second, Zhu and coworkers failed to identify many snoRNA genes in non-mammalian species. As an illustration, we present 1352 C/D snoRNA genes that we have identified and annotated in vertebrates.

**Conclusions:**

Our results demonstrate that conclusions based only on databases with automatically annotated ncRNAs can be erroneous. Special investigations aimed to distinguish true RNA genes from their pseudogenes should be done. Zhu and coworkers, as well as most other groups studying vertebrate snoRNAs, give new names to newly described homologs of human snoRNAs, which significantly complicates comparison between different species. It seems necessary to develop a uniform nomenclature for homologs of human snoRNAs in other vertebrates, e.g., human gene names prefixed with several-letter code denoting the vertebrate species.

## Background

Small nucleolar RNAs constitute one of the largest groups of ncRNAs. They guide 2'-O-methylation and pseudouridylation of target RNAs, mainly rRNAs. SnoRNAs are divided into two groups according to the modification type: C/D box snoRNAs guide 2'-O-methylation, while H/ACA box snoRNAs guide pseudouridylation [1,2]. To date, ~200 RNAs of both groups have been described [3]. C/D box snoRNAs contain conserved C (UGAUGA) and D (CUGA) boxes brought together by complementary interactions between the snoRNA termini [4]. In addition, their (often imperfect) copies C' and D' are located internally [5]. Four core proteins bind these boxes, NOP56, NOP58, 15.5 kDa protein, and fibrillarin that catalyzes 2'-O-methylation [6]. Upstream of the D and/or D' box there is an antisense element of 9-20 nucleotides that is complementary to one of the cellular RNAs and is able to interact with it. A nucleotide in the cellular RNA located four nucleotides from the D/D' box in the resulting RNA/RNA duplex is 2'-O-methylated [2,7]. H/ACA box snoRNAs carry boxes H (ANANNA) and ACA (ACA) located at the base of two hairpins. The hairpins contain the antisense elements that are complementary to the target RNAs and are capable to interact with them. Four core proteins bind the H and ACA boxes, NHP2, NOP10, Gar1, and dyskerin; the latter catalyzes pseudouridylation [1,8]. Some C/D and H/ACA RNAs called scaRNAs are localized to Cajal bodies rather than to the nucleolus and guide modification of the snRNAs [9]. According to the new nomenclature accepted for human snoRNAs and scaRNAs, C/D snoRNAs, H/ACA snoRNAs, and scaRNAs are designated as SNORD, SNORA, and SCARNA, respectively [10].

Nearly all snoRNAs and scaRNAs genes in vertebrates are located within introns of other genes called host genes. The small RNAs are processed from pre-mRNAs of host genes [6,11]. Only SNORD3, SNORD13, SNORD118, SCARNA2, and SCARNA17 are transcribed from intrinsic promoters [3]. Most snoRNAs guide rRNA modifications. These modifications are essential for the ribosome function and probably contribute to rRNA folding, maturation, and stability [12,13]. The modification pattern is conserved in vertebrates: most 2'-O-methylation sites are identical between *Xenopus laevis *and human [14]. Homologous snoRNAs in different vertebrate species share the same antisense elements.

Recently, vertebrate snoRNAs have attracted the attention of several research groups [15-18]. In particular, our study of C/D snoRNAs in vertebrates demonstrated a trend towards low copy numbers of C/D snoRNA genes in placental mammals [16]. We have also demonstrated that the set of C/D snoRNAs is well conserved among vertebrates and that species-specific snoRNAs guiding rRNA modifications are extremely rare. Shortly after this publication, Zhu and coworkers reported opposite results [18,19]. Here, we demonstrate that their conclusions are incorrect due to a number of technical errors. We have mainly focused our criticism on their paper in *BMC *Genomics [18]; however, we also considered two other recent publications from the same group which are based on the same erroneous approaches [19,20].

## Results

### Lineage-specific and species-specific expression patterns of snoRNAs in rhesus monkey are experimental artifacts

Zhang et al. cloned 64 rhesus monkey snoRNAs encoded by 80 genes [18]. All of them were homologs of known human snoRNAs. Expression of these RNAs was tested by Northern hybridization in the muscle of several vertebrate species. Based on the results, Zhang et al. claimed that most of the cloned snoRNAs are not expressed in chicken, and some were not detected even in human and mouse (Table one in Zhang et al. [18]). Stated differently, they claimed lineage- or species-specific expression pattern for most of the cloned snoRNAs (59 out of 64).

This statement is contrary to the following. First, all snoRNAs cloned from rhesus monkey have been previously found in human (which allowed Zhang et al. to identify them) [3]. Second, the pattern of rRNA modifications as well as the set of snoRNAs guiding these modifications are conserved in vertebrates [14-17,21].

The data obtained by Zhang et al. can be interpreted in the following way. The efficiency of Northern hybridization is well known to decrease when a probe contains regions not complementary to the target. Sequence identity between snoRNA homologs from different vertebrate species ranges from ~55 to ~90%. Taxonomically close species have more similar snoRNA homologs. At the same time, different snoRNAs have different similarity levels (Table 1). Accordingly, a hybridization probe for a rhesus snoRNA does not necessarily allow the detection of this snoRNA homologs in other vertebrate species. For instance, we failed to detect SNORD87 RNA in birds using a probe for rat SNORD87, although it readily detected the homologs in different mammals ([22] and our unpublished data). This explains why Zhang et al. could detect only six chicken snoRNAs using rhesus snoRNA sequences as probes (Table one in Zhang et al. [18]). They claim that 58 out of 64 snoRNAs studied are not expressed in chicken; however, 33 of them have been identified by other researchers [17] by cDNA cloning (Additional file 1). Moreover, Zhang et al. reported many snoRNA species as not expressed in chicken [18] but had previously cloned them from chicken [19] (Additional file 1 and see below).

**Table 1:** Examples of similarity variation between mammalian and avian snoRNAs

SnoRNA	Human snoRNA identity to	
	
	mouse snoRNA, %	chicken snoRNA, %
SNORD46	92	61

SNORD87	88	71

SNORA13	82	56

The failure to detect snoRNA expression in human and mouse can be explained similarly. As one would expect, the closer genomic sequences, the more snoRNAs can be detected. Rhesus snoRNA probes detected more snoRNAs in human than in mouse, and more snoRNAs in mouse than in chicken (Table one in Zhang et al. [18]). Note that some snoRNAs whose expression was not detected in mouse (7 out of 17) had been described before (Additional file 1) [23-25]. Due to the same reasons, the attempt of Zhang et al. to detect snoRNAs that were not detected in muscle, in other human and mouse tissues also failed since the same rhesus probes were used.

The cases when snoRNA expression was not detected in human look particularly odd considering that all these snoRNAs have been initially described in human (Additional file 1). Moreover, the names specified, SNORA and SNORD, correspond to the new nomenclature specifically designed for human snoRNAs [10], a fact that alone indicates their expression in human. Thus, the lineage-specific and species-specific expression patterns of rhesus snoRNAs reported by Zhang et al. are experimental artifacts.

### Identification of species-specific ncRNAs in chicken results from improper use of Northern hybridization

A similar mistake was made by Zhang et al. in their publication describing chicken snoRNAs [19]. They cloned 125 chicken ncRNAs, mainly snoRNAs, and attempted to detect these RNAs in chicken, mouse, and human tissues by Northern hybridization. Similarly to the results discussed above, positive signal was largely observed in chicken only.

**Figure 1:**
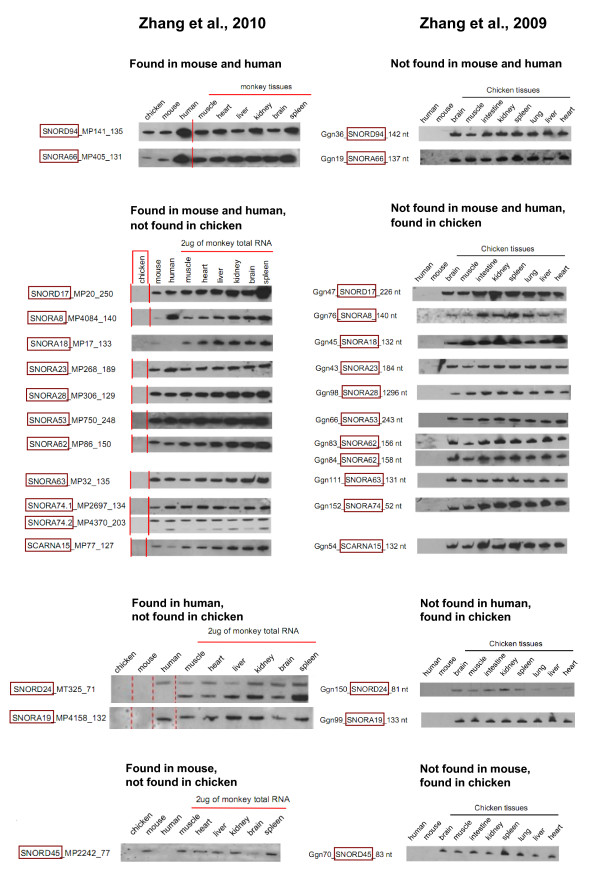
**Controversial results of detection of snoRNAs**. Hybridization of RNA isolated from different tissues of rhesus monkey, chicken, human, and mouse with rhesus snoRNA probes (left panel; from Zhang et al., 2010 [18]) and with chicken snoRNA probes (right panel; from Zhang et al., 2009 [19]). Conventional names are framed. The same RNAs are shown side-by-side. Clearly, the hybridization results on the left and on the right are mutually exclusive.

Zhang et al. detected the same snoRNAs in chicken but not in human and mouse [19]; and later, in rhesus, human, and/or mouse but not in chicken [18]. Each time species-specific expression of these snoRNAs was alleged. Examples of such detection experiments are given in Figure 1 and Additional file 2.

### Novel chicken ncRNAs are homologs of known human ncRNAs

Zhang et al. reported 35 new ncRNAs in chicken [19]. They claimed that these RNAs (with a single exception) can be detected by Northern hybridization only in chicken, and genes for most of them (28 out of 35) are absent in the genomes of other vertebrates. Table 2 demonstrates that 30 out of 35 so-called "novel" RNAs are homologs of previously described human small RNAs, 27 of which are snoRNAs. In each case, a snoRNA shares the antisense element with a human homolog (Additional file 3). Most of these allegedly new chicken RNAs can be identified by the search systems of the Rfam database of ncRNAs [21] and the snoRNABase of human nucleolar RNAs [3] (Table 2). Moreover, a good fraction of these "novel" chicken RNAs had been cloned by Shao et al. [17], and this fact was acknowledged by Zhang et al. (Table one in Zhang et al.[19]). Shao et al. managed to identify these RNAs as human snoRNA homologs, while Zhang et al. presented them as new RNAs. Thus, most novel ncRNAs described by Zhang et al. in chicken are homologs of well-known human ncRNAs.

**Table 2:** Chicken ncRNAs cloned and presented as novel RNAs by Zhang at al [19] are homologs of well-known human ncRNAs

**RNA ID**^1^	GenBank ID	RNA name	Identifiable by Rfam search	Identifiable by snoRNAbase search	**Cloned and properly identified by Shao et al**. [17]
GGN11	EU240230	**SNORD102B**^2^	No	**yes**	no

GGN20	EU240238	**SNORD1B**	No	**yes**	**yes **(GGgCD64)

GGN86	EU240302	**SNORD13**	**Yes**	**yes**	no

GGN120	EU240333	**fragment of SNORA84**	**Yes**	**yes**	no

GGN148	EU240352	**SNORD104**	No	**yes**	no

GGN100	EU240315	**SNORD11A**	**Yes**	**yes**	**yes **(GGgCD12A)

GGN71	EU240287	**SNORD127**	**Yes**	**yes**	no

GGN107	EU240321	**SNORD81**	**Yes**	**yes**	**yes **(GGgCD31)

GGN52	EU240268	**SNORD44**	**Yes**	**yes**	**yes **(GGgCD6)

GGN34	EU240252	**SNORD87C**	No	**yes**	**yes **(GGgCD46a)

GGN108	EU240322	**SNORD46A**	No	**yes**	**yes **(GGgCD47a)

GGN80	EU240296	**SNORD62**	No	**yes**	**yes **(GGgCD14)

GGN82	EU240298	**SNORD4**	**Yes**	**yes**	**yes **(GGgCD4)

GGN17	EU240236	**SNORD1A**	No	**yes**	**yes **(GGgCD64)

GGN79	EU240295	**SNORA77**	**Yes**	**yes**	**yes **(GGgACA12)

GGN72	EU240288	**SNORA40**	**Yes**	no	**yes **(GGgACA20)

GGN87	EU240303	**SNORA44**	**Yes**	**yes**	no

GGN58	EU240274	**SNORA17**	**Yes**	**yes**	no

GGN56	EU240272	**SNORA15**	**Yes**	no	no

GGN32	EU240250	**SNORA31B**	**Yes**	no	**yes **(GGgACA38)

GGN123	EU240336	**SNORA4**	No	no	**yes **(GGgACA26)

GGN74	EU240290	**SNORA64**	No	no	**yes **(GGgACA47)

GGN103	EU240318	**U4atac**	**Yes**	no	no

GGN141	EU240348	**SNORA25**	No	**yes**	**yes **(GGgACA11)

GGN67	EU240283	**fragment of SCARNA11**	**Yes**	no	**yes **(GGgACA29)

GGN105	EU240320	**NET3**^3^	No	no	no

GGN68	EU240284	**SNORD97**	No	**yes**	no

GGN46	EU240262	**SNORD43**	No	**yes**	**yes **(GGgCD29)

GGN147	EU240351	**Vault RNA**	**Yes**	no	no

GGN16	EU240235	**fragment of SNORD46B**	No	No	**yes **(GGgCD47b)

^1 ^According to Zhang et al. [19]**;**listed in the same order as in Table one in [19].

^2 ^The SNORD102B transcript has a longer antisense element than SNORD102A, and thus can guide the modification the rRNA nucleotide adjacent to that guided by SNORD102A [16].

^3 ^NET3 RNA is described by us [16] and is specific for vertebrates except placental mammals.

### Too long antisense elements and wrong target site predictions

**Figure 2:**
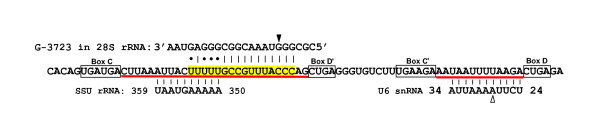
**Wrong prediction of snoRNA targets exemplified by rhesus monkey SNORD87 RNA**. C, D', C', and D sequences are boxed; the antisense element is marked yellow, and the complementary region in 28S rRNA is shown. The target nucleotide for 2'-O-methylation guided by SNORD87 is indicated by the solid arrowhead. The regions erroneously identified as antisense elements by Zhang et al. [18] are underlined in red. The putative SNORD87 targets identified by Zhang et al. are given below. The only possible SNORD87-guided modification among these targets is indicated by the empty arrowhead. This nucleotide is not methylated in human U6 snRNA.

Zhang et al. presented sequences of the C/D snoRNAs cloned from rhesus monkey and identified the whole fragments between C and D' boxes, as well as between C' and D boxes as the antisense elements (Additional file one in Zhang et al.[18], one example is given in Figure 2). However, it is known that an antisense element (or a guide sequence) is not a snoRNA fragment between the conserved boxes but rather a specific fragment complementary to the target RNA. In most cases it is not long, usually from 9 to 20 nt [3], which is much shorter than the fragments specified by Zhang et al.

Zhang et al. performed a computer search for the targets of rhesus C/D snoRNAs (Additional file three in Zhang et al.[18]). However, the targets for these snoRNAs were identified long ago, and the methylation of most of them was demonstrated [3]. For instance, SNORD87 RNA can guide modification of G-3723 in 28S rRNA, and this nucleotide is actually 2'-O-methylated [14,22] (Figure 2). With a few exceptions, the targets identified by Zhang et al. do not correspond to the confirmed ones. For example, the nucleotide in rhesus U6 RNA putatively modified by SNORD87 RNP is not methylated in human RNA [3] and, considering the conserved pattern of RNA modifications, is almost surely unmethylated in rhesus monkey (Figure 2). Zhang et al. identified methylation targets in 5S rRNA, whereas it has no 2'-O-methylated nucleotides in eukaryotes [26]. In addition, due to a small size of antisense elements, hundreds of potential targets can be proposed; and presenting some of them without experimental verification of their methylation status is unsubstantiated.

It was shown that a modified base is located four nucleotides upstream of the D/D' box in the C/D snoRNA/target RNA duplex [2,7]. In many cases presented by Zhang et al., e.g., in the putative SNORD87 target in SSU rRNA (Figure 2), a complementary sequence is more than four nucleotides away from the D/D' box, which makes the modification of these putative target RNAs by the proposed snoRNAs impossible.

### Numbers of snoRNAs and their gene copies in non-mammalian species is substantially underestimated

**Figure 3:**
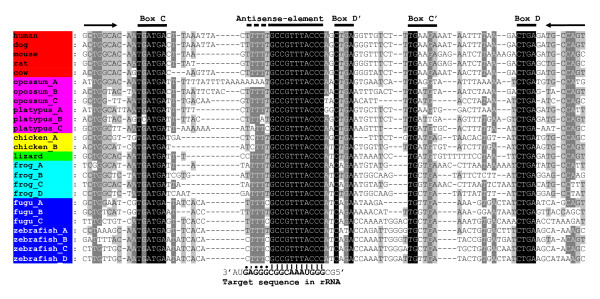
**Alignment of SNORD87 RNA genes**. Conserved elements are marked with lines above the alignment. A fragment of 28S rRNA complementary to the antisense element in SNORD87 is given below the alignment. The G-T complementarity is marked with dots. SNORD87 sequences are given for the following vertebrates: human (*Homo sapiens*), dog (*Canis familiaris*), mouse (*Mus musculus*), rat (*Rattus norvegicus*), cow (*Bos taurus*), opossum (*Monodelphis domestica*), platypus (*Ornithorhynchus anatinus*), chicken (*Gallus gallus*), lizard (*Anolis carolinensis*), frog (*Xenopus tropicalis*), fugu (*Takifugu rubripes*), and zebrafish (*Danio rerio*).

Zhang et al. stated that the numbers of snoRNAs and their genes increase from fish, amphibians, and birds to mammals [18]. Instead of a search for the new snoRNA genes, they used ENSEMBL annotations based on the Rfam database [27]. Identification of homologs of the experimentally detected ncRNAs is much more complex compared to protein homologs due to their low sequence similarity. In the case of snoRNAs, the conserved elements (antisense elements and C, C', D, and D' boxes in C/D snoRNAs or H and ACA boxes in H/ACA snoRNAs) comprise a half of the sequence length at most. The similarity level in non-conserved sequences varies between vertebrates and is usually low (Figure 3; Additional file 3). In addition, snoRNA genes in different species can be located within different introns of the same host gene or within different host genes. Thereby, many snoRNA genes are missing from lists created by annotation programs.

**Figure 4:**
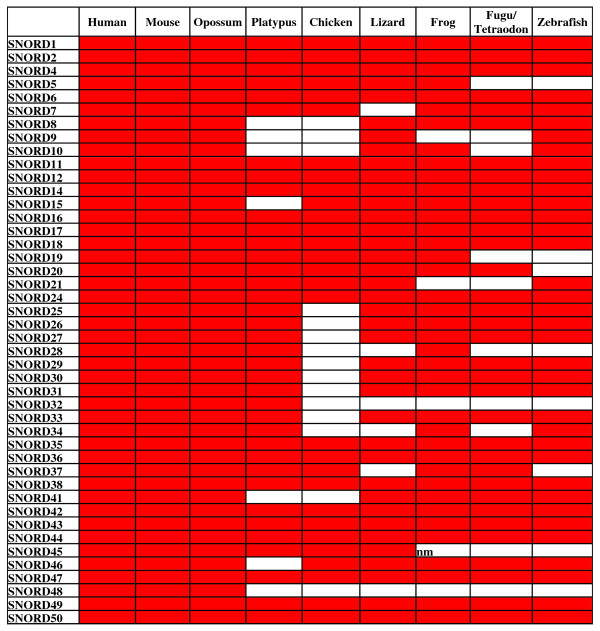
**Taxonomic distribution of C/D snoRNAs with identified targets^1^**. The genes that have been found by us in the genomes assemblies are marked red (Additional File 4). “nm,” not methylated site in *Xenopus *[14]. 1Targets are unknown for SNORD23, SNORD64, SNORD83, SNORD84, SNORD86, SNORD89, SNORD90, SNORD97, SNORD101, SNORD107, SNORD108, SNORD109, SNORD112, SNORD113, SNORD114, SNORD116, SNORD117, and SNORD124. Records SNORD39, SNORD40, SNORD106, SNORD120, and SNORD122 were deleted from the NCBI Nucleotide database. SNORD85 is an isoform of SNORD103. SNORD3, SNORD13, SNORD22, and SNORD118 guide no modifications.

**Figure 5:**
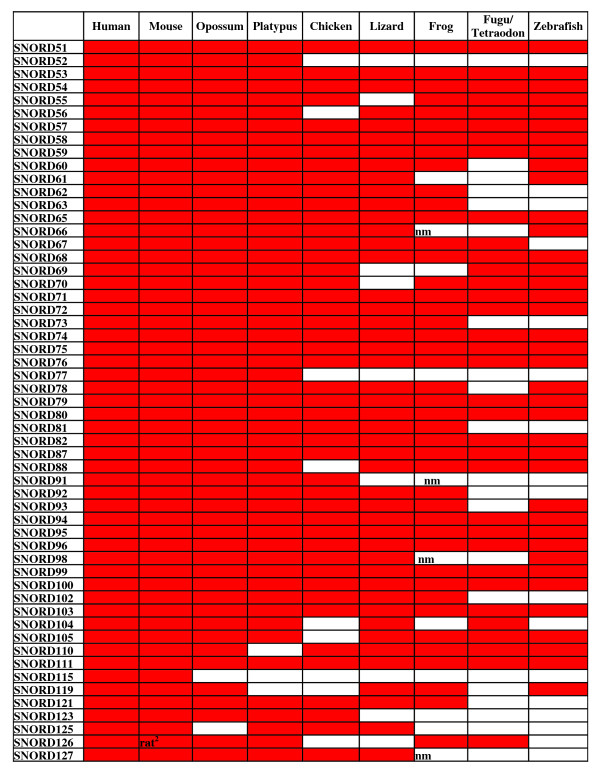
**Taxonomic distribution of C/D snoRNAs with identified targets**. *(Continued)*^2^The gene is missing in the mouse genome since the locus is deleted.

Our study on the numbers of C/D snoRNAs and their genes in representatives of different vertebrate classes [16] yielded results contrary to those obtained by Zhang et al. [18]. Instead of using automatic annotations, we searched for each C/D snoRNA in the vertebrate genomes using the WU BLAST 2.0 algorithm with specifically selected relaxed parameters; and the results of each search were manually inspected [16]. The data obtained and supplemented in this work (1352 C/D snoRNA genes; Figure 4, 5 and Additional file 4) did not reveal any significant increase in the number of C/D snoRNAs in mammals, as compared to other vertebrates. We found that most human snoRNAs have homologs in other vertebrate classes. Moreover, our data demonstrated a trend towards low copy numbers of C/D snoRNA genes in placental mammals. For instance, SNORD87 RNA is encoded by four genes in *Xenopus *and zebrafish each; two genes, in chicken; and by a single gene in human.

**Figure 6:**
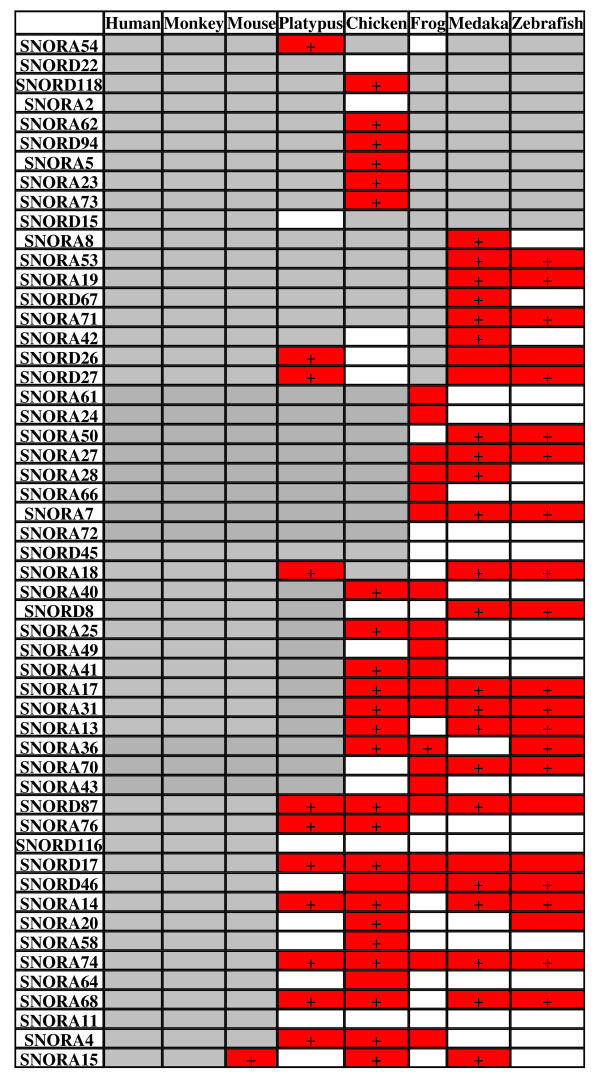
**Taxonomic distribution of snoRNA genes cloned from rhesus monkey by Zhang et al**. The gene names are listed in the same order as in Figure three of Zhang et al. [18]. The genes detected by Zhang et al. are marked grey, while those not detected by them but available in the open sources (see Additional file 5) are marked red. The latter genes available in Rfam 10.0 are indicated by the plus sign.

Zhang et al. failed to find many snoRNA genes in vertebrates. Figure 6 lists snoRNA genes identified by Zhang et al. (marked gray, according to Figure three in [18]) and missed by them but identified by other researchers (marked red [3,17,21], including our own data (Additional file 5)). The latter portion also includes snoRNAs cloned by Zhang et al. from chicken [19] (even though they claimed the absence of these RNAs in chicken in subsequent paper [18]). A plus sign in Figure 6 indicates genes present in the new release of Rfam (10.0), which shows how severely the conclusions by Zhang et al. depend on the Rfam release used. However, this release still does not contain many snoRNA genes identified in specific snoRNA studies (Figure 6). This particularly applies to the C/D RNA genes described by us (Additional file 4). Thus, studies specifically designed for a search of a particular group of ncRNAs in the whole genomes give much better results than the use of databases with automatically annotated ncRNAs.

In contrast to the consecutive increase in the number of snoRNAs from fish to mammals alleged by Zhang et al., we found that most mammalian C/D snoRNA genes have homologs in the genomes of other vertebrate classes (Figures 4, 5 and 6). This is not surprising considering that most snoRNAs are involved in rRNA modifications, and that the pattern of rRNA 2'-O-methylation and, likely, pseudouridylation is rather conserved in vertebrates [14]. The cases when some snoRNA gene is not found in a particular species can be attributed to the gaps in the genome sequences (which are abundant in the genomes of vertebrates excluding human and mouse). A minor fraction of snoRNA genes can be missing in some vertebrate classes considering some variations in the pattern of rRNA modifications between vertebrates. For instance, differential rRNA 2'-O-methylation between human and frog is observed in 9 out of ~100 sites [14]. It is of interest that about a half of missing snoRNA genes is observed in fishes (Figures 4, 5 and 6), which can point to a specific pattern of their rRNA methylation relative to other vertebrate classes.

### Number of mammalian snoRNA genes is substantially overstated

Zhang et al. stated that the number of snoRNA genes steadily increases in the series from fish to mammals, and that there is a burst in their number in mammals [18]. Again, ENSEMBL annotations based on the Rfam database were used rather than their own data. For each ncRNA, Rfam specifies all homologs in different species without specifying if a particular sequence is a gene or a pseudogene. This problem requires detailed examination of both the proper sequence and its genomic environment which is not covered by Rfam. Accordingly, Rfam records do not necessarily represent ncRNA genes, but may represent their pseudogenes as well, and this is clearly indicated in the Help section of the database [21]. However, Zhang et al. considered all corresponding Rfam and ENSEMBL entries as snoRNA genes: they reported the identification of 744 snoRNA genes in rhesus monkey, 922 genes in mouse, more than 1000 genes in human, and ~2200 genes in platypus. The problem of snoRNA gene copy numbers in mammals is discussed in several publications by different groups (see review [28] and references therein). All these data agree with each other, as well as with our data [16]: while the number of known mammalian snoRNAs is about 200, the total number of their genes does not exceed ~450 (i.e., some snoRNAs are encoded by single genes, and others are encoded by two, three, or more). This is substantially less than proposed by Zhang et al. Most mammalian-specific snoRNA genes found by them reside in intergenic regions rather than in introns. It is generally accepted that nearly all snoRNA genes of vertebrates are localized in introns of host genes, and only SNORD3 (U3), SNORD118 (U8), SNORD13 (U13), SCARNA2, and SCARNA17 are transcribed from their own promoters. It has been well documented that expression of the intronic snoRNAs requires transcription of the host genes (e.g., review [29] and references therein). That is why any sequence similar to an intronic snoRNA gene outside of introns is most likely a nonfunctional pseudogene. Only full-length copies with intact conserved regions and specific secondary structure can be considered as putative snoRNA genes. In addition, a search for a host gene, which may remain unannotated, should be done. Zhang et al. made no such analysis for the intergenic sequences annotated by ENSEMBL as snoRNA genes. Screening the human genome for snoRNA-like sequences revealed that most of them proved to be nonfunctional retrogenes with substitutions in the conserved regions [16,30]. Clearly, Zhang et al. considered such pseudogenes as snoRNA genes. We have demonstrated that the number of C/D snoRNA pseudogenes is much higher in mammals than in other vertebrates [16]. Therefore, the burst in mammalian snoRNA gene numbers alleged by Zhang et al. most likely represents the burst in the number of their pseudogenes.

Thus, Zhang et al. overestimated the number of snoRNA genes in mammals but underestimated the numbers of snoRNAs and their genes in other vertebrates. This led to a false conclusion that the numbers of snoRNAs and their genes increase in the series from fish to mammals.

### Are intronic snoRNA genes indeed transcribed from their own promoters?

SnoRNA pseudogenes with intact conserved regions could, in theory, be functional even when located outside of host gene introns, i.e. in intergenic regions. For that to happen, they should possess their own promoters that would allow independent transcription. Li et al. attempted to find such promoters for intergenic snoRNA-like sequences as well as independent promoters for snoRNA genes located within introns of the host genes [20]. They selected 745 putative human snoRNA genes, 326 of which were located in intergenic regions. This is much a higher number than the generally accepted estimate of the number of snoRNA genes (~450, see above). Again, Li et al. used ENSEMBL annotations, thus, combining snoRNA genes and pseudogenes. The search for snoRNA promoters using the CoreBoost_HM program [31] identified them in 179 out of 745 loci: 155 intronic loci and 24 intergenic ones (Table two in Li et al. [20]).

Based on these results, Li et al. proposed five models of snoRNA transcription. The first model assumes that transcription of a snoRNA and a host gene occurs from a common promoter and is generally accepted. This model describes most of the snoRNAs studied. Other models assume that transcription of a snoRNA gene occurs from an independent promoter.

The second model suggests an intronic snoRNA gene with its own promoter independent of a host gene promoter. This model was exemplified by one of SNORD3 (U3) genes located in an intron of the TEX14 gene on chromosome 17 (Model I, Figure one in Li et al. [20]). However, it is well known that SNORD3 always possesses its own promoter and requires no host gene for its transcription. Therefore, SNORD3 can not be used as an illustration of the proposed model. Moreover, the sequence on chromosome 17 has numerous substitutions in the functional regions and, hence, is a nonfunctional SNORD3 pseudogene (Additional file 6).

**Figure 7:**
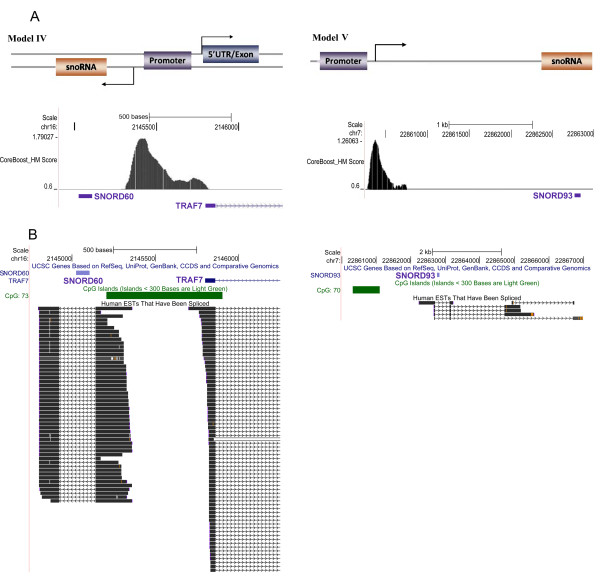
**Examples given by Li et al.**[**20**]**do not prove models IV and V of independent transcription of snoRNA genes.** (a) Models IV and V with the corresponding examples from [20]. (b) Screenshots of UCSC Genome Browser for the loci in panel (a) demonstrating that all snoRNA genes are localized within introns of host genes (EST track). Genomic coordinates for the March 2006 human reference sequence (NCBI Build 36.1) are given.

The other three models describe snoRNA genes located outside of host genes and putatively transcribed from their own promoters. However, the SNORA75 gene located on the plus strand of chromosome 12 and used for illustrating the third model (Model III, Figure one in Li et al. [20]) is actually a pseudogene with missing 5'-terminus (Additional file 6). Models IV and V are presented in Figure 7. One can see that the snoRNA genes are within introns of overlooked host genes rather than within intergenic regions. Thus, the promoters identified by Li et al. as snoRNA promoters are, in fact, host gene promoters.

Other genes identified by Li et al. as independently transcribed snoRNA genes are presented in Additional file 6. In each case, there is either an unnoticed host gene harboring snoRNA genes in its introns or a snoRNA pseudogene with substitutions questioning its functionality. A few exceptions are SNORA26-like sequence with intact functional regions and seven SNORD115 genes. However, there are no ESTs confirming independent transcription of these genes, whereas for all independently transcribed human snoRNAs ESTs marking their transcription can be found.

Thus, all examples of snoRNA independent transcription presented by Li et al. (possibly, excluding SNORA26-like sequence and SNORD115 genes) are inadequate.

## Discussion

### How many snoRNA genes are there?

Studies by Zhu and coworkers attracted our attention since their results were at variance with our data. The main contradiction was the estimated number of snoRNA genes in vertebrates. Our estimation of the number of mammalian C/D snoRNA genes [16] agrees with the data obtained by other groups: the total number of mammalian snoRNA genes known to date does not exceed ~450 (review [28] and references therein). In addition, we have shown a lower number of C/D snoRNA genes guiding rRNA modifications in mammals relative to other vertebrate classes [16]. Conversely, Zhang et al. stated that the number of mammalian snoRNA genes sharply increased to ~1000 compared to other vertebrate classes [18]. Here we demonstrated inadequacy of their techniques, which invalidates their conclusions. In particular, they considered numerous pseudogenes as snoRNA genes in mammals and failed to detect many snoRNA genes in other vertebrate classes.

### Northern hybridization has its limitations when used for detection of homologous ncRNAs in vertebrates

Possible existence of species-specific ncRNAs is extremely interesting, and it is being explored by many groups. Zhang et al. reported numerous lineage-specific and species-specific snoRNAs in chicken [19] and in rhesus monkey [18]. Here we demonstrated that their conclusions were based on a systemic error: Zhang et al. detected snoRNA homologs in vertebrate species using a probe for snoRNA of another vertebrate species, while the sequence identity of such homologs can go below 60% (Table 1). Under these conditions, standard Northern hybridization technique can not be used for homologs detection.

### Using automatically generated ncRNA databases alone can lead to erroneous conclusions

While application of genomic and EST sequence collections has become routine in bioinformatic studies, using automatic annotations of genes, especially ncRNA genes, requires great caution. For instance, ENSEMBL ncRNA annotations based on the Rfam data are excellent landmarks for genome researchers. However, the rates of false positives and missed genes in these annotations, at least in snoRNA annotations, make their application unacceptable for studies specifically designed to identify new ncRNA genes. For example, Rfam makes no distinction between snoRNA genes and pseudogenes, but Zhang et al. considered all annotated snoRNA sequences as snoRNA genes, which led them to erroneous conclusions [18,20]. In addition, existing automatically generated databases still do not include all ncRNA homologs in different species. Therefore, special studies are needed to prevent underestimation of ncRNA number. E.g., Rfam lacks many snoRNA sequences presented here (Additional file 4) or available in the snoRNABase [3]. Zhang et al. made no attempt to overcome this problem, and, as a result, missed many snoRNA genes in different vertebrates. Thus, relying only on automatic annotations can lead to erroneous conclusions. Actually, most researchers pursue their own way through the genomic thicket to succeed in snoRNA studies [25,32-34].

We especially focused on this issue since at least one more publication reported questionable conclusions concerning vertebrate snoRNAs based on the Rfam and ENSEMBL annotations as well as multispecies whole-genome alignments [35]. Again, the fact that snoRNA genes and pseudogenes are not distinguished in the Rfam entries was not taken into account.

### Names of snoRNA homologs need unification

Lots of snoRNAs have been described in different vertebrates to date, which necessitates the unification of their nomenclature. Zhang et al. gave a new name to each chicken homolog of human snoRNA [19]. This practice is not exclusive to Zhang et al. but is common in almost all publications describing snoRNAs in vertebrates apart from human. This was justified during the period of time when novel snoRNAs rather than homologs of known ones were being identified (e.g., [23]). Presently, a convenient nomenclature has been developed for human snoRNAs [10], and identification of novel snoRNAs has become extremely rare. In this context, giving new names to snoRNAs, whose homologs have been identified in other vertebrates, is highly confusing. It gives an erroneous impression that novel snoRNAs have actually been found and confuses the overall picture. For instance, a special investigation should be conducted to understand that the GGgCD37b snoRNA identified in chicken by Shao et al. [17] corresponds to Ggn109 found by Zhang et al. in chicken, too [19], and is a homolog of human SNORD38. The analysis of the whole set of data presented in these papers becomes hardly practicable. Finally, it is very hard to recognize the rare cases of a truly novel RNA identification. A positive practice in the field can be exemplified by the Rfam database specifying all homologs of human snoRNAs by the human RNA name. Since new publications describing snoRNAs in vertebrates can be expected, we propose to develop a nomenclature convention for the homologs. The human snoRNA names can be used with prefixes denoting the vertebrate species, e.g., mmusSNORD87 for the mouse homolog of human SNORD87. We propose to use four-letter prefixes to distinguish species such as *Mus musculus *(mmus) and *Microcebus murinus *(mmur).

### Independent transcription of snoRNA genes is an intriguing possibility, but it needs strong support

Recent data indicate that many miRNA genes located within introns of host genes have their own promoters [36]. This interesting and unexpected finding inspires one to test a similar pattern in snoRNAs, nearly all of which are encoded within introns in vertebrates. Noteworthily, no experimental data supporting the hypothesis of intronic snoRNAs transcription from their own promoters are available to date. At the same time, their transcription within the host gene pre-mRNA from the host gene promoter has been well documented dozens of times (e.g., review [29] and references therein). Thus, the idea of transcription of intronic snoRNAs from their own promoters is at variance with our current knowledge about their expression, and identification of such promoters should have solid experimental support. Preliminary bioinformatic analysis can be beneficial, but it should be adequate and thorough, which was not the case with Li et al. [20].

### Erroneous data begin to shape our view of ncRNAs

Currently, discovery of the species-specific ncRNAs is generally anticipated that may lead to less critical peer reviewing of publications reporting such RNAs. Here we show that the result can be harmful to the field. Even more importantly, such publications began to misshape our understanding of ncRNAs: one of the papers criticized here [18] has already been cited in a recent review [37].

Vertebrate genomes may actually contain many not yet identified snoRNAs. This idea is supported by the data from several groups [32,33,38]. However, publications like the ones considered here only add confusion to the problem rather than contribute to the solution. Thus, it is very important to prevent a false start in this exciting field.

## Methods

Homologs of human C/D box snoRNA genes in vertebrate genomes were searched as follows. First, homologs of human host genes were found in vertebrate genomes using the Comparative Genomics panel of UCSC Genome Browser at http://genome.ucsc.edu[39]. Then, the introns of the host genes were manually searched for the presence of snoRNA genes. If unsuccessful, snoRNA sequences were searched by WU-BLAST 2.0 http://www.ensembl.org/Multi/blastview with increased sensitivity parameters: high sensitivity (search for distant homologies) was chosen; W (word size for seeding alignments) = 3 and Q (cost of first gap character) = 1 were set. The intronic location of the search hits was checked using the mRNA and EST databases integrated into the UCSC Genome Browser. The hits with intact C, D/D' boxes, and the antisense element, flanked by short inverted repeats and located within introns of host genes were considered as snoRNA genes. Finally, extra copies of snoRNA genes were searched in the host gene introns.

NcRNAs discussed in [18-20] were analyzed using the UCSC Genome Browser and snoRNABase and Rfam databases [3,21]. Pairwise and multiple alignments were generated by Clustal V and Clustal W [40,41]. RNA secondary structures were analyzed using the mfold program [42,43].

## Conclusions

Several recent publications reported numerous lineage-specific snoRNAs in vertebrates. However, the myriads of novel snoRNAs are just a mirage. The approaches used allowed no identification of human homologs of these "new" RNA species. Despite substantial sequence variation in snoRNA homologs in different vertebrates, they can be easily identified by the same antisense elements. The conclusion of elevated numbers of snoRNA genes in mammalian genomes relative to other vertebrates also proved erroneous, since no distinction was made between snoRNA genes and pseudogenes and no thorough analysis of recently sequenced genomes of non-mammalian vertebrates was conducted. The reported evidence for the transcription of many snoRNA genes from their own promoters is inconclusive.

## Authors' contributions

JM and DK conceived the study. JM carried out all analyses and drafted the manuscript. Both authors read and approved the final manuscript.

## Supplementary Material

Additional file 1 **NcRNAs whose expression has not been detected by Zhang et al**. [**18**]**by Nothern hybridization in chicken, mouse, and human but was detected previously by other authors as well as by Zhang et al.**[**19**]. The order of RNAs is as in Table one from Zhang et al. [18]. Click here for file

Additional file 2 **Controversial results of ncRNA detections in chicken and rhesus monkey (14 extra examples)**. Hybridization of RNA isolated from different tissues of rhesus monkey, chicken, human, and mouse with rhesus snoRNA probes (left panel; from [18]) and with chicken snoRNA probes (right panel; from [19]). The same RNAs are shown side-by-side. Chicken ncRNAs were cloned by Zhang et al. but not identified as homologs of human snoRNAs [19] (shown on the right). The same RNAs are presented in Table 2. Click here for file

Additional file 3 **The majority of chicken ncRNAs cloned and presented as novel RNAs by Zhang at al**. [19] are homologs of ncRNAs described previously. Alignments of chicken ncRNAs with the homologs in human or sometimes other vertebrates are shown. GGN sequences are from Zhang et al. [19]. Vault RNA sequence corresponds to the GenBank AF045143 sequence. Other sequences are from snoRNABase [3] and Additional file 4 in this paper. C, D/D', H, ACA, and CAB boxes are underlined; antisense elements are boxed; sequence numbering corresponds to human rRNAs in snoRNABase. In C/D snoRNAs, the nucleotide complementary to the modification site is indicated by the red arrowhead. For the vault RNAs, the secondary structures predicted by mfold [42,43] are shown. The order of ncRNAs is as in Table 2. The SNORD102B transcript has a longer antisense element, and thus can guide the modification of the rRNA nucleotide adjacent to that modified by SNORD102A (marked with black and red arrowheads, respectively) [16]. Click here for file

Additional file 4 **Nucleotide sequences of C/D box snoRNA genes in different vertebrate species**. Boxes C, D, and D' are shown in gray, and sequences of the antisense elements are highlighted in yellow. The G-T complementarity in the antisense elements or terminal stems is indicated in olive. The 5' and 3' terminal complementary regions forming the stem in snoRNAs are shown in blue. Species-specific complementary substitutions in the antisense elements are marked in pink. Pseudogenes are indicated by Ψ. SNORD115 gene clusters are not listed. They have been found only in eutherian mammals and are available in snoRNABase [3] and UCSC Genome Browser. The following genome assemblies were used: human, March 2006, NCBI Build 36.1; mouse, July 2007, NCBI Build 37; rat, November 2004, version 3.4; dog, May 2005, whole genome shotgun assembly v2.0, cow, October 2007, Baylor release Btau_4.0; horse, January 2007, UCSC version equCab1; opossum, January 2006, monDom4; platypus, March 2007, the v5.0.1 draft assembly; chicken, May 2006, galGal3 version 2.1 draft assembly; lizard, February 2007, Broad Institute AnoCar 1.0; frog, August 2005, whole genome shotgun assembly version 4.1; zebrafish, July 2007, Zv7 assembly; fugu, October 2004, v4.0 whole genome shotgun assembly; tetraodon, February 2004, V7 assembly; stickleback, February 2006, v 1.0 draft assembly; medaka, October 2005, v 1.0 draft assembly. Click here for file

Additional file 5 **SnoRNA genes not found in the genomes of studied species by Zhang et al**. [18] but found in the same species by other researchers. Gene names are listed in the same order as in Figure three in [18]. Click here for file

Additional file 6 **Nearly all examples of independent transcription of snoRNA genes in Li et al**. [20] are erroneous. Screenshots of UCSC Genome Browser (March 2006, NCBI Build 36.1) and nucleotide sequence alignments of snoRNA genes and pseudogenes are shown. The antisense elements are boxed; H, ACA, C, and D/D' sequences are underlined. The nucleotides whose modification is guided by snoRNA are indicated in some cases. SnoRNA genes and pseudogenes (designated as pseudo or Ψ) are listed in the same order as in Tables three, four, and five of Li et al. [20]. The secondary structures were predicted by mfold [42,43]. Click here for file

## Figures and Tables

**Table 3:** Summary of C/D box snoRNA numbers predicted by M&K in 16 vetebrate genomes (data from additional file five of M&K)

Species	Predicted snoRNA number
stickle-back	1
horse	2
medaka	9
human	20
cow	27
rat	27
fugu	53
dog	64
tetraodon	82
chicken	118
lizard	129
mouse	143
opossum	156
platypus	166
zebrafish	167
frog	188

TOTAL	1352

**Table 4:** Numbers of C/D box snoRNAs in human genome reported by different groups

Data resourse	Number of C/D box snoRNA	Released year	References
snoRNA-LBME-db	269	2006	[3]

The HUGO Gene Nomenclature Committee	272	2011	[53]

Rfam (release 10.0)	223	2010	[44]

ENSEMBL (release 63)	460 (593)*	2011	[54]

ENSEMBL (release 50)**	387 (502)*	2008	[27]

Reported by M&K	141	2009	[16]

Reported by M&K	20	2011	Additional file four of M&K

* Numbers of C/D box snoRNAs excluding U3 and U13 are given. Copy numbers of those two snoRNA families are shown in brackets.

** The version of ENSEMBL database used in our previous study [18].

**Figure 8:**
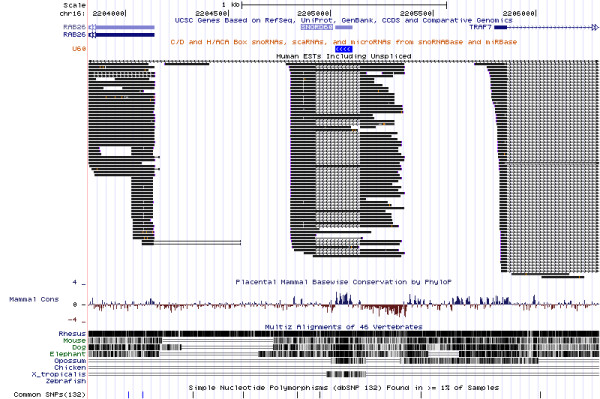
**Screenshot of UCSC Genome Browser for the SNORD60 locus to demonstrate presence of many unspliced ESTs**.
