# Supplementary material for: SNOntology: Myriads of novel snornas or just a mirage?
Source: BMC Genomics. 2011 Nov 3;12:543. doi: 10.1186/1471-2164-12-543 (PMC3349704; doi:10.1186/1471-2164-12-543)
Supplement: Additional file 1 — NcRNAs whose expression has not been detected by Zhang et al. [18]by Nothern hybridization in chicken, mouse, and human but was detected previously by other authors as well as by Zhang et al.[19]. The order of RNAs is as in Table one from Zhang et al. [18]. [file 1471-2164-12-543-S1.DOC]

**Additional file 1. NcRNAs whose expression has not been detected by Zhang et al. [18] by Nothern hybridization in chicken, mouse, and human but was detected previously by other authors as well as by Zhang et al**. [19]. The order of RNAs is as in Table 1 from Zhang et al. [18].

**Non-coding RNAs, whose expression has not been detected in chicken by Zhang et al. [18].**

| RNA name | | Cloned from chicken by | |
| --- | --- | --- | --- |
| New | Old | Shao et al. [17] | Zhang et al. [19] |
| SCARNA11 | ACA57 | GGgACA29 | GGN67 |
| SCARNA15 | ACA45 | GGgACA49 | GGN54 |
| SNORA13 | ACA13 | GGgACA25 | - |
| SNORA15 | ACA15 | - | GGN56 |
| SNORA17 | ACA17 | - | GGN58 |
| SNORA18 | ACA18 | GGoACA2 | GGN45 |
| SNORA23 | ACA23 | - | GGN43 |
| SNORA24 | ACA24 | GGgACA16 | - |
| SNORA28 | ACA28 | GGgACA15 | GGN98 |
| SNORA36 | ACA36 | GGgACA24A | GGN124 |
| SNORA4 | ACA4 | GGgACA26 | GGN123 |
| SNORA41 | ACA41 | GGgACA28 | GGN101 |
| SNORA5 | ACA5 | GGgACA22,  GGgACA23 | - |
| SNORA53 | ACA53 | - | GGN66 |
| SNORA54 | ACA54 | GGgACA39 | - |
| SNORA58 | ACA58 | GGgACA6 | - |
| SNORA62 | E2 | GGgACA40 | GGN83 |
| SNORA63 | E3 | GGgACA30 | GGN111 |
| SNORA71 | U71 | GGgACA8 | - |
| SNORA72 | U72 | GGgACA1 | GGN49 |
| SNORA74 | U19 | - | GGN152 |
| SNORD13 | U13 | - | GGN86 |
| SNORD15 | U15 | GGgCD48 | GGN122 |
| SNORD17 | HBI-43 | GGgCD50 | GGN47 |
| SNORD46 | U40, U46 | GGgCD47 | GGN108 |
| SNORD67 | HBII-166 | GGgCD82 | - |
| U4atac | U4atac | - | GGN103 |
| Vault RNA | Vault RNA | - | GGN147 |
| SNORA31 | ACA31 | GGgACA7, GGgACA38 | GGN13, GGN32 |
| SNORA8 | ACA8 | GGoACA3 | GGN76 |
| SNORD16 | U16 | GGgCD11 | GGN69 |
| SNORA25 | ACA25 | GGgACA11 | GGN141 |
| SNORA19 | ACA19 | GGgACA17, GGgACA37 | GGN99, GGN125 |
| SNORA27 | ACA27 | GGgACA13 | - |
| SNORA40 | ACA40 | GGgACA20 | GGN72 |

Non-coding RNAs, whose expression has not been detected in chicken and mouse by Zhang et al. [18].

| RNA name | | Cloned from chicken by | | Detected in mouse |
| --- | --- | --- | --- | --- |
| New | Old | Shao et al. [17] | Zhang et al. [19] |
| SNORD116 | HBII-85 | - | - | MBII-85^1^ |
| SNORA64 | U64 | GGgACA47 | GGN74 | MBI-29 [23] |
| SNORA68 | U68 | - | - | MBI-30 [23] |
| SNORD24 | U24 | GGgCD38 | - | U24 [24] |
| SNORA76 | ACA62 | - | - | ACA62 [25] |
| SNORD80 | U80, Z15 | GGgCD36 | GGN104 | U80^2^ |

^1^Cloned by Cavaille et al. (Cavaillé J, Buiting K, Kiefmann M, Lalande M, Brannan CI, Horsthemke B, Bachellerie JP, Brosius J, Hüttenhofer A: **Identification of brain-specific and imprinted small nucleolar RNA genes exhibiting an unusual genomic organization.** *Proc Natl Acad Sci USA* 2000, **97**:14311-14316).

^2^ Described by Smith and Steitz (Smith CM, Steitz JA: **Classification of gas5 as a multi-small-nucleolar-RNA (snoRNA) host gene and a member of the 5'-terminal oligopyrimidine gene family reveals common features of snoRNA host genes.** *Mol Cell Biol* 1998, **18**:6897-6909).

Non-coding RNAs, whose expression has not been detected in chicken, mouse, and human by Zhang et al. [18].

| RNA name | | Cloned from chicken by | | Detected in mouse | Detected in human |
| --- | --- | --- | --- | --- | --- |
| New | Old | Shao et al. [17] | Zhang et al. [19] |
| SNORA20 | ACA20 | GGgACA10 | - | - | ACA20^3^ |
| SNORD26 | U26 | - | - | - | U26^4^ |
| SNORD27 | U27 | - | - | - | U27^4^ |
| SNORD87 | HBII-276, U87 | GGgCD46 | GGN34 | MBII-276 [23], U87 [22] | U87 [22] |

^3^ Cloned by Kiss et al. (Kiss AM, Jády BE, Bertrand E, Kiss T: **Human box H/ACA pseudouridylation guide RNA machinery.** *Mol Cell Biol* 2004, **24**:5797-5807).

^4^ Described by Tycowski et al. (Tycowski KT, Shu MD, Steitz JA: **A mammalian gene with introns instead of exons generating stable RNA products.** *Nature* 1996, **379**:464-466).

Non-coding RNA, whose expression has not been detected in chicken and human by Zhang et al. [18].

| RNA name | | Cloned from chicken by | | Detected in human |
| --- | --- | --- | --- | --- |
| New | Old | Shao et al. [17] | Zhang et al. [19] |
| SNORD45 | U45 | GGgCD5 | GGN70 | U45 [2] |
