# Supplementary material for: SNOntology: Myriads of novel snornas or just a mirage?
Source: BMC Genomics. 2011 Nov 3;12:543. doi: 10.1186/1471-2164-12-543 (PMC3349704; doi:10.1186/1471-2164-12-543)

Additional file 2. Controversial results of ncRNA detections in chicken and rhesus monkey (14 extra examples). Hybridization of RNA isolated from different tissues of rhesus monkey, chicken, human, and mouse with rhesus snoRNA probes (left panel; from [18]) and with chicken snoRNA probes (right panel; from [19]). The same RNAs are shown side-by-side. Chicken ncRNAs were cloned by Zhang et al. but not identified as homologs of human snoRNAs [19] (shown on the right). The same RNAs are presented in Table 2.

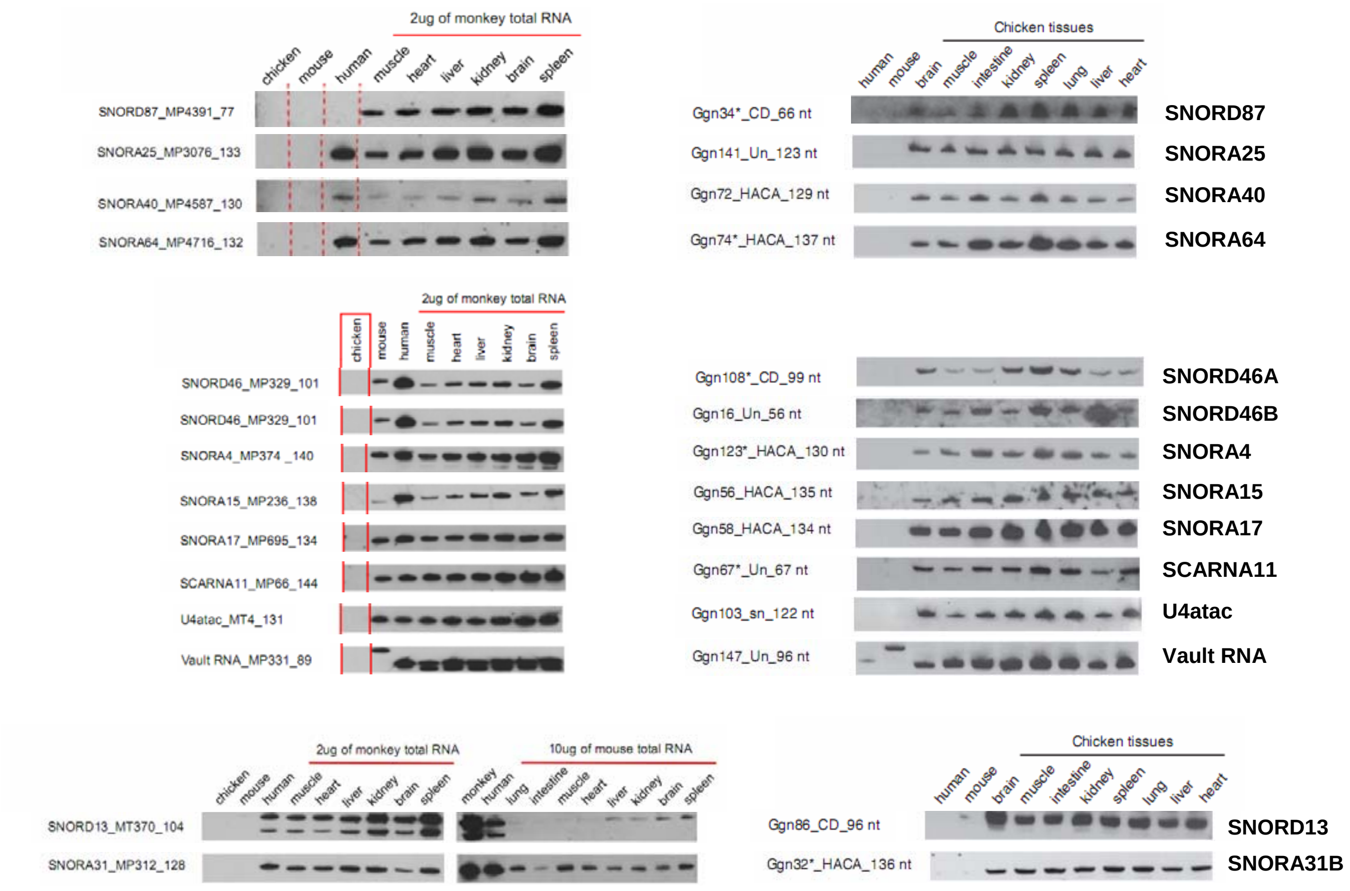

Supplement: Additional file 2 — Controversial results of ncRNA detections in chicken and rhesus monkey (14 extra examples). Hybridization of RNA isolated from different tissues of rhesus monkey, chicken, human, and mouse with rhesus snoRNA probes (left panel; from [18]) and with chicken snoRNA probes (right panel; from [19]). The same RNAs are shown side-by-side. Chicken ncRNAs were cloned by Zhang et al. but not identified as homologs of human snoRNAs [19] (shown on the right). The same RNAs are presented in Table 2. [file 1471-2164-12-543-S2.PDF]
