# Supplementary material for: SNOntology: Myriads of novel snornas or just a mirage?
Source: BMC Genomics. 2011 Nov 3;12:543. doi: 10.1186/1471-2164-12-543 (PMC3349704; doi:10.1186/1471-2164-12-543)
Supplement: Additional file 3 — The majority of chicken ncRNAs cloned and presented as novel RNAs by Zhang at al. [19] are homologs of ncRNAs described previously. Alignments of chicken ncRNAs with the homologs in human or sometimes other vertebrates are shown. GGN sequences are from Zhang et al. [19]. Vault RNA sequence corresponds to the GenBank AF045143 sequence. Other sequences are from snoRNABase [3] and Additional file 4 in this paper. C, D/D', H, ACA, and CAB boxes are underlined; antisense elements are boxed; sequence numbering corresponds to human rRNAs in snoRNABase. In C/D snoRNAs, the nucleotide complementary to the modification site is indicated by the red arrowhead. For the vault RNAs, the secondary structures predicted by mfold [42,43] are shown. The order of ncRNAs is as in Table 2. The SNORD102B transcript has a longer antisense element, and thus can guide the modification of the rRNA nucleotide adjacent to that modified by SNORD102A (marked with black and red arrowheads, respectively) [16]. [file 1471-2164-12-543-S3.PDF]

Additional file 3. The majority of chicken ncRNAs cloned and presented as novel RNAs by Zhang at al. [19] are homologs of ncRNAs described previously. Alignments of chicken ncRNAs with the homologs in human or sometimes other vertebrates are shown. GGN sequences are from Zhang et al. [19]. Vault RNA sequence corresponds to the GenBank AF045143 sequence. Other sequences are from snoRNABase [3] and Additional file 4 in this paper. C, D/D', H, ACA, and CAB boxes are underlined; antisense elements are boxed; sequence numbering corresponds to human rRNAs in snoRNABase. In C/D snoRNAs, the nucleotide complementary to the modification site is indicated by the red arrowhead. For the vault RNAs, the secondary structures predicted by mfold [43, 44] are shown. The order of ncRNAs is as in Table 2. The SNORD102B transcript has a longer antisense element, and thus can guide the modification of the rRNA nucleotide adjacent to that modified by SNORD102A (marked with black and red arrowheads, respectively) [16].

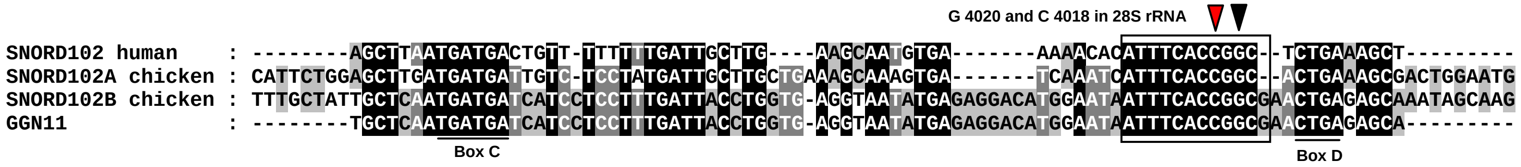

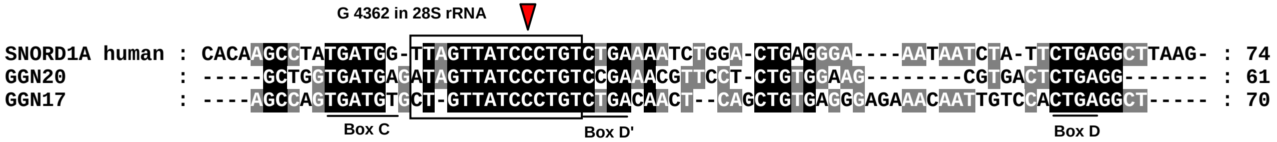

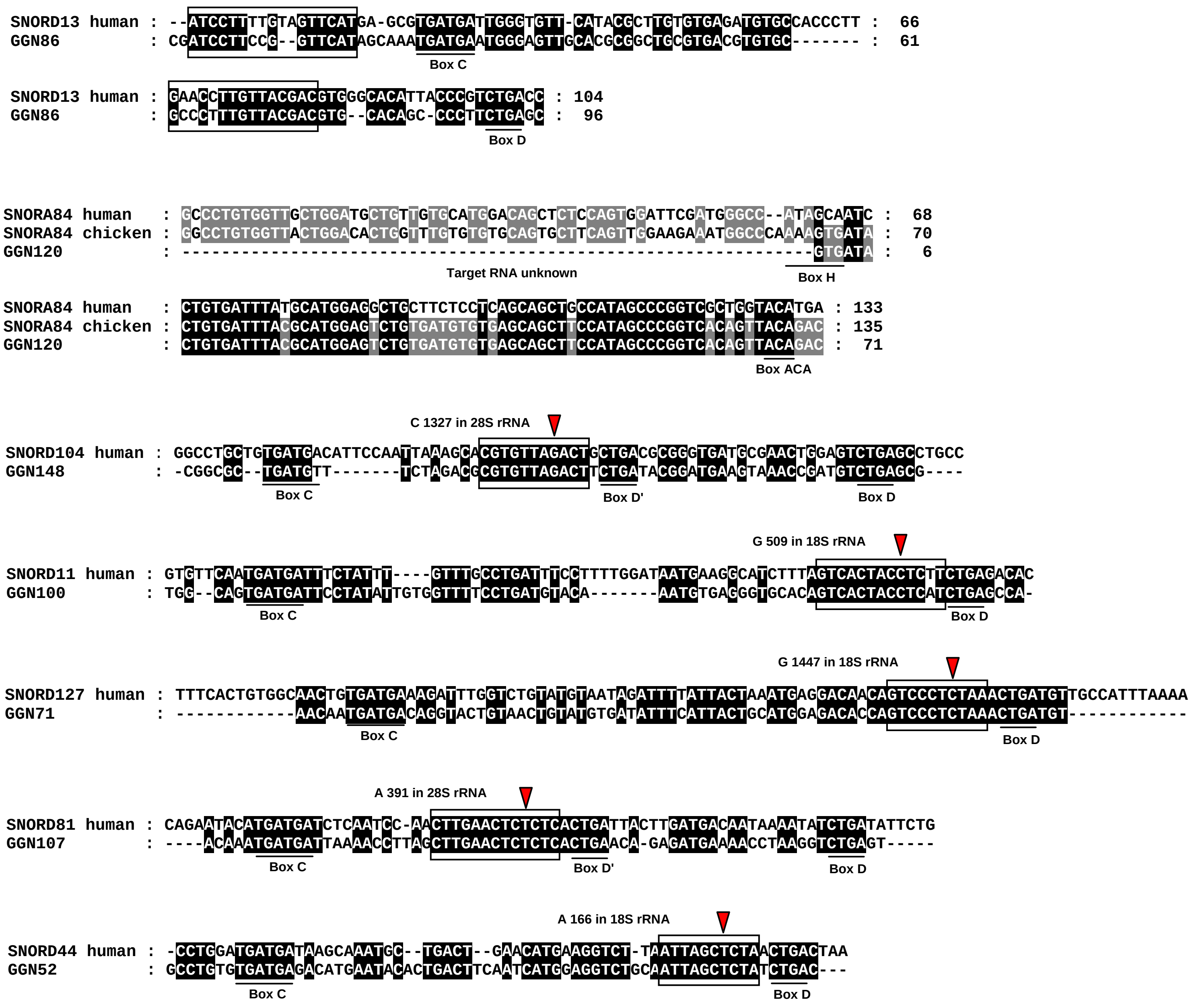
SNORD13 human : --ATCCTTTTGTAGTTCATGA-GCGTGATGATTGGGTGTT-CATACGCTTGTGTGAGATGTGCCACCCTT : 66
GGN86 : CGATCCTTCCG--GTTCATAGCAAATGATGAATGGGAGTTGCACGCGGCTGCGTGACGTGTGC------- : 61
Box C
SNORD13 human : GAACCTTGTTACGACGTGGGCACATTACCCGTCTGACC : 104
GGN86 : GCCCTTTGTTACGACGTG--CACAGC-CCCTTCTGAGC : 96
Box D
SNORA84 human : GCCCTGTGGTTGCTGGATGCTGTTGTGCATGGACAGCTCTCCAGTGGATTCGATGGGCC--ATAGCAATC : 68
SNORA84 chicken : GGCCTGTGGTTACTGGACACTGGTTTGTGTGTGCAGTGCTTCAGTTGGAAGAAATGGCCCAAAAGTGATA : 70
GGN120 : --GTGATA : 6
Target RNA unknown
Box H
SNORA84 human : CTGTGATTTATGCATGGAGGCTGCTTCTCCTCAGCAGCTGCCATAGCCCGGTCGCTGGTACATGA : 133
SNORA84 chicken : CTGTGATTTACGCATGGAGTCTGTGATGTGTGAGCAGCTTCCATAGCCCGGTCACAGTTACAGAC : 135
GGN120 : CTGTGATTTACGCATGGAGTCTGTGATGTGTGAGCAGCTTCCATAGCCCGGTCACAGTTACAGAC : 71
Box ACA
C 1327 in 28S rRNA
SNORD104 human : GGCCTGCTGTGATGACATTCCAATTAAAGCACGTGTTAGACTGCTGACGCGGGTGATGCGAACTGGAGTCTGAGCCTGCC
GGN148 : -CGGCGC--TGATGTT-------TCTAGACGCGTGTTAGACTTCTGATACGGATGAAGTAAACCGATGTCTGAGCG----
Box C
Box D'
Box D
G 509 in 18S rRNA
SNORD11 human : GTGTTCAATGATGATTTCTATTT----GTTTGCCTGATTTCCTTTTGGATAATGAAGGCATCTTTAGTCACTACCTCTTCTGAGACAC
GGN100 : TGG--CAGTGATGATTCCTATATTGTGGTTTTCCTGATGTACA-------AATGTGAGGGTGCACAGTCACTACCTCATCTGAGCCA-
Box C
Box D
G 1447 in 18S rRNA
SNORD127 human : TTTCACTGTGGCAACTGTGATGAAAGATTTGGTCTGTATGTAATAGATTTTATTACTAAATGAGGACAACAGTCCCTCTAAACTGATGTTGCCATTTAAAA
GGN71 : ------------AACAATGATGACAGGTACTGTAACTGTATGTGATATTTCATTACTGCATGGAGACACCAGTCCCTCTAAACTGATGT------------
Box C
Box D
A 391 in 28S rRNA
SNORD81 human : CAGAATACATGATGATCTCAATCC-AACTTGAACTCTCTCACTGATTACTTGATGACAATAAAATATCTGATATTCTG
GGN107 : ----ACAAATGATGATTAAAACCTTAGCTTGAACTCTCTCACTGAACA-GAGATGAAAACCTAAGGTCTGAGT-----
Box C
Box D'
Box D
A 166 in 18S rRNA
SNORD44 human : -CCTGGATGATGATAAGCAAATGC--TGACT--GAACATGAAGGTCT-TAATTAGCTCTAACTGACTAA
GGN52 : GCCTGTGTGATGAGACATGAATACACTGACTTCAATCATGGAGGTCTGCAATTAGCTCTATCTGAC---
Box C
Box D

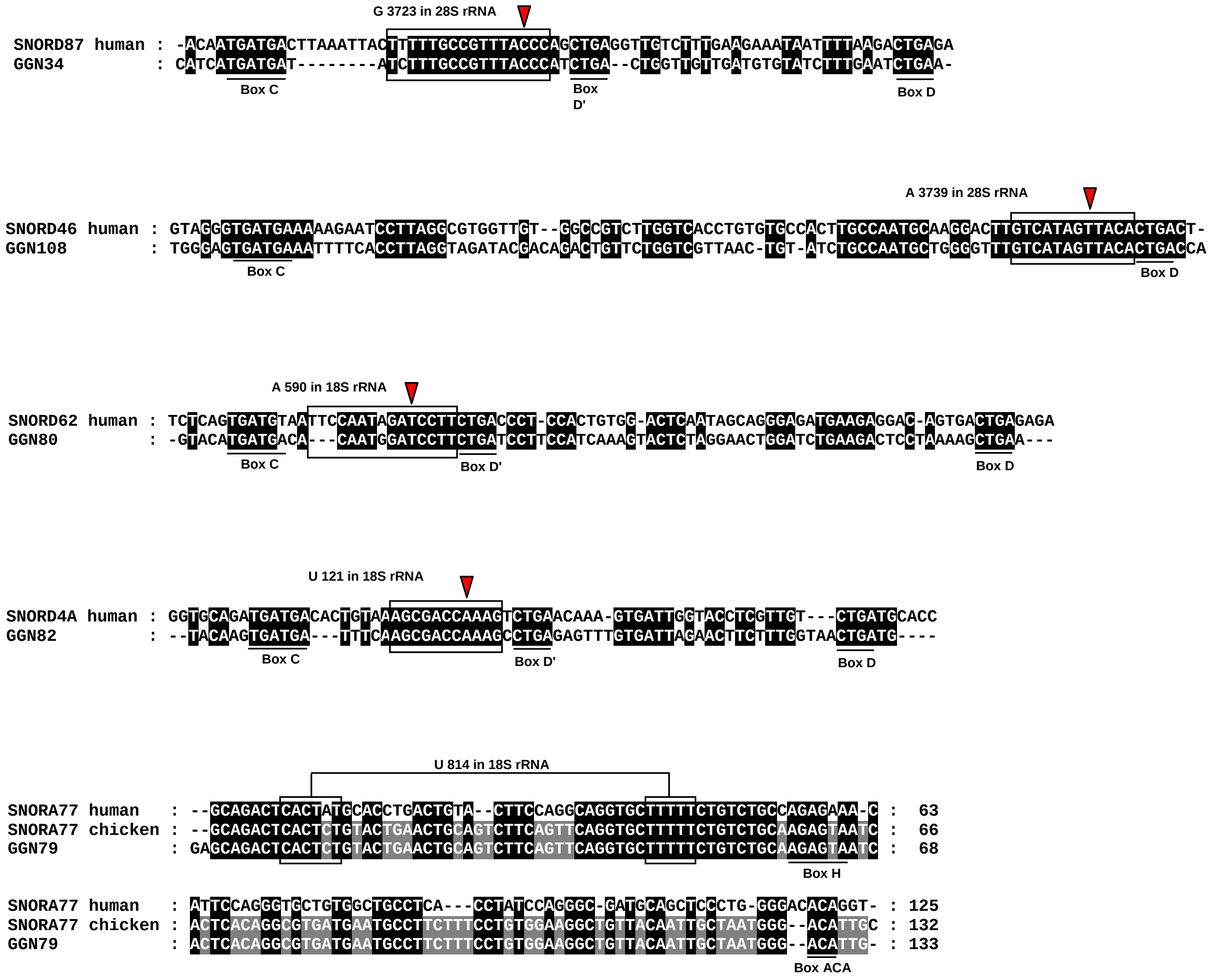
G 3723 in 28S rRNA
SNORD87 human : -ACAATGATGACTTAAATTACTTTTTGCCGTTTACCCAGCTGAGGTTGTCTTTGAAGAAATAATTTTAAGACTGAGA
GGN34 : CATCATGATGAT--------ATCTTTGCCGTTTACCCATCTGA--CTGGTTGTTGATGTGTATCTTTGAATCTGAA-
Box C
Box D'
Box D
A 3739 in 28S rRNA
SNORD46 human : GTAGGGTGATGAAAAAGAATCCTTAGGCGTGGTTGT--GGCCGTCTTGGTCACCTGTGTGCCACTTGCCAATGCAAGGACTTGTCATAGTTACACTGACT-
GGN108 : TGGGAGTGATGAAATTTTCACCTTAGGTAGATACGACAGACTGTTCTGGTCGTTAAC-TGT-ATCTGCCAATGCTGGGTTTGTCATAGTTACACTGACCA
Box C
Box D
A 590 in 18S rRNA
SNORD62 human : TCTCAGTGATGTAATTCCAATAGATCCTTCTGACCCT-CCACTGTGG-ACTCAATAGCAGGGAGATGAAGAGGAC-AGTGACTGAGAGA
GGN80 : -GTACATGATGACA---CAATGGATCCTTCTGATCCTTCCATCAAAGTACTCTAGGAACTGGATCTGAAGACTCCTAAAAGCTGAA---
Box C
Box D'
Box D
U 121 in 18S rRNA
SNORD4A human : GGTGCAGATGATGACACTGTAAAGCGACCAAAGTCTGAACAAA-GTGATTGGTACCTCGTTGT---CTGATGCACC
GGN82 : --TACAAGTGATGA---TTTCAAGCGACCAAAGCCTGAGAGTTTGTGATTAGAACTTCTTTGGTAACTGATG----
Box C
Box D'
Box D
U 814 in 18S rRNA
SNORA77 human : --GCAGACTCACTATGCACCTGACTGTA--CTTCCAGGCAGGTGCTTTTTCTGTCTGCCAGAGAAA-C : 63
SNORA77 chicken : --GCAGACTCACTCTGTACTGAACTGCAGTCTTCAGTTCAGGTGCTTTTTCTGTCTGCAAGAGTAATC : 66
GGN79 : GAGCAGACTCACTCTGTACTGAACTGCAGTCTTCAGTTCAGGTGCTTTTTCTGTCTGCAAGAGTAATC : 68
Box H
SNORA77 human : ATTCCAGGGTGCTGTGGCTGCCTCA---CCTATCCAGGGC-GATGCAGCTCCCTG-GGGACACAGGT- : 125
SNORA77 chicken : ACTCACAGGCGTGATGAATGCCTTCTTTCCTGTGGAAGGCTGTTACAATTGCTAATGGG--ACATTGC : 132
GGN79 : ACTCACAGGCGTGATGAATGCCTTCTTTCCTGTGGAAGGCTGTTACAATTGCTAATGGG--ACATTG- : 133
Box ACA

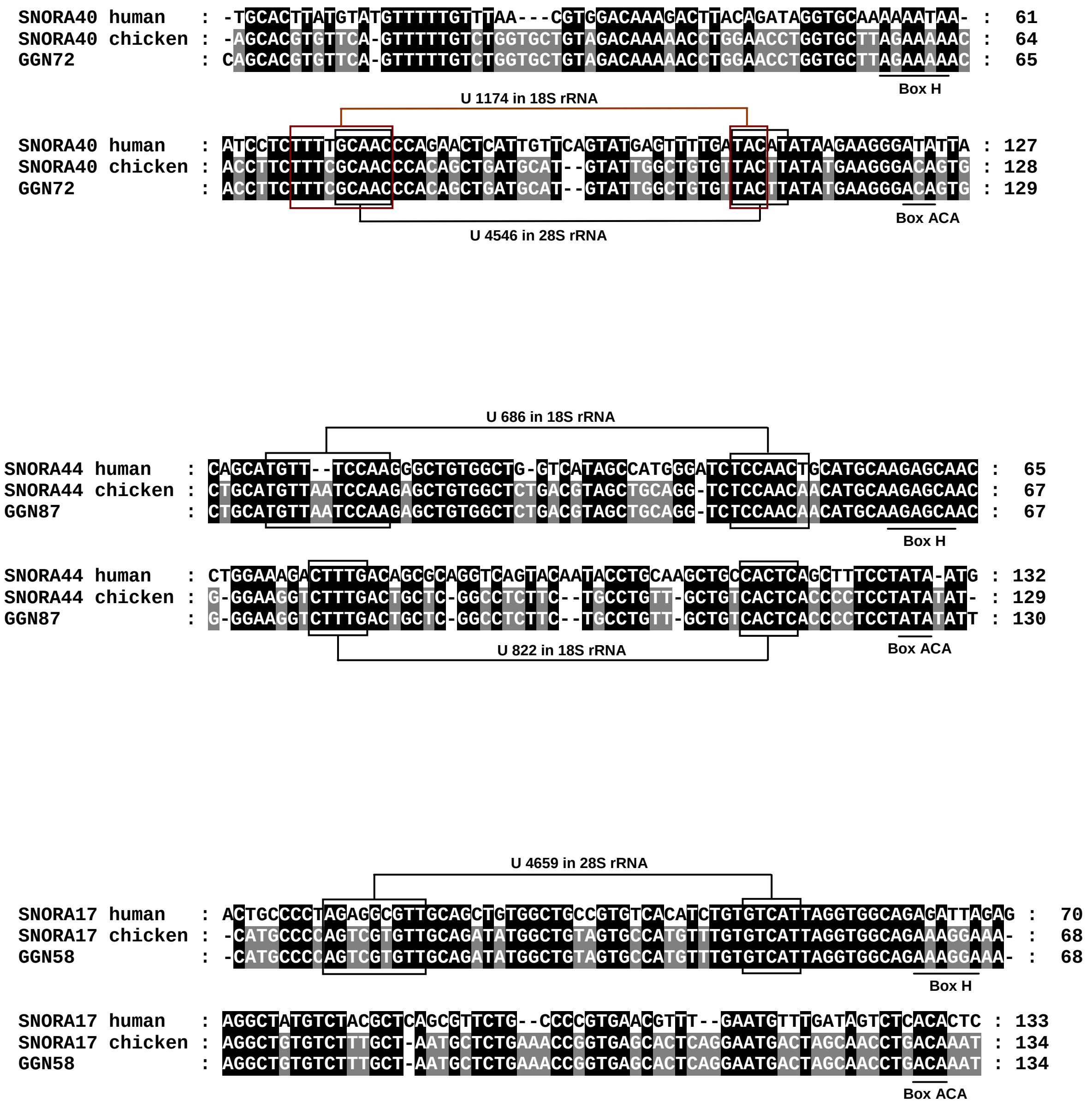
SNORA40 human : -TGCACTTATGTATGTTTTTGTTTAA---CGTGGACAAAGACTTACAGATAGGTGCAAAAAATAA- : 61
SNORA40 chicken : -AGCACGTGTTCA-GTTTTTGTCTGGTGCTGTAGACAAAAACCTGGAACCTGGTGCTTAGAAAAAC : 64
GGN72 : CAGCACGTGTTCA-GTTTTTGTCTGGTGCTGTAGACAAAAACCTGGAACCTGGTGCTTAGAAAAAC : 65
Box H
U 1174 in 18S rRNA
SNORA40 human : ATCCTCTTTTGCAACCCAGAACTCATTGTTCAGTATGAGTTTTGATACATATAAGAAGGGATATTA : 127
SNORA40 chicken : ACCTTCTTTCGCAACCCACAGCTGATGCAT--GTATTGGCTGTGTTACTTATATGAAGGGACAGTG : 128
GGN72 : ACCTTCTTTCGCAACCCACAGCTGATGCAT--GTATTGGCTGTGTTACTTATATGAAGGGACAGTG : 129
Box ACA
U 4546 in 28S rRNA
U 686 in 18S rRNA
SNORA44 human : CAGCATGTT--TCCAAGGGCTGTGGCTG-GTCATAGCCATGGGATCTCCAACTGCATGCAAGAGCAAC : 65
SNORA44 chicken : CTGCATGTTAATCCAAGAGCTGTGGCTCTGACGTAGCTGCAGG-TCTCCAACAACATGCAAGAGCAAC : 67
GGN87 : CTGCATGTTAATCCAAGAGCTGTGGCTCTGACGTAGCTGCAGG-TCTCCAACAACATGCAAGAGCAAC : 67
Box H
SNORA44 human : CTGGAAAGACTTTGACAGCGCAGGTCAGTACAATACCTGCAAGCTGCCACTCAGCTTTCCTATA-ATG : 132
SNORA44 chicken : G-GGAAGGTCTTTGACTGCTC-GGCCTCTTC--TGCCTGTT-GCTGTCACTCACCCCTCCTATATAT- : 129
GGN87 : G-GGAAGGTCTTTGACTGCTC-GGCCTCTTC--TGCCTGTT-GCTGTCACTCACCCCTCCTATATATT : 130
U 822 in 18S rRNA
Box ACA
U 4659 in 28S rRNA
SNORA17 human : ACTGCCCCTAGAGGCGTTGCAGCTGTGGCTGCCGTGTCACATCTGTGTCATTAGGTGGCAGAGATTAGAG : 70
SNORA17 chicken : -CATGCCCCAGTCGTGTTGCAGATATGGCTGTAGTGCCATGTTTGTGTCATTAGGTGGCAGAAAGGAAA- : 68
GGN58 : -CATGCCCCAGTCGTGTTGCAGATATGGCTGTAGTGCCATGTTTGTGTCATTAGGTGGCAGAAAGGAAA- : 68
Box H
SNORA17 human : AGGCTATGTCTACGCTCAGCGTTCTG--CCCCGTGAACGTTT--GAATGTTTGATAGTCTCACACTC : 133
SNORA17 chicken : AGGCTGTGTCTTTGCT-AATGCTCTGAAACCGGTGAGCACTCAGGAATGACTAGCAACCTGACAAAT : 134
GGN58 : AGGCTGTGTCTTTGCT-AATGCTCTGAAACCGGTGAGCACTCAGGAATGACTAGCAACCTGACAAAT : 134
Box ACA

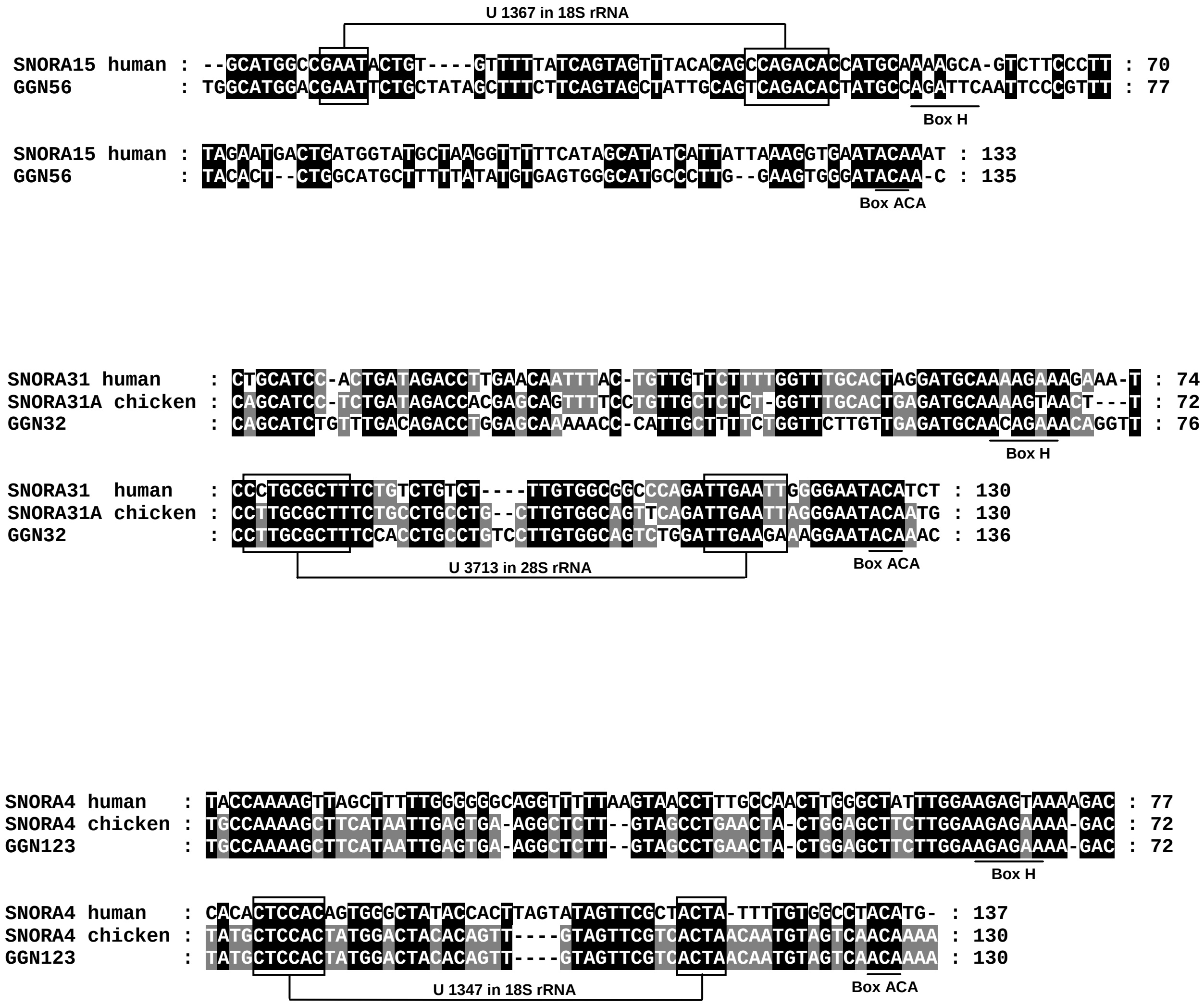
U 1367 in 18S rRNA
SNORA15 human : --GCATGGCCGAATACTGT----GTTTTTATCAGTAGTTTACACAGCCAGACACCATGCAAAAGCA-GTCTTCCCTT : 70
GGN56 : TGGCATGGACGAATTCTGCTATAGCTTTCTTCAGTAGCTATTGCAGTCAGACACTATGCCAGATTCAATTCCCGTTT : 77
Box H
SNORA15 human : TAGAATGACTGATGGTATGCTAAGGTTTTTCATAGCATATCATTATTAAAGGTGAATACAAAT : 133
GGN56 : TACACT--CTGGCATGCTTTTTATATGTGAGTGGGCATGCCCTTG--GAAGTGGGATACAA-C : 135
Box ACA
SNORA31 human : CTGCATCC-ACTGATAGACCTTGAACAATTTAC-TGTTGTTCTTTTGGTTTGCACTAGGATGCAAAAGAAAGAAA-T : 74
SNORA31A chicken : CAGCATCC-TCTGATAGACCACGAGCAGTTTTCCTGTTGCTCTCT-GGTTTGCACTGAGATGCAAAAGTAACT---T : 72
GGN32 : CAGCATCTGTTTGACAGACCTGGAGCAAAAACC-CATTGCTTTTCTGGTTCTTGTTGAGATGCAACAGAAACAGGTT : 76
Box H
SNORA31 human : CCCTGCGCTTTCTGTCTGTCT----TTGTGGCGGCCCAGATTGAATTGGGGAATACATCT : 130
SNORA31A chicken : CCTTGCGCTTTCTGCCTGCCTG--CTTGTGGCAGTTCAGATTGAATTAGGGAATACAATG : 130
GGN32 : CCTTGCGCTTTCCACCTGCCTGTCCTTGTGGCAGTCTGGATTGAAGAAAGGAATACAAAC : 136
U 3713 in 28S rRNA
Box ACA
SNORA4 human : TACCAAAAGTTAGCTTTTTGGGGGCAGGTTTTTAAGTAACCTTTGCCAACTTGGGCTATTTGGAAGAGTAAAAGAC : 77
SNORA4 chicken : TGCCAAAAGCTTCATAATTGAGTGA-AGGCTCTT--GTAGCCTGAACTA-CTGGAGCTTCTTGGAAGAGAAAA-GAC : 72
GGN123 : TGCCAAAAGCTTCATAATTGAGTGA-AGGCTCTT--GTAGCCTGAACTA-CTGGAGCTTCTTGGAAGAGAAAA-GAC : 72
Box H
SNORA4 human : CACACTCCACAGTGGGCTATACCACTTAGTATAGTTCGCTACTA-TTTTGTGGCCTACATG- : 137
SNORA4 chicken : TATGCTCCACTATGGACTACACAGTT----GTAGTTCGTCACTAACAATGTAGTCAACAAAA : 130
GGN123 : TATGCTCCACTATGGACTACACAGTT----GTAGTTCGTCACTAACAATGTAGTCAACAAAA : 130
U 1347 in 18S rRNA
Box ACA

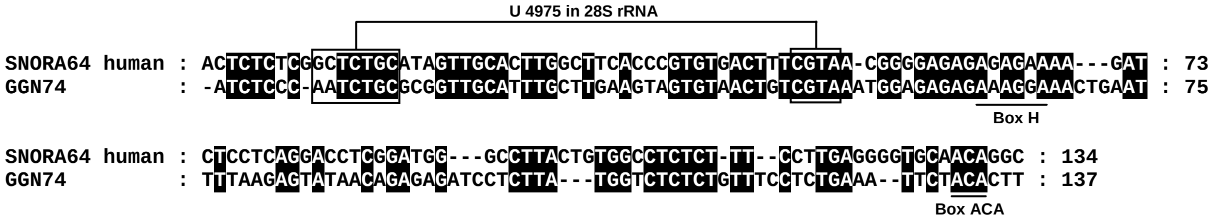
U 4975 in 28S rRNA
SNORA64 human : ACTCTCTCGGCTCTGCATAGTTGCACTTGGCTTCACCCGTGTGACTTTCGTAA-CGGGGAGAGAGAGAAAA---GAT : 73
GGN74 : -ATCTCCC-AATCTGCGCGGTTGCATTTGCTTGAAGTAGTGTAACTGTCGTAAATGGAGAGAGAAAGGAAACTGAAT : 75
Box H
SNORA64 human : CTCCTCAGGACCTCGGATGG---GCCTTACTGTGGCCTCTCT-TT--CCTTGAGGGGTGCAACAGGC : 134
GGN74 : TTTAAGAGTATAACAGAGAGATCCTCTTA---TGGTCTCTCTGTTTCCTCTGAAA--TTCTACACTT : 137
Box ACA

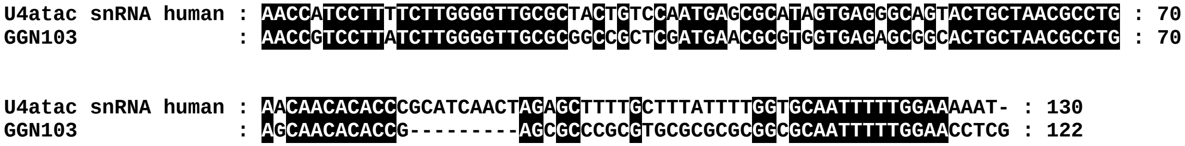
U4atac snRNA human : AACCATCCTTTTCTTGGGGTTGCGCTACTGTCCAATGAGCGCATAGTGAGGGCAGTACTGCTAACGCCTG : 70
GGN103 : AACCGTCCTTATCTTGGGGTTGCGCGGCCGCTCGATGAACGCGTGGTGAGAGCGGCACTGCTAACGCCTG : 70
U4atac snRNA human : AACAACACACCCGCATCAACTAGAGCTTTTGCTTTATTTTGGTGCAATTTTTGGAAAAAT- : 130
GGN103 : AGCAACACACCG---------AGCGCCCGCGTGCGCGCGCGGCGCAATTTTTGGAACCTCG : 122

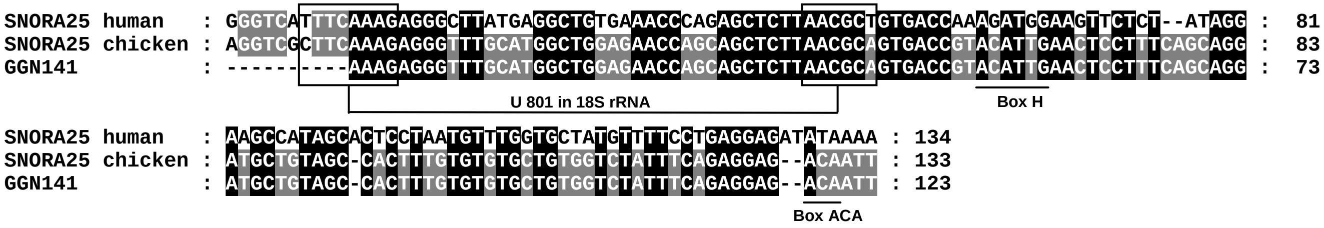
SNORA25 human : GGGTCATTTCAAAGAGGGCTTATGAGGCTGTGAAACCCAGAGCTCTTAACGCTGTGACCAAAGATGGAAGTTCTCT--ATAGG : 81
SNORA25 chicken : AGGTCGCTTCAAAGAGGGTTTGCATGGCTGGAGAACCAGCAGCTCTTAACGCAGTGACCGTACATTGAACTCCTTTCAGCAGG : 83
GGN141 : ----------AAAGAGGGTTTGCATGGCTGGAGAACCAGCAGCTCTTAACGCAGTGACCGTACATTGAACTCCTTTCAGCAGG : 73
Box H
U 801 in 18S rRNA
SNORA25 human : AAGCCATAGCACTCCTAATGTTTGGTGCTATGTTTTCCTGAGGAGATATAAAA : 134
SNORA25 chicken : ATGCTGTAGC-CACTTTGTGTGTGCTGTGGTCTATTTCAGAGGAG--ACAATT : 133
GGN141 : ATGCTGTAGC-CACTTTGTGTGTGCTGTGGTCTATTTCAGAGGAG--ACAATT : 123
Box ACA

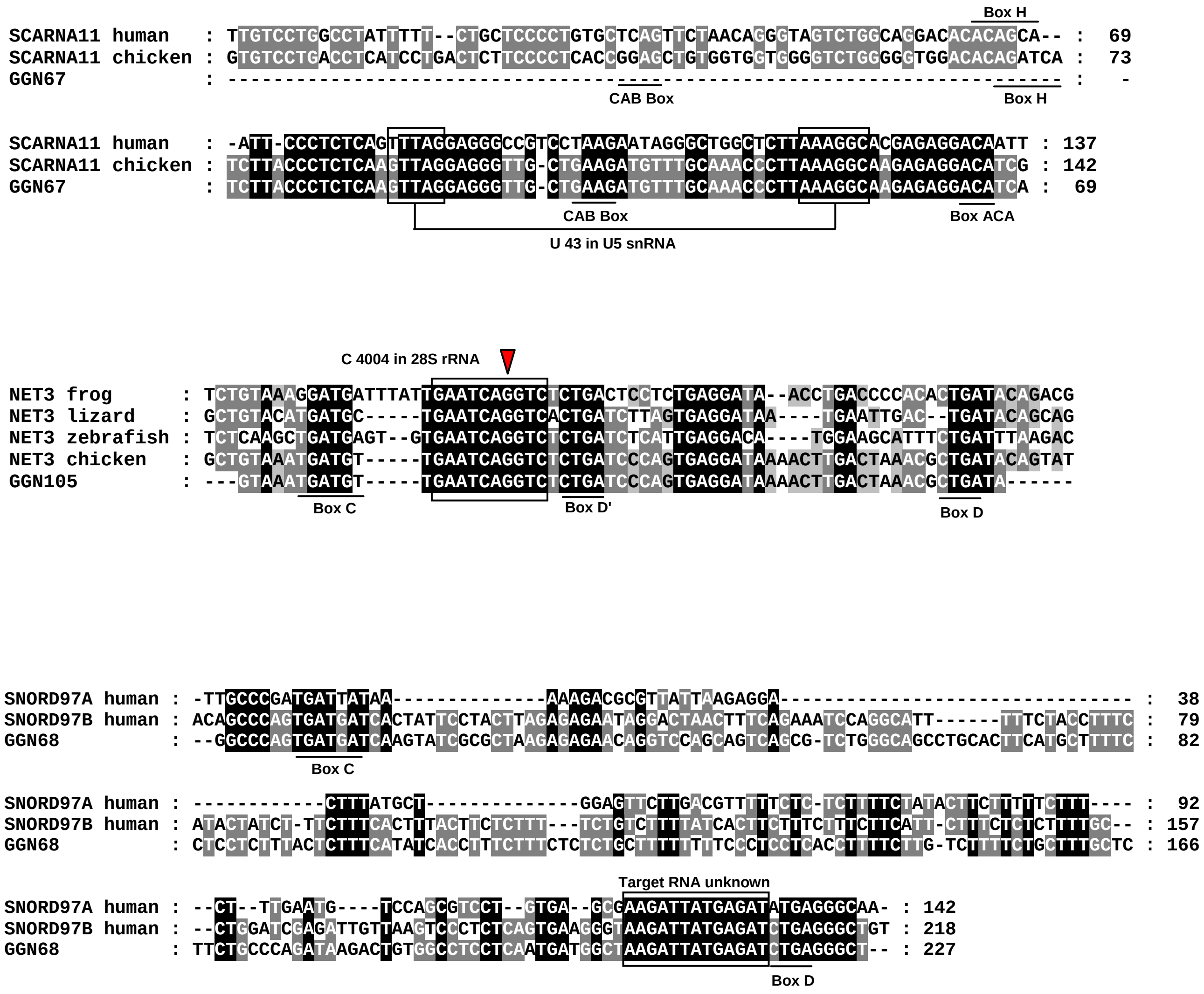
Box H
SCARNA11 human : TTGTCCTGGCCTATTTTT--CTGCTCCCCTGTGCTCAGTTCTAACAGGGTAGTCTGGCAGGACACACAGCA-- : 69
SCARNA11 chicken : GTGTCCTGACCTCATCCTGACTCTTCCCCTCACCGGAGCTGTGGTGGTGGGGTCTGGGGGTGGACACAGATCA : 73
GGN67 : -- : -
CAB Box
Box H
SCARNA11 human : -ATT-CCCTCTCAGTTTAGGAGGGCCGTCCTAAGAATAGGGCTGGCTCTTAAAGGCACGAGAGGACAATT : 137
SCARNA11 chicken : TCTTACCCTCTCAAGTTAGGAGGGTTG-CTGAAGATGTTTGCAAACCCTTAAAGGCAAGAGAGGACATCG : 142
GGN67 : TCTTACCCTCTCAAGTTAGGAGGGTTG-CTGAAGATGTTTGCAAACCCTTAAAGGCAAGAGAGGACATCA : 69
CAB Box
Box ACA
U 43 in U5 snRNA
C 4004 in 28S rRNA
NET3 frog : TCTGTAAAGGATGATTTATTGAATCAGGTCTCTGACTCCTCTGAGGATA--ACCTGACCCCACACTGATACAGACG
NET3 lizard : GCTGTACATGATGC-----TGAATCAGGTCACTGATCTTAGTGAGGATAA----TGAATTGAC--TGATACAGCAG
NET3 zebrafish : TCTCAAGCTGATGAGT--GTGAATCAGGTCTCTGATCTCATTGAGGACA----TGGAAGCATTTCTGATTTAAGAC
NET3 chicken : GCTGTAAATGATGT-----TGAATCAGGTCTCTGATCCCAGTGAGGATAAAACTTGACTAAACGCTGATACAGTAT
GGN105 : ---GTAAATGATGT-----TGAATCAGGTCTCTGATCCCAGTGAGGATAAAACTTGACTAAACGCTGATA------
Box C
Box D'
Box D
SNORD97A human : -TTGCCCGATGATTATAA--------------AAAGACGCGTTATTAAGAGGA-------------------------------- : 38
SNORD97B human : ACAGCCCAGTGATGATCACTATTCCTACTTAGAGAGAATAGGACTAACTTTCAGAAATCCAGGCATT------TTTCTACCTTTC : 79
GGN68 : --GGCCCAGTGATGATCAAGTATCGCGCTAAGAGAGAACAGGTCCAGCAGTCAGCG-TCTGGGCAGCCTGCACTTCATGCTTTTC : 82
Box C
SNORD97A human : ------------CTTTATGCT--------------GGAGTTCTTGACGTTTTTCTC-TCTTTTCTATACTTCTTTTCTTT---- : 92
SNORD97B human : ATACTATCT-TTCTTTCACTTTACTTCTCTTT---TCTGTCTTTTATCACTTCTTTCTTTCTTCATT-CTTTCTCTCTTTGC-- : 157
GGN68 : CTCCTCTTACTCTTTCATATCACCTTTCTTTCTCTCTGCTTTTTTTCCTCCTCACCTTTCTTG-TCTTTTCTGCTTTGCTC : 166
Target RNA unknown
SNORD97A human : --CT--TTGAATG----TCCAGCGTCCT--GTGA--GCGAAGATTATGAGATATGAGGGCAA- : 142
SNORD97B human : --CTGGATCGAGATTGTTAAGTCCCTCTCAGTGAAGGGTAAGATTATGAGATCTGAGGGCTGT : 218
GGN68 : TTCTGCCCAGATAAGACTGTGGCCTCCTCAATGATGGCTAAGATTATGAGATCTGAGGGCT-- : 227
Box D

```
                    C 1703 in 18S rRNA
SNORD43 human : CACAGATGATGAACTTATTGACGGGCGGACAGAAA----------------CTGTGTGCTGATTGTCACGTTCTGATT : 62
GGN46         : TACGTATGAGGATACT-TCGACGGGCGGACATAAGGAAATCGCCTCTGGCGCTTGTTGTTGAGTGTCGAGTCCTGAC- : 76
                     Box C                 Box D'                                   Box D
```

```
                      ((((((.....((((.((((....((((((((((....)))))......((((((((....))))))))))))..)))))))).....)))))).....
vault RNA1 human : ----GGCTGGCTTTAGCTCAGCGGTTACTTCGACAGTTCTTTAATTGAAACAAGCAACCTGTCTGGGTTGTTCGAGACCCGCGGGCGCTCTCCAGTCCTTTT : 98
GGN147           : AAAGGGCCGGCTTTAGCCCAGCGGTTCCTTCGGCAAACAGTTT-CTGAGTTGCGGGCCCCGACTGGG--GTTCGAT-CCCGCGGGCAC-CCCTGGCCCATT- : 96
                   ...(((((((.....((((.((((....(((((.((....)).-)))))...((((((((....)))--))))).. .)))))))).. ..)))))))...
```

```
SNORD46 human    : -------GTAGGGTGATGAAAAA-GAATCCTTAGGCGTGGTTG--TGGCCGTCTTGGTCACCTGTGTGCCACTTGCCAAT :  70
SNORD46A chicken : GAGGCAATGGGAGTGATGAAATTT-TCACCTTAGGTAGATACGACAGACTGTTCTGGTCGTTAAC-TGT-ATCTGCCAAT :  77
SNORD46B chicken : --AGTACGCACGGTGATGATAACTGTGACCTTATGTGGCCTT----GGGAGGCTTGGATGTCTGC-TGG-GT-TCACAAT :  71
GGN16            : ------------------------------------------------GAGGCTTGGATGTCTGC-TGG-GT-TCACAAT :  29
                              Box C
                   A 3739 in 28S rRNA
SNORD46 human    : -GCAAGGACTTGTCATAGTTACACTGACT---------- :  98
SNORD46A chicken : -GCTGGGGTTTGTCATAGTTACACTGACCACGTTGCCTT : 115
SNORD46B chicken : GATTGGGGT--GTCATAGTTACACTGAAGGGCGTGCT-- : 106
GGN16            : GATTGGGGT--GTCATAGTTGCACTGAAG---------- :  56
                                          Box D
```
