# Supplementary material for: SNOntology: Myriads of novel snornas or just a mirage?
Source: BMC Genomics. 2011 Nov 3;12:543. doi: 10.1186/1471-2164-12-543 (PMC3349704; doi:10.1186/1471-2164-12-543)
Supplement: Additional file 4 — Nucleotide sequences of C/D box snoRNA genes in different vertebrate species. Boxes C, D, and D' are shown in gray, and sequences of the antisense elements are highlighted in yellow. The G-T complementarity in the antisense elements or terminal stems is indicated in olive. The 5' and 3' terminal complementary regions forming the stem in snoRNAs are shown in blue. Species-specific complementary substitutions in the antisense elements are marked in pink. Pseudogenes are indicated by Ψ. SNORD115 gene clusters are not listed. They have been found only in eutherian mammals and are available in snoRNABase [3] and UCSC Genome Browser. The following genome assemblies were used: human, March 2006, NCBI Build 36.1; mouse, July 2007, NCBI Build 37; rat, November 2004, version 3.4; dog, May 2005, whole genome shotgun assembly v2.0, cow, October 2007, Baylor release Btau_4.0; horse, January 2007, UCSC version equCab1; opossum, January 2006, monDom4; platypus, March 2007, the v5.0.1 draft assembly; chicken, May 2006, galGal3 version 2.1 draft assembly; lizard, February 2007, Broad Institute AnoCar 1.0; frog, August 2005, whole genome shotgun assembly version 4.1; zebrafish, July 2007, Zv7 assembly; fugu, October 2004, v4.0 whole genome shotgun assembly; tetraodon, February 2004, V7 assembly; stickleback, February 2006, v 1.0 draft assembly; medaka, October 2005, v 1.0 draft assembly. [file 1471-2164-12-543-S4.DOC]

**Additional file 4**. **Nucleotide sequences of C/D box snoRNA genes in different vertebrate species.** Boxes C, D, and D' are shown in gray, and sequences of the antisense elements are highlighted in yellow. The G-T complementarity in the antisense elements or terminal stems is indicated in olive. The 5' and 3' terminal complementary regions forming the stem in snoRNAs are shown in blue. Species-specific complementary substitutions in the antisense elements are marked in pink. Pseudogenes are indicated by Ψ. SNORD115 gene clusters are not listed. They have been found only in eutherian mammals and are available in snoRNABase [3] and UCSC Genome Browser.

The following genome assemblies were used: human, March 2006, NCBI Build 36.1; mouse, July 2007, NCBI Build 37; rat, November 2004, version 3.4; dog, May 2005, whole genome shotgun assembly v2.0, cow, October 2007, Baylor release Btau_4.0; horse, January 2007, UCSC version equCab1; opossum, January 2006, monDom4; platypus, March 2007, the v5.0.1 draft assembly; chicken, May 2006, galGal3 version 2.1 draft assembly; lizard, February 2007, Broad Institute AnoCar 1.0; frog, August 2005, whole genome shotgun assembly version 4.1; zebrafish, July 2007, Zv7 assembly; fugu, October 2004, v4.0 whole genome shotgun assembly; tetraodon, February 2004, V7 assembly; stickleback, February 2006, v 1.0 draft assembly; medaka, October 2005, v 1.0 draft assembly.

| **Species** | **SnoRNA** | **Nucleotide sequence** | **Genomic location** | **Comments** |
| --- | --- | --- | --- | --- |
| mouse | SNORD1A | GCCCATAAGCCTATGATGGATTGGTTATCCCTGTCTGAAGATTTCAGCTGAGGGAAAATACTCTATTCTGAGGCTTAGGGGT | chr.11(+): 116535908- 116535989 |  |
| mouse | SNORD1B | GGTTGAGCTGAGGATGATTTAAGTTATCCCTGTCTGAAATGACACTTTCTGAAGAGTCATGGTTTACTGAGGCTCGACT | chr.11(+): 116535460- 116535538 |  |
| mouse | SNORD1C | GTTGAGCTGAGGATGATTTAAGTTATCCCTGTCTGAAATGACACTTTTGTGAAGAGTCATGGTTTACTGAGGCTCGAC | chr.11(+): 116533819- 116533896 |  |
| dog | SNORD1A | TCTAGCCCCACAAGCCTATGATGGTTTAGTTATCCCTGTCTGAAAATCTCGACTGAAGGGAAACCGTGTATTCTGAGGCTTCAGGGTTTAGG | chr.9(-): 7166645- 7166736 |  |
| dog | SNORD1B | GGGAGCTGAGTCCATGATGATGTAAAGTTATCCCTGTCTGAAGGCAAAGGCCTTCCTGTGGATTATGGATGTCTGAAGCTCAGTTTCC | chr.9(-): 7167204- 7167291 |  |
| dog | SNORD1Ψ | GTATTTGTGGATGATTTAAAGTTATCTTTGTCTTAAAGGGCTCTTTTGTGAAGAGTTTTGATGCGTTGAGGCTCAGCTTTTTTTTTTTttt | chr.9(-): 7169798- 7169874 | A SINE is directly adjacent to the 3' end of the dog SNORD1 pseudogene. |
| opossum | SNORD1A | GAGAGCTGAGCTAATGATGATAGAAAGTTATCCCTGTCTGAACATCTTTTGTGTGGATTTTGATTTGCTGAGGCTCAGCTCT | chr.2 (-): 211816963- 211817044 |  |
| opossum | SNORD1B | AGCTGAGCTGGTGATGATAAGTTATCCCTGTCTGAAAAAAAAATGTGGAGGAAACTTCCTGAAGCTTGGCT | chr.2 (-): 211815682 211815752 |  |
| opossum | SNORD1C | AAGCCAATGATGTTTTGTTATCCCTGTCTGAAAACTTAACTGAGGGGAAAGCAAATCACTTCTGAGGCTT | chr.2 (-): 211815234 211815303 |  |
| platypus | SNORD1A | AGAGCGAGCTAGTGATGAGTAAAAGTTATCCCTGTCTGAACAAAATCCTTTGTGGAATTTTGATTTCCTGAGGCTCAGCTCT | contig 21402 (+): 4514-4595 |  |
| platypus | SNORD1B | GGGAGAGCTGAGTTGATGATGCTACTGAGTTATCCCTGACTGAAGAGGAATATTCCTCCCCTTGTGGAGATCAGACTTGCTGAACTTGAAGCTCTCCC | contig 21402 (+):5288-5385 |  |
| chicken | SNORD1A | TGAGCCAGTGATGTGCTGTTATCCCTGTCTGACAACTCAGCTGTGAGGGAGAAACAATTGTCCACTGAGGCTTA | chr.18(-): 4145894- 4145967 |  |
| chicken | SNORD1B | GCTGAGCTGGTGATGAGATAGTTATCCCTGTCCGAAACGTTCCTCTGTGGAAGCGTGACTCTGAGGCTCAGC | chr.18(-): 4309096- 4309167 |  |
| lizard | SNORD1A | TTTTATAAAGCTGAGCTGGTGATGATACAAAGTTATCCCTGTCTGAAAAGGAAACTCTGCTTGTGGAGTTGTGGAATCTGAAAGCTCAGCTTTAGGGG | chr.19(+): 5474342 - 5474439 |  |
| lizard | SNORD1B | AGCTGAGCTAGTGATGATACAAAGTTATCCCTGTCTGAAAAGGAAACTTTCTGCTTGTGGACTTATGGAATCTGAAAGCTCGGCT | chr.19(+): 5476195- 5476279 |  |
| lizard | SNORD1C | AAAAGCCTATGATGTGATGTTATCCCTGTCTGAAATTTCAAATGTGAGGTGAAAATGAGTATCAACTGAGGCTTTT | chr.19(+): 5479385 5479460 |  |
| frog | SNORD1A | CAGCCTGTGATGTTTTGTTATCCCTGTCTGAAATCATCTGAGGGAAAAAATTTTTTACTGAGGCTT | scaff.178 (-): 1749099- 1749164 |  |
| frog | SNORD1B | AGCCTGTGATGTTGTGTTATCCCTGTCTGAACTACTGTGAGGGACATTTCTTTTTAACTGAGGCT | scaff.178 (-): 1750913- 1750977 |  |
| zebrafish | SNORD1 | AAGCCTGTGATGATAGAAGTTATCCCTGTCTGAAAACTGTGTGGACTTCTGGTAACCTGAGGCTT | chr.3(+): 62755806- 62755870 |  |
| tetraodon | SNORD1A | ACAATGTAGCCAATGATGATACAAGTTATCCCTGTCTGAACTGAAGTGTGGAGTTGTGGTAACCTGAGGCTCAGGTGT | Un_random (-): 39743816- 39743893 |  |
| tetraodon | SNORD1B | GTTTCAGTGCATATGATGATTTTGTTATCCCTGTCTGAACTAATTGTGGACATAATGGTATCTGATCTGCAAAGT | Un_random (- ): 39744266- 39744340 |  |
| mouse | SNORD2 | AAGTGAAATGATGGCAAATCATCTTTCGGGACTGACCTGAAATGAAGAGAATACTCTTGCTGATCACTT | chr.16(+): 23109026- 23109094 |  |
| opossum | SNORD2 | GGGCGTGACATGATGGCCTACCATCTTTCGGGACTGACCTGAAATGGAGAGAACCGCTGTGCTGATCACAACCC | chr.7 (-): 208539176- 208539249 |  |
| platypus | SNORD2A | TGGAAGGGAAATGATGATTTCGTGTTTTACCATCTTTCGGGACTGATGTGGAAATGATGATAAACATGTTTCTCTGATTGCCCTCCTCTG | contig 46167 (+): 6322-6411 |  |
| platypus | SNORD2B | GAGTGACGTGATGGCATACCATCTTTCGGGACTGACCTGAGATGGAGATGGAAAAAAAAAAGTAAAGTCTGCTGATCACTT | contig 213555(-): 513- 593 |  |
| chicken | SNORD2 | TGGAACAAGTGATGTGATGGTACACCATCTTTCGGGGCTGACCTGAAATGGAGAGAACTTGTAATGCTGATCACTTATTTCA | chr. 9 (+): 17296538- 17296619 |  |
| lizard | SNORD2 | TTCCTTGAAAAGTGAGATGATGGTATACCATCTTTCGGGACTGACCTGAAATGGAGAAAAATGTGTAACTGCTGATCACTTGTAAAGGAA | scaff.183 (-): 1037953- 1038042 |  |
| frog | SNORD2A | ATACAAGTGAGATGATGGCATACCATCTTTCGGGACTGAGTTGATGTGAAGAGTTTTTCTGTTTTGCTGATCACTTGTAT | scaff.319 (-): 385001- 385080 |  |
| frog | SNORD2B | GAGCGAAGTGATGGTATACCATCTTTCGGGACTGAATCCCCTGATGACATCTTAATAAAACTGATCGCTC | scaff.319 (-): 381059- 381128 |  |
| zebrafish | SNORD2A | ACAAGTATGAAATGATGGTATGACTATCTTTCGGGACTGACCTTTTGTGGAGAAGACCTTTTCAACTGATCATACTTGT | chr.2(+): 4482629- 4482707 |  |
| zebrafish | SNORD2B | AAAGTATGAAGTGATGGCATCATCTTTCGGGACTGACCTGTTATGGAGATAATCACTAATTTAACTGATCATACTTT | chr.2(+): 4483010- 4483086 |  |
| zebrafish | SNORD2C | CAGGTATGAAGTGATGCACGTCATCTTTCGGGACTGACCTCTCATGGAGAGAACATTTCGACTTACTGATCATACTTG | chr.2(+): 4485941- 4486018 |  |
| tetraodon | SNORD2 | AGCGATATGATGGCATACCATCTTTCGGGACTGACTTTTACAGTGGAGATGTTTTACCCTTAACTGATCGCT | chr.15 (+): 3210779- 3210850 |  |
| mouse | SNORD4 | TGGGTGCATGTGATGACATTATTTAGCGACCAAAGTCTGATAAAGATGATTGTGATCTATTGTCTGATGCACCTA | chr.11(-): 77995186- 77995260 |  |
| dog | SNORD4 | GGGTGCAGATGATGACACTGTTTAGCGACCAAAGTCTGACAAAAATGATTGCTACTTCATTGTCTGATGCACCT | chr.9(+): 46243256 - 46243329 |  |
| opossum | SNORD4A | CAGGGTGCAGATGATGATATCTATAGCGACCAAAGTCTGAGTAAAATGATAGGCAACTTCAGTGTCTGATGCACCCTG | chr.2(+): 506649509- 506649586 |  |
| opossum | SNORD4B | GGGTGCAGGTGATGAGTCAGAGTGACCAAAGCCTGAGCCTCAGTGATTACTCTGCATTGGCTGATGCACCC | chr.2(+): 506650492- 506650562 |  |
| platypus | SNORD4A | CAGGGTGCAGATGATGACATCACAGCGACCAAAGCCTGAGTAAATGATTGCAACCTCACTGGCTGATGCACCCTG | Ultra497 (-): 339354- 339428 |  |
| platypus | SNORD4B | CGGATGCAAATGATGAACTGTAGTGACCAAAGCCTGAACCTTGGTGATGAAGCTTGACCACGTGATGCATCCG | Ultra497 (-): 338094- 338166 |  |
| chicken | SNORD4 | TGGGGTACAAGTGATGATTTCAAGCGACCAAAGCCTGAGAGTTTGTGATTAGAACTTCTTTGGTAACTGATGTACCCCA | chr.19 (+): 5761849- 5761927 |  |
| lizard | SNORD4 | GCACATGATGATGACTTAGCGACCAAAGCCTGACTGGTTGTGATTACAGCTCATAGTCACTGATGTGC | scaff. 2905 (+): 3896- 3963 |  |
| frog | SNORD4A | GTTTGGTGCAGATGATGTTGTAAAGCGATCAAAGTATGAAACTGATGATTCCAATCCTATTACTACTGATGCACCTGGGAAAC | scaff.817 (-): 83843- 83925 |  |
| frog | SNORD4B | ATATTGGGGGAACAGATGATGTGATGTTTATAGCGATCAAAGCCTGAAAAACCTGTGATTGGATATTCATTTACTGATGTGCCCCTAATGT | scaff.817 (-): 82598- 82688 |  |
| zebrafish | SNORD4 | GATGCAAATGATGGGAATTGCGATCAAAGCATGAAAATAATGTGATTACGCTTTTTTAAAACTGATGCATC | chr.21(-): 24552649- 24552719 |  |
| tetraodon | SNORD4 | TGGTGCAAATGATGGTAAATAGCGATCAAAGTATGAAACAAATGATTCCACATTTTAACTACTGATGCACCAAAGACTGC | chr. 7 (-): 3735953- 3736032 |  |
| mouse | SNORD5 | CCAGTTCAGATGATGAATTCAACTGTTCAACTGCTGAATGAGCCAAACATGAACTAACATTAACTCTGACAGAACTGG | chr.9(+): 15118515- 15118592 |  |
| opossum | SNORD5 | GCTTATATACATAGTTCAAATGATGAATTCTTTTGTTCAACTGCTAAGTGATAGAAACATGAACTGAAATGAATTCTGATAGAACCGACTTAAGC | chr.4(+): 255444855 255444949 |  |
| platypus | SNORD5 | GCATTGCCATTCAAGTGATGACATTATTGTTCAACTGCTAAGTGATGTAGATATGAACAAAAAGGAATTCTGAGGGGTTATGGGAATGC | ultra29(-): 2674033- 2674121 |  |
| chicken | SNORD5 | CAGTTCATGTGATGAATATTTCTGTTCAACTGCTGAGTGACTCAGAAATGAACTAAATTTCAATTCTGATTAGAACTG | chr.1(+): 190096779- 190096856 |  |
| lizard | SNORD5 | TGCACTACAGTTCTTGTGATGACATTCCTAGTTCAACTGCTGAGTGATTCCAGAAATGAACTAAAACTAAATACTCTGATTGGAACTGAGTGCA | scaff. 50 (+): 3744730- 3744823 |  |
| frog | SNORD5 | TCTGTTCATATGATGACATTTTAAAGTTCAACTGCTGTATGACTAAAGTATGAACAAATGCATTAACTACTGACTAGAACTGA | scaff.23 (-): 4319115- 4319197 |  |
| mouse | SNORD6 | GATGTTGTGATGATGGGCAAAAATGTTCAACTGCTCTGAATGGGCTGAATGAAAGTAGCCTTTCTGAACATC | chr.9(+): 15119967- 15120038 |  |
| opossum | SNORD6 | GATGTAGTGATGATTGGCAAAATGTTCAACTGCTCTGAAAAGGTGAATGAAAACTGCCTTTCTGAACATC | chr.4(+): 255446995- 255447064 |  |
| platypus | SNORD6 | GGAAATGTTATGATGATTGGCCAAATGTTCAACTGCTCTGAAGAGAGTGAATGAAAACAGCCTTTCTGAACATCC | ultra 29: (-): 2671159- 2671233 |  |
| chicken | SNORD6 | ATTTGTTGTGATGATTGGCAAAATGTTCAACTGCTCTGAAGAGAGTGAGTGAGAATGGCCTTTCTGAACAAAT | chr. 1 (+): 190099445- 190099517 |  |
| lizard | SNORD6 | AATTGTTATGATGATTGGCAAAATGTTCAACTGCTCTGAAAAGGGTGAATGATAAAAAGCCATTCTGAACATGTT | scaff. 50 (+): 3747628- 3747702 |  |
| frog | SNORD6 | ATGGCTTTGTTCTGATGAATGGCAATGTTCAACTGCTCTGAACTAGAGTGACTGACAAAAGCCTCTCTGAACTTAT | scaff.23 (-): 4320626- 4320701 |  |
| zebrafish | SNORD6 | TTAAATTGGGGTTAAGCTTTGTAATGATGATTGGCATAAATGTTCAACTGCTCTTAAAAACCATGAAAGAGGATTTGCCTTTCTGAACATGTACACATTTAA | chr.5(-): 54572835- 54572936 |  |
| tetraodon | SNORD6 | TGTCTGGTTCAACGTGATGATGATGGCTGAATTTGTTCAACTGCGCTGAGACGTGAGGACAGCTGCCTCTCTGAACCAGGCG | Un_random(-):109916859- 109916940 |  |
| human | SNORD7 | CAGCGATGCGATGATGAGTGAAGTAGAGCCTGACCTGGTATTGCCATTGCTTCACTGTTGGCTTTGACCAGGGTATGATCTCTTAATCTTCTCTCTGAGCTGATTCTG | chr.17 (+): 30924784- 30924891 |  |
| mouse | SNORD7 | TGGAGCCATGCAATGATGAGCGAAGTATAGTCTGACCTGGTATTGCCATTGCTTTGGCGCTGGCTCTGACCAGGGTGTGACCCCTTAATCTTCTCTCTGAGCTGATTCTG | chr.11 (+): 83107799- 83107908 |  |
| opossum | SNORD7 | GGTGATACGCAATGATGAGTGAAGTATAGTCTGACCTGGTATTGCCTTGGCTTCTGTTTTGGCTCTGACCAGGGCATGATCACTTAATCTTCTCTCTGAGCTAAACC | chr. 2 (-): 499393019- 499393125 |  |
| platypus | SNORD7 | ACGCCTCAACGCAGTGATGAGCGGAAGTATAGTTTGACCTGGTATGGCCCAGGCTTCCCGGCTTGGGCCCTGACCAGGACATGATCACTTAATCTTCTCTCTGAGCTGGCGT | contig 22580 (+): 11096-11207 |  |
| chicken | SNORD7 | AAGCAATGATGAGTGAAGTATTCTCTGAACTGGTATTGCTGAGACTCTTTGTCTTTGCTCTGACCAGGGCATGATCACGTAATCTTCTCTCTGAGCTT | chr.19 (-): 4431450- 4431547 |  |
| frog | SNORD7 | TCAGCAGTGATGAGCAAGATTTGTTTCTGACCTGGTATTGCTAGATTTGCATCTGCTTTGACCAGGAAGTGATCACTTAATCTTCTCTCTGAGCTGA | scaff. 72 (-):816413- 816509 |  |
| zebrafish | SNORD7A | TTAGCTGCAATGATGAGTGAAAGAATGCATCTTGACTTGGTATTGTTGCTTTTTGGCATCATTGACCAAGGTTTGATTGAGGTTTAATCTTCTCTCTGAGCTGCTAA | chr.5 (-): 54342139- 54342245 |  |
| zebrafish | SNORD7B | AGTTCTGTGCTGATGAGCTGTGATTGTGTCTGACCTGGTATTGCTGTTCCTCCGGGAGTAACTCTGACCAGGGTCTGAAGACTGTAATCTTCTCTCTGAGCAGAGCT | chr.3 (+): 16387599- 16387705 |  |
| zebrafish | SNORD7C | AGTTCTGTGCTGATGAGCTGTGATTGTGTCTGACCTGGTATTGCTGTTCCTCCGGGAGTAACTCTGACCAGGGTCTGAAGACTGTAATCTTCTCTCTGAGCAGAGCT | Zv7_NA456 (-):10554- 10579 |  |
| tetraodon | SNORD7 | ACTCTGCGAGGATGACTGTTGATACGACTGACCTGGCAGTGCCGGCCTCCGGGTCAGCTCTGGCCAGGAAATGACACCTGTTAATCTTCTCTCTGAGCAGAT | chr.13 (-): 7844188- 7844289 |  |
| human | SNORD8  (mgU6-53A) | ACATGTCCCAATGATGAGTTGCCATGCTAATACTGAGCCACCAGGTAGGGCAGTGTTGCCCTGGTTTGGGTGCCAGTGAGTTTAACAAAACTTCTCACATGAAGATCTGAGGGGCATGT | chr.14 (-): 20935287- 20935405 |  |
| mouse | SNORD8 | ACTGTGTCCCGATGATGAGCTGCCATGCTAATACTGAGTCACCAGGCAGGGCAGTATTGTCCTGGTTTGGGTGCCAGTGAGTTTAACAAAACTTCTCATGTGGAGACCTGAGGGACATTGT | chr.14 (-): 52829455- 52829575 |  |
| dog | SNORD8 | ATGCCTCAATGATGAGTTGCCATGCTAATACTGAGTCACCAGGTAGGGCAGTGTTGCCCTAGTTTGGGTGCCAGTGAGTTTAACAAAACTTCTCACGTGGAGACCTGAGGGGCAT | chr.15 (-): 21448385- 21448499 |  |
| opossum | SNORD8 | CATGATCCCCCATGATGAGTTGCCATGCTAATACTGAGTCACCAGGCAGGGTAGTATTACTCTGAGCTGGGTGCCAGTGAGTGGAAGAACACTTCTCACATGGAGACCTGAGGGGCACATG | chr.1 (-): 170008045- 170008165 |  |
| lizard | SNORD8 | GCACACCCATGATGACTAGCCATGCTAATACCGAGCCACCGAGTGGAgctgtctgctttgaactcgggTGTCAGTGAGTTAAGCTTGCTTCTCACAGGAAGACCTGAGGTGTGT | scaff.605 (+):226983- 227096 |  |
| frog | SNORD8 | GGCACAGCCGGGGATGAGTTGCCATGCTAATACTGACGTAGTAGCGCTGAGCGTGAGTGCTCCATGAGCTGACTGCGGCTGAGTGTAAGCAACTGACTGAGGCTGTTCC | scaff.1137 (-):36083- 36191 |  |
| zebrafish | SNORD8 | TTCCAGTGATGAGCCTGCCATGCTAATACTGATGCACCAGTGTTTTGCACTGGGTGAATGTGAGTTAACTTAACTTCTCACACTTGCAGTGTGCTCTGAGGAA | chr.7 (+): 14746266- 14746368 |  |
| tetraodon | SNORD8 | TATCTCCAGTGACGATGCAGCCATGCTAATACTGATGCACCAGACAGGCTTTGTCTGCCTGGGTGCATGTGAGTTCAGCTCAGCTTCTCACGCTGACGTTTTCAGTGTGTGCTGAGGAGATA | chr.9 (+): 4841053- 4841174 |  |
| medaka | SNORD8 | CTCCTGTGATGATTCAGCCATGCTAATACTGATGCATCAGTCAGCTGCATTAACTCTGACTGGATGCAAGTGAGTTCAACTCACCTTCTCACACTGGTGTGATCAGTGTGTGCTGAGGAG | chr. 7 (+): 14021886- 14022005 |  |
| human | SNORD9  (mgU6-53B) | TGCCCCTGTGATGAGTTGCCATGCTAATACGGAGACACCAGGTAGGGAGTTTTACCCTAACTTGGGTGTTGTTGAAATAAACTCTTTCTCGTAAATGCTGAGGGGCA | chr.14 (-): 20930148- 20930254 |  |
| mouse | SNORD9 | TGCCATTTCTCTGTCCCAATGATGAATTGCCATGCTAATACTGAGCCACCAGCCAGGCTATTCCTGCCTGGTTTGGGTGCCAGTGAGTTTAACAATACTTCTCACGTGGAGACCTGAGGGACAGAAGGAAAGGCA | chr.1 (+): 129271695- 129271829 |  |
| dog | SNORD9 | TGCCCCTGTGATGAGTAGCCATGCTAATATTGAGACATCAGATACAGGATGTTGTTGAGATAAATTCTTCTCTTATATTCACCTGAGGGGCA | chr.15 (-): 21442746- 21442837 |  |
| opossum | SNORD9 | TGTGCCTCTGTGATGAGTTGCCATGCTAATACTGAAGCACTAGGTAGGGCAATGATGCCCTGCACTGAGTGTTACTGAGTTAAACTCCACTTCTCTTATAAGACATCTGAGGGGCACA | chr.1 (-): 170001931- 170002048 |  |
| lizard | SNORD9 | GGTTGCCTTTGATGACTTGCCATGCTAATACTGAACCCCAGTTGGCCAATGGCTCTTCTGTGGAGTTTTGTGAATGGATCCCTTCTTGGACTGAGGCACC | scaff.605 (+):231826- 231925 |  |
| zebrafish | SNORD9A | TGTTGTGTTTCCAATGATGAGCCTGCCATGCTAATACTGATGCACCAGAGTTTGACTGTGTCTCACACTGGGTGTAAGTGTGTTCAACTAAACTTCTCACACTTGCAGTGTGCTCTGAGGAAACAGTACA | chr. 7 (+): 14748738- 14748867 |  |
| zebrafish | SNORD9B | TTTTGGTGTTTTCTGATGATGAGCCAGCCATGCTAATACTGATGCACCAGTCTGACTGTGTCTCTCTGGGTGTAAGTGAGTTCAACACAACTTCTCACACTCGTGGTGTGATCTGAAGATGAACACAAAA | chr. 7 (+): 14750586- 14750715 |  |
| mouse | SNORD10 | TTGCTCTGTGATGAACCCCATGCGTGTCATCTGAGCCTGGCTTCCCTGTTGTTACCTTAGCCCAGGTAGTGTTCTACTTACCAGGGCTTTTGCCTGGCTTATCAGGGAAGCTTTGGGCAAAGGATCAGTCCTTGTATTCTGAGAGCAG | chr.11(-): 69482352- 69482499 |  |
| opossum | SNORD10 | ATTTGCTCTGTGATGATCCCCCATGCGTGTCATCTGAGCTCAGCTTCCCCTGATCTCTCAGTTCCAAGTGGGTGTTTTGCCCTCTGGTCTCTGGGCTGGCAGTGGCTTCTGGGCACATGATCATAATTTAACCCTGAGAGCAAAT | chr. 2 (+): 280676281- 280676425 |  |
| lizard | SNORD10 | CAAACGGCTGCTGTTCCGTGATGATTGGCCTCATGCGTGTCATCTGACCCCCTCGCTGCCCCACAGTCTCCCTTCCGGAAGGCGTTCTGCCTTGTTGGGGGGAGATGTGGGGTTTGCTTGGGGGAAGTGATCATAACCTAACAAGGTCCTGAGGACAGCGGCTTCCCTTTG | scaff. 1440 (-):11666-11836 |  |
| frog | SNORD10 | GGGGGCTCAGTGTAGTGATGATATCCCATGCGTGTCATCTGATGCCCACGTCTCTCTCTGCCCTTGGCCTGCGCAGCCTGCGTCCTGCGGGTTGGGTTTGGCTAGAGCAGAGGAGTCTGGGAAATGATCTGACTGTCTATCTGAATACTTGCCCCC | scaff.1384 (+):13283- 13438 |  |
| zebrafish | SNORD10 | GCTGTTTTGTGATGATCCCCATGCGTGTCAACTGATCCCAGACTCCCTGCAGTGCTCAATGTCTGATGAGCGTTTTGCTCAACAGTGCATTTTGAGCCTGTAGGACTCTGGGAACTTGATCTTAACTTTTAAATCTGAAAACGGC | chr.7(-): 17494360- 17494504 |  |
| mouse | SNORD11A | GACCATGTGTTCAATGATGATTTCTGATGCTTTGCCTGAGTTCTCTTTGAATAATGAGGGCAGCTTCAGTCACTACCTCTTCTGAGACACCGTGGTC | chr.1(+): 59761628- 59761724 |  |
| mouse | SNORD11B | AGGTGGCAATGATGATTTTTCTTATTTGTTCACCTGACAATATATGAAGGTGTTCAGTCACTACCTCATCTGATGCCATCT | chr.1(+): 59761102- 59761182 |  |
| opossum | SNORD11A | GATGGCAGTGATGATGATTTTATATTGATTCTCACCCGATTTACAAATATGAGGGTATATAGTCACTACCTCATCTGATGCCATC | chr.7 (-): 195881066 195881150 |  |
| opossum | SNORD11B | TGAAGTGATCTGTGATGATTTTAATTTGTTTGCCTGACTTCCTTTTCAATAATGAGGGCAACTTTAGTCACTACCTCTTCTGAGACACTTCA | chr.7(-): 195879033 195879124 |  |
| platypus | SNORD11A | TTTCTCGATGGCGATGATGATTTCTAGATTTTTGCTCTCCTGATGTAGCCGTGAGGGTGCAAAGTCACTACCTCATCTGATGCCATCTCGGAA | contig10764 (+): 9381- 9473 |  |
| platypus | SNORD11B | GGGCCCAGTGATCGGTGATGATTTGGATTTGTTTGCCTGACTTCCTTTGTGGATAATGAAGGCAACTCAGTCACTACCTCTTCTGAGACACTTTGGACCC | contig10764 (+):11346 - 11445 |  |
| platypus | SNORD11C | GCCTACTCCTGGGGAGTGGTCTGTGATGACTGTTACCTCTTATTGCCTGACTCCTTTCGAAAGGAAGATGAAGGCAATGTTAGTCACTACCTCTTCTGAGACTCTCCTCAGTAGGC | contig10764 (+): 14810- 14925 |  |
| chicken | SNORD11A | GATGGCAGTGATGATTCCTATATTGTGGTTTTCCTGATGTACAAATGTGAGGGTGCACAGTCACTACCTCATCTGAGCCATC | chr.7 (+): 12850073- 12850154 |  |
| chicken | SNORD11B | TAGTAGTCTGTGATGATGTAGTTCTTGGTTGGCCTGAATCCTTTTTGGAATATGAGGGCAACTTAAGTCACTACCTCTTCTGAGACACTG | chr. 7 (+): 12851161- 12851250 |  |
| lizard | SNORD11A | AGAAGCATTCAATGATGATTCATTTCTGAAGTTTGCCTGATTTCATCTTTGAAAAGTGAAGGCAACTCTAGTCACTACCTCTTCTGAGATGCTTCT | scaff.881 (+):350218- 350313 | Guides modification of G-509 in 18S rRNA (according to human numbering) |
| lizard | SNORD11B | TTCTTGGTGGCAGTGATGATTCTTGTGATGCTGCATTCCTGATATACAAACTGAAGGGCTGCATAGTCACTACCCTCTTCTGATGCCATCTGAA | scaff881 (+):348367- 348460 | Lizard SNORD11B RNA can potentially guide modification of another site in rRNA (corresponds to G-2745 in human 28S rRNA) |
| frog | SNORD11A | GTGGTCAGTGATGATGTTTTATTCCTAATTGCCTGATAACCCATGAGGGCAATTTTAGTCACTACCTCATCTGAGACCAC | scaff.312 (+): 50366- 50440 |  |
| frog | SNORD11B | AGTTTGATCAGTGATGATGATTTTTGTCTAATTGCCTGATACTTAAATGAGGGCAATTTTAGTCACTACCTCATCTGAGACAAACT | scaff.312 (+): 46331- 46416 |  |
| frog | SNORD11C | TTGGGTCAATGATGATTTCAACTTTTGCCTGATTTTCCTGAAGGAAATAGCTGTCACTACCTCATCTGAGCCCCAA | scaff.312 (+): 52145- 52220 |  |
| zebrafish | SNORD11A | CAAGCGGTGATGAAGATGACTTGGATGTCACAGTCCTGAAAATAACATGTGGGTTTTTTGTCACTACCTCTTCTGAGCTTG | chr.6(-): 7938840- 7938920 |  |
| zebrafish | SNORD11B | TGTGAGAAACAACAAGCTGTGATGATGATGACCATGCGTCATTTTGCTGAAAAATCATGAGGCTTTTTTGTCACTACCTCATCTGAGCTTGTTTATTTCTCATA | chr.6(-): 7943027- 7943128 |  |
| zebrafish | SNORD11C | TGAGAAACAACAAGCTGTGATGATGATGACCATGCGTCATTTTGCTGAAAAATCATGAGGCTTTTTTTGTCACTACCTCATCTGAGCTTGTTTATTTCTCA | chr.6(-): 8041780- 8041880 |  |
| zebrafish | SNORD11D | CAAGCGGTGATGAAGATGACTTGGATGTCACAGTCCTGAAAATAACATGTGGGTTTTTTGTCACTACCTCTTCTGAGCTTG | chr.6(-): 7938840- 7938920 |  |
| fugu | SNORD11A | gacagtggtgatgatgatgacatttGTCTCTATCCTGAAAGCTAATGTGTAGGTTTTCAGTCACTACCTCATCTGAACTGTC | Un (+): 90193254- 90193335 |  |
| fugu | SNORD11B | ACCCCTGCGGTGATGATGAcgacatttctgtcttttttctgaaaatgaTTGATGTTTTTAAGTCACTACCTCATCTGAGCTTGT | Un (+): 90193579- 90193662 |  |
| mouse | SNORD12A | CAGGCATGTGTGATGACACAACTTTTTTCCCCATCAGATCGACCATGTTGATCACATTCTTTTAAGCCAGTATGTCTGACATGCCTG | chr.2(+): 166890499- 166890585 |  |
| mouse | SNORD12B | GGCTGGTGAACTGATGATATCATTTCTTTCCCCGTCAGATCGACCCTGTTGATCTCAAATACTAATTGCCAGTTTTGTCTGATGCATCAGCC | chr.2(+): 166890775- 166890866 |  |
| mouse | SNORD12C | GCTGGTGTAAATGATGAACTCACTTTTTTCCCCGTCATATCGACAGTGCTGATGTTTTAAAACATTTGCCAGTTTGTCCTGATAAACACCAGC | chr.2(+): 166888973- 166889065 |  |
| opossum | SNORD12A | GGTGCAAATGATGACTTGACTTTTTTCCCCATCAGATCGACCTTGCTGATCTCTTTGAATTTTTGCCAGTTTGATTCTGATGCACC | chr.1 (-): 497005780- 497005865 |  |
| opossum | SNORD12B | GCTGGTATATATGATGACTAAACTTTTTTCCCCATCTGATCGACTATGTTGATCTGTACTACTACTATGCCAGTTTTTTCTGATATACCAGC | chr.1 (-): 497004390- 497004481 |  |
| opossum | SNORD12C | GCTGGTATGTATGATGACTAAACTTTTTTCCCCATCAGATCGACCATGTTGATCCTAATTAATCTTTGCCAGTTTTTTTCTGACATACCAGC | chr.1 (-): 497003982- 497004073 |  |
| opossum | SNORD12D | CTTCAGGTCTGTGGTTACATGATGACTAAAACTTTTTTCCCCAACTGATCGACAATGCTGATCAGGAATACATTTAAGCCAAAACTTGTCTGATTTCCATCAGATCCTGGAG | chr.1(-): 497003556- 497003667 |  |
| platypus | SNORD12A | CTGGTGTAAATGATGACTAAACTTTTTTCCCCATCAGATCGACTGTGTTGATCTCATCGCTCTTTGGCCATTTTCATTCTGATACACCAG | Ultra516 (+):1568423- 1568512 |  |
| platypus | SNORD12B | GCTGGTGTATGTGATGACGAAACTTTTTTCCCCATCAGATCGACCATGTTGATCTCAATTCCTCTTTGCCAGGTATTTTCTGATACACCAGC | Ultra516 (+):1569155- 1569246 |  |
| platypus | SNORD12C | CTGGTGTATATGATGACTGCACTTTTTTCCCCATCAGATCGACCATGTTGATCTCACTACACTATGCCAGGTTCAATCTGATGCACCAG | Ultra516 (+):1569553- 1569641 |  |
| chicken | SNORD12A | CTGCTGGTGTAGGTGATGACTGAACTTTTTTCCCCATCAGAGCGACAGTGTTGATTACTCATCACTCTAGCCAGGTCTTGTCTGATGCACCAGCAG | chr.20 (+): 6433894- 6433989 |  |
| chicken | SNORD12B | CTGCTGGTGTAGGTGATGACTGAACTTTTTTCCCCATCAGAGCGACAGTGTTGATTACTCATCACTCTAGCCAGATCTTGTCTGATGCACCAGCAG | chr.20 (+): 6434972- 6435067 |  |
| chicken | SNORD12C | CCTGCTGGTGTAGGTGATGACTGAACTTTTTTCCCCATCAGAGCGACAGTGTTGATTACTCATCACTCTAGCCAGATCTTGTCTGATGCACCAGCAGG | chr.20 (+): 6435963- 6436060 |  |
| chicken | SNORD12D | TCTGTGGTATATGATGACTTCAAACTTTTTTCCCCATCAGATCGGCAATGCTGATACAGACTTGTGTTTAAGCCAGATTTGTCTGATTCCACAGA | chr.20 (+): 6436619- 6436713 |  |
| lizard | SNORD12A | GCTGATGTATATGATGACTAAAACTTTTTTCCCCATCAGATCGACActgttgattttgctatttttagcCAGATTTGTCTGATACATCAGC | scaff.15 (-): 1346026 - 1346116 |  |
| lizard | SNORD12B | ATTCAGTGGTATATGATGACTTTAtcttttttccccatcatatCGGTAATGCTGATAAGATGTGAATTCTAGCCAGATGGTCTGATTCCGCTGAAT | scaff.15 (-): 1344704- 1344799 |  |
| lizard | SNORD12C | ATTCAGTGGTATATGATGACTTTAtcttttttccccatcatatCGGTAATGCTGATAAGATGTGAATTCTAGCCAGATGGTCTGATTCCACTGAA | scaff. 15 (-): 1344253 - 1344347 |  |
| frog | SNORD12A | TCTGGGCCTATTGCTGACATGCATGATGACCAAACTTTTTTCCCCAACAGATCGACAGTGTTGACTTTGTTGCTATTAAGCCAGTTTTGTCTGATATGTCTGTAGAATAGCCCAGA | scaff.48 (+): 2921987- 2922102 |  |
| frog | SNORD12B | TCTGCTGTATGTGATGACTAGACTTTTTTCCCCAGCAGATCGACACTGTTGACCATAATCTGATTTAAGCCAAACATTGTCTGATACACAGAATAGA | scaff.48 (+): 2922406- 2922502 |  |
| frog | SNORD12С | TTGCCGTGCGATGTCTGTATGATGACTAAACTTTTTTCCCCAGCAGATCGACACTGTTGACTTGCAATGCTTTTAAGCCAGTTTTGTCTGATACATCGGCAA | scaff.48 (+): 2921691- 2921792 |  |
| zebrafish | SNORD12A | TCTGGTGCACGTGATGACTAACCTTTTTTCCCCAGCAGATCGACTCTGTTGACCTTTAAAACGAAAATAAGCCAAATTATCTGAACAACCAGA | chr.11(+): 6260095- 6260187 | SNORD12A-D genes are located in introns of an unannotated gene corresponding to an EST cluster (EH443988 etc.) |
| zebrafish | SNORD12B | TTATCTGGTGCACGTGATGACTAACCTTTTTTCCCCAGCAGATCGACTCTGTTGACTTGTAACACGAAAAATAAGCCAAATTATCTGAGCAACCAGATGG | chr.11(+): 6260365- 6260464 |
| zebrafish | SNORD12C | TTCTGGTAGCAATGATGACTTAATCTTTTTTCCCCAGCAGATCGACTCTGTTGACTTGTGAAACGAAAATAAGCCAAATTATCTGAGCAAACTGATGG | chr.11(+): 6259809- 6259906 |
| zebrafish | SNORD12D | GGTGCACATGATGACTGACCTTTTTTCCCCAGCAGATCGATAGTGTTGACTCCCTTAATCAATTTAAGCCAATTTATCTGAAGCACC | chr.11(+): 6260641- 6260727 |
| zebrafish | SNORD12E | ttgtggttaatgatgttgctctttTTTCCCCGTCTTATCGACTATGCTGATTCCTCTTGAAAATAAGCCAAATCCTCTGAaaccaccacga | chr.2(-): 24614778- 24614868 |  |
| tetraodon | SNORD12A | TGTGGTTAATGATGTGCTCTCTTTCCCCGACTTATCGACTATGCTGACACACTCTCGTCTTATAAGCCAAATGTTGCTGAAACCACA | chr.6 (+): 1992778 - 1992864 |  |
| tetraodon | SNORD12B | CAGGAATGTGGTTGATGATGTCCAACTTTTTTCCCCATCTTATCGACCATGGGGACTCCAAACCAATTTAAAGCCAATTGTGTCTGAAACCACGTTCTG | chr.6 (+):  1993145- 1993243 |  |
| tetraodon | SNORD12C | AGCTGTGGTTGATGATGCAAGCTTTTTTCCCCGTCTTATCGACTATGGTGAAAAAAATCAGAAAACTAAGCCAGTTGAGTCTGAAACCACATGCT | chr.6 (+):  1993794- 1993888 |  |
| tetraodon | SNORD12D | GTGGTTGATGATGCACTCTTTCCCCGTCTAAGCGACCATGGCGAAGACCCAGGTCTACTTTAAGCCAAACATGTCTGAAACCAC | chr.6 (+):  1994252- 1994335 |  |
| human | SNORD14C | GTGACtcgctgtgatgagtgattgttaaacattcgtagtttccaccaaaagcttggctaatgatggcaacaccttccttggatgtctgagcgaGTGAT | chr.11(-): 122435248- 122435345 | These copies are localized in introns of the HSPA8 host gene.  SNORD14E gene has a substitution in box D. |
| human | SNORD14D | GGGTCACAatgatgaatggtccaaaacattcgcggtttccaccagaattcaaggtgttggcaactaccttccttggatgtctgagtgaCC | chr.11(-): 122433993- 122434082 |
| human | SNORD14E | tcgctatgatgatggattccaaaaccattcgtagtttccaccagaaagtcttatgttggccagttccttccttggatgtttgagcga | chr.11(-): 122434827- 122434913 |
| mouse | SNORD14A | AGCtcacagtgatgatggtgttccaacattcgcagtttccaccagaaggactttccatgttgggttgaccttccttggatgtctgagtgaGCT | chr.7(-): 123476682- 123476774 | The gene is located in an intron of  RPS13. |
| mouse | SNORD14B | ATtcgctgtgatgatggattccaaaaccattcgtagtttccaccagaagtactgtgttggctagttccttccttggatgtctgagcgaaT | chr.9(+): 40611664- 40611753 | The genes are localized in introns of HSPA8. |
| mouse | SNORD14C | AAGTTCCtcgctgtgatgatggattccaaaaccattcgtagtttccaccagaaatgctgtgttggctagttccttccttggatgtctgagcgaaCTT | chr.9(+): 40612071- 40612167 |
| mouse | SNORD14D | GGGtcacaatgatgatgaatggtccaaacattcgcggtttccaccagaacgcaaggcagtgttggcagttaccttccttggatgtctgagtgaCTC | chr.9(+): 40612828- 40612923 |
| dog | SNORD14A | AGGCTGCTtcgctatgatgattgattctttaaacattcgtagtttccaccaaaagcttagctaatgatggtccaacttccttggatgtctgagcgaTTGACCT | chr.5(+): 14299879- 14299981 | The genes are localized in introns of HSPA8. |
| dog | SNORD14B | GGAAAGACTTGTCCcgctgtgatgatatgacctaaaccattcgtagtttccaccagaagattttgtgttggccaactccttccttggatgtctgagcgaTCAGTCTTTCC | chr.5(+): 14300304- 14300413 |
| dog | SNORD14C | AAATACAGGTCACgatgatgaatggtccaaacattcgcggtttccaccagaattccacgtgttggcaactagcttccttccttggatgtctgagtgaCCCAACATTT | chr.5(+): 14301191- 14301297 |
| opossum | SNORD14A | GTAGGTTGGCttgctgtgatgatattggtaccaaaaccattcgtagtttccaccagaagcttgctaatgatggcaagaaccttccttggatgtctgagcgaGCTTAATTTGT | chr. 4 (+): 225033375- 225033486 |  |
| opossum | SNORD14B | TCATttgctgtgatgaatgttccaaaaccattcgtagtttccaccggaagcttgctgatgatggccaagtaccttccttggatgtctgagcgaaAGA | chr. 4 (+):  225034547- 225034643 |  |
| opossum | SNORD14C | GGAAAAGCAAAAGCAGTGGCTgctatgatgaaagtgatgcaaaaccattcgtagtttccactagaagttgaaaaactaatgttggcattaagttccttccttggctgtctgagcgaCCACTGCTTTTCTTTCC | chr. 4 (+):  225035350- 225035482 |  |
| opossum | SNORD14D | ATGGttgctgtgatgaaagtgttcccaaaaccattcgtagtttccaccagaagtcttgacttgtgttggcaagatccttccttggatgtctgagcgaCCAT | chr. 4 (+):  225035913- 225036013 |  |
| platypus | SNORD14A | GCtcactgtgatgattgcttccataccattcgcagtttccaccagaaaggttttatctttgtgttggctaaaccttccttggatgtctgagtgaGT | Ultra270 (+): 436460- 436555 | The genes are localized in introns of RPS13. |
| platypus | SNORD14B | ATGTGGAGACACtcaccgtgatgatggttccaaaccattcgcagtttccaccagaaaagtctttttccttaatgttggctaaaccttccttggatgtctgagtgaGCGTATCCATGT | Ultra270 (+): 437307- 437423 |
| platypus | SNORD14C | GAtcgctgtgatgagtatttccaaaaccattcgtagtttccaccagaagttgactaatgatggcaaagtaccttccttggatgtctgagcgaTC | Contig7489 (-): 20490- 20583 | The genes are localized in introns of HSPA8. |
| platypus | SNORD14D | AGTAtcgctgtgatgaatgcttccaaaaccattcgtagtttccaccagaaggtgactgatgatggctaagttccttccttggatgtctgagcgaGACT | Contig7489 (-): 19294- 19391 |
| platypus | SNORD14E | AGGCTCTTGtcgctgtgatgaaaatgaccccaaaccattcgtagtttccaccagaagttgagacatgtgttggcccaagttccttccttggatgtctgagcgaCGAGTCT | Contig7489 (-): 18800- 18909 |
| platypus | SNORD14F | AGTGGTGGGAAAAGTCCAGGGGCCTgctgtgatgcaatgttccaaaccattcgtagtttccacctgaagtcccgagactcatgttggctcaagttccttccttggatgtctgagcgGCTGGACTTAACCACTACT | Contig7489 (-): 18135- 18269 |
| platypus | SNORD14G | TGgttgctatgatagattccaaaaccatttgtagtttccaccagaagctgacacatgttggcttaaaagttccttccttggatgtctgagcgaCTA | Contig7489 (-): 17502- 17597 |
| platypus | SNORD14Ψ | TGGTGACTACCTCCAAACCAT_CCC_GTTTCCACCAGAGAAGTTTTCCCTGCTGTTGACTTAAACCTTCCTTGGATCCCGGA | Ultra270 (+): 435918- 435997 | The pseudogene is localized in an intron of RPS13. |
| chicken | SNORD14A | TGGACTTGCAATGATGAATCAGATTCCAAAGCCATTCGTAGTTTCCACCAGAAGTCTACTAATGATGGCCAAAACCTTCCTTGGATGTCTGAGCGAGCTG | chr.24 (+): 3114275- 3114374 |  |
| chicken | SNORD14B | TGGTTGCAATGATGAAAGTGATTCCTGTGCCATTCGTAGTTTCCACCAGAAGTCGAAAGACGAGTGTTGGTCCAAGTACCTTCCTTGGATGTCTGAGCGACCA | chr.24 (+):  3114773- 3114875 |  |
| chicken | SNORD14C | GCTACTGGTTGCAGTGATGAAAGTGATTCCTGTGCCATTCGTAGTTTCCACCAGAAGTCGAAAGACGAGTGTTGGTCCAAGTACCTTCCTTGGATGTCTGAGCGACCACAGT | chr.24 (+): 3115801- 3115912 |  |
| lizard | SNORD14A | AGGTTGCTCACCATGATGATTGTTGGTAACCATTTGCAGTTTCCACCAGAAAGGTTTTCCTTTGTGTTGGCTAATCTTTCCTTGGATGCCTGAGTGAGCACCT | scaff.49 (-):2261112 - 2261214 |  |
| lizard | SNORD14B | TGCTATTCGCCCTGATGATTGAGTCCAAAACCATTCGTAGTTTCCACCAGAAGCCAACTAATGATGGTCCAAACCTTCCTTGGATGTCTGAGCGAATAAGTA | scaff.521 (+):452248- 452349 |  |
| lizard | SNORD14C | CTAAATGACTGGACGCAATGATGAAAGTGATCCAAAACCATTCGTAGTTTCCACCAGAGGTTGAAAGACCTATGTTGGCTTAAAGTACCTTCCTTGGATGTCTGAGCGACCAGTTAGTTAG | scaff. 521 (+):452710- 452830 |  |
| lizard | SNORD14D | AGTCGCAATGATGAAAGTGATCCAAAACCATTCGTAGTTTCCACCAGAGGCTGCAAATCCAGTGATGGCCAAAGCACCTTCCTTGGATGTCTGAGCGACT | scaff. 521 (+):454053- 454152 |  |
| frog | SNORD14A | GTTCCCCGGCTCACAGTGATGATTGGTTTCCAGTCATTCGCAGTTTCTACCAGAAAGTTCTCCTGCTGTTGGACTGTAACCTTCCTTGGATGTCTGAGTGAGCGGAAC | scaff.707 (+): 531789- 531883 | The genes are localized in introns of RPS13. RPS13 exons were identified by the alignment with human RPS13 mRNA. The SNORD14A gene is in the opposite strand, which is likely an assembly artifact. |
| frog | SNORD14B | GCTCACAGTGATGATTGGTTTCCAGTCATTCGCAGTTTCTACCAGAAAGTTCTCCTGCTGTTGGACTGTAACCTTCCTTGGATGTCTGAGTGAGC | scaff.707 (-): 530196- 530290 |
| frog | SNORD14C | GCTCACAGTGATGATTGGTTTCCAGTCATTCGCAGTTTCTACCAGAAAGTTCTCCTGCTGTTGGACTGTAACCTTCCTTGGATGTCTGAGTGAGC | scaff.707 (-): 530778- 530872 |
| frog | SNORD14D | TGATCGCTGTGATGAATTTGATTCCAAAGCCATTCGTAGTTTCCACCAGATGTCGCAAGACTTATGATGGTTTATTACCTTCCTTGGATGTCTGAGCGATCA | scaff.298 (-): 588277- 588378 | The genes are localized in introns of HSPA8. |
| frog | SNORD14E | ATTTGCTAAGATGAATATGATTCCAAAGCCATTCGTAGTTTCCACCAGATGTCGCAAGACTCATGATGGCTCTTTACCTTCCTTGGATGTCTGAGCGAAT | scaff.298 (-): 587492- 587591 |
| frog | SNORD14F | AGTCGCTATGATGAACTTGATTCCAAAGCCATTCGTAGTTTCCACCAGATGCCAAAAGGCTGATGATGGTCTAGCACCTTCCTTGGATGTCTGAGCGACT | scaff.298 (-): 586457- 586556 |
| zebrafish | SNORD14A | TTCGCTATGATGAAGTTTATTCCTTGCCATTCGTAGTTTCCACCAGAGGTTGAAAAACCAAAGATGGCCCAAGTACCTTCCTTGGATGTCTGAGCGAA | chr.10(+): 25062508- 25062605 | The genes are localized in introns of HSPA8. |
| zebrafish | SNORD14B | TTCGCTATGATGAAGTTTATTCCTTGCCATTCGTAGTTTCCACCAGAGGTTGAAAAACCAAAGATGGTCCAAGCACCTTCCTTGGATGTCTGAGCGAA | chr.10(+): 25062936- 25063033 |
| zebrafish | SNORD14C | AATTTCGCTATGATGAAGTTTATTCCTTGCCATTCGTAGTTTCCACCAGAGGTTGTATAACCAAAGATGGCCCAAGTACCTTCCTTGGATGTCTGAGCGACGTT | chr.10(+): 25063323- 25063426 |
| zebrafish | SNORD14D | TATCGCTGTGATGAAGTTTATTCCTTGCCATTCGTAGTTTCCACCAGAGGTTGAAAAACCAAAGATGGCCCAAGTACCTTCCTTGGATGTCTGAGCGTG | chr.10(+): 25064082- 25064180 |
| tetraodon | SNORD14A | GTGCAcgctgtgatgatgtcatttcctgccattcgtagtttccaccagaggtcacatgaccaaagacggttcaaagcttccttggatgtctgagcgaaGCGT | Un_random (-): 66723613- 66723714 |  |
| tetraodon | SNORD14B | TGTTTCCTGtcgctgtgatgatgtcatttcctgccattcgtagtttccaccagaggtcacatgaccaaagacggttcaaagcttccttggatgtctgagcgaCAGAAGCA | Un_random (-): 66723386- 66723495 |  |
| tetraodon | SNORD14C | GtcgctgtgatgatgtcatttcctgccattcgtagtttccaccagaggtcacatgaccaaagacggttcaaagcttccttggatgtctgagcgaC | Un_random (-): 66723141- 66723235 |  |
| tetraodon | SNORD14D | TGCAcgctgtgatgatgtcatttcctgccattcgtagtttccaccagaggtcacatgaccaaagacggttcaaagcttccttggatgtctgagcgaaGCG | Un_random (+): 87249678- 87249777 |  |
| tetraodon | SNORD14E | TTTCCTGtcgctgtgatgatgtcatttcctgccattcgtagtttccaccagaggtcacatgaccaaagacggttcaaagcttccttggatgtctgagcgaCAGAAGCAAA | Un_random (+): 87249899- 87250008 |  |
| tetraodon | SNORD14F | CTGtcgctgtgatgatgtcatttcctgccattcgtagtttccaccagaggtcacatgaccaaagacggttcaaagcttccttggatgtctgagcgaa | Un_random (+): 87250158- 87250254 |  |
| tetraodon | SNORD14G | TGTTTCCTGtcgctgtgatgatgtcatttcctgccattcgtagtttccaccagaggtcacatgaccaaagacggttcaaagcttccttggatgtctgagcgaCAGAAGCA | Un_random (+):  87250377 - 87250486 |  |
| tetraodon | SNORD14H | tcgctgtgatgatgtcatttcctgccattcgtagtttccaccagaggtcacatgaccaaagacggttcaaagcttccttggatgtctgagcga | Un_random (+): 87250639- 87250731 |  |
| tetraodon | SNORD14I | GTGCAcgctgtgatgatgtcatttcctgccattcgtagtttccaccagaggtcacatgaccaaagacggttcaaagcttccttggatgtctgagcgaaGCGT | Un_random (+): 87263271- 87263372 |  |
| tetraodon | SNORD14J | TTCCTGtcgctgtgatgatgtcatttcctgccattcgtagtttccaccagaggtcacatgaccaaagatggttcaaagcttccttggatgtctgagcgaCGGAA | Un_random (+):87263495- 87263598 |  |
| tetraodon | SNORD14K | CTGtcgctgtgatgatgtcatttcctgccattcgtagtttccaccagaggtcacatgaccaaagacggttcaaagcttccttggatgtctgagcgaa | Un_random (+): 87263750- 87263846 |  |
| tetraodon | SNORD14L | TGTTTCCTGtcgctgtgatgatgtcatttcctgccattcgtagtttccaccagaggtcacatgaccaaagatggttcaaagcttccttggatgtctgagcgaCGGAAGCA | Un_random (+):87263969- 87264078 |  |
| tetraodon | SNORD14M | GtcgctgtgatgatgtcatttcctgccattcgtagtttccaccagaggtcacatgaccaaagacggttcaaagcttccttggatgtctgagcgaC | Un_random (+): 87264230- 87264324 |  |
| mouse | SNORD15A | AGCCCTTCGATGAAGAGGTGATGACGAGTCTGAGTAGGAAGTGTTGTCTTTGTCCAAGATGCCTCACTATGCTGCGTTCTGTGGCACAGCTGAAAGCACTGTGGTCAAAAGAAACTTCCTAAAGATGACCAAGAGGCATTTGTCTGAGAAGGGTT | chr.7(-): 106631292- 106631446 |  |
| mouse | SNORD15B | CTGTGCCCTTCAGTGATGACACGATGACGAGTCAGAATGGCCACGTCTTGCTCTTGGTCCCTGTCAGTGCCATGTTCTGTGGTGCTGTACATGGTTCCCTTGGCAAAAGTGTCCTGCGCACTGATTGATTTAGAGGCATTTGTCTGAGAAGGGAACAG | chr.7(-): 106628067- 106628224 |  |
| rat | SNORD15A | TGTGGTAAAGCCCTTCGATGAAGAGGTGATGACGAGTCTGACTAGGAGGTATTTGTCTTTGCCCAGGATGCCTCACTGCGCTGCGTTCTGTGGCACAGTTGTGAGCACTGTGGTCAAAGAAACTTCCTAAAGATGACCAAGAGGCATTTGTCTGAGAAGGGTTGCTGCA | chr.1(-): 156815886- 156816054 |  |
| rat | SNORD15B | AGCCTTCTGTGTCCTTCGGTGATGACACGATGACGAGTCAGAAAGGCCACGTCTTGCTCTTGGGCCCTGTCAGTGCCATGTTCTGTGGTGCTGTAAGTGCGTCCCTTGGCAAAAGTGTCCTGCATACTGATTGATTTAGAGGCATTTGTCTGAGAAGGGAACAGAGCT | chr.1(-): 156812178- 156812345 |  |
| dog | SNORD15A | TTTAAAACCCTTCAATGAAGAGAAGATGACGAGTCTGACTGGAGGTGTTATCTTTGCCAGGCGGCCCCTACTCCTTGCTGCGTTCTGTGGCAGTTTTAAGGGTCCTTGGGTTAAAGTAACTTCCAAAGGACGATCTAGAGGCATTTGTCTGAGAAGGGTTGTTAAA | chr.21(-): 26190814- 26190979 |  |
| dog | SNORD15B | GTACCCTTCGGTGATGACACGATGACGAGTCAGAAAGGTCACATCCTGCTCTTGGTCCTTGTCAGTGCCACGTTCTGTGGTGCTGTGCATGGGTTCCTTTTGCAAAAGTGTCCTGTTTATTGATTGATTAGAGGCATTTGTCTGAGAAGGGAC | chr.21(-): 26186724- 26186876 |  |
| opossum | SNORD15A | AACCCTTCAATGAAGATGCGATGACGAGTCTGACAGGAGGTGTACTCTGTTCAGGTGTTCTACTCTGGTTCTGCGTTCTGCAGGCCAGAATTCAGACTCCTGACTTAAAGTCATTTCCTTAAGAAGATCTAGAGGCATTTGTCTGAGAAGGGTT | chr.4(-): 339912284- 339912437 |  |
| opossum | SNORD15B | CCCCTTCAGTGATGACAAGATGACGAGTCAGAAAGGGCACTACCTGCTCTGTACCCCCTGGCTTTGGCACGTTCTGTGCCACTGTGTGTGGGGGTCTTTGCAGAGGTGTCTGGACATTGGTTGATCTAGAGGCATTTGTCTGA | chr.4(-): 339909420- 339909562 |  |
| chicken | SNORD15A | ATCCCTTCAGTGATGATAAGATGACGAGTCAGAAGGGATGGCTCCTGCTGAGTGGTGCCCCTGCAGTGTCACGTTCTGTGCTGCTGCATGTGGTTCTTCAGCAGGAGCATCCCGTCGCTGAATGATTTAGAGGCATTTGTCTGAGAAGGGAT | chr.1(-): 200051593- 200051744 |  |
| chicken | SNORD15B | TCCCTTCAGTGATGATAAGATGACGAGTCAGAAGGGATGTCCTTGTGCCAGCACAGCCTGGTCAGTGCCATGTTCTGTGGTGCTGTACTAAGGCTGCCTTGGGCAGGATGTCCTCCTCATTGGTTGATTCAGAGGCATTTGTCTGAGAAGGGA | chr.1(-): 200050203- 200050355 |  |
| chicken | SNORD15C | GCCCTTCCATGAAGATGTGATGACGAGTCTGAAAGGGAATATCCTTGCCCAAGTGCCCTGCTTCTTGCTGTGTTCTACAGCATGGATCTGGGGGCCTTGGGTCAGAATTTGAGTGACCTAGAGGCATTTGTCTGAGAAGGGC | chr.1(-): 200049527- 200049668 |  |
| lizard | SNORD15A | AGGTCCCCTTCAGTGATGATTCGATGACGAGTCAGAAAAGGATGCTCTTCTTCCCAGAGCAAACAGTTCAGTATCACGTTCTGTGGTGCTGAGCCTGGCTGCTCTGGGAGAGGTGTCCTGTTCATTGGATGATCTAGAGGCATTTGTCTGAGAAGGGTGAACC | scaff.626 (+): 300909- 301071 |  |
| lizard | SNORD15B | ATCCCTTCCGTGATGATACGATGACGAGTCAGAAATGGCCACCCTCCTGCCCAGAGTCAACAACATTGTGCCACGTTCTGTGGCTCTCTGTTTAGTTGCTCTGGTCAATGGTGTCCTCTTCCTTGGATGATCCTGAGGCATTTGTCTGAGAAGGGAT | scaff.626 (+): 303816- 303972 |  |
| frog | SNORD15A | AGGATTCTTCAGTGATGATTTGATGACGAGTCGGACATTCTCCTCTCGTGCCCCAGTCGGCGGAAGGAGCTGCGTTCTGCCGCTGCTTTTGCTGTTGGGTTCTCATGAGAAAGTTGTGTGAAGAGTCTTGAGGCATTTGTCTGAGAAGAAAACCT | scaff.277 (-): 1315376- 1315530 |  |
| frog | SNORD15B | TCAGCCCTTCAGTGATGAGACGATGACGAGTCAGACAGGACAAGCAATGTAGTGTTTCCTGCACGGGGGCACGTTCTGTGTCGCTGGGCTTGGCTGCACTGCATGAGATTTGTCCTTCTTACTGATCGATATTGAGGCATTTGTCTGAGAAGGGTTGG | scaff.277 (-): 1313454- 1313611 |  |
| frog | SNORD15C | GTGCAGCCCTTCAGTGATGAGACGATGACGAGTCAGACAGGACAAGCAATGTAGTGTTTCCTGCACAGAGGCACGTTCTGTGTCGCTGTACTTGGCTGCACTGCATGAGATTTGTCCTTCTTACTGATCGATATTGAGGCATTTGTCTGAGAAGGGTTGGCA | scaff.277 (-): 1312049- 1312210 |  |
| frog | SNORD15D | CCTTCTGTGATGATACGATGACGAGTCTGATTGGGTAAATCCTTTTCCAGTGGTCCCTGTCTGCATCACGTTCTGTGCTGTAGGCTGAAGCCCACTGAGTCAAGGTTGCCCTTTGCTTGATGTCCATGAGGCATTTGTCTGAGAAGG | scaff.277 (-): 1310755- 1310901 |  |
| zebrafish | SNORD15A | ATTTCCCTTCGGTGATGATACGATGACGAGTCGGAGTAGGGCCGGTTCTGTCTCTGAGGATTCTGGGTCAGGTTCACGTTCTGTGCGTCTGGCTGCAGCTCCTCAAAGCAGTGAGTCCTTTTGCTTGAAGACATAGAGGCATTTGTCTGAGAAGGAGAAAT | chr.18(-): 1260524- 1260684 |  |
| zebrafish | SNORD15B | ATTTCCCTTCGGTGATGATACGATGACGAGTCGGAGTAGGGCCAGTTCTGACTTTGAGGATTCTGGGTCAGGTTCACGTTCTGTGCGTCTGGCTGCAGCTCCTCAAAGCAGTGAGTCCTTTTGCTTGAAGACATAGAGGCATTTGTCTGAGAAGGAGAAAT | chr.18(-): 1256789- 1256949 |  |
| zebrafish | SNORD15C | CTGAATTTCCCTTCGGTGATGATACGATGACGAGTCGGAACAGGGCCAGTTCTGACTTTGAGGATTCTGGGTCAGGTTCACGTTCTGTGCGTCTGGCTGCAGCTCCTCAAAGCAGTAAGTCCTTTTGCTTGAAGACAGAGGCATTTGTCTGAGAAGGAGAAACAG | chr.18(-): 1253839- 1254003 |  |
| zebrafish | SNORD15D | CCCTTCAGTGACGATACGATGACGAGTCGGATCGAGGACCAACTCTGAGGATTCTGGGTCAGACTCACGTTCTGTGCGTCTGATTTCAAGTCCTCGGAGATCATGCGTCCTCTAGATTGAAGATCTTGAGGCATTTGTCTGAGAAGGG | chr.18(-): 1251336- 1251483 |  |
| fugu | SNORD15A | TCTTTTCCCTTCGGTGAAGATAAGATGACGAGTCGGAATAGGACAGATCCTGCTTCTGTCGGTCACTGGTTCAGGTCCACGTTCTGTGGGTTTCTACCAGTTCTTCAGAGTGGGTCGTCCTCTTCATTTGAAGACACTGAGGCATTTGTCTGAGAAGGGTTAAGA | Un (+): 241499231- 241499395 |  |
| fugu | SNORD15B | CCCTTCAGTGATGATACGATGACGAGTCGGAACAAAGCCGTCTTGTTCTGGGGGTTGCTGGTTCAGCTCTACGTTCTGTGGTTCTGTTCCAGTTCCTCAGTTCAGGGTCTTTTTCTCTTGAAGACATAGAGGCATTTGTCTGAGAAGGG | Un (+): 241500319- 241500467 |  |
| mouse | SNORD16A | ACTTGCAGTGATGTCGTAATTTGCGTCTTACTCTGTTCACAGCGACAGTTGCCTGCTGTCAGTATGCTGGTTCAGAAGGTTGACGAACATTGTTACTGAGCAAGT | chr.9(+): 64023227- 64023331 |  |
| mouse | SNORD16B | GGGCTTTTGGGCTTGCAATGATGTTGTAATTTGCGTCTTACTCTGTTTTCAGTGACAGTTGCCTGCTGTCAGTAAGCTGAATCAGAAGGTTGACGAAAATTATTGCTGAGCAGCTCAAAACTTT | chr.9(+): 64024455- 64024578 |  |
| cow | SNORD16 | CCTTGCCATGATGTCGTAATTTGCGTCCGACTCTGTTCCCAGCGACAGTTGCCTGCTGTCAGTAGGCTGGTGCAGAAGGGTGACGAAAATTTTACTGAGCAAGG | chr.10(-): 13229053- 13229156 |  |
| opossum | SNORD16A | CATTGCTATGATGTCGTAATTTGCGTCTTACCTAGCGTTCAGTGACAGTTGCCTGCTGTCAGTAAACTGGCGCTTTCTGGGTGAAGACTACTTGTAACTGAGCAAT | chr.1 (-): 152729978- 152730083 |  |
| opossum | SNORD16B | CTTGCTGTGATGCTGTAATTTGCGCCTTACTCTGTTCTCAGTGACAGTTGCCTGCTGTCAATAAGCTGGTACAGAAGGTTGCTGAAAATGTTTTTCTGAGCAAG | chr.1 (-): 152728738- 152728841 |  |
| platypus | SNORD16A | CATTGCTATGATGCTGTAATTTGCGTCTTATCCAAGCGTTCGGCGGCAGTTGCCTGCTGCCAGAAAGCTGGCGCTCTTTGGATGACGATTACTCGTGACTGAGCAAT | Contig 21556(-): 2479- 2585 |  |
| platypus | SNORD16B | AGCTTGCCATGATGTCGTAATTTGCGTCTTACTCTGTTCTCAGTGACAGTTGCCTGCTGTCAAGAAGCTGGTACAGAAGGTTGACGAAAAGAAATCTCACTGAGCTAAGCT | Contig 21556(-): 1579- 1689 |  |
| chicken | SNORD16A | TATTGCTGTGATGATGTAATTTGCGTCTTATCCAGTGTTCAGCGACAGTTGCCTGCTGTTACCATGTTGGCACTGTTGAGCTGCAGACACATTTAATTCTGAGCAATA | chr.10 (-): 20672638 - 20672745 |  |
| chicken | SNORD16B | CTTGCCATGATGTCGTAATTTGCGTCTTACTCTGTGCTCAGTGACAGTTGCCTGCTGTCAGTGAACTGGTACAGATGGTTGACGAACAGTTAATGCTGAGCAAG | chr.10 (-): 20671249- 20671352 |  |
| lizard | SNORD16A | GCACATATTGCTATGATGTTGTAATTTGCGTCGTATCCGGTGTTCAGGGACAGTTGCCTGCTGTCCACATGCTGTCACCGATGAGCTGAAGATTCTCTAAAACTGAGCAATATTTGTGC | scaff.657 (-): 36480- 36598 |  |
| lizard | SNORD16B | CTTGCCATGATGTCGTAATTTGCGTCTGACTCTGCACTCAGCGACAGTTGCCTGCTGTCACAAGGCTGGTGCAGGTGGTTGGTGACCAGCTAATTCTGAGCAAG | scaff.657 (-): 34626- 34729 |  |
| frog | SNORD16A | ATGCAATGATGGTGTAATTTGCGTCTTACCTGGTATTCAGCGACAGTTGCCTGCTGTCATAAAGCTGGTGCCAAGGGGTGACGATAATACAAGTCTGAGCAT | scaff.363 (-): 1005088- 1005189 |  |
| frog | SNORD16B | TTTTTACATGCTATGATGTCGTAATTTGCGTCTTACTCCACATCATGCGACAGTTGCCTGCTGTCATTAAGCTGGTGTGGGTGACTGACGAATATCGCGTTCTGAGCAAAAA | scaff.363 (-): 1004363- 1004474 |  |
| zebrafish | SNORD16A | TCTTGCTATGATGGTGTAATTTGCGTCCTACTCTGTGTTCAGTGACAGTTGCCTGCTGTCTCATGCTGGCACAGAAGGTTGATGACAACAAAGTCTGAGCAAGA | chr.18(+): 14720688- 14720791 |  |
| zebrafish | SNORD16B | ACTTGCTGTGATGGTGTAATTTGCGTCCTACTCTACGCACAGCGACAGTTGCCTGCTGTCTTATGCTGGTGTGGAAGGTTGACGATCATTGAGTCTGAGCAGGT | chr.18(+): 14721346- 14721449 |  |
| tetraodon | SNORD16A | ACTTGCACTGATGGTGTAATTTGCGTCTGACTCTGTGACCGGTGACAGTTGCCTGCTGTCAATATGCTGGCACATACGACTGACGAGCAACAGTGTCTGAGCAGGT | chr.13 (-): 7619128- 7619233 |  |
| tetraodon | SNORD16B | GCCTGCCATGATGGTGTAATTTGCGTCTGATCCTGTGAACGCTGACAGTTGCCTGCTGTCTCTATGCTGTCATAGGTAGTTGATGTATGCTAAGGTCTGAGCAAGC | chr.13(-): 7618750- 7618855 |  |
| mouse | SNORD17 | GTGAAATGATGATTCTGTTTATCCATTCGCTGAGTACGCTGCTCTGACCTTCTTCCCAGTCTCGGTTCCTGTTCTGGGAGCTTGGGGCTGAGTAGCCACCAGCCCTGCTCTCTGCAGTGTTCTATTGTGGATTGCTTGTGTGCTGGCAGGCTACTACTGGTAAGAATGGCTAGTGTCAGCAGGGATGGCTCCTCTCTGGGTTCCATCTCACCAAGATGAGTGGTGCAAATCTGATCAC | chr.2(-): 144091715- 144091952 |  |
| opossum | SNORD17A | TTGCAGCATATGTGAAATGATGATGAAGTTTATCCATTCTCTGAGTGTGCTGCTCTGCCCTTCCTACAGCTCTGAGTTCCTGTTCTAGGAACTCTTGAGTTATGTAACCTCCAGATACCCTCCAGACTTGCAGTGTTCTACTGTGGATGGCAGGTGTGTGTGGTGGTCTTACTAGTAGGAAGAGATGGAGTTAGCAGAGGTGAAACCCCATTATTTGGGGTCCCATCTTACCAAGACGAATGACTGGTGTCTGATCACATGCTGCAG | chr.1 (-): 604587489- 604587755 |  |
| opossum | SNORD17B | TTGCAGCATATGTGAAATGATGATGAAGTTTATCCATTCTCTGAGTGTGCTGCTCTGCCCTTCCTACAGCTCTGAGTTCCTGTTCTAGGAACTCTTGAGTTATGTAACCTCCAGATACCCTCCAGACTTGCAGTGTTCTACTGTGGATGGCAGGTGTGTGTGGTGGTCTTACTAGTAGGAAGAGATGGAGTTAGCAGAGGTGAAACCCCATTATTTGGGGTCCCATCTTACCAAGACGAATGACTGGTGTCTGATCACATGCTGCAG | chr.1(+): 604804696- 604804962 |  |
| platypus | SNORD17 | CCCTGTACGTGACATGATGATGAAGTTTATCCATTCTCTGAGCTTGCAGACTCTGCCCTCCTTCAGCATCGAGGTTCCTGTTCTAGGGCCTCGGTGTTTCGCGGCCTTCTGTGCACTCCAGCCTTCCAGTGTTCTACTGGGGGTGCGACCGTGTCCGGTTGACTGCTTGAGGGTGTGGTGGAGTTAGCAGGGTGGGAACCCCTTCTGGGGGGATCCCATCTCGGCAAAGACGAATGATGGGAGTCTGATCACGTACTGTGGG | Ultra519 (-): 9352013- 9352274 |  |
| chicken | SNORD17A | TCAGGTGTGCCGTGTGTGAGGTGATGATGAAGTTTATCCATTCTCTGAGTGCAGTGGCACTGCCCTCCTGCAGCGCCCCGATTCCTGTTCTAGGGTCTGGGTGCCGTGCAGCCTCCAGAGACCCTGCAGTCTCCCAGTGTGCTGCTGGGGGCTGCTGTGGTGTGTGGCTGGCTGCCTGCAGGAGCCAAGCAGTGCCGGCCGGGTCAGGGCCCCTCTGTGGGGGGCTCTGTCACCACTGAGACGAGGGATGACATCTGA | chr.4 (+): 2100251- 2100508 |  |
| chicken | SNORD17B | TCAGGTGTGCCGTGTGTGAGGTGATGATGAAGTTTATCCATTCTCTGAGTGCAGTGGCACTGCCCTCCTGCAGCGCCCCGATTCCTGTTCTAGGGTCTGGGTGCCGTGCAGCCTCCAGAGACCCTGCAGTCTCCCATTGTGCTGCTGGGGGCTGCTGTGGTGTGTGGCTGGCTGCCTGCAGGAGCCAAGCAGTGCCGGCCGGGTCAGGGCCCCTCTGTGGGGGGCTCTGTCCCCACTGAGACGAGGGATGACATCTGA | chr.4 (+): 2103039- 2103296 |  |
| lizard | SNORD17 | ACATGTGAAATGATGATGAAGTTTATCCATTCTCTGAGTGTCGCTGCTCTGCCCTTCCTGCTACGTTCAGGTCTCCGTTCTGGGGTCTTGGATGAAAGGCCTCCAGGCATCTTTCAGCCTTCTGGAGTTCTTctgggggctgggagcatggctggttggctgctggcaaggcctgggcaggggctgcggggcCAGAGCACCTTTCTGAGGGGCTCTGACACCAACCAAGAAGAATGAAACTGTCTGATCACATGT | scaff.131 (-): 2621330- 2621584 |  |
| frog | SNORD17 | GTTGTGAAATGATGACACAGTTTATCCATTCTCTGAGCCTGCAGCCAGCCATTGCAGCTCACAGACTCCTAATGTTGTCTGTGAGTCTCTTCCCCTCTTAGTGTTGGTGATGCTGCGTCCTGCAGTGTGACCTTCACGCAGAGGTGTGTTGGGAGCAAGGGATGTGGCCAAAGGGCAAGACGAGAGAAAACTTACTGATCACTAC | scaff.456 (-): 555571- 555775 |  |
| zebrafish | SNORD17 | TGTATGTGGCGTGATGAACCTGTTTATCCATTCTCTGAGTGTCTCCGTCCCTCTCTCCTGCTGGCCTCCGGCTCTCCGCTGCAGTGTTTTACTGTAGCGTAGGCTGCTGGAGGCTCATGCGGGGGTTTAGGGTTCGGTTTCACAAAAGAAGAACTGACATTTCTGATCACATACA | chr.21(-): 34121018- 34121192 |  |
| tetraodon | SNORD17 | TCTTCAGCATGTGAAATGATGAATCTGTTTATCCATTCTCAGAGCAGCTGTCTCTTAGGTCTCTGCCTCTTCTCCCGTTCTGGGGGAAGGGTCTCTTCTTTTGTGGCGTGGTTGTCGCAGTGTTTTGCTGGACGATGGCCCAAAACTTAAGAGAGATTAGGCCTGAGCACTGCCAAGA | Un_random (+): 82905386- 82905563 |  |
| medaka | SNORD17 | GTGTGACATGATGAAGCAGTTTATCCATTCTCTGAGCACTGCAGTCTTGGCGTTTCTTTGCCCGTCTCCATCTTTGGGGCCTGGTCTCTGCTGGTCACCAACTGTGACCTGGCGTTCTGCCAGGTTATAGGAGGTGACCGTGTAGAGGTGGAAGCGACCAGACTGTTGTCAAGATGATGCTCTGCTCTGATCACAC | scaffold 956 (+): 9550- 9743 |  |
| mouse | SNORD18A | GCAGTAAATGATGAGATTCCATTGGTCCGTGTTTCTGAACTACATGATTTTCCTTGGCTATTCTGATACTGC | chr.9(+): 64022853- 64022924 |  |
| mouse | SNORD18B | TTGACTGTCAAAATGATGAAATTCCACTTAATGGTCCGTGTTTCTGAAACGCATGATATTAGTGGAAATTCTGATTTGGCGCAA | chr.9(+): 64023671- 64023754 |  |
| mouse | SNORD18C | GGCTCACTTAGCTTGGTTATGATGAAATTCCACTTCATGGTCCGTGTTTCTGAAATAACATGATATTGTGGAAGTTCTGAGTCCTACGTGAGTGAGCT | chr.9(+): 64024917- 64025014 |  |
| opossum | SNORD18A | AGTGCTACACAGAAATGATGAGATTTCCACTTCATTGGTCCGTGTTTCTGAACCACATGATTTTTCTGGAAGTTCTGATAGTGTGCACT | chr.1 (-): 152729482- 152729570 |  |
| opossum | SNORD18B | TAGCCTTGTAATGATGATATTCCACTTCATGGTCCGTGTTTCTGAAACAATGTGATTTTGTGGAAGTTCTGAAACTTGGTTG | chr.1 (-):  152727113 -152727194 |  |
| opossum | SNORD18C | AAATTAGCAGGAATGATGAGTTTCCACTTCATTGGTCCGTGTTTCTGAAGCCCATGATTTTAGTGGAAGTTCTGATTCTCGTCTTT | chr.1 (-):  152725822- 152725907 |  |
| platypus | SNORD18A | TTATGCCTCACGGTAATGATGAAATTCCACCTCATTGGTCCGTGTTTCTGAGCCACATGATTTGTTCTGGAAGTTCTGACACTGGGCATGA | contig21556 (-): 2043- 2133 |  |
| platypus | SNORD18B | CTGCTAAAGTGATGAGATTCCACTTCATTGGTCCGTGTTTCTGAAACTCATGATATGGTGGAAGTTCTGATTTGGCAG | contig21556 (-): 1119- 1196 |  |
| platypus | SNORD18C | GCCCGGGAATGATGACCCTCCACTTCATGGTCCGTGTTTCTGAAACTCGTGATTTGGTGGAAGTTCTGACTTGGGC | contig 109427 (-): 2873- 2948 |  |
| platypus | SNORD18D | CCTACAGATGATGAGATTCCATTTCATTGGTCCGTGTTTCTGAAATACGTGATTTGATGGAACTTCTGACTGGGGG | contig 109427(-): 2433- 2508 |  |
| chicken | SNORD18A | GGATTTGTTTTGCTGAAATGATGAGATTCCACTTCAATGGTCCGTGTTTCTGAACCATATGATAACAGTGGAAGTTCTGATGCTTGGTCC | chr.10 (-): 20671770- 20671859 |  |
| chicken | SNORD18B | GAGTCTTAAGAATGATGAACTTCTCACTGGTCCGTGTTTCAGATTATCAGTGATGATTGTGAAGTTCTGACTGAAGTGTC | chr.10(-): 20670088 20670167 |  |
| chicken | SNORD18C | TTTTTGGAAGACAGCCCAGAATGATGAGATTCCACTTCATTGGTCCGTGTTTCTGAAACACATGATTTTGTGGAAGTTCTGACTTCTAGCTAAAG | chr.10(-): 20669767- 20669861 |  |
| lizard | SNORD18A | GCCACATAACCGTGATGAGATTCCACTTCATTGGTCCGTGTTTCTGACTTCTGTGATCTCAGTGGAAGTTCTGATGTTGGC | scaff.657 (-): 35188- 35268 |  |
| lizard | SNORD18B | TCTGCAAAGTGATGAATTTTTCACTGGTCCGTGTTTCAGATGAACAGTGGCGATGGTGAAGTTCTGAGTTGGACAGA | scaff.657 (-):33534- 33610 |  |
| lizard | SNORD18C | TTTGAAAATGATGATATTCCACTTCATTGGTCCGTGTTTCTGAAACACATGATTTGGTGGAAGTTCTGACTCCGTAAA | scaff.657 (-):33203- 33280 |  |
| frog | SNORD18A | ATGAATATGATGAGTTCCACTTCATGGTCCGTGTTTCTGAACCCAGTGATAATAATGGAAGTTCTGATTAT | scaff.363 (-): 1004674- 1004744 |  |
| frog | SNORD18B | attgtTGCTTTATGATGAGTTCCACTTCTGGTCCGTGTTTCTGAAGCATCATGATCTTTGTGGAAGTTCTGATCAGCAAAAT | scaff.363 (-): 1003987- 1004068 |  |
| frog | SNORD18C | TTATACATGATGATCTCCACTTCTGGTCCGTGTTTCTGAAACTCATGATCTTTGTGGAAGTTCTGACTTAG | scaff.363 (-): 1002581- 1002650 |  |
| frog | SNORD18D | AGTGTGTTAATGATGAGTTCCACTTCATTGGTCCGTGTTTCTGATACCTTATGATTTCAGTGGAAGTTCTGATATATATT | scaff.363 (-): 1001895- 1001974 |  |
| zebrafish | SNORD18A | GCTCCAGTGATGATATTCCACTTCAGGTCCGTGTTTCTGACAACTGATTATGCTGGAAGTTCTGAGAGC | chr.18(+): 14721064- 14721132 |  |
| zebrafish | SNORD18B | CATTTTCTACATGATGATCTCCACTTCTGGTCCGTGTTTATGAGTCCTATGATTTTAGTGGAAGTTCTGACCACACAATG | chr.18(+): 14723649- 14723728 |  |
| zebrafish | SNORD18C | TTGAACAACAATGATGAGCTTACTTCTGGTCCGTGTTTATGAACACCTTGATTTTATTAGAAGTTCTGAGTCAA | chr.18(+): 14723990- 14724063 |  |
| zebrafish | SNORD18D | GTTGCAATGATGAGATTCTTACTTCAGGTCCGTGTTTCTGAAATAGTGATTGTATTGGAAGTTCTGAGCCAT | chr.18(+): 14724280- 14724351 |  |
| tetraodon | SNORD18A | TTTCAGTGATGATCTTCTACTTCAGGTCCGTGTTTTTGAAACAGTGTGATATTCATCGAAGTTCTGAGTTCTGAGTCAAA | chr.13 (-): 7617990- 7618069 |  |
| tetraodon | SNORD18B | TGTCGCCTATGATGATGTTCCACTTCTGGTCCGTGTTTCTGAGTCCTGATTGTTACGGAAGTTCTGAGCAACA | chr.13 (-): 7617736- 7617808 |  |
| human | SNORD19C | GTGAGAAATGATGAGGGTCAACATTCTTCATACCAAAGTGAAGACATGAGATCCAACTCTGAGCTCAC | chr.3(+): 52697944- 52698011 |  |
| human | SNORD19D | GCAAGAAATGAAGAAACTAAAATTGGTCTTAGTATTGAAGTGAAGACACTGAGATCCAACTCTGATCTTGC | chr.3(+): 52700432 - 52700502 |  |
| mouse | SNORD19A | TTTTGTGCACTATGAGGTATGAGGAAAATATCGGACAGTCTGATTACCACTGAAGACTGATAAGATCCAACTCTGACCTCAACAAAG | chr.14(-): 31829394- 31829480 |  |
| mouse | SNORD19B | TGATTGAAATGTGATGAGTACACAAATGTTGATGAAAGTGAAAAACATGAAGATCCAACTCTGATTTCATTA | chr.14(-): 31828338- 31828409 |  |
| dog | SNORD19A | GAGGTATGAGGAAAACATGTGAGCAGTCTGATTTTCATTGAAGACTGATAAGATCCAACTCTGACCTC | chr.20(-): 40102739- 40102806 |  |
| dog | SNORD19B | TGGCTGAAATATGATGAGCATACAAAATCTTGATTAAAAAATGAGAATTTATAAGATCCAACTCTGATTTCATCCA | chr.20(-): 40101787- 40101862 |  |
| dog | SNORD19C | GCAAGAAATGAAGACTCTAAGATTGGTCTTAGTGTTGAAGTGAAGACACTCAGATCCAACTCTGATCTTGC | chr.20(-): 40101243- 40101313 |  |
| cow | SNORD19A | TGAGGTATGAGGAAAACATCTGCACAGTCTTACTGTCTGTGAAGACTCGTAAGATCCAACTCTGACCTCA | chr.22(-): 49219442- 49219511 |  |
| cow | SNORD19B | GGTTCTTTCAGATGGGTGAAACATGATGAGTGTACAAAATCTTGATTAAAAAATGAAAACTTATAAGATCCAACTCTGATTTCATCCAGAAATAATC | chr.22(-): 49218620- 49218716 |  |
| cow | SNORD19C | GCAAGAAATGAAGATAGCAAGAGTGGTCTTAGTGTTGAAGTGAGGACGCTCAGATCCAACTCTGATCTTGC | chr.22(-): 49218183- 49218253 |  |
| opossum | SNORD19A | TGAGGTATGAGGAAAACATCTAAACAGTCTGAAATACCATTGGAGACTGTAAGATCCAACTCTGACCTCA | chr.6(-): 229004678- 229004747 |  |
| opossum | SNORD19B | TTTGGGTTGAAATATGATGAACACTGAAAACTAGATGGAATTGTGAAGATCACAAGATCCAACTCTGATTTCACCCAGA | chr.6(-): 229003961- 229004039 |  |
| opossum | SNORD19C | GGGTGAGAAATGAAGATAGAAATGGCCTTATTAAAATGATGATAAGAGATCCAACTCTGATCTCACTC | chr.6(-): 229002148- 229002215 |  |
| platypus | SNORD19A | GGGCAAGAGATGATGATGATACACATAGTCTTATTGTGACGGTGAAGACATCAGATCCAACTCTGATTTTGCCC | Contig 1718 (-): 54508- 54581 |  |
| platypus | SNORD19B | AGGAATGAGGAAAATATCTGATCAGTCTGATATACCACTGAAGACTGTAAGATCCAACTCTGACCT | Contig 1718 (-): 54148- 54213 |  |
| platypus | SNORD19C | TGGGTTGAACTGTGATGAAACTGATCGCTCTGGATACAAGAGTGAAGAATACAAGATCCAACTCTGATTTCATCCCA | Contig 1718 (-): 53548- 53624 |  |
| platypus | SNORD19D | GAGTGAGAAGTGATGAGGAAACAAATAGTCTTACTGTATAAGTGAAGACATCAGATCCAACTCTGATCTTGCTC | Contig 1718 (-): 52669- 52742 |  |
| chicken | SNORD19A | AGTGAGCTGTGAAGAAATATATAAAGAAATCTGAGATTCTGTTGAAGTCATAAGATCCAACTCTGAGCTCGCT | chr.12(+): 735868- 735940 |  |
| chicken | SNORD19B | ATGTGAGAAATGAGGATACAGACTTTGTCTGAGTTTTATATGAAGACTTCAGATCCAACTCTGATCTCACAT | chr.12(+): 738198- 738269 |  |
| chicken | SNORD19C | ATAGAGTGAAAAATGAAGATACTTTCATTGCTTCTTACTACAGTGAAGACTTCAGATCCAACTCTGATTTCACTCTAT | chr.12(+): 739298- 739375 |  |
| lizard | SNORD19A | GAAAGTGAGCTATGAGGAAAACACAAAAACATACTGACATTCCTCTGAAGACTGTCAGATCCAACTCTGAGCTCACAGTTC | scaff. 44 (+): 1499476- 1499556 |  |
| lizard | SNORD19B | GGAGTGAGAAATGAGGATACATATTGTTTGTCTTACTGTTATTGTGAAGACTTCAGATCCAACTCTGATCTCACTTT | scaff. 44 (+):  1501313- 1501389 |  |
| lizard | SNORD19Ψ | AAGTGAGAAAATGAGGATACTTGACATGTCTTACTgttacagtgaagacatcagatCCAACTCCGATCTTGCTT | scaff. 44 (+):  1503729- 1503802 |  |
| frog | SNORD19A | AGCGGTAAGAGATGAGGAGAAACTGAAATGAATCTGATGTAACTATGACGACTGTCAGATCCAACTCTGATCGCCGCT | scaff.1084 (-): 52823- 52900 |  |
| frog | SNORD19B | GTCAGGCTGGAATGTGATGATTAAACTGGATTCTGACTTTTCCTGACTGAGCACAAGATCCAACTCTGATTTCTGCCTGAT | scaff.1084 (-): 52117- 52197 |  |
| mouse | SNORD20 | TGACAACTGGATATGATGACTGATTACCTGAGAAATAATTGATGAAATCTCAAGAAAATTCCTCTAGATAGTCAAGTTCTGATCCAGCTATGTCA | chr.1(-): 88248575- 88248669 |  |
| opossum | SNORD20 | TGATACTGGATATGATGACTCATTATCTGAGAATTGCTGATGAAACCTCCAGAAAATTCCTCTAGATAGTCAAGTTCTGATCCAGCTGTCA | chr. 2 (-): 536433013- 536433103 |  |
| platypus | SNORD20 | ATGCTGGATATGATGACTGATTATCTGAGACAAACTGATGAAACCAATAAAAATTCCTCTAGATAGTCAAGTTCTGATCCACAT | Contig 30838(-): 1796-1879 |  |
| chicken | SNORD20 | AGTGGATGTGCTGGATATGATGACTGATTATCTGAAATGCTGATGAAACCACTTCTAAAGTCCTCTAGATAGTCAAGTGCTGATCCAGCAGTGTCTGTT | chr.9(-): 16372619- 16372717 |  |
| lizard | SNORD20 | AGCTGGAAAATGATGATTTCTCGCTGAGCTGATGTGATACGAATCACAAATGACCGCTTCCAAAGATAGTCAAGTCCTGATCCAGCT | scaff. 852 (-): 25132 - 25218 |  |
| frog | SNORD20 | AGGTGCTGGAAGTGATGAGCTGTTATCTGAGAATTTTGTGATGAAAATTAACAATTTCTCTAGATAGTCAAGTACTGATCCAGCATTT | scaff.285 (+): 1175766- 1175853 |  |
| fugu | SNORD20 | AATGGGACCAGATATGATGAAATCTTGTCATCCAGAAACCCTGTGAGGACTCAGAGATAGTCAAGTCCTGATCTGTCCCGATT | Un(-): 102410348- 102410430 |  |
| mouse | SNORD21 | GAGCTGCCAATGATGACACCACACTAACTGAGCAGTTAGTAGTTGGTCCTTTGATTGCATGTGATGTAATCATTGTTTCAAGACGGGACTGAAGGCGGCTC | chr.5(+): 108333569- 108333669 |  |
| opossum | SNORD21 | TAGCTGCTGAATGATGATATCCCACTCACTGAGTTGCTTATGATGCAATTGTTTCAAGACGGGACTGATGGCAGGTA | chr. 2 (-): 11545731- 11545807 |  |
| platypus | SNORD21 | GCTGCCGGATGATGATCTCCCACGACTGAGCATTCTGTAGTTGGTCCTTGGTTTGCACATGATGCAATTTCAGTGTTTCAAGACGGGACTGACGGCAGC | Contig9329 (-): 11700- 11798 |  |
| chicken | SNORD21 | TGCTATGTGATGATACCCCAATGTCTGACCACTCCGTAGTAGGTCCATGGTGTGTAAATGATGGACTGCGTAGTTTCAAGACGGGACTGATGGCA | chr.8(-): 14748804- 14748898 |  |
| lizard | SNORD21 | TTGGGCTACTATGTGATGATATCTCACTGTCTGAACATACTGTAGTTGGTCCTCGGCATGTGTATGATGCATCTCACTGTTTCAAGACGGGACTGATGGTGGCTAA | scaff. 199 (-): 771230- 771335; scaff.4448 (+): 4105- 4210 |  |
| zebrafish | SNORD21A | GTCTTGATGATGACACCACTTGAAACTGAACAGCTGCTATTTCAGTTGTTCCACTGATGTGATTTTCAAGACGGGACTGATTGCAAGAC | chr.6(+): 20521193- 20521281 |  |
| zebrafish | SNORD21B | GCTGCTTGATGATGATACCCTTTCAGACTGAGCAAATTGTAGTTGGTCCTACATTTGCACAGTTGATGCAAGCATATTTCAAGACGGGGCTGAGAGCAGT | chr.6(+): 20528481- 20528580 |  |
| human | SNORD22 | TCCCAATGAAGAAACTTTCACATGTCTTACTCTCTGTCCTAGTCCCAGAGCCTGTAAAGGTGAACCCACTGGGACTGGCTGGGGGAGAAGAGGAAGATTTGTTCCAGAAGGAACTGTCTGAGGGA | chr.11 (-): 62376959- 62377083 |  |
| mouse | SNORD22 | TCCCCATGAAGAAATGTTCACACGTCCTACTTCCTGTCCTAGCTCCAGAGCCTGAAAAGGTGAACCCACTGGGGCTGGCTGGGGGAAAAGAGGAAACTTTGTTCCAGAAGGAACTGTCTGAGGGA | chr.19 (+): 8800356- 8800480 |  |
| opossum | SNORD22A | TGTTCCCTGGTACCCATGAGGAAAGCAAACAAATGTCTTAACCCCAGTCCTGGCCCCAGAGCCAGTAATGGTGAACCCACTGGGGCTGGCTGGGGGAAAAGAGGAAGCCCTGTTCCTGAAGGAACTGTCTGAGGTACACAGGAGTA | chr.5 (-): 300774815- 300774960 |  |
| opossum | SNORD22B | GAGCAGAGGTACCCAATGAAGAAGACATCACCTGTCTTACTCCCTGACCTGCCCCAGAACCTGCAAAGGTTGAACCCACTGGGGCTGGCTGGGAGGCAGGAGGAAACATGTTCCAGAGAACTGTCTGAGGGACCTTCTGATTT | chr.5 (-): 300773489- 300773631 |  |
| platypus | SNORD22A | AGGGTACTAATGAGGAAATCCACACCTGTCTTAGCTCCTGTCCAGTTCCAGAGCCTGCAAAGGCGAACCCACTGGAATTGGCAGGGGAGAGAGGAAGATAAAATGTTCCTGAGTGAACAGTCTGATGTGCCCT | Contig18615 (-):10084- 10216 |  |
| platypus | SNORD22B | TGGGTCCTAATGAAGAAAACCAGCACCTGTCTTACTCCTTGTCCTGGCCCCAGAGCCTGCAAAGGCGAACCCACTGGGGTCGGCTGGGGGAGAAGATGATAATCTGTTCCAGCGGGAACAGTCTGAGGGACCCA | Contig18615 (-): 8359- 8492 |  |
| lizard | SNORD22 | ACGTTTTAGGCCAATCCCAATGATGATCTCTCATACGTCTTACTATCTGTTTTGGGGCTAGAACCTGCAAAGGTTGAACCCACTAGCACCAGCAGATGGGAAAGAGGAAACTTGGCTCCGGAAGGAACAGTCTGATGGGATGTCTCAAAAACGT | scaff.1488 (+): 63788- 63941 |  |
| frog | SNORD22 | TTTATTCAGATATGCCCCAATGATGAAACCTTCACATGTCCGACCAGCTGCCTGGTGCCAGAGCCTGAAAAGGTGAACCCACTGGTACCAACAGCTGGAGAGGAAGGAAGACTTGTTCCAGCTGGAACTGTCTGAGGGCTTATTTATAATAAA | scaff. 782 (+): 33791- 33943 |  |
| zebrafish | SNORD22 | CTTTTATGATGCAACTCGCACACGTCTTACGCCCTGTGTCAATGCCAGAACCTGAAAAGGTGAACCCACTGGTGTGTCCTGGGCAATAAGGGGATGATCCGTTGGGGTTTCCAACAGTCTGAGAAG | chr.7 (+): 15719521- 15719646 |  |
| tetraodon | SNORD22 | CCCGGTTTCCTTGGTGATGACACCCGCACACGTCCGTCGCCTCGCGCCAACGCCAGAACCTGAGAAGGTGAACCCACTGGCGTTGCCTTGGCAGCCGGGGGAAGACTGGTTGGGGTTACCAACAGTCTGAAGGGAAACCCGGG | Un_random + 21179285 21179427 |  |
| mouse | SNORD24 | GTCGTGTGCAGATGATGTGACAGAATATTTGCTATCAGATGAGTGGTGATGACACTAGAACCACCAAGATCGCTGATGCACCGAC | chr.2(+): 26766775- 26766859 |  |
| cow | SNORD24A | GGCTTGTGCAGATGATGTGAAAGAATATTTGCTATCTGAGTGATGGTGAGGACTGTCCTGACCACCAAGATCGCTGATGTACGAGCC | chr.11(+): 108184736- 108184822 |  |
| cow | SNORD24B | TGTGTGCAAGTGATGCAAAAGTATGTTACCTGGAAGATGGTGATGACACTTTAAACCACCAAGATCGCTGATGCACTACA | chr.11(+): 108185471- 108185550 | The first antisense element is disrupted. |
| opossum | SNORD24A | AACCATTGTGCAAATGATGGAAAAGAATATTTGCTATCTTGAATGATGGTGATGAATTTCATAACCACCAAGATCACTGATGCACTTCTGGTT | chr.1(+): 460617858- 460617950 |  |
| opossum | SNORD24B | GAATATGTGCAGATGATGATTACGATATTTGCTATCTGAGTTGCAATGATTACATATGTACCACCGAGATCGCTGATGCACAGATTT | chr.1(+): 460618340- 460618426 |  |
| platypus | SNORD24A | GTGCAGATGATGCAAAAGAACATCCGCTGTCAGAATGGTCGTGGTGACACGTCGCTAACCACCAAGATCGCTGATGCAC | Contig11099 (+): 18597- 18675 |  |
| platypus | SNORD24B | AGTGCAGATGATGATCCATATATTTGCTATCTGAATAGCAGTGATTACAGCCTTACCACCAAGATCGCTGATGCACT | Contig11099 (+): 20221- 20297 |  |
| chicken | SNORD24 | GTGCAGATGATGTGAAAAAATACTTGCTATCTGAATGGTAGTGCTGACATACATAACCACCAAGATCGCTGATGCAC | chr.17(+): 7532606- 7532682 |  |
| lizard | SNORD24A | AGCAAAGGATGCAAGCCGAATATTTGCTATCTGAGTGGCACTGCTGACAAGGCTCATCCACCAAGATCTCTGANNNNNN | scaff.1674 (-): 42496 - 42574 |  |
| lizard | SNORD24B | GGGTGCAGATGATGCCAAGAAATATTTGCTATCTGAGCTGCCTTGATGACTCTCGGCCACCAAGATCTCTGATGCACCC | scaff.1674 (-): 38685- 38763 |  |
| frog | SNORD24A | TAAGCCTGTGCAAGTGATGTGATGAAATATTTGCTATCTGAGCCACAGTGCTGACACTCTACCCACCAAGATCGCTGATGCACAGCCTTA | scaff.878 (-): 20591- 20680 |  |
| frog | SNORD24B | GTGCAGATGATGCCAACTAATATTTGCTATCTGACTAGCAGTGATTACACACTTTCCACCAAGATCGCTGATGCAC | scaff.878 (-): 19207- 19282 |  |
| zebrafish | SNORD24A | GTGCAGATGATGTAATGAATATTTGCTATCTTAACAGCAATGCAGACAATCTCCCACCAAGATCGCTGATGCAC | chr.5(+): 27273530- 27273603 |  |
| zebrafish | SNORD24B | GTGCAGATGATGTGAAGAATATTTGCTATCTGAGTAGTAAATGCGGACAGTTTCCACCAAGATCACTGATGCAC | chr.5(+): 27274591- 27274664 |  |
| tetraodon | SNORD24 | AAGTGCATGTGATGTAAATGAATATTTGCTATCTTAATGCTACTGATGACAATATTCCACCAAGATCGCTGATGCACTT | chr.4(+): 3067788- 3067866 |  |
| mouse | SNORD25 | GGCCACCCCCTATGATGAGGACCTTTTCACAGACCTGTACTGATATATCTGTGAGGATAAGTAACTCTGAGGAGGCTGGCC | chr.19(+): 8798479- 8798559 |  |
| rat | SNORD25 | GGCCATGCCCTATGATGAGGACCTTTTCACAGACCTGTACTGATCTCTGTGAGGATAAGTAACTCTGAGGAGGCTGGCC | chr.1(+): 211390951- 211391029 |  |
| dog | SNORD25 | GCCCTCTCTGTGATGAGAACCTTTCACAGACCTGTACTGATCTCCGTGAGGATAAAAAAAACTCTGAGGAgggc | chr.18(+): 56868593- 56868666 |  |
| opossum | SNORD25 | GGGTCCCTCGGTGATGAGACCTATCACAGACCTGTACTGATACTCTGTGAGGACAAGAGAAACTCCTGAGGGGGAGCCT | chr.5(-): 300776310- 300776388 |  |
| platypus | SNORD25A | TGCAGTAGCTTTTCTCGATGATGATTTTCACAGACCTATACTGATTTCTGTGAGGAAGAAATACTTTCTGAAGAGACTGAGCCTTGCA | Contig18615 (-): 12501- 12588 |  |
| platypus | SNORD25B | TGCAGGAGCCTTTCTCGGTGATGAAAAATTTTCACAGACCTGTACTGACATCTGTGAGGAAAAAATACTTTTCTGAAGAGACTGAGCTTTGCA | Contig18615 (-): 11932- 12024 |  |
| lizard | SNORD25 | TGTCTTCCTGTGATGAAACTTTACACAGACCTGTTCTGAAAAGTTCTGTGAAGTTAACCTTGTTTCTGAGAAGACT | scaff.1488 (+): 58711- 58786 |  |
| frog | SNORD25 | GATGTGCCAATGACGAGACCAATCACAGACCTGTTCTGAGAGCAATGAGGACAGCTTAATATCTCTGAGGCCCCATT | scaff.782 (+): 31474- 31550 |  |
| zebrafish | SNORD25A | GATCTCTCAATGATGATAACTTTCACAGACCTGTACTGAATCACTGTGATTGCACAATATTTATCTGACGTGAGATC | chr.7(+): 15716102- 15716178 | The host gene (UHG) has two copies. |
| zebrafish | SNORD25B | GATCTCTCAATGATGATAACTTTCACAGACCTGTACTGAATCACTGTGATTGCACAATATTTATCTGACGTGAGATC | chr.7(+): 15954797- 15954873 |
| tetraodon | SNORD25 | GATCGGTGTCTCGATGATGATAATTCTCACAGACCTGTACTGAAAACTGTGATTGCAACTATTAAATCTGATGAGACATTTGTT | Un.random (+): 21177457- 21177540 |  |
| fugu | SNORD25 | GGTGTCTCGATGATGATAATTTTCACAGACCTGTACTGAATGTACTGTGATTGCAATTATTAAATCTGATGAGACACT | Un.(+): 294290814- 294290891 |  |
| mouse | SNORD26 | TGTGCTACGGGGATGATATGAAAACTGAACTCTCTCTTTCTGATGGTTTAGTGGAGAAAACAAAAATTCTCTGAGTAGCACA | chr.19(+): 8798737- 8798818 |  |
| rat | SNORD26 | GTGCTACGGGGATGATTTAGAAGCTGAACTCTCTCTTTCTGATGGTTTAGTGGAGAAATCAAAAATACTCTGAGTAGCAC | chr.1(+): 211391231- 211391310 |  |
| dog | SNORD26 | GTGCTACGGGGATGATACTAGGAATTGAACTCTCTCTTTCTGATGGGTTGGTGGAGAAAATGTAAAGATTCTGAGTAGCAC | chr.18(+): 56869059- 56869139 |  |
| opossum | SNORD26 | GGGTGTGCTACGAGGATGATTTGGACATGGAATTCTCTCTTGCTGATGGGTCAGTGGAGAAAGCTATGACGTCCTGAGTAGCCTGCCT | chr.5(-): 300776058- 300776145 |  |
| platypus | SNORD26 | TGATGTAAAAATGAACTCTCTCTTTCTGATGGTTCAGTGGGAGAAATTTTACTAATTTNNNNNNNNNNNN | Contig 18615(-): 11213- 11270 | The 3' end of SNORD26 gene is adjacent to the gap. |
| lizard | SNORD26 | AGTGCTACAGAGATGAGAAACATGAACTCTCTCTTTCTGAAGCTTCAGTGGAGAAAAACATTAGCAGATTCTGAGTAGCACT | scaff.1488 (+): 59558- 59639 |  |
| frog | SNORD26 | TGTGCTGCACTGATGATAAAATGAACTCTCTCTTACTGAGTTTACAGTGGAGACAGACCTTATAATCTGAGTAGCACA | scaff.782 (+): 31689- 31766 |  |
| zebrafish | SNORD26A | TCCAGTGCTACCATGATGACTTCAGAATTCTCTCTTCCTGAATTGCTTTGATGTAACTGTTCTAACCTGAGTAGCATTGGA | chr.7(+): 15717914- 15717994 | The host gene (UHG) has two copies. |
| zebrafish | SNORD26B | TCCAGTGCTACCATGATGACTTCAGAATTCTCTCTTCCTGAATTGCTTTGATGTAACTGTTCTAACCTGAGTAGCATTGGA | chr.7(+): 15956609- 15956689 |
| tetraodon | SNORD26 | TTGCTGCTGTGATGAGACTATGAACTCTCTCTTACTGAACTGGCTTTTGAAGAAACCTCTTTACCAGATTCTGAGCGGAG | Un._random (+): 21177953- 21178032 |  |
| fugu | SNORD26 | GTGCTGCAATGATGAATCTCTGAACTCTCTCTTACTGAATTGGCCTTTGAAGAAATGTCTTTACCAGATTCTGAGTAGAAC | Un. (+): 294291284- 294291364 |  |
| medaka | SNORD26 | GCTCAGTGTGCTGCTGTGATGAGTTCCTGAACTCTCTCTTACTGAATCAGCTTTGAAGAAAGTCTCTTTACCAGATTCTGAGTGGCATATTGAGC | chr. 18(-): 12522707- 12522801 |  |
| mouse | SNORD27 | TGGCCACTTAATGATGAACAATGTAAATGACAAGCATATGGCTGAGTTGCAATGATGTCATCTTACTACTGAAATGGGCTG | chr.19(+): 8799017- 8799097 |  |
| rat | SNORD27 | GTCCACTTGATGATGAATGTATTATAAATGACAAGCATATGGCTGAACTTAGGTGATGTCATCTTACTGCTGAAAAGTGGGCTGA | chr.1(+): 211391506- 211391590 |  |
| dog | SNORD27 | CCACTCTATGATGAACATAAAAAAAAGACAAGCATATGGCTGAACTTAGAAGTGATGTCATCTTACTGCTGAGAAGTGG | chr.18(+): 56869420- 56869498 |  |
| opossum | SNORD27 | TTGCCCACATTGTGATGAGTGTTAAACGACAAGCATATGGCTGAATATTTTTGATGCCATCTTTTCTTCTGAGAAGTGGGCAG | chr.5(-): 300775688- 300775770 |  |
| platypus | SNORD27 | TTTCCCTGATGAAGGTAAAAAAGACAAGCATATGGCTGATGGCATAGTGATGTCACTTTACTTCTGAGAAA | Contig 18615(-): 10643- 10710 |  |
| lizard | SNORD27 | TCCTATGTACAATGATGTGAATAAATAGACAAGCATATATCTGATATCTCTTGATGTCAGCTTTCTGTCTGAACATGGGG | scaff.1488 (+): 59972- 60053 |  |
| frog | SNORD27 | TCATTTGTTGTCAATGATGAATAAAATAGACAAGCATATGTCTGAATAGTATGTGTTGCCATCTTATTTTTTCTGAGAAACTGG | scaff.782 (+): 32748- 32831 |  |
| zebrafish | SNORD27A | GTTAAATGATGATTTCCATGACAAGCATATATCTGAATTCCAGTGATGTTTGCCTATTTATTCTGATAAT | chr.7(+): 15718147- 15718216 | The host gene (UHG) has two copies. |
| zebrafish | SNORD27B | GTTAAATGATGATTTCCATGACAAGCATATATCTGAATTCCAGTGATGTTTGCCTATTTATTCTGATAAT | chr.7(+): 15956842- 15956911 |
| tetraodon | SNORD27A | AGTGGCCGTGGCTGATGAGTTATTCATCTAGACAAGCATATGCCTGACTGGAATGATGCCACCCTTTGAATCTGATAAGGCCACT | Un.random (+): 21178168- 21178252 |  |
| tetraodon | SNORD27B | TACTGAGGCTTTCATGTGATGATTCCAATCATGACAAGCATATGTCTGATTTGCAGTGATGCCATGCTCGATTCTGATCCCCAGCGTGTA | Un.random (+): 21178390- 21178479 |  |
| fugu | SNORD27A | AGTGGCTATTGTTGATGATATTTATATAGACAAGCATATGTCTGATTTTAATGATGCCACATTTTCAATCTGATGGGCCACT | Un. (+): 294291512- 294291593 |  |
| fugu | SNORD27B | ACTGGGGTCTAAACATGATGATTCCAAATAATGACAAGCATATGCCTGATTTTGAAGTGATGCCAACCTTGAATCTGATCCCCCTGGT | Un. (+): 294291735- 294291823 |  |
| medaka | SNORD27 | TTAACTGCTGTGCAAATATGatgattaaaatatttgacaagcATATGCCTGATCATAGTGATGTCATCCTTAAATCTGACTACCTCAACATTAG | chr.18 (-): 12521941- 12522034 |  |
| mouse | SNORD28 | AACTGTCAGATGATTTGAAATGATGACACTGAACACTCCGTGAAGGTGTGAATTTATAGCATGTTAGAGTTCTGATGGCAGTT | chr.19(+): 8799354- 8799436 |  |
| rat | SNORD28 | ACTGTCAGATGATTTGAATTGATACACTGATAACTCCGTGAAGGTGTGAATTTATTATAGCATGTTAGAGTTCTGATGGCAGT | chr.1(+): 211391837- 211391919 |  |
| dog | SNORD28 | ACTGTCAGATGATTTGAATAGATATGCTGATCCTCTGTGAGGTACAACAATTAATAGCATGTTAGAGTTCTGATGACAGT | chr.18 (+): 56869838- 56869917 |  |
| opossum | SNORD28 | TTTAGTCTCACTGTCAGATGATTTGAATCTTGGCTTACTGATGCTCTATGAGGGTCCACATTCTTATAGCATGTTAGAGTTCTGATGGCAGCTCCTGTTGTTAAA | chr.5(-): 300775361- 300775465 |  |
| platypus | SNORD28 | ATTGCCAGATGATTTCAATCCTTATTTTCTGATGCTCCGTGAGGTACAGATATCATAGCATGTTAGAGTTCTGATGGCAAT | Contig 18615(-): 10369- 10449 |  |
| frog | SNORD28 | TGTTCCAAATGATGACTGAATGAAGTCTCGTTCGTCTGAAAATCTTTGAAGAATACTTTTAGCATGGTGGAGTTCTGATGGAGCA | scaff.782 (+): 32965- 33049 |  |
| mouse | SNORD29 | TTTGCTGTTTCTATGATGAATCAAACTAGCTCACTATGACTGCTAATGAAAACACAGGAACACCTGAGAAACCAAA | chr.19(+): 8799575- 8799650 |  |
| rat | SNORD29 | GCTGTTTCTGTGATGAATCAACTAGCTCACTATGACTGCTAATGAAAACACTGGAACACCTGAGAAACTGAAGGGTGGC | chr.1(+): 211392413- 211392491 |  |
| dog | SNORD29 | TCTGCAGTTTCCATGATGAGTCAAACTAGCTCACTATGACTTACACAATGAAAATAAGTGAACACCTGAGAAACTGGAGA | chr.18(+): 56870751- 56870830 |  |
| opossum | SNORD29 | GGTGTGGTTTCAATGATGAGTCT_AACTAGCTCACTATGATTTACATAAAATGAAAAGGCTTGAACATCTGAGAAACTGTACC | chr.5(-): 300774580- 300774661 |  |
| platypus | SNORD29 | GCAGTTTCTATGATGATTCTAACTAGCTCACTTTGATAACTAAAATGAAAACCCTTGAACATCTGAGAAACTGC | Contig 18615(-): 9844- 9917 |  |
| lizard | SNORD29 | TGCTGCAGTTTTAATGATGAAGCTAACTAGCTCACTTTGATATTGTAATGAAAATTTTTGAACAACTGAAAAACTGTATGCA | scaff.1488 (+): 62178- 62259 |  |
| frog | SNORD29 | TGTAGAATTCTGGTTGCTGTGATGAAACTAACTAGCTCACTTTGAATAAAGATGAAAAGTTAAGAACACCTGAGCATCTTTTGCA | scaff.782 (+): 35074- 35158 |  |
| zebrafish | SNORD29A | AATCTCTGTGATGAAGAAACTTAGCTCACTTTGACCCAATGTGAAAAGTCACGAACAACTGAGAGATT | chr.7(+): 15718853- 15718920 | The host gene (UHG) has two copies. |
| zebrafish | SNORD29B | AATCTCTGTGATGAAGAAACTTAGCTCACTTTGACCCAATGTGAAAAGTCACGAACAACTGAGAGATT | chr.7(+): 15957548- 15957615 |
| tetraodon | SNORD29 | GCGCGGTGCAGTGATGATGAAACTTAGCTCACTTTGACCCCATGTGAAACGACAGGAACATCTGAGCATCTGC | Un.random (+): 21178838- 21178910 |  |
| fugu | SNORD29 | CTCGGCGTGGTTCAATGATGATGAAACTTAGCTCACTTTGACCCGATGTGAAACGACACGAACATCTGAGCAGCTGCGCTTCAG | Un. (+): 294292188- 294292271 |  |
| mouse | SNORD30 | CTGAGAACGTACTTTGTGTATATATGATGACTTTCATAGAATCTCGTTCGGCTGATGATTGCTGTTGAGACTTGGAAATCTGATTTTTCTCAG | chr.19(+): 8799812- 8799904 |  |
| rat | SNORD30 | TCCTAAGTACTTCATATATATGATGACTTTCATAGAATCTCGTTCGGCTGATGATTGCTGTTGAGACTTGGAAATCTGATTTTTCTGAGGA | chr.1(+): 211392635- 211392725 |  |
| dog | SNORD30 | CCTAAATCCATGATGACTTATGTGGAATCTCGTTCGGCTGATAACTTGCTGTTGAGACTCGGAATTCTGACTTTCCTAGG | chr.18(+): 56870993- 56871072 |  |
| opossum | SNORD30 | TCCTGAAATGATGCATTCATTGAAGAATCTCGTTCGTCTGAACCCCCCTGTTGAGACGTGCACATCTGACCAGGA | chr.5 (-): 300774335- 300774409 |  |
| platypus | SNORD30 | TTGTGAGGGGAGGATGTGATGAATTTCTTGGAATCTCGTTCGGCTGAACTCTGCTGTTGAGACTTGGAAATCTGATTGTCAGCA | Contig 86933(-): 1643- 1726 |  |
| lizard | SNORD30 | CTGGTTCCCAGTTTCATGATGAATTTGCTTTGGAATCTCGTTCGGCTGAATTTCGCTGTTGAGAAGCAAATCTGAGTCTTTTGGGAACCAG | scaff.1488 (+): 60894- 60984 |  |
| frog | SNORD30 | ATGCCTAATGCACTGACGTGATGAATTCAATCTCATGGAATCTCGTTCGTCTGAATAATTCTGATGAGACTTATTATCTGACTGTGTTCTGTGT | scaff.782 (+): 35633- 35726 |  |
| zebrafish | SNORD30A | TGTAACTTATGATGATTTTCATTGGAATCTCGTTCGTCTGAATTCCTGCTGATATCTTTTAAATACTGATGTTACA | chr.7(+): 15719079- 15719154 | The host gene (UHG) has two copies. |
| zebrafish | SNORD30B | TGTAACACATGATGATTTAGCAGTCTCGTTCGTCTGAAGTACTGTTGAGAAACCTAGCTTTATACTGATGTTACA | chr.7(+): 15719310- 15719384 |
| zebrafish | SNORD30C | TGTAACTTATGATGATTTTCATTGGAATCTCGTTCGTCTGAATTCCTGCTGATATCTTTTAAATACTGATGTTACA | chr.7(+): 15957774- 15957849 |
| zebrafish | SNORD30D | TGTAACACATGATGATTTAGCAGTCTCGTTCGTCTGAAGTACTGTTGAGAAACCTAGCTTTATACTGATGTTACA | chr.7(+): 15958005- 15958079 |
| tetraodon | SNORD30 | TGCTGACAGTGATGAGAGAAACTTGGAATC_CGTTCGTCTGAACAGCTGTTGAGAAACGGAATATCTGACATCGGCG | Un. random (+): 21179071- 21179147 |  |
| fugu | SNORD30 | TGCTGACAATGATGAGATAATATTGGAATCTCGTTCGTCTGAACACCTGTTGAGAAATGGAACATCTGACATCAGTG | Un. (+): 294292425- 294292501 |  |
| mouse | SNORD31 | GCTCACCCTGATGAACTGAATACCGCCCCAGTCTGATAGCTGTGGAGAAAGGTATTTTCTGAGTTGTGAGC | chr.19(+): 8800083- 8800153 |  |
| rat | SNORD31 | GCTCACCCTGATGAACTGAATACCGCCCCAGTCTGATAGCTGTGGAGAAAGGTATTTTCTGAGTTGAGC | chr.1(+): 211392891- 211392959 |  |
| dog | SNORD31 | ACAAGCTCCCCCTCACCAGTGATGAATTCAATACCGCCCCAGTCTGATCACCGTGACTGAAAGGTATTTTCTGAGCAGTGAGCTTGT | chr.18(+): 56871324- 56871410 |  |
| opossum | SNORD31 | CCCACCAGTGATGAGACGATTACCGCCCCAGGCTGATTCCTGTGACTGATAGGTTGTTCTGAGTGGG | chr.5(-): 300774086- 300774151 |  |
| platypus | SNORD31 | GATAGCAATATTCTGTGATGAATCTATACCGCCCCAGTCTGATCATTGTGACTGAAAGGTAATTCTGAGCTGTC | Contig 86933 (-): 769- 842 |  |
| lizard | SNORD31 | GGCTCCAGTGATGAGTTGTTTAACCGCCCCAGTCTGATTTCCAATGACTGAGTGGTCCCTCTGAGTTTAGGAGCC | scaff.1488 (+): 62446- 62520 |  |
| frog | SNORD31A | CTGGCTAAAACCTGATGAGTTCTATTACCGCCCCAGTCTGATTATCTGTGACTGAATGGTATCTTCTGATTGCCTG | scaff.782 (+): 34862- 34937 |  |
| frog | SNORD31B | TAGGTTCAGCTGTGATGATCGTATACCGCCCCAGTCTGATGTCCGTGACTGAGTGGTACAATCTGAGCTGCATTTA | scaff.782 (+): 36349- 36424 |  |
| zebrafish | SNORD31A | TGAACCTATAGGTGTGATGATTTTAGATTACCGCCCCAGTCTGATTTATCTGACTGATTGGTACCCCTCTGAACCTTTTG | chr.7(+): 15716676- 15716755 | The host gene (UHG) has two copies. |
| zebrafish | SNORD31B | TTGTGAACCTATAGGCATGATGATCTTAGATTACCGCCCCAGTCTGATTTATCTGACTGATTGGTACCCCTCTGACCTCTCTTAATGA | chr.7(+): 15719802- 15719889 |
| zebrafish | SNORD31C | GGTATGGACATGATGAGTGACTATTACCGCCCCAGGCTGAAAAGTTATGACTGATTGGTATCCCCTCTGATCCATACT | chr.7(+): 15720019- 15720096 |
| zebrafish | SNORD31D | TGAACCTATAGGTGTGATGATTTTAGATTACCGCCCCAGTCTGATTTATCTGACTGATTGGTACCCCTCTGAACCTTTTG | chr.7 (+): 15716676 15716755 |
| zebrafish | SNORD31E | TTGTGAACCTATAGGCATGATGATCTTAGATTACCGCCCCAGTCTGATTTATCTGACTGATTGGTACCCCTCTGACCTCTCTTAATGA | chr.7(+): 15719802- 15719889 |
| zebrafish | SNORD31F | GGTATGGACATGATGAGTGACTATTACCGCCCCAGGCTGAAAAGTTATGACTGATTGGTATCCCCTCTGATCCATACT | chr.7(+): 15958714- 15958791 |
| tetraodon | SNORD31A | ATCCCACCAAATGATGAGTACGTTACCGCCCCAGTCTGATAACTCGTGACTGATGGGTATTTTCTGACTGAAGCTGTTGGGGT | Un. random (+): 21179587- 21179669 |  |
| tetraodon | SNORD31B | AGGATTGGAGTCAGATGATGAGTTAGTTACCGCCCCAGTCTGATTATTCATGACTGATTGGTATCCTCTGATTGGTATCCT | Un. random (+): 21179790- 21179860 |  |
| fugu | SNORD31A | TTACTCGTATCCGACCAATGATGACTTAAGTTACCGCCCCAGTCTGATGATTCATGACTGATCGGTATTTTCTGACTGAAACGGTTGCAACGCCGTG | Un. (+): 294292917- 294293013 |  |
| fugu | SNORD31B | GGAAAATGTCAAATGATGAGTAAGTTACCGCCCCAGTCTGATGATTCATGACTGATCGGTATTCTCTGAATTAAAATTCC | Un. (+): 294293142- 294293220 |  |
| mouse | SNORD32 | ctgggaGTCCATGATGAGCAACACTCACCATCTTTCGTTTGAGTCTCACGACTGTGAGATCAACCCATGCACCGCTCTGAGActcgccag | chr.7(-): 52382748- 52382837 |  |
| dog | SNORD32 | AGCGGTCAGTGATGAGCAACAATCACCATCTTTCGTTTGAGTCTCACGACCATGAGATCAACCCCATGCACCGCTCTGAGACCTACTAGCT | chr.1(-): 109900146- 109900236 |  |
| opossum | SNORD32 | GGGGGCTGCCGGGCCGGTGACGAGGAATCTTCACCATCTTTCGTTCGAGTCTCGCGACTATGAGACCAAACCATGCACCGCTCTGAGGCCCGGTGTGGCCCCC | chr.4 (-): 404701883- 404701985 |  |
| platypus | SNORD32A | CCGGCCCGTGATGAGGAACATTCACCATCTTTCGTTCGAGTCTTGCGACCGTGAGACCGACTCCATGCACCGCTCTGAGGCC | Contig14108 (+): 13576- 13657 |  |
| platypus | SNORD32B | AGCAGTCCCCAACCCCCGGCCAGTGATGAGGAACATTCACCATCTTTCGTTCGAGTCTCGCGACTGTGAGACCAACTCCATGCACCGCTCTGAGGCCTCTCCTCTGCT | Contig14108 (+): 20524- 20631 |  |
| mouse | SNORD33 | ggcAGCTTGTGATGAGACATCTCCCACTCATGTTCGAGTTGCTCGACTATGAGATGACTCTACATGCACTACCATCTGAGGCTGTt | chr.7(-): 52382233- 52382318 |  |
| dog | SNORD33 | GGTGGCCAGTGATGAGGACTTCTCCCACTTGTATTCGAGTTTCCTGACTATGAGATGACTCCACATGCACTACCATCTGAGGCCACC | chr.1(-): 109899415- 109899501 |  |
| opossum | SNORD33 | GCCTGAGACTGTGATGATAATGCTTACCAAAGCACGTTCGAGTTTGGCGACCATGAGATGAAAAGCTCCATGCACCTCCATCTGAGCTCTCAGGC | chr. 4 (-): 404701254- 404701348 |  |
| platypus | SNORD33A | GGACACCGGTCTGTGATGACGACTGTCCACTTTATTTCGAGTCTCCCGACCTTGAGATCACATCTCCATGCACTACCATCTGAGACCGTCC | Contig14108 (+):14076- 14166 |  |
| platypus | SNORD33B | CATGGGTCTGTGATGATGACTTTCACTTTATTTCGAGTCTCACGACCTTGAGATCACAGCTCCATGCACTACCATCTGAGACCCCTG | Contig14108 (+): 20857- 20943 ; Contig 153541 (+):431- 517 |  |
| lizard | SNORD33A | GGTAGTGGTCTGTGATGATGCCTTTCCACCATTAGTTCGAGTGTCATGGCCATGAGATAACTCTACATGCACTACCATCTGAGGCCACTACC | scaff.76 (-): 3691017- 3691108 |  |
| lizard | SNORD33B | ATGGACTGGTCTGTGATGATGACATTCCACCATTAGTTCGAGTCTCAAGACCATGAGACAACAACTCTTCATGCACTACCATCTGAGAACCAATCCAT | scaff.76 (-): 3688798- 3688895 |  |
| frog | SNORD33A | CAGCGATGTCTGTGATGAGTTGAGTTCTTTGAGTTGCGCTATGAAACCACCCTACATGCACTACCATCTGAGACTCGCTG | scaff.709 (+): 222642- 222721 |  |
| frog | SNORD33B | GGGGCAGAGGTCAATGATGATGGTTCTTGTTCGAGTTTAATGCCAATGAGACAACTTGCATGCACTACCATCTGAGACCTACGTGTGGCGCCCC | scaff.709 (+): 222881- 222974 |  |
| zebrafish | SNORD33A | AGCTTTTTGTCGGTGACGAGCTCGCAACCACAGTTCAGTATTCGAGTTTAACGACCATGAGAATACTTTACGTGCACTACCATCTGAGACAAAAAGCT | chr.17(-): 2088795- 2088892 |  |
| zebrafish | SNORD33B | GCTTTTTGTCAGTGATGTTTTTCCACAGTAAGTATGAGTTTAACGACCATGAGTCCACTTCATGCACTACCAGCTGAGACAAAAAGC | chr.17(-): 2087048- 2087134 |  |
| fugu | SNORD33A | GTCTGTCAGTGATGATATGTTTGTTTTTTTGCTTGAGTTTAGCGACCATGAGAAAACTCACATGCACTACCATCTGAGACGGAT | Un(+): 36446242- 36446325 |  |
| fugu | SNORD33B | CATGTCTGTGATGTGCTTAACCAATATAATTCGAGTTTCATTGACAATGAGACAACCTCACATGCACTACCATCTGAGACATC | Un (+): 36446517- 36446599 |  |
| mouse | SNORD34 | gCGTCTGTGATGTTCTGCTATTACCTACATTGTTTGAGCCTCATGAAAACCCCACTGGCTGAGACGC | chr.7(-): 52381973- 52382039 |  |
| dog | SNORD34 | TGGCGTCCATGATGTTGCACAGTTGTACCTACATTGTTTGATCCTCATGAGAACAGCATTGGCTGAGACGCTG | chr.1(-): 109899136- 109899208 |  |
| opossum | SNORD34 | GGCGTCTGTGATGTCTCCTTTACCTACATTGTTTGACATCTCCCTGAGAAAACATTGTTGCCTGAGACGCT | chr.4(-): 404701007- 404701077 |  |
| platypus | SNORD34A | GGGGTCAGTGATGCTGCACAATTACCTACATTGCTTGATTTTTCCCCATGAGAACATAACTGTGACTGAGGCCCC | Contig14108 (+):14370- 14444 |  |
| platypus | SNORD34B | GGGCCGATGATGCTGTTCAATTACCTACATTGTTTGATTTTCCTGTGAGAAAACAGCTATATCTGAGGCCC | Contig14108 (+):21139- 21209; Contig 153541 (+):713-783 |  |
| frog | SNORD34A | AATGTCAGTGATGTTTTTTTTACCTACATTGTTTGATTATCTAAATGAAAAACATCTTAGACTGAGACATT | scaff.447 (-): 770234- 770304 | U50HG is the host gene. The nucleotide complementary to the SNORD34 RNA target is shown in red. |
| frog | SNORD34B | TCAGATGTGTCTATGATGCTTTTTCTTTACCTACATTTTGATTTTCTAAATGATAAAAATGTTCACAGACTGAGACATGATTGA | scaff.7(+): 1359067- 1359150 | The unannotated host gene (EST [**BX757900**](http://www.ncbi.nlm.nih.gov/entrez/query.fcgi?cmd=Search&db=nucest&term=BX757900&doptcmdl=GenBank&tool=genome.ucsc.edu)) has two copies. In contrast to SNORD34A, a modification of a neighboring site in rRNA can be guided. |
| frog | SNORD34C | TCAGATGTGTCTATGATGCTTTTTCTTTACCTACATTTTGATTTTCTAAATGATAAAAATGTTCACAGACTGAGACATGATTGA | scaff.7(+): 1373310- 1373393 |
| zebrafish | SNORD34 | TTCACAGATTAATTCTGTCAATGATGCCACTTTTCTGCCTTGTTTGAGTGTCGACTGAGAATACACAAAATTAACTGAGACATCTTTACTTCTGGAG | chr.17(-): 2087305- 2087401 |  |
| mouse | SNORD35A | ctGGCACATGATGTTCTTATTCTCACGATGGTCTTCGGATGCCACAGTTAGGGCAGTGCCGATAATGCCAAAGGCTAAGCTGATGCCAG | chr.7(-): 52381719 52381807 |  |
| mouse | SNORD35B | TggcaagtgatgtctgTTCTCACGATGGTCTTCaGATGtcctcTAGGGCActgctgagacagccagttgacaaagctgatgcca | chr.7(-): 52378399- 52378482 |  |
| dog | SNORD35A | CTGGCAGATGATGGCTCTTTCTCACGATGGTCTGCGGATGCCACAGTGGGCAGTGCCGATAATGCCAATGGCTCAGCTGATGCCA | chr.1 (-): 109898865- 109898949 |  |
| dog | SNORD35B | TGGCAGATGATGTCTTTTCACGATGGTCTTCAGATGCCCACCGCGGGCACTGCTGAAAAAACCACTTGGCACAGCTGATGTCA | chr.1(-): 109889155- 109889237 |  |
| opossum | SNORD35A | GGCAGGCGGCATGTGATGGCTTTTCTCACGATGGTCTTCAGAGGCTCCCCCTTGGGGTCACTGCTGATCTGCCTCAGGGCTTAGCTGATGCCGATGTC | chr.4(-): 404701557- 404701654 |  |
| opossum | SNORD35B | CCTGGCAGATGATGTTTGTCATCACGATGGTCTTCGGATGCCTAAGGGTACCTGCCGACAAAGCCACAAGGCTCAGCTGATGCCAGG | chr.4(-): 404700447- 404700533 |
| platypus | SNORD35A | TCCCTTCCTGGCAGATGATGTCTATTCTCACGATGGTCTTCAGATTGCCCCCCGTGGGCGCTGGCGACACCGCCAAACGGCTTAACTGATGCCAGGGGGA | Contig14108 (+):19343- 19442 |  |
| platypus | SNORD35B | TCCCTCTGGCAGATGATGTCCATTCTCACGATGGTCTTCAGATTGCCCCCCGTGGGCCCTGATGACACCGCCGAAAGGCTCAGCTGATGCCAGGGGGA | Contig14108 (+):27784- 27881; Contig66837 (-):211-308 |  |
| chicken | SNORD35A | TCCTTGAGCTGCACATGATGCCATTCTCACGATGGTCTTCCAAGCCTCACGGTGAAGGGGCTGTGACGACAACGCCACATGGCTTTGCTGATGCCTGGTACAAGGA | chr.1(-): 199345577- 199345682 |  |
| chicken | SNORD35B | TTGTTTGCTGGTGGTGATGTCTGAACTCACGATGGTCTTCAGAGTCCCTGTTGGTGAGGGGACTGCTGACAATGCCAGTGGCAA | chr.1(-): 199344919- 199345002 |
| lizard | SNORD35A | CTCCTCTGGCGCTGACACATGATGTTCATTCTCACGATGGTCTTCCAAGCCTCACTGGTGAAAGGGGCTTTGACGAGAGCGGCCAAGAGGCCATAACTGAAGTCAGCTCATGCCAGAGGGG | scaff.985 (-):40036- 40156 |  |
| lizard | SNORD35B | TCCCAAAGTGCAAATGATGTCTTTCTCACGATGGTCTGCTGAACCCCGTGGTGAAGGGGTTCTGGTGAAACTGCCTTGTGGCTTAGCTGATGCCTTTGGGA | scaff.985 (-):38590- 38690 |  |
| lizard | SNORD35C | TGAGGGTCTTGAGCAGGGGCCTTGGTGATGATGCCTCAATTCACGATGGTCTGCTTAGCCCTTCCTTTCACAGAGAGCGAGGGCTGCTGAAAATGCCATTCTGGCCATTGCTGAGCCATGGCCCTTGCTCAGATCTGCCCTCA | scaff.985 (-):36475- 36617 |  |
| frog | SNORD35A | TGCAAATGATGTTTATACTCACGATGGTCTTCCAAGCCTCGTGGTGAAGAGGTTTTGACGAAAGTGCCAGATGGCTTTGCTGATGCTTTGCA | scaff.447 (-): 769478- 769569 |  |
| frog | SNORD35B | TAAGCAGCAATATGATGTATTTTCTCACGATGGTCTTCCAAGCATTGATGCTTTGACGAAACTGCCATTTGGCTTTGCTGATTGCTGAGCTTA | scaff.447 (-): 768804- 768896 |
| frog | SNORD35C | GGGCTCTGCAAATGATGTTTACTCACGATGGTCTTCCAAGCCTCACTGGTGAAGAGGTTTTGACGATATTGCCAAATGGCTTTACTGATGCTCTGAAGTTT | scaff.447 (-): 768531- 768631 |
| zebrafish | SNORD35A | GCTGCAAATGATGTTTTTTCTCACGATGGTCTTCCAAGTCTGTCAGGCTCTGACGACACTGCCTTATGGCATCACTGATGCAGC | chr.3(+): 35597580- 35597663 |  |
| zebrafish | SNORD35B | ACTTTGTTGCAGATGATGTCTTTCTCACGATGGTCTTCCTAAACTCTTAAAGAGCTCTGAGGAAACTGCCTAATGGCAATgctgatgcagtggagt | chr.3(+): 35597960- 35598055 |
| zebrafish | SNORD35C | GGGGTGCAGGTGATGTCTTTTCTCACGATGGTCTTCTGAACCCTTTGGGTTTTGAGGATGATGCCTTATGGCTTTACTGATGCTTCCC | chr.3(+): 35598236- 35598323 |
| zebrafish | SNORD35D | GCTGCAAATGATGTTTTTTCTCACGATGGTCTTCCAAGTCTTTCAGGCTCTGACGACATTGCCTTATGGCATCACTGATGCAGC | chr.3 (+): 35598623- 35598706 |
| fugu | SNORD35A | CTGCAAATGATGTCTTTCTCACGATGGTCTTCCAAACCGCAGGGTTCTGACGACACTGCCTAATGGCAATACTGATGCAG | Un. (-): 49181104- 49181183 |  |
| fugu | SNORD35B | CTGCAAATGATGTTTTTAATCACGATGGTCTTCCAAACCTTCTGGGTTCTGAGGATAGTGCCTGATGGCAATGCTGATGCAG | Un. (-): 49180785- 49180866 |
| fugu | SNORD35C | TGATTTAATTAGCTGCAAATGATGTCTTTCTCACGATGGTCTTCCAAGCTCAAGATGCTCTGACGACACTGCCTTTGGCAAACTCTGATGCAGCGTTAAATCG | Un. (-): 49180368- 49180470 |
| human | SNORD36A | TTGCAATGATGTGAATCTCTCACTGAATTCAACCTTGAAGTGCGAATCCATGAGCTTTTTAACCCTGAGCAA | chr. 9(+): 135207132- 135207203 | The first antisense element is disrupted. |
| human | SNORD36B | GTTGCAGTGATGTAAAATTTCTTGGCCTGAAATTACTGTGAAGAGTAAAACCGAGCTTTTTAACACTGAGT | chr. 9 (+): 135206770- 135206839 |  |
| human | SNORD36C | TTGCCAATGATGGTTAAGAATTTCTTCACCTGAATAAACCATGTGGTCAGCATTGCATCTGAGGCAAA | chr. 9(+): 135207522- 135207589 | The second antisense element is disrupted. |
| mouse | SNORD36A | AGGCTGTGCAGTGATGTGTGAATTTCTTCACCTGAAAATATTGTGAAGAGTAAATCGAGCTTTTTAACCCTGAGTCGCAGCTT | chr. 2(+): 26767733- 26767815 |  |
| mouse | SNORD36B | TGTGGCTCTTGCAATGATGTCAATCTTTGACTGAAGTGACCTTGAAGTGCAATTACTGAGCTTTTTAACCCTGAGCAATTGCCACA | chr. 2(+): 26768042- 26768127 | The first antisense element is disrupted. |
| mouse | SNORD36C | TTTGCCAGTGATGCTTAGGAATTTCTTCACCTGAATCAACTATGTGGTCACACTGTTGTCTGAGGCAAA | chr. 2 (+): 26768367- 26768435 | The second antisense element is disrupted. |
| cow | SNORD36A | ggctgttggcaatgatgcatgaatttcTTCACCTGGAATCACTGTGGAGAGTAAAATCCGAGCTTTTTAACCCTGAGTCACAGCC | chr.11(+): 108186233- 108186317 |  |
| cow | SNORD36B | TTGCAATGATGTGAACTTCTTACTGAATTAAACCTTGAAGTGCAAACACATGAGCTTTTTAACCCTGAGCA | chr.11(+): 108186641- 108186711 | The first antisense element is disrupted. |
| cow | SNORD36C | TTTGCCAATGATGGTTATGAATTTCTTCACCTGAATAAGCCATGTGGTCATGCTATATCTGAGGCAAG | chr.11(+): 108187038- 108187105 | The second antisense element is disrupted. |
| opossum | SNORD36A | AGCTATTGCAATGATGTATGAATTTCTTCACCTGAAATAACTTGTGTAGAACAAATACACGAGCTTTTTAACCCTGAGGCATAGCT | chr. 1 (+): 460618676- 460618761 |  |
| opossum | SNORD36B | TTGGCTTTTGCAATGATGTGTGAATTTCTTCACCTGAATTAATAGCATGAAGAATAAATACACGAGCTTTTTAACCCTGAGCAAAGGCCAA | chr.1(+): 460619147- 460619237 |  |
| opossum | SNORD36C | GAACCTTTTGACAATGATGTGTACAGAATTTCTTCACCTGACAAGACATGTAGTAATATTAGACCCTGAGACAAAAGGTTC | chr1(+): 460619624- 460619704 |  |
| platypus | SNORD36A | GGCTACTGCAGTGATGTAAGAATTTCTTCACCTGAAAGAACCGTGTCGATCGAACGTACGAGCTTTTTAACCCTGAGCCGTAGCC | Contig 11099(+): 20577- 20661 |  |
| platypus | SNORD36B | GGCTTTTGCAATGATGTATGAATTTCTTCACCTGAAAAAGAATGAAGAGCAAATACACGAGCTTTTTAACCCTGAGCAAAGGCC | Contig 11099(+): 20948- 21031 |  |
| platypus | SNORD36C | CCTTTTGCCAATGATGCGGAGAATTTCTTCACCTGAACGATTATGTGGCCGCTTTACGATCCTGAGACAAAAGG | Contig 11099(+): 21406- 21479 | The second antisense element is disrupted. |
| platypus | SNORD36D | TGGCTTTTGCAATGATGTATGAATTTCTTCACCTGAAAAAGAATGAAGAGCAAATACACGAGCTTTTTAACCCTGAGCAAAGGCC | Contig 11099(+): 22209- 22293 |  |
| platypus | SNORD36E | CCTTTTGCCAATGATGCGGAGAATTTCTTCACCTGAACGATTATGTGGCCGCTTTACGATCCTGAGACAAAAGG | Contig 11099(+): 22669- 22742 | The second antisense element is disrupted. |
| chicken | SNORD36A | TGCTATGGCAGTGATGTATGAATTTCTTCACCTGAGCTCAAAGTGAAGAGCGAAATAGACGAGCTTTTTAACCCTGAGCTTTAGCA | chr.17(+): 7533498- 7533583 |  |
| chicken | SNORD36B | TGGCCCTTGCGATGATGTATGAATTTCTTCACCTGAATCAACAATGAAGAGCAAAATGAGCTTTTTAACACTGAGCAATTGCCA | chr.17(+): 7533943- 7534026 |  |
| lizard | SNORD36A | GCTACGGCAATGATGTATGAATTTCTTCACCTGAACTGCAGACTGAAGAGCAAAAATTAACGAGCTTTTTAACCCTGAGCCGTAGC | scaff.1674(-): 36549- 36634 |  |
| lizard | SNORD36B | GCTGCTGCAATGATGTGTGAATTTCTTCACCTGAACTCCTCTCTGAAGATCTAAACCATACGAGCTTTTTAACCCTGAGCAGCAGC | scaff.1674(-): 36198- 36283 |  |
| frog | SNORD36A | GCTATTGCAGTGATGGCTGAATTTCTTCACCTGAAACCACTTGTGTGGATGTAAAAACACGAGCTTTTTAACACTGAGCAGTAGC | scaff.878 (-): 18172- 18256 |  |
| frog | SNORD36B | TTTGGATCGCAATGATGTAATTGAATTTCTTCACCTGACTGGATAATGAAGATTAAATCCGAGCTTTTTAACCCTGAGCTGTCCAAG | scaff.878 (-): 17855- 17941 |  |
| frog | SNORD36C | GTCCCTCCTGTGCAATGATGTGCATAGAATTTCTTCACCTGAATGACCGTGTGGCACTCAGAGCTTTTTAACCCTGAGCGGCAGTTGGGGGGC | scaff.878 (-): 17499- 17591 |  |
| zebrafish | SNORD36A | AGTGGTTGTCTGCAATGATGTCTTGAATTTCTTCACCTGAATTCAAACTGAAGATCAAAATACGAGCTTTTTAACCCTGAGCAACAACCACT | chr.5 (+): 27275197- 27275288 |  |
| zebrafish | SNORD36B | CTACAGCTATGATGTCTGAATTTCTTCACCTGAACAACCATGTGGTTTAAATGAGCTTTTTAACCCTGAGCTAAATGG | chr.5(+): 27275560- 27275637 |  |
| tetraodon | SNORD36A | CGAATGCAGTGATGACTTGAATTTCTTCACCTGATGGCTTCAGTGATGCGTATTGAGCTTTTTAACCCTGAGCATTGG | chr.4(+): 6203306- 6203383 |  |
| tetraodon | SNORD36B | GGTTCCTTGCAATGATGTTTGAATTTCTTCACCTGAAGTCACTGTGTAGATCAAAACAGACGAGCTTTTTAACCCTGAGCAAGGGCC | chr. 4 (+): 6203581- 6203667 |  |
| mouse | SNORD37 | CCCACCATGATGACGGAAATTCTTCACTTTGACCTGATGTCTGTTGAAGAAACTCAGTGTCTGAGACTGTGGG | chr.10 (+): 80641693- 80641765 |  |
| opossum | SNORD37 | TTATTTATGTTCATGATGATGGAATATTTCTTCACTTTGACCTGATGTCTAATGAAGAAATCTAACCTCTGAGAAGCATTGTAA | chr.3 (-): 440394414- 440394497 |  |
| platypus | SNORD37 | CTCCACATGATGATTAAACATTTCTTCACTTTGACCTGATGTCTACTGAAGAAATCCAGTATCTGACAAG | Contig 14104(-): 10027- 10096 |  |
| chicken | SNORD37 | GCAGTGCGTGATGATGGAAAATTTCTTCACTTTGACCTGACTTGTTTGATTGAAGAAATTTGAGTATCTGACGCGAGCTGAGCTCGTTG | chr.28(+): 1055202- 1055290 |  |
| frog | SNORD37 | ATCTGAGGAGACAAATGATGACTCATTTCTTCACTTTGACCTGAAAGCAAAGATGAAACCATATAGTCTGACTTTCCTCAGAT | scaff.768 (+): 160237- 160319 |  |
| tetraodon | SNORD37 | ATTGTGCCTGCAAATGATGAGTTAAAACATT_CTTCACTTTGACCTGATTGACAGGTGATGATAAATTTGACTGAACCAGGCACGGT | chr.1 (+): 13802696- 13802781 |  |
| mouse | SNORD38A | TGCCTTCTTGTGATGAAAATACTGTCCAGTTCTGCTACTGAAGGAATGAGATGAACACTTTAGTGCTGAAGAAGGCA | chr.4(-): 116827116- 116827192 |  |
| mouse | SNORD38B | ACTGTCTCGGTGATGAGAACTTTGTCCAGTTCTGCTGCTGATCTCTTAAGTGAGGATGAAGTTATCTGAGGAGACGGT | chr.4(-): 116826694- 116826771 |  |
| rat | SNORD38A | CCTTCTTGTGATGAAAATACTGTCCAGTTCTGCTACTGAAGGAATGAGATGAATACTTTAGTGCTGAAGAAGGC | chr.5 (-): 137467409- 137467482 |  |
| rat | SNORD38B | GCTGTCTCAGTGATGAGAACTTTGTCCAGTTCTGCTGCTGACTTCTAAGTGAGGATGAAGTGTATCTGAGGAGACAGT | chr.5(-): 137466981- 137467058 |  |
| dog | SNORD38A | GCCTTCTTATGATGAAAACTGTCCAGTTCTGCTACTGAAAGGAAAGAGATGAAAGCCTTTAGTGCTGAGGAAGGC | chr.15(-): 18548774- 18548848 |  |
| dog | SNORD38B | GTCTTGGTGATGAAAACTTTGTCCAGTTCTGCTACTGACTTTAAAGTGACGATAAAGTATATCTGAGGAGAC | chr.15(-): 18548271- 18548342 |  |
| opossum | SNORD38A | GCCTTCTGATGAGGAGAACCTTGTCCAGTTCTGCTACTGATGTATCAGAGATGACAGCTTTGTGTGCTGAAGAAGGTC | chr.2(-): 9661315- 9661392 |  |
| opossum | SNORD38B | GAGTCTCCTAGTGATGAGAACCCTGTCCAGTTCTGCTACTGAGGCTGAGTGACGACAGAGGTTTGTCTGAAGAGACTC | chr.2(-): 9660784- 9660861 |  |
| platypus | SNORD38 | AGCCTTCACGTGATGATAATATTGTCCAGTTCTGCTACTGAAGGGACAGCGGTGACACCCTTAGAATCTGAAGAAGGCT | Ultra56(-): 5367576- 5367654 |  |
| chicken | SNORD38A | GTCTTCTAATGATGATACTTCTGTCCAGTTCTGCTACTGAAGGGAGAGCGATGACACTTGTGATGCTGAGGAAGAC | chr.8(+): 21482676- 21482751 |  |
| chicken | SNORD38B | TGAAGTCTTCTGGTGATGAGACCTTTGTCCAGTTCTGCTACTGAATTTGAGGGATGACTGTTTGGAGATCTGAAGAGGACTTCA | chr.8(+): 21483923- 21484006 |  |
| lizard | SNORD38A | TGTCTTCTGGTGATGATACCTTTGTCCAGTTCTGCTGCTGAATGAAGAGCGATGACATCTTTTCGTATCTGAAGAAGACG | chr.4(-): 6843492- 6843571 |  |
| lizard | SNORD38B | TGAAGTCGACCTTATGATGAGAAATTTGCCCAGTTCTGCTACTGAATGGAAGTGGTGACAATTGGTTTCTGAAGGTGACTTCA | chr.4 (-): 6842126- 6842208 |  |
| frog | SNORD38A | TCTTCTGCTGATGATAACCTTGTCCAGTTCTGCTACTGAGACTGTACGATGATATTCCTTAATCTGAAGAAGA | scaff.261 (-): 1536258- 1536330 |  |
| frog | SNORD38B | TGAGTTACGTCTTCTGCTGATGATAACTTTGTCCAGTTCTGCTACTGAAATTATGTGGTGATATTTGTCAACCTGAAGAAGGCAACCTGA | scaff.261 (-): 1533578- 1533667 |  |
| frog | SNORD38C | TTGTCTTCTGCTGATGATAACTTTGTCCAGTTCTGCTACTGAAATTATGTGGTGATATTTGTCAACCTGAAGAAGGCA | scaff.261 (-): 1533319- 1533396 |  |
| frog | SNORD38D | GTCTTCTTCTGATGATAACCTTGTCCAGTTCTGCTACTGAAACTATGCGATGATATTTCTGAATCTGAAGAAGAC | scaff.261 (-): 1529566- 1529640 |  |
| zebrafish | SNORD38A | AGTTCTTTCGGTGAAGATAATCTGACCAGTTCTGCTACTGAATGTTCATGATGGATATCTATGTTCCAACTCTGAGAAAGAATT | chr.2(+): 22150891- 22150974 |  |
| zebrafish | SNORD38B | TTTATAGTCTTTCTGTGACGATAACTTTGTCCAGTTCTGCTACTGAAATATAAGTGATGCAGTTTAAGACTCTGAGGAAGGCTTGTAAA | chr.2(+): 22151247- 22151335 |  |
| zebrafish | SNORD38C | TAATGTGTTCATGATGAAACCTTTAACCAGTTCTGCTACTGAAAAAATGGTGCTGAGCTTTTAACCTTTCTGACATGCACTGAAAAGCTG | chr.2(+): 22151564- 22151653 |  |
| tetraodon | SNORD38 | ATCTTTCAGTGATGATAACTTTGTCCAGTTCTGCTACTGAATGAAAGTGGTGATAGCAAAGACTCTGAGAACGAT | Un_random (+): 17144386- 17144460 |  |
| mouse | SNORD41A | CTGGGAAATGATGATGCCTGTGACTGCTGACAAGACTCTGATGTCTGTTGTATTCGTACTGGCTGATCCTGG | chr.8(+): 87574105- 87574176 |  |
| mouse | SNORD41B | TGGCCTTAAGTGATGGAGACATACCCTGGGAGGCCCATGACCGGACTGGGATGTATTCGTACTGTCTGATGGGGCTG | chr.8(+): 87577486- 87577562 |  |
| cow | SNORD41A | CGGGAAGTGATGATACTTATGACTCCTGACAGTGCGCTGATTTCTCACCGTATTCGTACTGGCTGATCCCG | chr.7(+): 11068626- 11068696 |  |
| cow | SNORD41B | GGCCTCAAGTGATGGAGATGACAACCTGGGAGTACTGTGACCGAACTGGGATGTATTCGTACTGTCTGATGGGGCC | chr.7(+): 11070518- 11070593 |  |
| opossum | SNORD41A | CTCTTGGGAAATGATGCATCCTTCTCAGCCTGAGTGCTCTCTGAAGGTTTCTTGTATTCATACTGTCTGATCCCAGGAG | chr.3(+): 429281582- 429281660 |  |
| opossum | SNORD41B | CTTGGAACATGATGCAGTTATCATCCCTGAGCCTATGGTGATGGCTATTGTATTCATACTGTCTGATCTCCTGGG | chr.3(+): 429282006- 429282080 |  |
| opossum | SNORD41C | CCTTGGATGTGATGGACAACTTACTGTTTGATGTCAAACTGATCTTTGTAGTATTCATACTGTCTGATCCTGGG | chr.3(+): 429282796 -429282869 |  |
| lizard | SNORD41A | TTCATGGAAGTGATGCATAAACATATAACCTTCACCCTGATGACAATGATGGTTGGTTGTATTCGTACTGTCTGATCCATGGA | scaff.411 (+): 468738- 468820 |  |
| lizard | SNORD41B | GGCTTTCTTTGGAAGTGATGAAGTTGACGTTTGAGCATTGCAGTGATGTGTCTTGTGTTCGTACTGTCTGATCCAGAGAAATGCC | scaff.411(+): 471587- 471671 |  |
| frog | SNORD41A | CCCTGGAAATGATGCATGTAATTAACCTGATATAATATGAGGGTTATTGTATTCATACTGTCTGATCCAGGG | scaff.377 (+): 227522- 227593 | A complementary substitution in the antisense element (shown in pink). |
| frog | SNORD41B | TTCGGAGATGATGCAGCAGAACTTAACCCGACCCAAAGCTGGGGGTACTTGTATTCATACTGTCTGATCCGGG | scaff.377 (+): 229353- 229425 |
| frog | SNORD41C | TCAGTTCCGTGGACATGATGCAGACTGACTGGCCTGACCCCTGAGGGTTCCACGTATTCATACTGTCTGACCCGGGCTGA | scaff.377 (+): 230676- 230755 |
| frog | SNORD41D | TCTGGAAGTGATGCACATTGTGAATATTGAAGTTATCTCATGATTCCCTTTGTATTCATACTGTCTGATCCAGA | scaff.377 (+): 232889- 232962 |
| zebrafish | SNORD41A | CTCAGGTGTGATGATAAAATTTCTCTTGACGTTCTCCGTGATTTTCAAGTATTCATACTGTCTGACCTGAG | chr.3(+): 15856572- 15856642 |
| zebrafish | SNORD41B | TTGTGGAGATGATTACAAACTGTATCATGAATGTCAGTGATGTTCAAGTATTCATACTGCCTGATCCACAA | chr.3(+): 15857793- 15857863 |
| fugu | SNORD41A | GTGCACATGGGCTTGAGAAATGATGCAAAATGAGCAACACCTGAGCTTTGCTGATGTTGGTTGTATTCGTACTGTCTGATCTGAAGCCCATGTGCGT | Un (+): 210227764- 210227860 |  |
| fugu | SNORD41B | CTGGAAATGATGCACAAATAAAATAACCTGACAACTAATGCAGATATTTGTATTCGTACTGTCTGATCCAG | Un (+): 210228842- 210228912 |  |
| mouse | SNORD42A | TGTGCAAATGATGGAAAACTCAATGTTTGGAAAAGAATGACACCAACAAAGGAACCACTGATGCTG | chr.11(-): 77994801- 77994866 |  |
| mouse | SNORD42B | TGGTATCGTGCATATGATGGAAAAAATCTGAAGTCTCCTGAGACCTGTGATGTCTTCAAAGGAACCACTGATGCACATGCTG | chr.11(-): 77996552- 77996633 |  |
| dog | SNORD42A | TCTTATGCAAATGATGGAAAAGTCACTATTTAGGAAAGAATGACATGAACAAAGGAACTGCTGATGTGCCAGA | chr.9(+): 46243903- 46243975 |  |
| dog | SNORD42B | CGTGCATATGATGGAAAAATCTTAATCTCCTGAGACTTGTGATGTCTTCAAAGGAACCACTGATGCACG | chr.9(+): 46241312- 46241380 |  |
| opossum | SNORD42A | TTCATGCAAATAATGGAAAAATATCATTTTACCGGAAAAAAAATGATGCTAACAAAGGAACCACTGATGCATGAG | chr.2(+): 506650225- 506650299 |  |
| opossum | SNORD42B | ATGCCAATGATGGAAACATGTATTTTCCTGAGAATACTGATGTTGTCAAAGGAACCACTGATGGCAT | chr.2(+): 506648472- 506648538 |  |
| platypus | SNORD42A | TGCCGTGCAGATGATGGAAAAGTTTTTTTGCTGACAAAAGTGATGTTAACAAAGGAACCGCTGATGCACGTCGA | Ultra 497 (-): 338422- 338495 |  |
| platypus | SNORD42B | GCCCGTGTCAATGATGGAAAACTTTCCTGGCTGAGAACAGTGATGTTGTCAAAGGAACCACTGATGCACCGGC | Ultra 497 (-): 343850- 343922 |  |
| chicken | SNORD42 | TGTGCAGATGATGTGAACAATTTTTACCACTGAGTAAACTGATGTTCCCAAAGGAACCACTGATGCACA | chr.19 (+): 5762412 - 5762480 |  |
| lizard | SNORD42 | GTGGAAGGTTTCTCTGCGCAGATGATGGAAAACACTTTCGAACTGAGCTCAGTGATGCTTTCAAAGGAACCACTGATGCACCGAGAGCCTTCCAT | scaff.2905 (+): 4254- 4348 |  |
| frog | SNORD42A | AGTTTCTTGTGCGAATGATGGAAAACATATTAATGCTGAAAATATTGATGTTTCCAAAGGAACCACTGATGCATAAGTTAAAACT | scaff.817 (-): 80290- 80374 |  |
| frog | SNORD42B | TCTGCAGTTTTAAACTTTGCAAATGATGGAAATGTTATTTGGACTGAATACATTGATGAGCTCAAAGGAACCACTGATGCACAGTATAACTGCAGA | scaff.817 (-): 79888- 79983 |  |
| zebrafish | SNORD42A | CTGTGTAATGATGTAAAATAATTATTTCTCTGAAATGTGATGTTCTCAAAGGAACCACTGATACACAG | chr.21(-): 24551849- 24551921 |  |
| zebrafish | SNORD42B | GAAGCTCTGCATATGATGTAAATTAAGTTTCTGCCTGAATGATTGTGATGCATTCAAAGGAACCACTGATGCTCTGTTTT | chr.21(-): 24551563- 24551642 |  |
| tetraodon | SNORD42 | CTGTGCAAGTGATGCAAACTTTTACTATAGCTGAATGTATGTGATGCATTCATCAAAGGAACCACTGATGCCAG | chr.7 (-): 3735359- 3735432 |  |
| mouse | SNORD43 | gaagcCACAAATGATGAACCTTTTGACGGGCGGACAGAAACTCTGTGCTGAATGTCAAGTTCTGATTtggcttt | chr.15(-): 79913282- 79913355 |  |
| opossum | SNORD43 | GGCTACACGTGATGAAATCTATGACGGGCGGACAGAGAAAACACGTGCTGAACGTCATATTCTGATGGTAGCC | chr. 8 (+): 60577867- 60577939 |  |
| platypus | SNORD43 | ACAAGTGATGAGACCCTTGACGGGCGGACATAGGAAACCGCCTCTGGCGCTTGATGCTGATTGT | Contig 4527 (-): 35314- 35377 |  |
| chicken | SNORD43A | AACAGGCTACGTATGATGATACTTCGACGGGCGGACATAAGGAAATCGCCTCTGGCGCTTGTTGTTGAGTGTCGAGTCCTGACCGTAGCCCTGTT | chr.1(+): 52601608- 52601702 | The gene lies more than 3.7 kb from the 3' end of its host intron. |
| chicken | SNORD43B | AACAGGCTACGTATGATGATACTTCGACGGGCGGACATAAGGAAATCGCCTCTGGCGCTTGTTGTTGAGTGTCGAGTCCTGACCGTAGCCCTGTT | chr.1(+): 52606324- 52606418 |  |
| lizard | SNORD43 | ACTACAAATGATGATTTACTCGACGGGCGGACATAAAGAAATCGCCTTTGGCGCTTGTTGCTGATTGTCGAGTACTGAATGTAGT | scaff.242 (+): 1161173 - 1161257 |  |
| frog | SNORD43 | TGGGCAAAATTTAAAGGATGAAACTTTTTCGACGGGCGGACAGACTGATTGCCAGAGGCAGTCTCTGTTGAATGTCGAATTCTGACTGCTTGCCTA | scaff.69 (+): 768349- 768444 |  |
| zebrafish | SNORD43 | CTGCCATTGATGATACTCTCGACGGGCGGACATATTTGGGCCATGAGCCCTTGCTGAATGTCGAGTACTGAGAGCAG | chr.3(-): 27586049- 27586125 |  |
| tetraodon | SNORD43 | TGACGGCGTGCCTGTGATGAGACCCTCGACGGGCGGACATAGTGGCTCTACAGCCCCTTTGCTGAATGTCGAGTACTGAGCCACAGCCGGTCA | chr.3 (+): 14526176- 14526268 |  |
| mouse | SNORD44 | GCCTGCATGATGACGAACAAATACTGACTACCTGAAGATCTTATTAGCTCTATCTGATGGT | chr.1(+): 162966812- 162966872 |  |
| rat | SNORD44 | AGCCTACATGATGACAAACAAATGCTGACTAATGTGAAGATCTTAATTAGCTCTATCTGATGTTGGGCT | chr.13 (+): 76596305- 76596373 |  |
| dog | SNORD44 | GCCTGTATGATGATAAGCAAATACTGACTGAACATGAAGGTCTTAATTAGCTCTACCTGACATCAGGC | chr.7(+): 28330728- 28330795 |  |
| opossum | SNORD44 | TCAGCCTGTATGATGAAAAGCCAATAGACTGACTTAATCATGGAGGTCTAATTAGCTCTACCTGATTTCAGGCATTGA | chr.2(-): 66318939- 66319016 |  |
| platypus | SNORD44 | TAGCCTGTATGATGATGCTGCAAAGACTGATTCAATCATGATGGTCTTATTAGCTCTACCTGATCTCAGGCAT | Ultra 341 (-): 3115304- 3115376 |  |
| chicken | SNORD44 | TGCCTGTGTGATGAGACATGAATACACTGACTTCAATCATGGAGGTCTGCAATTAGCTCTATCTGACTTCAGGCA | chr.8(+): 7732322- 7732396 |  |
| lizard | SNORD44 | AAATGCAAGCAGGTAATGTGATGATAAGCCATTAGAACTGACTTAAACAATGGAGGTTTTAATTAGCTCTACCTGATTATAAAGATATGCTTGCATTT | scaff.169 (+): 2598467- 2598564 |  |
| frog | SNORD44 | AACCTCTTAATATGATGAAATAAACAGGCTTGACATTTCCATGGAGGTCTGTAATTAGCTCTATCTGATGCTGGGGTT | scaff.1(+): 6873505- 6873582 |  |
| zebrafish | SNORD44 | GACTAGGGTGAGGAATCTGATGTCCACTGACTTTAGCCATGGAGGGATGGATATTAGCTCTGTCTGATCTGGTC | chr.8(-): 13994881- 13994953 | A complementary substitution in the antisense element (shown in pink). |
| tetraodon | SNORD44 | GACCAGTGTGATGACAAATGTAAAGGCTGACACCTAAACGTGGAGTCGTTATTAGCTCTAACTGACTTGGTC | chr.1(-): 11036155- 11036226 |  |
| fugu | SNORD44 | ACCGACCTGTGTGATGACAAATGTACAGCCTGACACTTGAACGTGGAGTCGTTATTAGCTCTAACTGACCTGGTCGGT | Un. (-): 193906984- 193907061 |  |
| human | SNORD45A | AAGGTCAATGATGTGTTGGCATGTATTATCTGAATCTATTGCTGATGTGTAATAACACTTTAGCTCTAGAATTACTCTGAGACCT | chr.1(+): 76026160- 76026244 | The nucleotides complementary to the SNORD45 RNA targets are shown in red. Human and mouse SNORD45C and platypus SNORD45A RNAs can guide the modification of a neighboring site in RNA. |
| human | SNORD45B | TGTCCTACAAGGTCAATGATGTAATGGCATGTATTAGCTGAATCTAAAGTTGATGTGAGTTCTAAAATTACACTGAGACCTTGGAGGGTA | chr.1 (+): 76027740- 76027829 | The second antisense element has nucleotide substitutions. |
| human | SNORD45C | TTTTTGGGTCAATGATGAGTTGGCATGTATTCTGAATCTAAAGTTGATTATTACTACTTTAGCTCTAGAATTACTCTGAGACCTGAAAA | chr.1(+): 76025339- 76025427 |  |
| mouse | SNORD45A | GGGTAAAACCTTGAAGGTCAATGATGTGTTGGCATGTATTATCTGAATTCGCTGATGTGTCATAACACTTTAGCTCTAGAATTACGCTGAGACCTTGAAAAGGTTTTACCT | chr.3(-): 153574559- 153574669 |  |
| mouse | SNORD45B | CTTCCCACAGGTCAGTGATGTCATGGCATGTATTAGCTGAATGCTAACCTGATGCAAGTTACAAATTACACTGAGACCTTGGAAG | chr.3 (-): 153573522- 153573606 | The second antisense element is disrupted. |
| mouse | SNORD45C | TTCGGGTCAATGATGAGCTGGCATGTATTCTGAATCTAAAGTTGATTATAAACCACTTTAGCTCTAGAATTACTCTGAGACCTGA | chr.3(-): 153575088- 153575172 |  |
| opossum | SNORD45A | GGGGCCGAGCCTGCAGGTCAATGATGTCTTGGCATGTATTAGCTGAGCCGCCGCTGATGCTTCCTACCCGGAAAGCTCTAGAATTACTCTGAGACCGTGCGGAGCCCC | chr.2 (+): 1474091- 1474198 |  |
| opossum | SNORD45B | TCCAGGTCAGTGATGTGATGGCATGTATTACCTGAATCCAAAGTTGATGGATATCACACTTTAGCTCTAGAATTACACTGAGACCTTGGA | chr.2 (+): 1475231- 1475320 |  |
| platypus | SNORD45A | CAGAGTTTTCTCTGTCAATGATGAGTTGGCATGTATTCTGAATCTAAAGTTGATGTTTATCACCATTTAGCTCTAGAATTACTCTGAGGCTTGAGAAAACTTTG | Contig 5910 (-): 23921- 24024 |  |
| platypus | SNORD45B | TTTCAGGGTCAATGATGTTTTGGCATGTATTACCTGAATCTAAAGTTGATGCTAATTACCTCTTCAGCTCTAGAATTACTCTGAGACCCTGAAA | Contig 5910 (-): 23420- 23513 |  |
| platypus | SNORD45C | CCAAGGTCAATGATGGTTGGCATGTATTATCTGAATCTAAAGTTGATGTTTATCACCACATTAGCTCTAGAATTACTCTGAGACCTTG | Contig5910 (-): 21625- 21712 |  |
| platypus | SNORD45D | TTCAAGGTCAGTGATGTGTTGGCATGTATTATCTGAATCTAAAGTTGATGCTTACCATTCCTATAGCTCTAGGATTACTGAGACCTTGAA | Contig 5910 (-): 19700- 19789 |  |
| chicken | SNORD45A | TTGATTTCTTTAGGTCTGTGATGAGTTTTGGCATGTATTATCTGAGCATAAGTGTGAAGATTATCAGTTTAGCTCTAGAATTATCTGAGACCTTAGAAATAA | chr.8(+): 30596298 30596400 |  |
| chicken | SNORD45B | TTGGGTCAGTGATGATTGGTGGCATGTATTATCTGATGTAAAGGTGATGTTTATCATCACATTAGCTCTAGAATTACTCTGAGACCCAGG | chr.8(+): 30597833- 30597922 |  |
| lizard | SNORD45A | AGGTCAATGATGTAACACTGGCAtgtattatctgagtccaaagCTGAAGATTTAGCTCTTTAGCTCTAGAATTACACTGAGACCT | scaff.195 (-): 393791- 393875 |  |
| lizard | SNORD45B | CCTTGGGTCAATGATGAATTCTGCATTTTTTATCTGAAACTATAATTGATGTAGTTTTAATCCTTAGCTCTAGAATTACTCTGAGACCTGGGG | scaff.195 (-):  392286- 392378 |  |
| lizard | SNORD45C | TTCTCAGATCTATGATGAGCTGTTAATATTTATCTGACTAAGGTTGATGTTACATCACAATTTTAGCTCTAGAATTACTCTGAGATTTGAGAG | scaff.195 (-):  391577- 391669 |  |
| mouse | SNORD46 | TGGAGGTAACTAGGGTGATGAAAACGTATCCTTAGGCGTGGTTATGGCCGTCTTGGCCACCTGTACACCACTTGCCAATGCAAGGACTTGTCATAGTTACACTGACTGTTACCTCCG | chr.4 (-): 116827785- 116827901 |  |
| rat | SNORD46 | GGAGGTAACTAGGGTGATGAAAAAGTATCCTTAGGCGTGGTTCTGGCCGTCTTGGTCACCTGTATGCCACTTGCCAATGCAAGGACTTGTCATAGTTACACTGACTGTTACCTCCG | chr.5(-): 137468352- 137468467 |  |
| dog | SNORD46 | CTCTTGGAGGTAACCGGAGTGATGAGAAAGAATCCTTAGGTGTGGTTGGGGCCGTCTTGGCCACCTGTGTGCCACATGCCAGTGCTAGGACTTGTCATAGTTACACTGACTGTGTTGCCTCCACGGGG | chr.15(-): 18549640- 18549767 |  |
| opossum | SNORD46 | CACGTGAGTGATGATCGCCGGAGCCTTAGGGGTCCGAGCCGGGCCGTGCTGGTCCGAGGGCCGCCGATGCTGGGGCTACTCTGTCATAGTTACACTGACACGTG | chr.2(-): 9662024- 9662127 |  |
| chicken | SNORD46A | GAGGCAATGGGAGTGATGAAATTTTCACCTTAGGTAGATACGACAGACTGTTCTGGTCGTTAACTGTATCTGCCAATGCTGGGGTTTGTCATAGTTACACTGACCACGTTGCCTT | chr.8(+): 21480970- 21481084 |  |
| chicken | SNORD46B | AGTACGCACGGTGATGATAACTGTGACCTTATGTGGCCTTGGGAGGCTTGGATGTCTGCTGGGTTCACAATGATTGGGGTGTCATAGTTACACTGAAGGGCGTGCT | chr.8(+): 21481377- 21481482 |  |
| lizard | SNORD46A | GGCACCTGAAGTGATGAGACTGACACCTTAGGTGGGTGCTGTAGACTGTTCTGGTCTTCATCACACTCACCAGTGCTAGGAGTTGTCATAGTTACACTGATCAGGTTGCC | scaff.4(-): 6845600- 6845709 |  |
| lizard | SNORD46B | CAAAACAACTGTGATGATAACTTTGACCTTAGGAGAGCTGTTGGTGAATTCCTCAGCTTTCAGTGATGGGAGTGTCATAGTTACCCTGAGTTTTG | scaff.4(-): 6845286- 6845380 |  |
| frog | SNORD46A | GGCAATCTGTGATGAAAAGCTGTCCTTAGGCATGCAGTTCAGGCTGTTTTGGCCCTTGTTTTTGCTGCCTGCCAGTGATAGGCCTGTCATAGTTACACTGATGGATTGCC | scaff.261 (-): 1542383- 1542492 |  |
| frog | SNORD46B | GCAGTGCTAATGATGACAACTTTGGCCTTATGGGGGTTTTCTATGATCTTTTTGATCTTGAATCTCTCTGTGCTAGGAAAAGTCATAGTTACTCTGACTGCGCTGC | scaff.261 (-): 1541291- 1541396 |  |
| zebrafish | SNORD46 | ATGTGGGTGATGTTAACATGTCCTTAGGTTGGTCTTATTTGGCCGTCTTGGTCAAAGTCTTTGACCTTCCAATGTTAGGATTCGTCATAGTTACACTGACACAT | chr.2(+): 22149873- 22149976 |  |
| fugu | SNORD46 | TGCTGTGGGTGATGAACGTGTGACCTTAGGTTTGTCTTTTTTTGGACCGTTTTGGTCCAAAACCTTGGGCTTACCTATGAAAGGACTTGTCATAGTTACTCTGACACTGTA | Un. (-): 152045832- 152045942 |  |
| medaka | SNORD46 | GCGTGGATGATGAAAACTGGACCTTAGGTAGGCCTTTTTTGGGCTGTTTTGGCCTAAAGCTTTGGGCTTACCAATGGTAGGGCTTGTCATAGTTACACTGACACGC | chr.17(+): 3706849- 3706954 |  |
| mouse | SNORD47 | GTAACCAGTGATGTGATGATTCTGCCAAATGATACAAAGTGATATCACCTTTAAACCGTTCCATTTTATTTCTGAGGTTAC | chr.1(+): 162968211- 162968291 |  |
| rat | SNORD47 | GTAACCAGTGATGTGATGATTCTGCCAAATGATACAAAGTGATATCACCTTTACAACCGTTCCATTTTATTTCTGAGGTTAC | chr.13(+): 76597644- 76597725 |  |
| dog | SNORD47 | GTAACCAGTGATGTAGTGATTCTGCCAAATGAAATAGAATGATATCACTCATAAAACCGTTCCATTTTGATTCTGAGGTTAC | chr.7(+): 28332270- 28332351 |  |
| opossum | SNORD47A | TTTAATTGTAACCAGTGATGTAAAAGATTCTGCCAAATGAGAGGACATGATTTTACTCCTAAACCGTTCCATATTTTATACTGAGGTTACATTTGAA | chr.2(-): 66317213- 66317309 |  |
| opossum | SNORD47B | TAACCAGTGATGTTAATGATTCTGCCAAATGAAATGGCAATGATTTCACCCATATACCGTTCCACTTTTATTCTGAGGTTA | chr.2(-): 66316912- 66316992 |  |
| platypus | SNORD47 | TAACCAATGATGATAAGATTCTGCCAAATGAAAGAGACTGATTTCACCTATAAAACGTTCCTTTTTTTATTCTGAGGTTA | Ultra 341 (-): 3113139- 3113218;  Contig 37762 (+): 1075-1154 | Very short contig (contig 37762)contains a fragment of the SNORD47 RNA host gene (gas5) which includes the SNORD47 sequence. Until the final assembly of the platypus genome become available it remains unclear wether SNORD47 RNA has one or two genes. |
| chicken | SNORD47 | TTGGTAGCCACTGATGAAACTTGATTCTGCCAGATGAGTTTCTGTGATATTACCATTTCCGTTCCATTCTTTCTGAGGTTACACAG | chr.8(+): 7734784- 7734869 |  |
| lizard | SNORD47 | TGTAACCAGTGATGAAATTGAATTCCGCCATATGAATCCTTGTGAAATTAACACAATTCCGTTCCATTTACTGAGGTTACA | scaff.169 (+): 2599626- 2599706 |  |
| frog | SNORD47A | GTTGGTGACCTGTGATGAAAATGATTCTGCCATATGATTAGGATGATATTATCCTTAACCGTTCCATTTCTGAGGTAGCCAGC | scaff.1(+): 6874828- 6874909 |  |
| frog | SNORD47B | TGTAACCAATGATGAAAATGATTCTGCCATATGAAACACTGAAACACTGATTACACTTATTTTACCGTTCCACATTTTCTGAGGTTATA | scaff.1(+): 6877560- 6877648 |  |
| zebrafish | SNORD47A | TAACCTGTGATGAAATGATTCTGCCAAATGAATCCTAATGATTATACCCCAACCGTTCCATATTTCTGAGGTTA | chr.8(-): 13994451- 13994524 |  |
| zebrafish | SNORD47B | TAACCTGTGACGACATGATTCTGCCAAATGAATCTAATGATTATACCCCAACCGTTCCATATTTCTGAGGTTA | chr.8(-): 13994193- 13994265 |  |
| tetraodon | SNORD47 | CCACTCTTCCTGTGATGATCAGATTCTGCCAGATGATTTCTGATTATAATAAATACCGTTCCATTATTTCTGAGGTTGAGTGG | chr.1(-): 11035895- 11035977 |  |
| fugu | SNORD47 | GACCACTCTTCCTGTGATGAACATATTCTGCCAGATGACTTCTGATTATAACTCAATACCGTTCCATTATTTCTGAGGTTGAGTGGTC | Un. (-): 193906720- 193906807 |  |
| mouse | SNORD48 | TTCGGGGAGAGGACAGTAATGATGATTTCTGGTGATTCTGAGTGTCTCGCTGACGCCATCACCGCAGCGCGCTGACCGTCCCCGGG | chr.17(-): 35088949- 35089034 |  |
| opossum | SNORD48 | CTTGATAGGCATGATGACCTCAGTGGATCTGAGTATCTCGTTGATGCTACCACCGCAGCGCGCTGACTTTTCCTGGTTGGG | chr.2(+): 258063852- 258063932 |  |
| mouse | SNORD49A | GCTTGACTGCTGTGATGAGATGACTAAGTAGGAAGTGCCGTCAGAGTCGATAACTGACGATAACTGCTCCTGGCTGACTGCAGTCAAGC | chr.11(+): 62416945- 62417033 |  |
| mouse | SNORD49B | CGGCTGCAATGATGATGAAACTAGAAAAAAAAGGAAGTGCCGTCCGATGCGACAACTGACGACATCCCTAGTTAGCTGACTGCAGTCG | chr.11 (+): 62416572- 62416659 |  |
| opossum | SNORD49A | GGGCTGCTGTGGCGTGATGAGAACCCAGAATAGGAAGTGCCGTCAGACCGACAACTGACGATAGCCTGAGCTGGTGTCTGACCCGCAGCCCGccc | chr.2 (+): 520354168- 520354262 |  |
| opossum | SNORD49B | TGGCTGTTCATGATGATACCACTCAATAGGAAGTGCCGTCAGATGCGATAACTGACGATGAACTTGATCATGTCTGACAGCAGCCG | chr.2 (+): 520355669- 520355754 |  |
| platypus | SNORD49A | GCCCAGATGATGAGAACCGTGGATAGGAAGTGCCGTCAGATGCGAGAACTGACGATGGATTTTCACGGGGTCTGACTGGGC | Ultra 42 (+): 1071101- 1071181 |  |
| platypus | SNORD49B | ACTCGGCTGCAATGATGATAACATGGAATATAGGAAGTGCCGTCAGATGCGATAACTGACGATAATCTCTGTACCTAATCTGACCGCAGCTAGT | Ultra 42 (+): 1071848- 1071941 |  |
| chicken | SNORD49 | GCTGGCTGTTGATGATGATACCCCAGAATAGGAAGTGCCGTCAGATGCGAGAACTGACGATAACCTTCTTATTTATCTGAAATACAGC | chr.19 (-): 5110851- 5110938 |  |
| lizard | SNORD49 | CCGCGCGCGGGGTGATGATGCCGAGATAGGAAGTGCCGTCAGATGCGAGAACTGACGAGACGCTCATCTCCGTCTGACCGCCCGG | scaff.2527 (+): 5198- 5282 |  |
| frog | SNORD49A | AGTGCCATGAGCATGATGAAACCAGGATATAGGAAGTGCCGTCAGATGCGATAACTGACGAGAACCTTTTCCTGTTCTGACTTTAATCTGCT | scaff.178 (-): 1751170- 1751261 |  |
| frog | SNORD49B | GTAAACATGATGAAATCTGGATATAGGAAGTGCCGTCAGATGCGATAACTGACGAGAACCTTTTCCTGTTCTGACTTTGT | scaff.178 (-): 1749570- 1749649 |  |
| zebrafish | SNORD49A | GAGTGTGTTCTGTGATGAAAACGCTATATAGGAAGTGCCGTCTGATGCGATAACTGACGATGGCAAAGCAAAATCTTTCTGAGACGCAC | chr.3(+): 62751858- 62751946 |  |
| zebrafish | SNORD49B | TGTGTTCTGTGATGAAAACGCTATATAGGAAGTGCCGTCTGATGCGATAACTGACGATGGCAAAGCAAAATCTTTCTGAGACGCACA | chr.3 (+): 62753681- 62753767 |  |
| fugu | SNORD49A | CTATTGCCAATGATGAAAACTGAATGGGAAGTGCCGTCTGATGCGATAACTGACGATGTTTAATACTTAACTTATCTGAGGCTTGG | Un. (+): 268305955- 268306040 |  |
| fugu | SNORD49B | AAGCCTTGTCAATGATGAAATCCTGGAATAGGAAGTGCCGTCAGATGCGACAACTGACGATTTCTCATCCACTTTTCTGAGGCTT | Un. (+): 268306609 268306693 |  |
| fugu | SNORD49C | GGTTTCAGTGATGAGAAGTGCTGCATAGGAAGTGCCGTCTGACACGATAACTGACGATGGCACAAGCTGCTCTGAGAAACC | Un. (+): 185326987- 185327067 |  |
| mouse | SNORD50A | TGGTCTAGTCTATGATGATCC_TATCCCGAACCTGAATTCCTGTTGAAAAACCATAATTACGGATCTGGCTTCTGAGATGGACCA | chr.9(-): 88416629- 88416712 | U50HG is the host gene. |
| mouse | SNORD50B | TGGTCTAGTCTATGATGATCC_TATCCCGAACCTGAATTCCTGTTGAAAAACCATACTTACGGATCTGGCTTCTGAGATGGACCA | chr.9 (-): 88416361- 88416444 |
| mouse | SNORD50C | TGGTCTAGTCTATGATGATCC_TATCCCGAACCTGAATTCCTGTTGAAAAACCATAATTACGGATCTGGCTTCTGAGATGGACCA | chr.9(-): 88752525- 88752608 | uc009qzd.1 (UCSC ID) is the host gene. |
| mouse | SNORD50D | TGGTCTAGTCTATGATGATCC_TATCCCGAACCTGAATTCCTGTTGAAAGACCATACTTACGGATCTGGCTTCTGAGATGGACCA | chr.9(-): 88493180- 88493263 | uc009qyw.1 (UCSC ID) is the host gene. |
| mouse | SNORD50E | TGGTCTAGTCTATGATGATCC_TATCCCGAACCTGAATTCCTGTTGAAAAACCATACTTACGGATCTGGCTTCTGAGATGGACCA | chr.9(+): 88970168- 88970251 | The host gene is unannotated (representative EST [**CJ162032**](http://www.ncbi.nlm.nih.gov/entrez/query.fcgi?cmd=Search&db=nucest&term=CJ162032&doptcmdl=GenBank&tool=genome.ucsc.edu)**).** |
| rat | SNORD50A | CGGTCTGATCTATGATGATCCTATCCCGAATCTGAATTCCTGTTGAAAAAATATAATTACGGATCTGGCTTCTGAGATGGACCG | chr.8(-): 93792102- 93792185 | U50HG is the host gene.  The first antisense element in chicken SNORD50A gene is disrupted. |
| rat | SNORD50B | CGGTCCGATCTATGATGATCCTATCCCGAATCTGAATTCCTGTTGAAAAACTGTAGTTACAGATCTGGCTTCTGAGATGGACCG | chr.8(-): 93900303- 93900386 |
| dog | SNORD50A | TGGTCTTAATCTATGATGATCTTATCCCGAACCTGAATACATGTTGAGAAAACCAATACGGATCTGGCTTCTGAGATAGACCA | chr.12 (-): 48681845- 48681927 |
| dog | SNORD50B | ATTCTGGTCTAATCAATGATGAAACCTATCCCGAAGCTGACCACCTGAAGAAAAGCATATACGGATTCGGCTTCTGAGATGAGACCAGAAT | chr.12 (-): 48682167- 48682257 |
| opossum | SNORD50A | TTTGGTCTTATTAATGATGATTCTATCCCGAACCTGAATTCCTGTTGAAAAACTACATATACGGATCTGGCTTCTGAGATAAGACCAAA | chr.2 (-): 342122618- 342122706 |
| opossum | SNORD50B | TGGTCTACTCAATGATGAACCTATCCCGAACCTGATTTCATGATGAAAAAACTTAAATACGGATCTGGCTTCTGAGAAGACCA | chr.2 (-): 342122998- 342123080 |
| platypus | SNORD50A | CTTGGTCTTGTCTGTGATGATTCTATCCCGAACCTGAACTCCTGTTGAAAAAAAACACAAACGGATCTGGCTTCTGAGATAAGACCAAG | Ultra.42 (+): 85176- 85264 |
| platypus | SNORD50B | AAGGAGGTGGTACAATCAGTGATGAGCATATCCCGAACCTGACTTCTTGATGAAAACACTATTATACGGATCTGGCATCTGAGAAACCACTCCTT | Ultra.42 (+): 84853- 84947 |
| chicken | SNORD50A | AAGTGGTGGATTGTGATGAAGTTGCCATACCTGATTTGTTGATGAAAAACTTCATACGGATCTGGCTTCTGAGATAAAACCACTT | chr.3 (+): 79582945- 79583029 |
| chicken | SNORD50B | TCTGCATCTCATCTATGATGATCCTATCCCGAACCTGAATTCTTTGCTGAAAAACCTACTACGGATCCGGCATCTGAGATGAGATGAGA | chr.3 (+): 79585086- 79585174 |
| lizard | SNORD50 | TGATATAATCATTGATGAGACTATCCCGAACCTGATTTCTTTGATGAAAAAACTGCCAATATGGATCCGGCTTCTGAGGTTATATCA | scaff.205 (+): 2324832- 2324918 |
| frog | SNORD50A | TTGAACTGTGATGAGTCTATCCCGAACCTGAGTTGTGATGACACTTTCTTATGGATCTGACTGCTGAGTTTGA | scaff.447 (-): 771829- 771901 | A complementary substitution in the antisense element (shown in pink).  U50HG is the host gene for frog SNORD50 RNAs.  RPL13A is the host gene for fish SNORD50 RNAs.  The first antisense element in fugu SNORD50B gene is disrupted. |
| frog | SNORD50B | GGGAGGCTTTTCAAGCAGTGATGAGTCTATCCCGAACCTGAGTCTTTGATGACACACATATGGATCTGACTGCTGAGCTGTTACAAAGCCTCCC | scaff.447 (-): 771566- 771659 |
| frog | SNORD50C | GTTAACTATGATGAGTCTATCCCGAACCTGATTTGTGATGACACTTTCTTATGGATCTGACTGCTGAGTTTAAC | scaff.447 (-): 767881- 767954 |
| frog | SNORD50D | TTTGCAAGCTGTGATGAGTCTATCCCGAACCTGAGTCTTTGATGACACAATTATGGATCTGACTGCTGAGCTTATTAAA | scaff.447 (-): 767239- 767317 |
| zebrafish | SNORD50A | TTCAAACAATGATGAACTCCTTTATCCCGAATCTGAGCCAAGTTTGATGATACCAATCTACGGATCTGACTACTGAGTTTGAG | chr.17(-): 2087885- 2087967 |
| zebrafish | SNORD50B | TTCAAACAGTGATGAACTCCTTTATCCCGAATCTGAGTCCAGTTTGCTGATACCCATCTACGGATCTGACTACTGAGTTTGAA | chr.17(-): 2087586- 2087668 |
| fugu | SNORD50A | TTGTTCCTGTTCTTACTGTGATGAGCACTTTATCTCAAAACTGATCCTCTGTGATGAGACGCAAGATACGGATCTGATTACTGAGTTCAGCAGGAACAA | Un. (+): 36446991- 36447089 |
| fugu | SNORD50B | CAACTTGACAATGAAGATCAATGTAACCCTTAACTGATCATTTATTGATGAGTCCTACTTTTCGGATCTGATTGCTGAGTCGAGTTG | Un. (+): 36447445- 36447531 |
| mouse | SNORD51 | tcactgGGTACATGATGAATAAAATCAAATCACCTTCTTTCGGCTGAGCTCCTGATGGATTTGCTTTTTCTGATAaatctggtgg | chr.1(+): 63225248- 63225332 |  |
| opossum | SNORD51 | AAAACTTGCTGGCTACATGATGAATAAAATCTAATCACCATCTTTCGGCTGAGTTCGTGATGGAATTGCTTTTTTCTGATTGGCTGGTCTGTTTT | chr.7 (-): 191106982- 191107076 |  |
| platypus | SNORD51 | TGCTAAAACACGGCTGGCTACATGATGAATCAAATCAAATCACCATCTTTCGGCTGAGATCGTGATGGATCTGCTTTATTCTGACCAAGCTGGCTTATTTGCA | Ultra 170 (-): 93699- 93801 |  |
| chicken | SNORD51 | TTGACTGGTTACATGATGAATAAAACAAAAATCACCATCTTTCGGCTGACAGCTGTGATGATTTGTTTCTTTCTGAGTAGATGCCAGTCAA | chr.7 (-): 13642716- 13642806 |  |
| lizard | SNORD51 | TCATTGGTGACATGATGAACAAAACCAAATCACCATCTTTCGGCTGAAATTGTGAtgatttgtttttttctgaatga | scaff.1298 (-): 62663-62739 |  |
| frog | SNORD51A | CAGGCTGGTCATGTGATGAATGAGCTAAATCACCATCTTTCGGCTGATTTGTGATGCATATTGTCTTCTAAACTGACTACACCAGCCTG | scaff.208 (+): 301529- 301617 |  |
| frog | SNORD51B | AACTGTAATGATGATTAAAATATCACCATCTTTCGACTGATGCTAATGATGGATGTATTTTTATTCTGACTGCCTT | scaff.208 (+): 302534- 302609 |  |
| zebrafish | SNORD51A | TTCCCCTAACACGTGATGATTATCCTAACACCATCTTTCGCCTGAGTGACTGATGCACAATTCTTTAATCTCTGATTGGTGTGGGAA | chr.6 (-): 12296341- 12296427 |
| zebrafish | SNORD51B | CTGGTCATGTGATGATTTATCTCACCATCTTTCGACTGATGCCGTGATGGATCTCTTTTATTCCTGAATACCAG | chr.6(-): 12296032- 12296105 |  |
| zebrafish | SNORD51C | GTGACTAGTTCAGTGATGATTTCTTATAACCATCTTTCGGCTGAATATGGTGATCGACACACTCTTATCTGATGTATTACTAGTCAT | chr.6 (-): 12292850- 12292936 |
| tetraodon | SNORD51A | GGGCTGACTTCCAATGATGATTTATTTTCACCATCTTTCGGCTGACTGCCTGATGGACACTTGTTTATCTGAGAGAGTATCAGCTC | chr.3 (-): 17711-17796 |  |
| tetraodon | SNORD51B | AAAATGATGCATGTTGTGCAGTAGTGATGATCTAACTCACCATCTTTCGGCTGAACTTAAGATGATACTCATAAATCTGAAATGTTTCAAGGTTTT | chr.3 (-): 17412-17507 |  |
| human | SNORD52B | GGAGTGGTAATGATGATCTGGTTGGACAAGAGTCTCTGAGCTTTTCTCTGAGGATCTTTGAACCCACCTGATCCACCTTC | chr.6(+): 31913221- 31913300;  chr.6_cox_hap1(+): 3251323- 3251402; chr.6_qbl_hap2(+): 3052845- 3052924 |  |
| mouse | SNORD52A | GGCCGGGTATGATGACCCACATTTACAGACTAGAGTTTCTGATGTCCACGTGACGCCACTCCTTGTAACTGACCCAGGCC | chr.17(-): 35089179 35089258 |  |
| mouse | SNORD52B | CCTCAATTGAGAGTGATGATTTCACAGACTAGAGTCTCTGACGCTGTCCTTGATGTCAGCTATAAATCTGACTCATTTGGGG | chr.17 (-): 35087889- 35087970 |  |
| opossum | SNORD52A | CTGGACAGGATGATCCTACTTTTACAGACTAGAGTTTCTGATATCTGTGACGTCACCATTTGGAACTGATCCAG | chr.2(+): 258063623- 258063696 |  |
| opossum | SNORD52B | AGGTGGGAATGATGAGTTTTTCAGACTAGAGTCTCTGACATCTGCCTTGGAGACAAGAAAACCACTCTGACCCATCCT | chr.2(+): 258064854- 258064931 |  |
| platypus | SNORD52 | CAGAGTGGGGGTGATGAGTCCTTCAGACTAGAGTCTCTGACCACCTTTGCTGAAGACACAGATCTCTGACCCATTCTG | Contig 5910 (-): 16014- 16091 |  |
| mouse | SNORD53A | GCCAACTCATGCTGTGATGATATCCTCATGGTTTCGCGTCTGTCTGAGTCTCAGAGATGACACCTTTCTCTTGGCTGTTTGAGCATGGTTGGC | chr.17 (+): 71990211- 71990303 | A substitution in box D. |
| mouse | SNORD53B | AGTCAGGGCTTGCTGTGATGACTGTCATTGGGTTTCGCATATTGCTGAGTTCCCATGATGCCTCTTCTCTTGGCTGTCTGAGCAGCCCTGGCT | chr.17 (+): 71990915- 71991007 |  |
| opossum | SNORD53A | GCTGGGAGCTGCTGTGATGACTTCCATTTGGTTTCGCTTGTGACTGAGTTTCAGAGCTGATGCTTCTCTCTTGGCTGTCTGAGTGGTCCTGGC | chr.1 (+): 510847854- 510847946 |  |
| opossum | SNORD53B | AATGCCAGTACTCGCTGTGATGATTTCATTCTGGTTTCGCTTTTTGCTGAATTCGTGATGCCTTTCTCTTGGCTGTCTGAGCATGTCTGGCATT | chr.1 (+): 510848647- 510848740 |  |
| platypus | SNORD53A | AGCCAGGACATGCCGTGATGATTACCATCTGGTTTCGCTTTTGGCTGAGTTTCCGTGGTGACACCTTTCTCTTGGCTGTCTGAGCTTGCCTGGCT | Ultra 202 (+): 1029223- 1029317 |  |
| platypus | SNORD53B | CCAAACTAGGCATACAGTGATGATCTACTTCTGGTTTCGCTTTTTGCTGAGATCCAGTGATGTTGCCTTTCTCTTGGCTGTCTGAGTGTGCCTAGTGTGG | Ultra 202 (+): 1031751- 1031850 |  |
| platypus | SNORD53С | ACTAGGCATACAGTGATGATCTACTTCTGGTTTCGCTTTTTGCTGAGATCCAGTGATGTTGCCTTTCTCTTGGCTGTCTGAGTGTGCCTAGT | Ultra 202 (+): 1032813- 1032904 |  |
| chicken | SNORD53A | GCCTAGCTGTGATGATACCCAATTGGTTTCGCTCACTACTGATGTGCAGTGGGGACAATTCCACTTGGCTGTCTGAGCAGGCT | chr.3 (-): 8535483- 8535565 |  |
| chicken | SNORD53B | GGCATGCAGTGATGATCTCCATCTTGGTTTCGCTTTTTGCTGAGTTCCAGTGAAGACTCATTTCTCTTGGCTGTCTGAGCATGCC | chr.3(-): 8526951- 8527035 |  |
| lizard | SNORD53A | CTGCTGTGATGATGTCCACGTGGTTTCGCTCACTTCTGATATGTGGTGAGGACAGTTTCCTCTTGGCTGTCTGAGCAG | scaff. 221 (-): 1024792- 1024869 |  |
| lizard | SNORD53B | GGGTATGCTATGATGACCTTCGTCTTGGTTTCGCTTATTGCTGAATTCCATTGATGACGACACATTCTCTTGGCTGTCTGAGCATGCCT | scaff. 221 (-):  1016249- 1016337 |  |
| lizard | SNORD53С | CCTCGGGTATGCTATGATGACCTTCATTTTGGTTTCGCTTATTGCTGAATTCCATTGATGATGACACATTCTCTTGGCTGTCTGAGTGTGCCTAGCAGG | scaff. 221 (-):  998098- 998196 |  |
| frog | SNORD53A | CCCGCTCTGATGACTCTCTCATTGGTTTCGCTTGTTTCTGAGAAGCAATGACGCCACCGTGTTCCTCTTGGCTGTCTGAGCGAGCTGGG | scaff.372 (-): 391012- 391100 |  |
| frog | SNORD53B | GGGTCGTGCTGTGATGATGATCAGAGAATGGTTTCGCACACTTCTGAGACCTAGTGATGTGCCAATCTCTTGGCTGTCTGAGCATGCCC | scaff.372 (-): 386931- 387019 |  |
| frog | SNORD53C | TGGTCCTGCAGTGATGAGTTGCCTCTGGTTTCGCTTGTGTCCGACAAGCTGTGACGACGCATCTCTCTTGGCTGTCTGAGCAGAACCA | scaff.372 (-): 386007- 386094 |  |
| zebrafish | SNORD53A | GGAGGAAATGCACATGATGATACTGGAGAATGGTTTCGCTTTTATGACCTGATGTCATGTGATGATACTCTTCTCTTGGCTGTCTGATCTACCTCT | chr.17(-): 8955073- 8955168 |  |
| zebrafish | SNORD53B | CAGCAGAGTCAAAAATGATGATACCAGAGAATGGTTTCGCTTGCATGACTGATTTCATGTGATGTCACGCTTCTCTTGGCTGTCTGATTAATCTCTG | chr.17 (-): 8952565- 8952661 |  |
| zebrafish | SNORD53C | GGGGTCAAACATGATGATACCAGAGAATGGTTTCGCTAACTTGACTGATGTCATGTGATGTTACACTTCTCTTGGCTGTCTGATTTAGCTCC | chr.17(-): 8949515- 8949606 |  |
| zebrafish | SNORD53D | GAGGGTCAAAAATGATGATACCAGAGAATGGTTTCGCAAATTTGACTGATGTCATGTGATGTTATGCTTCTCTTGGCTATCTGATTAACCTCTG | chr.17 (-): 8944707- 8944800 |  |
| zebrafish | SNORD53E | CAAGCGAAAAGAGGGACAAAAGTGATGATACCAGAGAATGGTTTCGCAAATTTGACTGATGTCATGTGATGTTATGCTTCTCTTGGCTATCTGATTAACCTCTGCTTG | chr.17(-): 8941018- 8941125 |  |
| tetraodon | SNORD53A | CTCAGGACGGGATGATGACCCACAGTGGTTTCGCTCATTGGCCTGAGCCCCAGTGATGTCACTTCCTCTTGGCTGTCTGACCTGTTCCTGAG | Un. random (+): 83016749- 83016840 |  |
| tetraodon | SNORD53B | GGGTGATGATGCTGAGAACGGTTTCGCTAAGTGAACTGAGAACGTGTGATGTCACACTCTCTCTTGGCTGTCTGACCAATCACCT | Un. random (-): 83029261- 83029345 |  |
| mouse | SNORD54 | GTCGGCGATGAGGAGATACCAACCGTATTGAGTTATAGTGATATTTCTTATACGCTATTCTGAGCCGAT | chr.4(-): 3762227- 3762295 |  |
| opossum | SNORD54 | AAGTTGGCAATGATGATGAAATTTAAGCAGTTGAATTTCGGTGATTGTCTCATACGCTATTCTGAGCCAGCTT | chr. 3 (+): 184035111- 184035183 |  |
| platypus | SNORD54A | CGTTGGCTATGAAGATAAAATCATGCAGATTGAATTTCCGTGATTGTTTTTAT_CGCTATTCTGAGCCCACG | chr.7 (+): 6583823- 6583893 |  |
| platypus | SNORD54B | GTTGGCTATGAAGATAAAATCATGCAGATTGAAGTTCCGTGATTGTTTTATACGCTATTCTGAGCCAAC | chr.7 (+): 6590951- 6591019 |  |
| chicken | SNORD54 | GTTGGCTATGAGGATAAACTCTTAAACCTGAATTGCAGTGATCATTTTATACGCTATTCTGAGCCAAC | chr.2 (-): 114877288- 114877355 |  |
| lizard | SNORD54 | GTTGGCAATGAGGACAAAAAGTGTTAACTGAACTACCATGATTAGTGAATACGCTATTCTGAGCCAAC | scaff.235 (-): 2094264- 2094331 |  |
| frog | SNORD54 | GAGGTTGGCAATGAGGATACATTTTCTCATGAATCAAAGTGATTGGTTTATACGCTATTCTGAGCTTACTCATACCTC | scaff.83 (-): 2701352- 2701429 |  |
| zebrafish | SNORD54 | GGCTATGAGGATAAAATTGTATGAACGACTTAAACATGAGTTATATCATACGCTATTCTGAGCC | chr.7(+): 57739435- 57739498 |  |
| tetraodon | SNORD54 | GAACACCTGAGCTATGAGGACAAACATTTGAACAGATGCACTCTGGTGACTGTATACGCTATTCTGAGCCACAGCTTC | Un_random (+): 134241681- 134241758 |  |
| mouse | SNORD55 | CCGCACGCGGATGATGACACCTGGGTATGCTGCACACTCCCGACTGCGTCGTGGGGAAGCCAACCTTGGAGAGCTGAGCGTGCGG | chr.4(-): 116828374- 116828458 |  |
| rat | SNORD55 | CGCGGGCCGCACGCGGATGATGACACCTGGGTATGCTGCACATTCCCGACTGCGTCGTGGGGAAGCCAACCTTGGAGAGCTGAGCGTGCGGCCCGCG | chr.5(-): 137468950- 137469046 |  |
| dog | SNORD55 | GCTGGGCCGCGCGTGGATGATGACACCTTCGTAATGCGGAAAACTCCCGAGTGCGCAGTGGGGGAGCCAACCTTGGAGAGCTGAGCGTGCGGCGCGCCCGGT | chr.15 (-): 18550221- 18550322 |  |
| opossum | SNORD55 | GGGCAGGGCGCGCTGATGACACCTTGGTTACTGCTGCAAACACCGAGATCCTGGTGGGGATGCAAACCTTGGAGAGCTGAGCGCCGCCG | chr.2(-): 9661729- 9661817 |  |
| platypus | SNORD55 | TGGGGTCGGGGGCCATGATGACCCTTCGGTTCATGCTGCGAAACCCGATGGTGCAGTGGGGATGCAGACCTTGGAGAGCTGAGcccccccca | Contig 25597 (+): 8667 - 8758 |  |
| chicken | SNORD55 | CTCTGAGTCTCAATGATGAAGCTTCTCTGACTGCTGCATAAAGCTGAGTGTGCTGTGCGGATGCCAACCTTGGAGAGCTGAGGACTCAGAG | chr.8(+): 21480644- 21480734 |  |
| frog | SNORD55A | CAGAGTGTCAATGATGTAGTAATGCTGCGAAAAAGCTTAACTTTCTGTGTAGAAAACAACCTTGGAGAACTGAGCACTCTG | scaff.261 (-): 1534073- 1534153 |  |
| frog | SNORD55B | GAGTGTAAATGATGTAACAATGTTGCAAAAATGCTTAATTTGCTGTGTTGAAAACAACCTTGGAGAACTGAGCACTT | scaff.261 (-): 1533033- 1533109 |  |
| zebrafish | SNORD55 | TGTGGGTTGATAATAAAGCTTGAGTGTACATGATGAATCCAAATTGCGGAATCCGATTTTCCATGCGGATAAACACCTTGGAGAACTGAACGCTCAAGCATTTATTGAGTTTACG | chr.2(+): 22149544- 22149658 |  |
| fugu | SNORD55 | TTGCTTGAGTAACGTGATGAGACTTAAAAGCGGATTCCAAAATAATTTGCGGACAACTTATACCTTGGAGAACTGATTACTCAAGCAG | Un. (-): 152046150- 152046237 |  |
| mouse | SNORD56 | agcttCTACAATGATGGTAATATTTTTCGTCAACAGAGTTCACCTAGTGAGTGTTGACACCTTGGGTCTGAGTGAagc | chr.2(+): 130103428- 130103505 |  |
| opossum | SNORD56 | TGGCTTCTGCAATGATGGGAAAATTTTTCGTCAACAGCAGTTCACCTTGTGAGTGTTGAATTTCTTAGTCTGAGCAAAGCTG | chr.5 (+): 237929504- 237929585 |  |
| platypus | SNORD56 | GGCTTCCGCCGTGATGGAACGATTTTTCGTCAACAGCAGTTCACCCCCGTGAGTGTTGATAAGCCTGTGTCTGAGCGAAGCC | Contig 17567(-): 10362- 10443 |  |
| lizard | SNORD56 | GCTTTGCTGTGATGCTTACATTTTTCGTCAACAGTATTTCACAATAGTGAATGGTGACGCATCAGATCTGAGCAAAGC | scaff.671 (+): 156397- 156474 |  |
| frog | SNORD56 | GGGCTTTGCAATGATGGGAATAATTTTTCGTCAACAGTATATCACGTTCTGCGTGAATGGTGAGAGATTCTGTCTGAGCAAAGCCC | scaff.1626 (+): 23203- 23288 |  |
| zebrafish | SNORD56 | TTGCATTTTTGCTATGATGCTTTAATTTTTCGTCAACAGTAACCCACCTCAGTGGTTGTTGAGATGATATTCCTGAGCATAAGTGTGA | chr.21(+): 3757975- 3758062 |  |
| tetraodon | SNORD56 | GCATTTCCCGTGGCGTTGTTGCAGTGATGCTCGTATTTTTCGTCAACAGCATTCACCCCTCGGTGGTGTTGAAAACCGATTCCTGAGCAAACGCCACAGAAACTGC | chr.11 (+): 780387- 780492 |  |
| mouse | SNORD57 | tgtTGGAAAGGATGAACGAACTTGGCCTGACCTTCAGAAATGGAGGCAATACAACTGATTTAATGAGCCTGATCCagca | chr.2(+): 130103749- 130103827 |  |
| opossum | SNORD57 | GTTGGAGGTGATGAGCATCCTTACCCTGACTCCAGAGTGGAGGCAAAAACTGATTTAATGAGCCTGATCCAACA | chr.5(+): 237929875- 237929948 |  |
| platypus | SNORD57 | GGGTGGAGATGATGAGCCACTTGGTCCTGAACCTAGCGTGAGGGCAAAAGCTGATTTAATGAGCCTGATCCACCC | Contig 17567(-): 10060- 10134 |  |
| chicken | SNORD57 | GCTGTCGGATGTGATGTGAAGCGCCCACCTGACCCCCCGTGGAGGCAAAAAGCTGATTTAATGAGGCTGATCCGACAGC | Un_random (+): 42528420- 42528498 |  |
| lizard | SNORD57 | CAGCATCGATGTGATGAGATGCAGGCCTGACACATCTGCTGTGAGGCAAAAGCTGATTTAATGAGCCTGATCCAACACTG | scaff.671 (+): 158146- 158225 |  |
| frog | SNORD57 | TGTCGGAGATGATGTCAATCTTTTGCCCTGACCTGAAGCCATGAGGCAAAAACTGATTTAATGAGCCTGATCCGACA | scaff.1626 (+): 23766- 23842 |  |
| zebrafish | SNORD57 | GTGCTGTGAGTGATGACTCTTTCCTCCTGACTCCTACGTGGAGGCGAAAACTGATTTAATGAGCCTGACACAGCAC | chr.21 (+): 3759526- 3759601 |  |
| tetraodon | SNORD57 | CCCGCCGCTGTAAGTGATGACCCCTCTTCCCTGATGTCCCTGAAGGTCAAAACTGGTTTAATGAGCCTGATGCAGCCGGAGGG | chr. 11 (+): 780707- 780789 |  |
| mouse | SNORD58A | GCTGCAGTGATGACTATCTTAGGACACCTTTGGATTTACCGTGAAAAGAAGTAACCTCTGAGCAGC | chr.18 (+): 75161108- 75161173 |  |
| mouse | SNORD58B | GATGGCTGCGATGATGTCATATCTTAGGACACCTTTGGATTAATCATGAAAATAACTATCCTCTGAGCAGCTGTT | chr.18(+): 75160707- 75160781 |  |
| mouse | SNORD58C | GGGTTGCTGTGATGACTATCTTAGGACACCTTTGGATTAACCGTGAAATCAAACAAGTGCTGAGCAACCT | chr.18 (+): 75161531- 75161600 |  |
| opossum | SNORD58A | GTGGCTGCAGTGATGACGATTCTTAGGACACCTTTGGATTAACCATGAAAACAATGATAATTCTGAGCAGCTAC | chr. 3 (-): 69315860- 69315933 |  |
| opossum | SNORD58B | GCTGCAGTGATGACTTTTCTTAGGACACCTTTGGATTAACAATGAAAATAAAAACAATACTGAGCAGC | chr. 3 (-):  69315318- 69315385 |  |
| opossum | SNORD58C | TTTCTTGGTTGCTATGATGACAATCTTAGGACACCTTTGGATTAAATAATGAAAAAAAAATATGTTCTGAGCAATTTACAAA | chr. 3 (-):  69314618- 69314699 |  |
| opossum | SNORD58D | TTTGGTGGCAGTGATGACTCTCTTAGGACACCTTTGGATTAAGCAATGAAAATAATTACTACCTGAGCCATCAAG | chr. 3 (-):  69314261- 69314335 |  |
| opossum | SNORD58E | GGTTGCTATGATGACAATCTTAGGACACCTTTGGAATAACCATGAAAAAAAACATGTATTCTGAGCAACC | chr. 3 (-):  69313706- 69313775 |  |
| platypus | SNORD58A | GCTGCGATGATGACCTTCTTAGGACACCTTTGGATTAACTGTGAAAACAAGGAAATTCTGAGCAGC | Contig 16366(+): 4488- 4553 |  |
| platypus | SNORD58B | GCTGCTATGATGACTTTCTTAGGACACCTTTGGATTAACTGTGAAAATACTAAAACATTCTGAGCAGC | Contig 16366 (+): 4997- 5064 |  |
| platypus | SNORD58C | GGTTGCTGTGATGACATTCTTAGGACACCTTTGGATTAATCGTGAAAATAAATTTCTATTCTGAGCAACC | Contig 16366(+): 6080- 6149 |  |
| platypus | SNORD58D | TTGATGGCGGTGATGACATTCTTAGGACACCTTTGGATTAACAATGAAAACAATCATCGACTGAGCCATCAG | Contig 16366 (+): 6444 6515 |  |
| platypus | SNORD58E | TGGTTGCTGTGATGACAATCTTAGGACACCTTTGGATTAACCGTGAAAAGAAATATATTCTGAGCAATCA | Contig 16366(+): 6888- 6957 |  |
| chicken | SNORD58A | GGCTGCGGTGATGACTCTCTTAGGACACCTTTGAGTTGAGTTGTGAAAAGAAACGTAAGCTCTGAGCAGCT | chr.Z(+): 965266- 965336 |  |
| chicken | SNORD58B | GAGATTGCTGTGATGACTTTCTTAGGACACCTTTGGATTCAATGTGAAAAGAAACTTGTATTCTGAGCAATCTT | chr.Z (+): 966942- 967015 |  |
| chicken | SNORD58C | TTGATGGCGGTGATGACTATCTTAGGACACCTTTGGAACAACCATGAAATAATAACTTTTTTTTCTGAGCCATCAG | chr.Z(+): 967496- 967571 |  |
| chicken | SNORD58D | GGGTTGCTGTGATGACTTATCTTAGGACACCTTTGGAATACTCATGAAAAAAAATGTTATTTCTGAGCAACCT | chr.Z(+): 968024- 968096 |  |
| lizard | SNORD58A | TAACTGCAATGATGACCAGCTTAGGACACCTTTGGAATCCTCATGAAAACAGCTAAGTGATTCTGAGCAGTTA | scaff.330 (+): 345158- 345230 |  |
| lizard | SNORD58B | CAGGTTGCTGTGATGACTTTCTTAGGACACCTTTGGATTAACCATGAAAAGAATGTTTAGGTCTGAGCAGCCTG | scaff.330 (+):  346246- 346319 |  |
| lizard | SNORD58C | CAGTGGCAATGATGACTATCTTAGGACACCTTTGGAATCACCATGAAACAAATGCAAATTCTGAGCCACTG | scaff.330 (+):  347050- 347120 |  |
| frog | SNORD58A | TGGAACTGCAATGATGACTTTCTTAGGACACCTTTGGAAATAAAAGATGAAAATAACACTTATAATACTGAGCAGTTTCA | scaff.217 (+): 1149673- 1149752 |  |
| frog | SNORD58B | GGTTGCAGTGATGACTATCTTAGGACACCTTTGGAAAACCCATGAAACAAAAAATTTTATTCTGAGCGACC | scaff.217 (+): 1151254- 1151324 |  |
| frog | SNORD58C | AAATTGTGGCAATGATGACTATCTTAGGACACCTTTGGAATCAAACATGAAACCAACCTTAATTCTGAGCCACTATTT | scaff.217 (+): 1152541- 1152618 |  |
| frog | SNORD58D | GGTGGCAATGATGACTATCTTAGGACACCTTTGGAATCAAACATGAAACCAAACTTCATTCTGAGCCACT | scaff.217 (+): 1153278- 1153347 |  |
| zebrafish | SNORD58 | ATGGCTGTAATGATGACCACCACTTAGGACACCTCTGAATTAATGAAGAGACGAGTTTCTGAGCAGCTGT | chr.21 (-): 13362829- 13362898 |  |
| tetraodon | SNORD58A | CGGATGCTGTGATGACTTCTTAGGACACCTTTGGATTAGTCATGAAAATAAAAGTGAATTACCTGAGCAAACG | chr. 4 (+): 2731703- 2731775 |  |
| tetraodon | SNORD58B | GAAAAACTGCGGTGATGACTATCTTAGGACACCTTTGGATAAACGATGAAACTACTCATGTGTCTGAGCACCTTC | chr. 4 (+):  2732377- 2732451 |  |
| tetraodon | SNORD58C | TGTTGCACTGATGACCACTTAGGACACCTTTGGATGAACCATGAAAACAAACTGTTAAACCTGAGCAGCA | chr. 4 (+):  2733026- 2733095 |  |
| mouse | SNORD59A | tctcCATTCTGTGATGACTTTACCAAATGACTTTCGTTCTTCTGAGTTTGCTGAAGCCACACTCAGGTGCTGAGAGGGGagg | chr.10(+): 127521195- 127521276 |  |
| mouse | SNORD59B | ccttcagtgatgattagcttcTGACTTTCGTTCTTCTGAaTTTGCTGAAGCCAgatgccgttcctgagaagg | chr.10(+): 127522345- 127522416 |  |
| opossum | SNORD59A | AGCTCTCTTCCATGATGATTTCTAACTAGATGACTTTCGTTCTTCTGAGTTTGCTGAAGCCATGCTTAGAATCTGAGCAAGAGAGTT | Un (-): 101335535- 101335621 |  |
| opossum | SNORD59B | TGTTTTAGAGCAGCATCCTTCACTGATGAGTAGCTTTCTGACTTTCGTTCTTCTGATTTTGCTGAAGCCAGATGCCACTTCTGAGAAGGAAAAACA | Un (-): 101335535- 101335621 |  |
| platypus | SNORD59 | TCCTTCGCTGATGAGTGACTCTTTGACTTTCGTTCTTCTGAGTCTGCTGAAGCCATTTGTCTATTCTGAGAAGGA | Contig21023 (+): 5895- 5969 |  |
| chicken | SNORD59 | TTCCTTCGCTGATGAGTAACCTTTTGACTTTCGTTCTACTGAGCTTGCTGAAGCAACGTTCATTACCTGAGGAGGAA | E22C19W28_E50C23 (+): 894549- 894625 |  |
| lizard | SNORD59A | TTCCTTCCTTGATGATCCATAAAATATGACTTTCGTTCTTCTGAGCTTGCTGAAGCCATGTTTTTGATCTGAGAAGGAA | scaff. 42 (-):450149- 450227 |  |
| lizard | SNORD59B | TTTTTTCCTTCACTGATGAGTAAACCCAAGACTTTCGTTCTCCTGAAACTGCTGAAGCAACTCTCTTTTCTGAGAAGGAAAAGA | scaff.42 (-):48220- 448303 |  |
| frog | SNORD59A | TTTCCTTCTATGATGATTTTTTCATTTGACTTTCGTTCTTCTGAGTTATTCTGAAGCGATTTTATATAGTCTGAAAAGGAAA | scaff.101 (+): 1038648 - 1038729 |  |
| frog | SNORD59B | TTACATGCCTGCAGTGATGAGTAAACTGACTTTCGTTCTACTGAATTTGCTGAAGACATATTTTTATCTGAAAAGGCAGTAA | scaff.101 (+): 1040255- 1040336 |  |
| zebrafish | SNORD59A | CTCTTGATGATGATTTGACAATTTGACTTTCGTTCTTCTGAGTCTGATGAAGCCAAATTTTGTCCTGAAAGAG | chr.23(+): 15730161- 15730233 | The host gene (naca) has two copies. |
| zebrafish | SNORD59B | CTCTTCTATGATGAATAAACTTTTGACTTTCGTTCTTCTGAGTTTGTCCTGAAGCCAGTTTATTTGTTCTGAGAAAAG | chr.23(+): 15730706- 15730783 |
| zebrafish | SNORD59C | CTCTTGATGATGATTTGACAATTTGACTTTCGTTCTTCTGAGTCTGATGAAGCCAAATTTTGTCCTGAAAGAG | chr.23(+): 15673991- 15674063 |
| zebrafish | SNORD59D | CTCTTCTATGATGAATAAACTTTTGACTTTCGTTCTTCTGAGTTTGTCCTGAAGCCAGTTTATTTGTTCTGAGAAAAG | chr.23(+): 15674536- 15674613 |
| tetraodon | SNORD59A | CGTCTCTTCTGTGATGATCAACACATTGACTTTCGTTCTTCTGAGTCTACTGAAGCCACCTCGTTGTCCTGAGATGATG | chr. 9 (-): 7542925- 7543003 |  |
| tetraodon | SNORD59B | GCTCTTCACTGAGGATCTCAACTTTGACTTTCGTTCTTCTGAGTTTCATGAAGCTGATGTTTTTCACACTGAGAAGAGC | chr. 9 (-): 7542025- 7542103 |  |
| mouse | SNORD60 | cccaAGCCCGTGATGAATTAATTTCTGACACCTCGTATGAAAACTGCATGCGTAGTCTGATTACATTATAAGACTGAGGCTTggg | chr.17(+): 24665417- 24665501 |  |
| opossum | SNORD60A | CTGTAGCCAGTGATGAAATCCACATTTCTGACACCTCGTATGAGTATTGCCACTACCAATACTTGATTATGCTTGAGATCTGAGGCTGCAG | Un (+): 48615293- 48615383 |  |
| opossum | SNORD60B | TGAGCCAGTGATGAGACATTTCCATTTCTGACACCTCGTATGAAATCTGCATGTGCAGTTTGATTATATAGCAACCCTGAGGCTCA | Un (+): 48615543- 48615628 |  |
| platypus | SNORD60A | CTCGGGCGCTGCAGCCGGTGATGAGCTCCCACATTTCTGACACCTCGTATGAGTATTGCACGAGCAATACCTGATTTTGCTTGGGTGCTGAGGCTGCATGGCCCGAG | Contig 20555(+): 3573-3679 |  |
| platypus | SNORD60B | TGAGCCGATGAAGACACTTTGCGATTTCTGACACCTCGTATGAGAGCTGCATCTGCAGCCTGATTCTAACACTGGACTGAGGCTCG | Contig 20555(+): 4222-4307 |  |
| chicken | SNORD60 | GCGAGCCGGGGATGATTTCTGCCTTTCTGACACCTCGTCTGAGGGCGGCGGGCGGCCGTGGCCACTGCTGACGCGCTACGGCTGAGGCTCGC | chr.14 (-): 6454558- 6454649 |  |
| lizard | SNORD60A | GCCCCAGCCCGTGATGAGCTCTTCCATTTATTTTCTGACACCTCGTCTGAACACTGCGCTTGCAGTGAACTGATTATGAACATGAGCTGAGGCTTGAGGC | scaff.1914 (-): 30239- 30338 |  |
| lizard | SNORD60B | CCTCAGCCCGTGATGAGCTCTTCCATTTATTTTCTGACACCTCGTCTGAACGCTGCGCTTGCAGTGCACTGATTATGGACATGAGCTGAGGCTTCGAGG | scaff.1914 (-): 24958- 25056 |  |
| frog | SNORD60A | TTGCCAAGGATGTATCCTGAATTCTGACACCTCATATGAAACTGTAACAACAGCTTCTTGATTCTGTCCTTTGGGCTGAGGCAA | scaff.27 (+): 3129886 3129969; scaff.3248 (-):2406-2489 | Scaffold 27 and short scaffold 3248  are probably represent the same genomic locus. |
| frog | SNORD60B | TGCCAGTGATGTTCCCTAAATTCTGACACCTCATATGAAACTGTTAATAACGGCTTCTTGATTCTATTGCTTGGGCTGAGGCA | scaff.27 (+): 3131096- 3131178;  scaff.3248 (-):1197-1279 |
| frog | SNORD60C | CTTTGCCAAGGATGTTCCCTAAATTCTGACACCTCATATGAAACTGTTAATAACGGCTTCTTGATTCTATTGCTTGGGCTGAGGCACTAAG | scaff.27 (+): 3131360- 3131450; scaff.3248 (-):925-1015 |
| frog | SNORD60D | TTTCTTTGCCAAGGATGTTCCCTAAATTCTGACACCTCATATGAAACTCTCTTGATTCTATTGCTTTGGGCTGAGGCATAAA | scaff.27 (+): 3131632- 3131713;  scaff.3248 (-):662-743 |
| frog | SNORD60E | TGCCTAGGATGGACCCTAAATTCTGACACCTCATATGAAACTGTCTTGATTCTATTGCTTGGGCTGAGGCA | scaff.27 (+): 3131893- 3131963;  scaff.3248 (-):412-482 |
| frog | SNORD60F | GGCTTTGAGTTGCCTCTTATTACTTTGCCAAGGATGTTCCCTAAATTCTGACACCTCATATGAAACTGTAAACACAGCATTGTGATTTTGTTCTTTGGGCTGAGGCTTTGCTCTGCAACATAAAGTT | scaff.27 (+): 3132133- 3132259;  scaff.3248 (-):116-242 |
| zebrafish | SNORD60A | GAGCCGATGATGACTTCAATTTACTTTTTCTGACACCTCGTATGAACGCTGTAATTCAGCAGCTGATTATAAACTTAAGTCTGAGGCTC | chr.1(+): 5158539- 5158627 | The host gene has two copies. |
| zebrafish | SNORD60B | GAGCCGCTGATGACTTCAGTTTACTTTTTCTGACACCTCGTATGAACGCTGTAATTCAGCAGCTGATTATTAAGTTTAGTCTGAGGCTC | chr.1(+): 5159216 5159304 |
| zebrafish | SNORD60C | GAGCCGCTGATGACTTCTATTTTAACTTTTTCTGACACCTCGTATGAATGCTGTATTTCAGCAGCTGATTATAAACTTTTAGTCTGAGGCTC | chr.1(+): 5159913- 5160003 |
| zebrafish | SNORD60D | GAGCCGATGATGACTTCAATTTACTTTTTCTGACACCTCGTATGAACGCTGTTATTCAGCAGCTGATTATAAACTTAAGTCTGAGGCTC | chr.1(+): 5292382- 5292470 |
| zebrafish | SNORD60E | GAGAGCCGCTGATGACTTCAGTTTACTTTTTCTGACACCTCGTATGAACGCTGTAATTCAGCAACTGATTATTAAGTTTAGTCTGAGGCTCTT | chr.1(+): 5293019- 5293111 |
| zebrafish | SNORD60F | GAGCCGCTGATGACTTCCATTAACTTTTTCTGACACCTCGTATGAAAGCTGTATTTCAGCATCTGATTATAAACTTAAGTCTGAGGCTC | chr.1(+): 5293706- 5293794 |
| mouse | SNORD61 | aagacaaGCTGTGATGAATTTGAATCCACTGATCTTCCGACATGATAAATGCATTGTCCTCTAAGAAGTTCTGAGCTTgctt | chr.X(-): 54644622- 54644703 |  |
| opossum | SNORD61 | CGGCTGGGCGATGATGAATGTCAATTCTATTGAACTTATGAAGTGATGATGTATTTGTTGTTTACTCTAAGAAGTTCTGAGCTGCTG | chr.X (-): 35828534- 35828620 |  |
| platypus | SNORD61 | GGGTGGTCCATGATGAATTTCACGGCGTTGATCGTCCGAAGTGATGATGCATCCTTGTCCTCTAAGAAGTTCTGAGATCCC | chr.6 (+): 7382937- 7383017 |  |
| chicken | SNORD61 | AGCTATGATGACTCCTATAATGTTGATCTTACGACATGATCATGTTCTCTAAGAAGTTCTGAGCT | chr. 4 (-): 4430893- 4430957 |  |
| lizard | SNORD61 | AGCTTTTTGGGTCTGCTGTGATGAATGTAACAGCTTTGATCAGCTGAGATGATAGGGCTCATGTGTCCTCTAAGAAGTTCTGAGCAGGTCTTTGTAAAAGCT | scaff.772 (-): 354835- 354936 |  |
| zebrafish | SNORD61A | GTAAGCTGTGATGTTTTTTTTTGTGGCAGTGATCTACTTGAGCTGATCTAGTCCACAATTTCCTCTAAGAAGTTCTGAGCTTGC | chr.14(-): 36097873- 36097956 |  |
| zebrafish | SNORD61B | GTTGCTGTGATGACACATTCTTGATGATCTGTTTGAGTTGATTGTCCTCTAAGAAGTTCTGAGCCAAC | chr.14(-): 36097531- 36097598 |  |
| zebrafish | SNORD61C | TGTAAGCTGTGATGAATTCTTGTGGTGGTGATCTACTTGAACTGATCTAGTCCACAATTGTCCTCTAAGAAGTTCTGAGCTTACG | chr.14(-): 36096552- 36096636 |  |
| mouse | SNORD62A | ggtctcagtgatgtgtttccaatagatCCTTCTGACCCTCCACTGTGGACTCAAAgCAGGGAGATGAAGAGGACAGTGACTGAGAGact | chr.2 (+): 32077488- 32077576 |  |
| mouse | SNORD62B | gtctcagtgatgtgtttccaatagatCCTTCTGACCCTCCACTGTGGACTCAAAgCAGGGAGATGAAGAGGACAGTGACTGAGAGac | chr.2 (+): 32081172- 32081258 |  |
| opossum | SNORD62 | TTGAGTCTCTATGATGTATTCCAATAGATCCTTCTGACCCTTCAATGTGTACTCAGTAAAAGGGAAATGAAGAGACAATACCTGAGAGACTTAA | chr.1 (+): 458235524- 458235617 |  |
| platypus | SNORD62A | TGAGTCTCTATGATGTATTCCAATAGATCCTTCTGACCCTCCAGTGTGCATTCCAAAGAAGGGGAAATGAAGAGATGTGTGACTGAGAGACTTG | chr. 4 (-): 20025603- 20025696 |  |
| platypus | SNORD62B | GTCTCTATGATGTGTTCCAATAGATCCTTCTGACCCTCCACTGTGCATTCCAAAGAAGGGCAAATGAAGAGATGTGTGACTGAGAGAC | Contig 139675 (-): 842 - 929 |  |
| chicken | SNORD62 | TGCTCTTGGTGTACATGATGACACAATGGATCCTTCTGATCCTTCCATCAAAGTACTCTAGGAACTGGATCTGAAGACTCCTAAAAGCTGAAAACACCAAGCAGCA | chr. 17 (+): 6820228- 6820333 |  |
| lizard | SNORD62 | CTTTGGCCTCAGTGATGTGTTTCTCTCAATGGATCCTGCCGAGCCCTTTCTAAAGGGCCTTGAAGAGGATTCTGTGGCTGAGAGGCCAAgg | scaff. 2889 (+): 6630- 6720 |  |
| frog | SNORD62A | TTCTCTGTCTCTATGATGTTCCAATAGATCCTTCTGATCCTTCACTATGTACTCAGAATGGACCTGAGGAGATTGTATTACTGAGGGGCAGAGAA | scaff.191 (-): 1061584- 1061678 |  |
| frog | SNORD62B | TCTCTGGGATGTTCCAATAGATCCTGCTGACCTTTCACTATGTACTCAGAAAGGATCTGAAGAGA | scaff.191 (-): 1060475 1060539 |  |
| frog | SNORD62C | GGTCTCCGGGATGTTCCAATAGATCCTGCTGACCCTTCACTATGTACTCAGAAGGGATCTGAGGAGATCCAGTAACTGAGAGACC | scaff.191 (-): 1059516- 1059600 |  |
| human | SNORD63B | GTATATTTGTGCCGTGATGTATTTGTCAACACATCACTCTGAAGAAAAGTATGTGGTGACTTTCTGTGACTGAGCATGATAC | chr.5(-): 137922554- 137922635 |  |
| mouse | SNORD63 | TGTGCGATGATGCATTTTATTCAACACATCATTCTGAAAATAGATGTGTAGAGAAATGATAACTGAGCACA | chr.18(-): 35102298- 35102368 |  |
| dog | SNORD63A | TTGTGCAATGATGGATTTTATTTATCAACACATCATTCTGATAAGATAGGTGTGTGGAAAACTAATGACTGAGCACAG | chr.11(-): 29147976- 29148053 |  |
| dog | SNORD63B | TATTGCTATTTGTTCAGTGATATATTGCCAACACATCATTCTGAAGAAAACTGTGGTGACTTCTGTGACTGAGTATA | chr.11(-): 29145328- 29145404 |  |
| opossum | SNORD63 | TGGAGTATATTTGTGCTATGATGTATTTGTTTAACCCCTCATTCTGAAAAAAAGAAAAAATGTGGTTAATCACTGAAACTGAGCACAAAATATACCCA | chr.1 (-): 329586295- 329586392 |  |
| platypus | SNORD63 | TGGTTCAGCCGGGGAGCCCTGTGCCGTGATGGATTTAACGATCGACACATCATTCCGAAACGAGCGGTGTGGACAGCTAATGACTGAGCACAGAAACCCTGCTGAACCG | Contig 5329 (-): 24944- 25052 |  |
| chicken | SNORD63 | GTGTGTGCCATGATGTAATGTCAACACATCATTCTGAAGAGGAATTGTGTGGAGACCTTCTGTAACTGAGCACAGCAC | chr.13(+): 2525864- 2525941 |  |
| lizard | SNORD63 | TAGTGTGTGCTGTGATGCAATTATATCAACGCCTCATTCTGAAATGTTATGTGGAAGATTGATGACTGAGCATGCACTG | scaff.109 (-): 1294143- 1294221 |  |
| frog | SNORD63 | GCAATCGGTACAAGTGATGAAGATTAAAACAACACATCATTATGACAAATCAAGTGATATTGTAAACGCTTTCTGATGTACCGATTGC | scaff.76 (+): 2106906- 2106993 |  |
| mouse | SNORD65 | gatatcAAATGATGATATCACCTAAAATAGCTGGAATTACCGGCAGATTGGTAGTGGTGAGCCTATGGTTTTCTGAAGatatc | chr.11(+): 62418011- 62418093 |  |
| dog | SNORD65 | AAGGATATCAAATGATGAGATCACCCAAAATAGCTGGAATTACCGGCAGATTGTATAGTGGTGAATGTAATGGTTTTCTGAAGATAACCTT | chr.5(-): 42487266- 42487356 |  |
| cow | SNORD65 | AAGGATATCAATGATGAAATCACCCAAAAGAAGCTGGAATTACCGGCAGATTGTGTAGTGGTGAACCTACGGTTTTCTGAGGATATCTTT | chr.19(-): 34099680- 34099769 |  |
| opossum | SNORD65A | CTAAGGCTTTTTTGTGATGAAACAAAACCGAAACAGCTGGAATTACCGGCAGATTGTCAGTGGTGACAAGGTTTTATTCTGATTCAGCTTGG | chr. 2 (+): 520355091- 520355182 |  |
| opossum | SNORD65B | TGATATGGAGATACTAAATGATGAGATTCACCCAAACAGCTGGAATTACCGGCAGATTGTTCAGTGGTGAGCCCGCGGTTTTCTGATTCTATCTCCAGTCA | chr.2 (+): 520355933- 520356033 |  |
| platypus | SNORD65A | AGGCCTCAGTGATGAGAACAAACCCCAAAACAGCTGGAATTACCGGCAGAGTCCCCGTGGTGACTCCGGTTTCTCTGAGTGAGCCT | Ultra 42 (+): 1071453- 1071538 |  |
| platypus | SNORD65B | CCGCGGGAAGTTATTGGATGATGAAAGTTACCAAAATAGCTGGAATTACCGGCAGATGGAATGGTGGTGAGCCATGGTTTTCTGAAGATGCCTGCGG | Ultra 42 (+): 1072369- 1072465 |  |
| chicken | SNORD65 | GATATCATATGATGAAACTAACCAAAAATTGCTGGAATTACCGGCAGATTGACTGGTGGTGAGCAAAGGTTTTTCTGATGATATC | chr.19 (-): 5110005- 5110089 |  |
| lizard | SNORD65 | GGAGGGGGATTGTGATGAAACCAAACCTACAAAGCTGGAATTACCGGCAGACTTTTGCTGTTGAGAAGGTTCTCTCTGAGCCGCCCCGTCC | scaff. 2527 (+):7768- 7858 |  |
| frog | SNORD65A | GTGCCTTATGATGAAGTGACCATAAATATAGCTGGAATTACCGGCAGACTGTAAGTGGTGATATTGGTTGCTCTGAGGGCAC | scaff.178 (-): 1753232- 1753313 |  |
| frog | SNORD65B | TGTGCCTTATGATGAAGTGACCATAAATATAGCTGGAATTACCGGCAGACTGTAAGTGGTGATTATTGGTTGCTCTGAGGGCAC | scaff.178 (-): 1752137- 1752220 |  |
| frog | SNORD65C | TAGCTTTGGCTTTAAGGTAACTGTTCCCTGTGATGAATTAACCTGAAAAATAGCTGGAATTACCGGCAGATTGCTGGTGGTGATATTGGTTGTCTGAGCTTTGTATGCAAAGGGTAAATTG | scaff.178 (-): 1751758- 1751878 |  |
| frog | SNORD65D | TGCCTTATGATGAAGTAACCATAAATATAGCTGGAATTACCGGCAGACTGCAGGTGGTGATACTGGTTGCTCTGAGGGCA | scaff.178 (-): 1750255- 1750334 |  |
| zebrafish | SNORD65A | TGTGTTGTGATGAAACTTAAACTGAAAATAGCTGGAATTACCGTCAGATGGTTCAGTGGTGATGATCGGTTTTCTGAGCACG | chr.21(+): 32549132- 32549213 |  |
| zebrafish | SNORD65B | TTGATTAAATATGATGAAACCTAAACTGATAATAGCTGGAATTACCGTCAGATTGATCAGTGGTGATAATCGGTTTTCTGACATGAATCAA | chr.21(+): 32549352- 32549442 |  |
| tetraodon | SNORD65A | GAATGTTCTGATGAAATAGAAACGGAAATAGCTGGAATTACCGGCAGATTGTTCAGTGGTGATTCTCTGTTTTCTGAACATTC | chr. 7 (-): 7281318- 7281400 |  |
| tetraodon | SNORD65B | TGATGTGGTGATGAAATCTAAACTGAAAATGCTGGAATTACCGTCAGATTGTTCAGTGGTGATATTTGGTTTTCTGAACATCG | chr. 7 (-): 7280779- 7280861 |  |
| mouse | SNORD66 | tTTCCGCTGATGACTTCTGTCAGTGCCACGTGTCTGGGCCACTGAGACCACATGATGGGATTGAGGACCTGaggaag | chr.16 (+): 20684311- 20684387 |  |
| dog | SNORD66 | ctTTCCACTGATGACTTCTTGTTAGTGCCACGTGTCTGGGCCACTGAGACCACATGATGGAACTGAGGATCTGag | chr.34 (+): 20212311- 20212385 |  |
| opossum | SNORD66A | CTGGGTCTTCACTGATGAGATCCTGTTTGTGCCGTCGTGTCTGGGCCACTGAAGCCTTGTGATGGAGTTGAGGATCTGAGGAGACCCAG | chr. 2 (+): 540244445- 540244533 |  |
| opossum | SNORD66B | GAGGCTGGGTTCTCCACTGATGACCACCTATTTGGGCCGTTGTGTCTGGGCCACTGAGTTTTGTTGATGGAACTGAGGTTCTGAGGAGAGGCCACCTT | chr. 2 (+): 540247672- 540247769 |  |
| opossum | SNORD66C | GACTCTCCAGTGATGACCTTTTGAGTACCATGTGTCTGGGCCACTGAGATACTGTGATGGAGCTGAGGTTCTGAGGAGAGTT | chr. 2 (+): 540248360- 540248441 |  |
| platypus | SNORD66 | ccggccgTCTCCGCTGACGAATGCTTGTTTGAGCCATCGTGTCTGGGCCACTGACACCCCGTGATGGAACTGAGGATCTGAGGAGAGGCTGG | Contig 34618 (+): 1575-1666 |  |
| chicken | SNORD66A | TGCCTTCTTGTGATGAATTCTACTTTGTGCCATCGTGTCTGGGCCACTGATACTTCATGATGGTAAAGAGGATCTGAGGGGGCA | chr.9(+): 17011522- 17011605 |  |
| chicken | SNORD66B | CCCCTGCTTCTCTGTGATGATCAAGCCTTTGAGCCACTGTGTCTGGGCCACTGATGTCAGTGATGGAACTGAGGATCTGAGCAGGCAGACGGGG | chr.9(+): 17012730- 17012823 |  |
| lizard | SNORD66A | GGGGTCTTGTTGAGTCTTCTGTGATGACCTCCTTTTTGAGCCATCGTGTCTGGGCCACTGACACACTGTGATGGAGTTGAGGATCTGAGCGGACAACACACCCC | scaff. 517 (+): 354020- 354123 |  |
| lizard | SNORD66B | TGTTGAGTCTTCTATGATGACCTCTTTTTTGAGCCATCGTGTCTGGGCCACTGACACACTGTGATGGAGTTGAGGATCTGAGCAGACAATA | scaff. 517 (+):  357677 - 357767 |  |
| lizard | SNORD66C | CTGCCTACGTGATGAGCTCCTAGTTAGTGCCATCGTGTCTGGGCCACTGATAACCCTGTGATGGAACTGAGGATCTGAATGGCAG | scaff. 517 (+):  359525- 359609 |  |
| zebrafish | SNORD66 | GCTCCACTGATGACCTTCATTAAGAGCCACGTGTCTGGGCCACTGATCTACACTGATGGGATTGAGGAACTGAGGAGC | chr.2(+): 12553342- 12553419 |  |
| human | SNORD67 | CTGTGAGAGTGATGAGTTGCACACTGGTGGAGCCATGGTATCAGGTGATACAGGCACCACTCAGTATCACCCTGGTGACAAAATCAAGTGCACAGGGGCCATCTGACTCACAG | chr. 11(-): 46740514- 46740626 |  |
| mouse | SNORD67 | CTGTGAGAGTGATGAGTTGCACACTGGTGGAGCCATAGTATCAGGTGATACAGGCACCACTCAGTACTACCCTGGTGACAAAATCAAGTGCACAGGGGCCATCTGACTCACAG | chr. 2 (+): 91436226- 91436338 |  |
| opossum | SNORD67 | GACTGTGAGAGTGATGAGTTGCACATTAGTGTGGCCATAGCATCAGGTGATGCAGGCTATACTCAGTAATAAACTGGTGACAAAGCCATGTGCACAGGGGCCATCTGACTCGCAGTC | chr. 5 (-): 283781513- 283781629 |  |
| platypus | SNORD67 | GACTGTGAGGGTGATGAGTTGCACACTGGTGGAGCCAAAGCATCGGTCGATGTGGGCGCCGCTCAGTATCACCTTGGTGACAGAACCAGGTGCACAGGGGCCATCTGACTCTCAGTC | Ultra 7(-): 105992- 106108 |  |
| chicken | SNORD67 | GCGAGAGTGATGAGCTGCACTCTGGTGGAGCAACCATGTCAGGTGACGTGGTCTCTGCTCAGAATCTCCCTGATGACAAAACTAGGTGCACAGGGGCCATCTGACTCGC | chr. 5 (+): 25578974- 25579082 |  |
| lizard | SNORD67 | GCGAGAATGATGAGTTGCACGTTTGGTGGGGCTGAGTGTCAGATGACACAAGCACCACTCAGAAACCACCCTGATGACAATACCAAGTGCACAGGGGCCATCTGACTCGC | scaff.130 (+): 1937045- 1937154 |  |
| frog | SNORD67A | TGGAGTATGATGAGTTGCACTAAAGGGAGAGGCGGTGCTGTGTACTGCCAAACCCGACAGACCCAGACTGGTGACAGAACCATGTGCACAGGGGCCATCTGACTCCA | scaff. 82 (-): 2704545- 2704651 |  |
| frog | SNORD67B | AAGCTGCTGGAGGATGATTAGTTGCACCAAGGGGCCTGCGGTGCTGTGTACTGCGGAGCCTCACAGAGCAAAAATGGTGACAGAGCCACGTGCACAGGGGCCAGCTGACTCCATGCAGCTT | scaff. 82 (-): 2703327- 2703447 |  |
| frog | SNORD67C | TGGAGTATGATGAGTTGCACTAAAGGGAGAGGCGGTGCTGTGTACTGCCAAACCCGACAGACCCAGACTGGTGACAGAACCATGTGCACAGGGGCCATCTGACTCCA | scaff.82 (-): 2679551- 2679657 |  |
| tetraodon | SNORD67 | GGGTTTGAGAGTGATGAGTTGCACGAATCAGTTGGGGCGCTCGGTGCAGTGGCAACGGCGCCCCAGTCCGAGCAGCGCTGAGGACACCCTGGGTGCACAGGGGCCATCTGACTCaaccc | chr. 5 (+): 11836641- 11836759 |  |
| medaka | SNORD67 | GGGAACTGGGTGTGAGAGTGATGAGTGGCACTGAAAAGTTGGCGTGTTGTTGCAGTGGCAACAAAACCCCAATCCGAACCGCCATGGGGACACACCATGTGCACAGGGGCCATCTGACTCCACCCACCC | chr.14 (+): 8977415- 8977543 |  |
| mouse | SNORD68 | gggctcggtGGAATGATGACATTCTCCGGAATCGCTGTACTGACTTGATGAAAGTACTTTTGAACCCTTTTCCATCTGATgacaccgagttt | chr.8(+): 125626925- 125627016 |  |
| rat | SNORD68 | GGGCTCGGTGGAATGATGACATTCTCCGGAATCGCTGTACTGTCTTGATGAAAGTACTTTTGAACCCTTTTCCATCTGACTACGAGCTT | chr.19 (+): 53438256- 53438344 |  |
| dog | SNORD68 | gcccgcgggccAATGATGACAGTCTCCGGAATCGCTGTACTCACGTGATGAAAGTACACTCGAACCCTTTTCCATCTGACGGCCAAGCGCGGC | chr.5(-): 66977374- 66977466 |  |
| opossum | SNORD68A | AGAAACCTGATCTCACAGTCAAATGATGACATATTCTCCGGAATCTCTGTACTGCCTCTGATGAAAGTACATTTGAACCCTTTTCCATCTGA | chr.1(-): 664202248- 664202339 |  |
| opossum | SNORD68B | CCATTGGTCTCAGTCAAATGATGACAAATTCTCCGGAATCTCTGTACTGCCTCTGATGAAAGTACATTTGAACCCTTTTCCATCTGATGACTGTATTCTGG | chr.1(-): 664201785- 664201885 |  |
| platypus | SNORD68 | GCCTCACAGTCAAATGATGACACATTCTCCGGAATCTCTGTACTGCCTCTGATGAAAGTATAATTGAACCCTTTTCCATCTGATGACTGCGGC | chr.11 (-): 5855474- 5855566 |  |
| chicken | SNORD68 | GTGGGGCCGGCCGCAGTCAGGTGATGAGAAGTCTCCGGAATCGCTGTACTGCATTGATGATTCCCTCGAACCCTAGTCTGTCTGATGGCTGCGGCAGCTCTGT | chr. 11 (+): 20693443- 20693545 |  |
| lizard | SNORD68A | TGGATCTGTTCCAGCCAGATGATGAAAATTCTCCGGAATCTCTGTACTCCATTGATGATCCCTTGAACCCATATCTGCTGATGGCTGTGGTAATGGTTTA | scaff.822 (+): 232629- 232728 | The second antisense element is disrupted. |
| lizard | SNORD68B | GCAGTCAGATGATGACACTGTCTCTGGACTATCTGTAGTGTCCATGATGACCATGTCTTGAACCCTTTTCCAGCTGATGACTGC | scaff.822 (+): 234074- 234157 | The first antisense element is disrupted. |
| frog | SNORD68A | CTATGGTCAAATGATGACAATTTCTCCGGAATCTCTGCATCTTTGTGATGATTTACAGTTTGAACCCTTTTCCAGCTGATGACCATAG | scaff.66 (+): 2603865- 2603952 |  |
| frog | SNORD68B | GACCAGTCACGTGATGACAATTTCTCCGGAATCTCTGCATCTCTGTGATGATTCCTTTGAACCCCATATTCTGATGACTGTTC | scaff.66 (+): 2604967- 2605049 | The second antisense element is disrupted. |
| frog | SNORD68C | TGCAGTCAGTTGATGACACTTTCTCCGGAATCTCTGCATTTTCTGTGATGATTTGTGGTGAACCCTTTTCCAGCTGATGACTGTA | scaff.66 (+): 2605537- 2605621 |  |
| zebrafish | SNORD68A | ACAGTCAAATGATGACAATTCTCCGGAATCTCTGTACTTCCTTGATGATCCACTTTGAACCCTTTTTGCTGATGACTGT | chr.7(-): 66812655- 66812733 | SNORD68A RNA (its second antisense element) can guide the modification of a neighboring site in rRNA. The nucleotides complementary to the SNORD68 RNA targets are shown in red. |
| zebrafish | SNORD68B | TTGCAGTCAGATGATGACGGCTTTCTCCGGAATCTCTGCACATCCTTGATGACTTTTGAACCCTTTTCTGGCTGATGACTGCAG | chr.7(-): 66810617- 66810700 |  |
| zebrafish | SNORD68C | ACAGTCAAATGATGACAATTCTCCGGAATCTCTGCACTTCCTTGATGATCCACTTTGAACCCTTTTTGCTGATGACTGT | chr.7(-): 66807863- 66807941 | SNORD68C RNA (its second antisense element) can guide the modification of a neighboring site in rRNA. |
| fugu | SNORD68A | AATGCAGCAGTCAAGTGATGACAAATCTCCGGAATCTCTGTATTTACTTGATGAATACTTGAACCCATTAATCTGATACTGACTGCATATT | Un. (+): 46509682- 46509772 | The host gene has two copies.  The second antisense element in SNORD68A and SNORD68C genes is disrupted.  SNORD68B and SNORD68D RNAs (their second antisense element) can guide the modification of a neighboring site in rRNA. |
| fugu | SNORD68B | AGCAGTCAGATGATGACAAATCTCCGGAATCTCTGTATTTTCTTTTGATGAAACTGTTGAACCCTTTTTGCTGATGACTGCT | Un. (+): 46510489- 46510570 |
| fugu | SNORD68C | AATGCAGCAGTCAAGTGATGACAAATCTCCGGAATCTCTGTATTTACTTGATGAATACTTGAACCCATTAATCTGATACTGACTGCATATT | Un. (+): 46518692- 46518782 |
| fugu | SNORD68D | AGCAGTCAGATGATGACAAATCTCCGGAATCTCTGTATTTTCTTTTGATGAAACTGTTGAACCCTTTTTGCTGATGACTGCT | Un. (+): 46519501- 46519582 |
| tetraodon | SNORD68B | AGCAGTCAGATGATGACAAATCTCCGGAATCTCTGTACTTGCCTTTGATGAAACCCCTGAACCCTTTTGCTGATGGCTGCT | chr.5(-): 6064080- 6064160 |  |
| stickle-  back | SNORD68B | GCAGTCAGATGATGACAAAACTCCGGAATCTCTGTATTTCTTTGATGAAACACTTGAACCCTTTTTTCTGATGACTGC | chr.II (+): 10573505- 10573582 |  |
| medaka | SNORD68B | AGCAGTCATGTGATGACACATCTCCGGAATCTCTGTAGTTCTTTGATGAAAAACTTGAACCTTTTTCTGATGACTGCT | chr.3(+): 18940221- 18940298 |  |
| mouse | SNORD69 | AACATGAAGCAGTGATGACAGATTGGATCTGACTTAACTGCTGTGCTGAGTTTGTTCAATCCAACCCTGAGCTTCATGTT | chr.14(-): 31827473- 31827552 |  |
| dog | SNORD69 | AACATGAAGCAAATGATGATAAACTGGATCTGACAGACTGTGCTGAGTTTGTTCAATCCAACCCTGAGCTTCGTGTT | chr.20(-): 40100602- 40100678 |  |
| cow | SNORD69 | AACATGAAGCAAATGATGATAAACTGGATCTGACAGACTGTGCTGAGTTTGTTCAATCCAACCCTGAGCTTCGTGTT | chr.22 (-): 49217564- 49217640 |  |
| opossum | SNORD69 | ACATGAAGCATATGATGACAAACTGGATCTGATATTCTATGTTGAATTTCTTCAATCCAACCCTGAGCTTCATGT | chr.6(-): 229001543- 229001617 |  |
| platypus | SNORD69 | AAACATAAGCCTATGATGAAAGTTGGAACTGACATACCATGCTGAGTTTGTTCAATCCAACCCTGAGCTTCATGTTT | contig 1718 (-):52115- 52191 |  |
| chicken | SNORD69A | TAGCCTGTGCAGGGATGATGGGGTAGCCTGCATAACATCACCCTTCGTACCACACAGAGTCTCCTTGTGATACTCCAACACTGAAAACACAGGGTA | chr.12(+): 736330- 736425 |  |
| chicken | SNORD69B | AGCCCAAAGCACATGATGAGGAAGCACTGAAACTGACTCTGTATGAAGTGCATTCAATCCAATGCTGAGCTTTGTGGCT | chr.12(+): 738668- 738746 |  |
| zebrafish | SNORD69A | AAGCATGATGAACTGTATTGAACTGAACACAACATGACGTCTACATTCAATCCAACACTGAGCTT | chr.11(+): 2665358- 2665422 |  |
| zebrafish | SNORD69B | AGAGAAATGATGAGTTTGGTGATCACATAATTCCTTGATGACCATCTAATCCAACTCTGATCTCT | chr.11(+): 2668280- 2668344 |  |
| tetraodon | SNORD69A | CTTCATTTTGGACATGAGGACAAAAAAGAATTTCTGGATTTCTTTCTGAAGACGTTTAATCCAACTCTGATCCAAGAGGAG | chr.11 (+): 8454759- 8454839 |  |
| tetraodon | SNORD69B | GCTGGAAATGAGGACAAAACTGCAAGTCTTGATTTATTTATGAAGACTTCTAATCCAACTCTGATCCAGC | chr.11 (+): 8455291- 8455360 |  |
| human | SNORD70B | ATTCAATGTTGTCAATGATGCATTCTTATTGGAACTGAATTTAAGTGATCTGACTCATTCGTCACTACCACTGAGACAACATTGAGT | chr.2 (+): 202851075- 202851161 |  |
| mouse | SNORD70A | TTCATTGTTGTCAATGATGTATACCTTTTGGAACTGAATCTAAGTGATTTAACAAAAATTCGTCACTACCACTGAGACAACAATGAA | chr.1(+): 59748778- 59748864 |  |
| mouse | SNORD70B | CTCAATGTTGTCAATGATGCATCTTATTGGAACTGAATTTAAGTGATCTAACCCATTCGTCACTACCACTGAGACAACATTGAG | chr.1(+): 59749353- 59749436 |  |
| opossum | SNORD70A | TGTTGTCAGTGATGTACATATGTTGGAACTGAATCTTAAGTGATATCATTTACCAATTCGTCACTACCACTGAGACAACA | chr.7 (-): 195895150- 195895229 |  |
| opossum | SNORD70B | TTTCCCTTATTGTTGTCAGTGATGGATTTTGTTGAATGAAAAACAAGTGATTTGACATTCGTCACTACCACTGAGACAACAGGGAAA | chr. 7 (-): 195883529-195883615 |  |
| platypus | SNORD70A | TGTTGTCAATGATGCATTCATGTTGAAACTGAATCCAAGTGATTTAACTGAACAATTCGTCACTACCACTGAGACAACA | Contig 10764 (+): 835- 913 |  |
| platypus | SNORD70B | TGTTGTCAATGATGCATTCTTGTTGGAACTGAATCAAAATGATTTAATTCAACAATTCGTCACTACCACTGAGACAACA | Contig 10764 (+):2794-2872 |  |
| chicken | SNORD70A | TGTTGTCAATGATGGATACCTGTTGGAACTGAATGTAGCTGATTTGAGTCAACATTTCGTCCCTACCACTGAGACAACA | chr.7 (+): 12843055- 12843133 |  |
| chicken | SNORD70B | ATTCCCAGACTGTTGTCAATGATGAAACATAGTTAAGCTGAAAATAATGATATAATTCAGTTCCTTCGTCACTACCACTGAGACAACAGATGGGAAT | chr.7 (+): 12847692- 12847788 |  |
| frog | SNORD70A | TATTGCCAATGATGGATCCTTTGTTGAACTGATTACTTGTGATTTCATTCAACAATTCGTCACTACCACTGAGGCAATA | scaff.312 (+): 43624- 43702 |  |
| frog | SNORD70B | TGTCACCTATGATGGATAAATGTTGATCTGAAACAAAATGATTTTAGATTCAACTCTTCGTCACTACCACTGAGGTGATG | scaff.312 (+): 44082- 44161 |  |
| zebrafish | SNORD70A | ATCTGTGCGTCTATGATGTTTCTGTTCACCCAATTCAACCATGAGGTCACCAAACAGTTATTCGTCACTACCACTGAGACCACAAT | chr.6 (-): 7943390- 7943475 | The host gene has two copies. |
| zebrafish | SNORD70B | TGCTCATGTCGATGATTATTTTGTGCTGACTGACTTTTCTGACGATATTCTTCAGCCTTCGTCACTACCACTGAGACTCTGAGCA | chr.6(-): 7941755- 7941839 |
| zebrafish | SNORD70C | ATCTGTGCGTCTATGATGTTTCTGTTCACCCAATTCAACCATGAGGTCACCAAACAGTTATTCGTCACTACCACTGAGACCACAAT | chr.6 (-): 8042144- 8042229 |
| zebrafish | SNORD70D | TGCTCATGTCGATGATTATTTTGTGCTGACTGACTTTTCTGACGATATTCTTCAGCCTTCGTCACTACCACTGAGACTCTGAGCA | chr.6(-): 8040507- 8040591 |
| tetraodon | SNORD70A | GTGTGGTCGATGATGCAGTTATGTGTCTGATTAAAACATGAGGCAGTTCACATTTCTCGTCACTACCACTGAGACCACAT | chr.3(-): 3411904- 3411983 |  |
| tetraodon | SNORD70B | TGCCAATGATTACACTTGCTGCCCGAAACATTTGTGTGGATTTATTTCAGCATTCGTCACTACCACTGAGGCA | chr.3(-): 3411297- 3411369 |  |
| mouse | SNORD71 | ACGTGTGCTGGAGGATGAAGTAAGGAGTGATCCATCGGCTAAGTGTTTTCCCACTGTGCTGACACTCACATTGCTGACAGTGCACGT | chr.8 (+): 112363194- 112363280 |  |
| opossum | SNORD71 | TGTGTGTTGGAGGATGAAAACTTTGAGTGATCCATCGGCTGAGTGATTTGTCACAATGCTGAGACTCAAGTTGCTGACAGCACACA | chr. 1 (-): 658458800- 658458885 |  |
| platypus | SNORD71 | GTGTGTTGGAGGATGAAAACTTTGAGTGATCCATCGGCTAAGTGACCTGTCACAGTGCTGAGACTCAAAACTGCTGACAACACAC | chr.11 (-): 2438209- 2438293 |  |
| chicken | SNORD71 | AGTGCTGTTGGAGGATGAAGCCTTTGAGTGATCCATCGGCAGAGCTTGATGTTGCTGTGCTGACACTCGTGCTGCTGACAATGCACT | chr.11 (+): 21753369- 21753455 |  |
| lizard | SNORD71 | TGTGCTCTTGGAGGATGAGGCCTGAGAGTGATCCATCGGCAGAGTGCCCAGTGCAATGGTGAAACTCATGCAGCTGACGGAGCACA | scaff. 661 (-): 307284- 307369 |  |
| frog | SNORD71 | TTGTTGTGTGATGATTATTAAGTGATCCATCGGCAGAGACTTACCGTGCTGAGATCCTAAAACCTGACAACAA | scaff.66 (+): 3121685- 3121757 |  |
| zebrafish | SNORD71A | ATAGAATGATGAAGACTTTGAGTGATCCATCGACTGAGTTTTAAATGATGACCTCATTGTTTCTGACTAT | chr.7(+): 69234017- 69234086 |  |
| zebrafish | SNORD71B | AGTGGCTGTGTGATGATAAAATGAGTGATCCATCGGCAGAAGATGATTCTGTTGACACTTTTATATCTGACAGCACT | chr.7 (+): 69235242- 69235318 |  |
| tetraodon | SNORD71 | ATGCTGCTTGATGATAAAAAGAGTGATCCATCGTCTGAAGGACTGTGATGATTCCATTTAGTTTCTGACAGCAT | chr.5 (+): 5594011- 5594084 |  |
| mouse | SNORD72 | taataGCTTATCAATGATGTCCTAAAAAATAAATGTCTGAACATATGACTGCTATAATGATTTCAGCATTTAACTGAGATAAGCTcatta | chr.15 (+): 5068400- 5068489 |  |
| cow | SNORD72 | AGCTTATCGGTGATGTTCTAAAAAAATAAATGTCTGAACTTATGAATTCAATATTGATTTCATCATCTAACTGAGATAAGCT | chr.20 (+): 35788257- 35788338 |  |
| opossum | SNORD72 | AATATTTGCAGCTTGTCAGTGATGTACTTTGAAAAAAATGTCTGAACATATGAATTAATAGTGATTTCAGCATTTAACTGAGATGAGCTGAGTAAAATGTT | chr.3 (+): 234018942- 234019042 |  |
| platypus | SNORD72 | GCTTATCGATGATGTACTCGAAAAAAAATGTCTGAACATATGAGTCCGCTAGTGATTTCAACGCGCACTGAGATAAGC | Contig 6062 (-): 27469- 27546 |  |
| chicken | SNORD72 | CAGCTTGTCAATGATGTTTCTTACTAAAATGTCTGAACATATGAACTTCATAGTGATTTCAGTGCATTACTGAGATAAGCTG | chr.Z (-): 12332125- 12332206 |  |
| lizard | SNORD72 | GTCAGCTTATCCATGATGTCTTTATTACAAAAAATGTCTGAACATATGAGTTCTAGTGATTTTACATTGTAACTGAGATAAGCTGAC | scaff.3(-): 4780029 - 4780115 |  |
| frog | SNORD72A | GCATTTATCAGTGATGTCTTATAATAAATGTCTGAACATATGACACTTAGTGATTTCAGTTTTCTAACTGAGATAAATGC | scaff.418 (+): 288818- 288897; scaff.14166 (-): 6591-6670 | Scaffold 418 and short scaffold 14166 are probably represent the same genomic locus. |
| frog | SNORD72B | CTTATCCATGATGTTTAAAAATAAATGTCTGAACTTATGAATTAATTGTGATTCAGATTTCTAACTGAGATAAG | scaff.418 (+): 292673- 292746 |  |
| zebrafish | SNORD72A | GCTTACCAGTGATGTGCAAAAATAAATGTCTGAACAAATGAGTGTCCAGTGATAACTGATTTTGAACTGAGGTAAGC | chr.21(-): 1638737- 1638813 |  |
| zebrafish | SNORD72B | GGCTTACCAGTGATGTTCAAAAATAAATGTCTGAACAAATGACTGTCCAGTGATAACAGATTTGTACTGAGGTAAGCC | chr.21(-): 1637581- 1637658 |  |
| tetraodon | SNORD72A | GTCTTGTCAGTGATGTTAATCATAAATGTCCGAACATATGACTTATTGTGATTCATTTCAATGCACTGAGACAAGAC | chr. 4 (+): 3989056- 3989132 |  |
| tetraodon | SNORD72B | GCTCACCCATGATGGACCTAGAAAAATGTCCGAACAGATGAATAAAATGTGAAATCATTCTTGTCTCTGAGGTGAG | chr.4 (+): 3989820- 3989895 |  |
| mouse | SNORD73A | TGCATGGGAACATGTGATGAGAAACTGTTTCGGTCCCAGTTGATGGCCACTGATAACATTACATTTTTCTGATGTTCCCATGCA | chr.3(-): 85942705- 85942788 |  |
| mouse | SNORD73B | GTATATGGGAATGAGTGATGACAAGATGTTTCGGTCCCAAATGAAGCCTACTGATTACCGTAACATTTATTGCTGACATTCCCTTATAC | chr.3(-): 85944531- 85944619 |  |
| dog | SNORD73A | TGTATGGGAATAGATGATGACAAAATGTTTCGGTCCCAGATGACAACCACTGATAAAACTTAACATTTTTCTGATGTTCCCATGCA | chr.15(+): 52081705- 52081790 |  |
| dog | SNORD73B | GGGAATGAGTGATGACAAAATGTTTCGGTCCCAAATGATACATATTGATTATACCGCTACATTTACTTCTGACATTCCC | chr.15(+): 52080442- 52080520 |  |
| cow | SNORD73A | GTATGGGAATAAATGATGACAAATGTTTCGGTCCCATATGATGTCTGATGATAAAACTTAACATTTTTCTGATGTTCCCATGC | chr.17 (-): 7221107- 7221189 |  |
| cow | SNORD73B | GGGAATGAATGATGACAAAATGTTTCGGTCCCAAATGACATAATGATTATACCGTTACATTTCTGACATTCCC | chr.17 (-): 7222312- 7222384 |  |
| opossum | SNORD73A | GGAATGAATGATGACAAAATGTTTCGGTCCCAAATGATACATAATGATTATACCATTGCATTTTTTCTGACATTCC | chr.5 (-): 126921943- 126922018 |  |
| opossum | SNORD73B | GGAATAAATGATGAAAAATGTTTCGGTCCCAATTGATATCTAATGATAATACATTGTTCTGATATTCT | chr.5 (-): 126920194- 126920261 |  |
| platypus | SNORD73A | CGTGGGAACGGGTGATGACCACATGTTTCGGTCCCAGATGACGCCGTGTGATTTCACCGTTGCGATTTTCTGACGTTCCCACG | Contig 819 (-): 313833- 313915 |  |
| platypus | SNORD73B | GGGATACGTGATGACAAGATGTTTCGGTCCCAGATGACGTCCCGTGATTTGACTGTTGCATTTTTCTGACGTCCC | Contig 819 (-): 314989- 315063 |  |
| chicken | SNORD73 | GTTTGGGAATGAATGATGACAAATTGTTTCGGTCCCAGATGACACGAAATGATTTTAATTTTCCCATTTTCTGACATTCCCATAC | chr.4 (+): 34371653- 34371737 |  |
| lizard | SNORD73 | GTATGGGAATGAATGATGACACCTTGTTTCGGTCCCATATGAGTCAGTGATTATGCTTTTCTAATGCTGACATTCCCATAC | scaff.122 (-):922735- 922815 |  |
| frog | SNORD73A | TGGAATGAATGATGACAAAATGTTTCGGTCCCAAATGATTTCTATGATTACTCTTTGAGATCGTTCTGACATTCCA | scaff.60 (-): 2451823- 2451898 |  |
| frog | SNORD73B | GGGAATGAATGATGACAATATGTTTCGGTCCCAAATGATCATCTGTGATTATTGCTTTGAGATCGTTCTGACTTTCC | scaff.60 (-): 2448921- 2448997 |  |
| frog | SNORD73C | GGAAACATGATGACAAACTGTTTCGGTCCCATATGAGTTCTTGTGATTACTGACATGAGATCGTTCTGATGTTCC | scaff.60 (-): 2447649- 2447723 |  |
| mouse | SNORD74 | GCGTGTTCATGCTATGATGAAGGCTATGTTGGTAGGGACAACTGAGCTTGTTGATGAATACCAACGATTCTGATGGCAGAGCATGC | chr.1(+): 162965523- 162965608 |  |
| rat | SNORD74 | GTGACTCTTGCAATGATGAAGGTTATGTTGGTAGGGACAACTGACCTTGTTGATGAATACCAACAATTCTGATGGCAGAGCAT | chr.13(+): 76594999- 76595081 |  |
| dog | SNORD74 | CTGCCTGTGATGAAGCCTGTGTTGGTAGGGACATCTGAGACTGTTGATGAATGCCAACGGCTCTGATGGCGG | chr.7(+): 28329367- 28329438 |  |
| opossum | SNORD74 | TTCAGGCCTTGCTGTGTGTGATGAAAGTAACGTTGGTAGGGACATCTGAGATTGCTGATGAATGCCAACGTCTCTGATGCGGGGCCTGGG | chr.2(-): 66320909- 66320998 |  |
| platypus | SNORD74 | GTCCTGCCCCTACCTGTGATGAAAACAAGTGTTGGTAGGGACATCTGAGAAAGCTGATGAATGCCAACACAGCTGAGTCGGGGCTTGGAC | Ultra 341 (-): 3117475- 3117564;  Contig 288115 (+): 317 - 406 | Very short contig (#288115) contains a fragment of the SNORD74 RNA host gene (gas5) which includes the SNORD74 sequence. Until the final assembly of the platypus genome become available it remains unclear wether SNORD74 RNA has one or two genes. |
| chicken | SNORD74 | ATGTGCCCGCACATGATGAAAACTATGTTGGTAGGGACATCTGAGAGGCTGATGAGTACCAACATATCTGAGCTGGGCATGT | chr.8(+): 7729343- 7729424 |  |
| lizard | SNORD74 | CCTCTCCCCTGCACATGATGATAAATGATGTTGGTAGGGACATCTGAGAATATGATGAATACCAACATCTCTGAGCCAGGGGTAGG | scaff.169 (+): 2593690- 2593775 |  |
| frog | SNORD74 | GGCATGCCTGAGAATGATGAAAATTATGTTGGTAGGGACATCTGAGAGCAGTGATGATTACCAACGTCTCTGATCAGGCCATGTC | scaff.1(+): 6870669- 6870753 |  |
| zebrafish | SNORD74 | TGTTTACTTGTATGATGAAGAATATGTTGGTAGGGACATCTGAGAAAATCTGATGAATGCCAACATTTCTGACATGTAGCA | chr.8(-): 13997233- 13997313 |  |
| fugu | SNORD74 | TCCCATGCGCACATGATGAAATAAGTGTTGGTAGGGACATCTGAGTTAAGGTGAAGAAATGCCAACTCTTCTGACTTCATGGGA | Un. (-): 193908961- 193909044 |  |
| mouse | SNORD75 | AAGCCCATGATGGTATGAGAGTAGTGGACAGAAGGGATTTCTGAAAAACACTTTTCTGAGGCTTT | chr.1(+): 162965980- 162966044 |  |
| rat | SNORD75 | AAAGCCCATGATGGTATGAGAGTAGTGGACAGAAGGGATTTCTGAAAAACACTGTTCTGAGGCTTT | chr.13(+): 76595482- 76595547 |  |
| dog | SNORD75 | TGTTTAGCCTGTGATGGTTTAAGAGTAGTGGACAGAAGGGATTTCTGAAAAGTTTTACTCTGAGGCTGAAATA | chr.7(+): 28329848- 28329920 |  |
| opossum | SNORD75 | GCTATTCAGCCTATGATGTCCTAAGAGTAGTGGACAGAAATGATTTCTGACAATAATATACTGAGGCTTTAATAGT | chr.2(-): 66320140- 66320215 |  |
| platypus | SNORD75A | CGAGGCAGACAAGCCTATGATGGTTTTAAGTATAAGAGTAGTGGACAGATGTGATTTCTGACAACTGACTACTGAGGCTTTGTGATTTG | Ultra 341 (-): 3116766- 3116854 |  |
| platypus | SNORD75B | GCAAAGCCAGTGATGACTTTAAATGATAAGAGTAGTGGACAGAAGAGATTTCTGAATACTTGAAACGCTGAGGCTTTGT | Ultra 341 (-): 3114496- 3114574 |  |
| chicken | SNORD75 | GGAGAGCCTGTGATGTTTTCAGTATTAGAGTAGTGGACAGAAGTGATTTCTGAAAACTCTGTGCTGAGGCTTTTT | chr.8(+): 7730160- 7730234 |  |
| lizard | SNORD75 | AAAAGCCAATGATGTTTCTAAGAGTAGTGGACAGAAGAATTTTCTGACATATATTGCACTGAGGCTTTT | scaff.169 (+): 2600384- 2600452 |  |
| frog | SNORD75 | TTAAGCCAGTGATGCTTCAAAGAGTAGTGGACAGGTGTGATTTCTGACAGCTTGTAAAACTGAGGCTTAA | scaff.1(+): 6875263- 6875332 |  |
| zebrafish | SNORD75A | TGCCTTTGATGAATTAAAAATGTATAAGAGTAGTGGACAGAAGTGATTTCTGAGAACATTTTTAATCTGAGGCA | chr.8(-): 13997012- 13997085 |  |
| zebrafish | SNORD75B | TGCCTATGATGAATAAAAATGTATAAGAGTAGTGGACAGGAGTGATTTCTGATAATTTTTACTCTGAGGCA | chr.8(-): 13996752- 13996822 |  |
| fugu | SNORD75 | AGTCTGTGATGATAGAGATAAGAGTAGTGGACAGAAGAGATTTCTGAACAAAAGTATGCTGAGACT | Un. (-): 193908550- 193908615 |  |
| mouse | SNORD76 | GTGCCACAATGATGACATCATATTTGCTACTCTTGACAGCTGGGGTGACGATAGCTTTAAACCACCATGATTATAACTGAGGCAC | chr.1(+): 162966187- 162966271 |  |
| rat | SNORD76 | GTGCCACAATGATGACATCATTTGCTACTCTTGACAGTTGGGGTGACGATAGCTATAGACCACCATTAATCATAACTGAGGCAC | chr.13(+): 76595694- 76595777 |  |
| dog | SNORD76 | TGCAATGATGACATTTTATTTGCTACTCTTGACTGTGAGAATGACGAGAATTATTACCACCGTTAAACTAACTGAGGCA | chr.7(+): 28330068- 28330146 |  |
| opossum | SNORD76 | GTGCTTTAATGATGAACTTTTATTTGCTACTCTTGATCATGAGTATGATGAGAAATACTACCACCTTTCTCTTATACTGAAGCAC | chr.2(-): 66319843- 66319927 |  |
| platypus | SNORD76 | GAGTGCCGCAATGATGACTCTTTATTTGCTACTCTTGACCATGAGAATGACGAGAGCTATTACCACCATTTTGATACTGAGGCACTT | Ultra 341 (-): 3116474- 3116560 |  |
| chicken | SNORD76 | CTGGTGCCACAATGATGACTGTATAGTTTGCTACTCTTGACCAAGAAAATGATGAGAGCTCAAACCACCATAATTTCTATCTGAGGCACAG | chr.8 (+): 7730463- 7730553 |  |
| lizard | SNORD76 | TGTTGTGCTGCAATGATGACCATTATTTGCTACTCTTGATCAAGAGGATGATGAGAATCTATTACCACTTTCATTTTAAAACTGAGGCACAATA | scaff.169 (+): 2595755- 2595848 |  |
| frog | SNORD76 | ATGCCACAATGATGATTCTTATTTGCTACTCTTGACTGCAGGAGTGATGAGAGTGCAAACCACCTTTTATGTATCTGAGGCAT | scaff.1(+): 6871749- 6871831 |  |
| zebrafish | SNORD76 | GTGCATATGATGACCCTATTTGCTACTCTTGATTTCATGCTGATGAAATTGCAACCACCAAAATACCTTGTCTGAAGCAC | chr.8(-): 13996382- 13996461 |  |
| fugu | SNORD76 | TGTGCTGCTGTGATGACATCATATTTGCTACTCTTGACAAAATGAATGACGAGACATCTCCCACCTTATGGAAACTGAGCCACA | Un. (-): 193908145- 193908228 |  |
| dog | SNORD77 | CAGATACTTATGATGGTTGCATAGTTCAGCAGATTGGATCATGAAGAGAGACACCATTTGTCTGATGTATCTG | chr.7(+): 28330354- 28330426 |  |
| opossum | SNORD77 | AAGCAGATACTATGATGGTTGCATAGTTCAGCAGATATTAGTGAAGAATACTTATCTTATTATCTGATGTATCTGACTT | chr.2(-): 66319614- 66319692 |  |
| platypus | SNORD77 | GCCGATACAGTGATGCTTGCATAGTTCAGCAGATGGAACAGTGACGAAATATACCACATAATCTGATGTATCTGGC | Ultra 341 (-): 3116194- 3116269 |  |
| mouse | SNORD78 | GGGTTCTTGTAATGATGTTGATCAAATGTCTGAGCTGAAAATAACTTGTAGACAATTTTTAACACTGAAGAACCT | chr.1(+): 162967109- 162967183 |  |
| rat | SNORD78 | ACGGGGTTTTTGTAATGATGTTGATCAAATGTCTGAGCTGAAAATAACTTGTAGACAATTTTTAACACTGAAGAACCCTGT | chr.13(+): 76596592- 76596672 |  |
| dog | SNORD78 | ACGGGGTTTTTGAAATGATGTTGATCAAATGTCTGACCTGAAATGACCATGTAGACAAATTTAACACTGAAGAACCCTGT | chr.7(+): 28331025- 28331104 |  |
| opossum | SNORD78 | ACAGGGTTTATAATGATGTCCTCAAATGTCTGACCTGAAATCAGCATGTAGATAAATCACAGCACTGAAGTGCCCTGT | chr.2(-): 66318502- 66318579 |  |
| platypus | SNORD78 | ACAGGGTTTACAATGATGTCTTCAAATGTCTGACCTGAAAGTCTATGTAGACAAGCTTGCTTCTGAAAGACCCTGT | Ultra 341 (-): 3114956- 3115031 |  |
| chicken | SNORD78 | TACAGGGTTGTAATGATGTTGTCAAATGTCTGACCTGAAACTACTAATGTAGATTTCTTTTTTTTACTGAAAAACCCTGTA | chr.8 (+): 7732717- 7732797 |  |
| lizard | SNORD78 | GCAGGGTTTAAAATGATGATTAAATGTCTGACCTGAAATCACATGTGGATTCTTTGCACTGAAAACCCTGT | scaff.169 (+): 2599383- 2599453 |  |
| frog | SNORD78 | CAGGGTTTGTATGATGTCTCAAATGTCTGACCTGAAGTCAATGTGTGGACAGAGTATAAACACTGACTCGACCCTG | scaff.1(+): 6873975- 6874050 |  |
| zebrafish | SNORD78 | TTTGGGTTTTGTGATGCTCTACAAATGTCTGACCTGAATGCACAGTGTGGATTTATCTTTGACTGAAACTCAAG | chr.8(-): 13994663- 13994736 |  |
| mouse | SNORD79 | AGGTACTGTTAGTGATGATCAATAAAGTTAAACAGATGGGAATCTCTCTGAATAAGATTGAAGATTGATTGTTAAGCTGAAACAGTATTT | chr.1(+): 162967484- 162967573 |  |
| rat | SNORD79 | GGGTACTGTTAGTGATGATCAATTAAGTTAAAACAGATGGGAATCTCTCTGAACAACATTGGAGATTGATTGTTAAGCTGAAACAGTATTT | chr.13(+): 76596977- 76597067 |  |
| dog | SNORD79 | AATGCTGTTAGTGATGATTTTTGAAATAGATGGGAATCTCTCTGAAAAAGAGTGAAGATTTTTAAAAACTGAAACAGTATT | chr.7(+): 28331372- 28331452 |  |
| opossum | SNORD79 | TGTACTGTTGGTGATGATTTCTATATTAAAACAATGGGAATCTCTCTGAAAGAGAATGAGGATTTTCTTTTTCTGAAACAGTACA | chr.2(-): 66318046- 66318130 |  |
| platypus | SNORD79 | CTGTGCTGTTCATGATGATTTGTTAAAATCAATGGGAATCTCTCTGAAGAGAATGAAGCCAGTTTTTATACTGAAACAGTACAG | Ultra 341 (-): 3113986- 3114069;  Contig 37762 (+): 203-286 | Very short contig (contig 37762)contains a fragment of the SNORD79 RNA host gene (gas5) which includes the SNORD79 sequence. Until the final assembly of the platypus genome become available it remains unclear wether SNORD79 RNA has one or two genes. |
| chicken | SNORD79 | TGTACTGTTCATGATGATTAGTTCTACACAATGGGAATCTCTCTGAAAGAGACTGAGGAGGACTCTTGATCTGAAACAGTACA | chr.8(+): 7733659- 7733741 |  |
| lizard | SNORD79 | GTACTGTTAATGATGATATTGTATGAAACAATGGGAATCTCTCTGAAAGTTAATGATGAAAGAATTACACTGAAACAGTAC | scaff.169 (+): 2601883- 2601963  scaff.5343 (-):4665- 4745 | The second search hit has several nucleotide substitutions, is located in the short scaffold (#5343) and is probably a fragment of gas5 pseudogene. |
| frog | SNORD79A | CTGTGCTGTGTATGATGATTTGGAAATATCAATGGGAATCTCTCTGAACAATAATGAGGGAACTCTTTACACTGAATCAGTACAG | scaff.1(+): 6872291- 6872375 |  |
| frog | SNORD79B | CATTGCAGTATGTGATGATTTTTTGTTTTTGCAATGGGAATCTCTCTGAACCCCATGATAGAAAAGCTAAAACTGATACAATGCATG | scaff.1(+): 6875496- 6875582 |  |
| zebrafish | SNORD79A | ACTGTTTATGATGATTGTTAAATGAAACAATGGGAATCTCTCTGAATTAGACTGAAAGAGACATCTTTATGCTGAAACAGT | chr.8(-): 13996140- 13996220 |  |
| zebrafish | SNORD79B | TGCTGTTCATGATGACTGTTTTATAACATTGGGAATCTCTCTGAATAAGACTGAAAGACCTCATTAATAAACTGAAACAGTA | chr.8(-): 13995870- 13995951 |  |
| fugu | SNORD79 | GCTGTTAATGATGATCTTGTTTTAAAAACAATGGGAATCTCTCTGAATAAGAGTGAAAGATGACTTTCAACTGACACAGT | Un. (-): 193907914- 193907993 |  |
| mouse | SNORD80 | AGCCGATACCATGATGATAACATAGTTCAGCAGACTTAACTCTGATGAACAATCATGTCTTTCGCTCCTATCTGATGTATCTGGCT | chr.1(+): 162967730- 162967815 |  |
| rat | SNORD80 | AGCCGATACTGTGATGATAACATAGTTCAGCAGACGTGACTCTGGTGAACAATCATGTCTTTCGCTCCTATCTGATGTATCTGGCT | chr.13(+): 76597228- 76597313 |  |
| dog | SNORD80 | AGCTGATACGATGATGAAGCCATAGTTCAGCTGACTAGCAGTGATGAGCAAAATTGTCTTTCGCTCCTATCTGACGTATCGGCT | chr.7 (+): 28331623- 28331706 |  |
| opossum | SNORD80 | AGCTGATACAATGATGATAACATAGTTCAGCAGATGTATGGTGGTGAACAACACTATTCTGTCTTTCGCTCCTATCTGATGTATCTGCT | chr.2(-): 66317746- 66317834 |  |
| platypus | SNORD80 | AAGCCGATACAATGATGATAACATAGTTCAGCAGATGAAAAGTGATGAACTCTCATTTTTGTCTTTCGCTCCTATCTGATGTATCTGGCTT | Ultra 341 (-): 3113749- 3113839;  Contig 37762 (+): 433-523 | Very short contig (contig 37762)contains a fragment of the SNORD80 RNA host gene (gas5) which includes the SNORD80 sequence. Until the final assembly of the platypus genome become available it remains unclear wether SNORD80 RNA has one or two genes. |
| chicken | SNORD80A | AGCAGATACAGTGATGATAACATAGTTCAGCAGATTATCTCGTGATGAACTATGTTCTGTCTTTCGCTCCTATCTGATGTATCTAGCT | chr.8 (+): 7730900- 7730987 |  |
| chicken | SNORD80B | AGGCTGTGTATGATGACAACATAGTTCAGCAGATAACTTGTGATGATCACTCTACTGGTCTTTCGCTCCTATCTGATGCAGCCT | chr.8 (+): 7731153- 7731236 |  |
| lizard | SNORD80A | AAGGATGTGTATGATGATGGCATAGTTCATCAGATACTCTGTGATGATTGCTGATGTCTTTCGCTCCTATCTGACGCATCCTT | scaff.169 (+): 2596171- 2596253 |  |
| lizard | SNORD80B | CAGATACAATGATGATCACATAGTTCAGCAGATGGAACAGTGCTGATCTGTCTTTCGCTCCTATCTGATGTATCTG | scaff.169 (+): 2602243- 2602318  scaff. 5343(-): 4310-4385 | The second search hit is located in the short scaffold (#5343) and is probably a fragment of gas5 pseudogene. |
| frog | SNORD80A | GTTTGACACAAATGATGAACACATAGTTCAGCAGATGAACCCTGATGAACTTGTCTTTCGCTCCTATCTGATGTGTCAGAT | scaff.1 + 6872685- 6872765 |  |
| frog | SNORD80B | GAAGGCTAGAACTTTGAATACAATGATGACTGCATAGTTCATCAGACTAGCAATGGTGAACAATCTTTCGCTCCTATCTGATATATCAAATGGCTAGCCTTC | scaff.1(+): 6875742- 6875843 |  |
| frog | SNORD80C | AAGATGCATGTGATGAAAACATAGTTCAGCTGACCTTTTGTGAAGATCAGTCTTTCGCTCCTATCTGATGTATCTT | scaff.1(+): 6876039- 6876114 |  |
| zebrafish | SNORD80A | GTGATGCAAATGATGACTGCATAGTTCAGCAGAATTTCCATGTAGAACACAGAGCAATGTCTTTCGCTCCTAGCTGATGCATCAC | chr.8 (-): 13995531- 13995615 |  |
| zebrafish | SNORD80B | GTGATGCAGATGATGACTGCATAGTTCAGCAGAAACTCCATGTAGAACACAGAGCAATGTCTTTCGCTCCTAGCTGATGCATCAC | chr.8(-): 13995317- 13995401 |  |
| fugu | SNORD80A | GTGATGCAGATGATGACTGCATAGTTCAGCAGAAACTCCATGTAGAACACAGAGCAATGTCTTTCGCTCCTAGCTGATGCATCAC | Un. (-): 193907708- 193907773 |  |
| fugu | SNORD80B | AGTCTGATGCAATGATGAGCCCATAGTTCAGCAGAGAAATCATGAAGAATAATTCTTTCGCTCCTATCTGATGTGTCACACT | Un. (-): 193907487- 193907568 |  |
| mouse | SNORD81 | CAGAATACATGATGATCTCACACAACTTGAACTCTCTCACTGATTACTTGATGATAGTAAAAGATCTGATGTTCTG | chr.1(+): 162968439- 162968514 |  |
| rat | SNORD81 | TACAGAATACATGATGATCTCACTCCAACTTGAACTCTCTCACTGATTACTTGATGAAAGTAAAAGATCTGATGTTCTGTA | chr.13(+): 76597868- 76597948 |  |
| dog | SNORD81 | CAGAATACATGATGATCTCATTGCAACTTGAACTCTCTCACTGATCATTTGATGATTTTAAAAGATCTGATATTCTG | chr.7(+): 28332508- 28332584 |  |
| opossum | SNORD81 | TTAAGAAAATGATGACAATCATCCTTTACTTGAACTCTCTCACTGATAAAGTGATGAGAAACTTAAGGTCTGATTTTGA | chr.2 (-): 66316672- 66316750 |  |
| platypus | SNORD81 | TGGGTACAATCCACAAATGATGACCACATCCTTAATTGAACTCTCTCACTGATATTTGATGAGAATCTAAGGTCTGATGCAACCTA | Ultra 341 (-): 3112919- 3113004  Contig 37762(+): 1289- 1374 | Very short contig (contig 37762)contains a fragment of the SNORD81 RNA host gene (gas5) which includes the SNORD81 sequence. Until the final assembly of the platypus genome become available it remains unclear wether SNORD81 RNA has one or two genes. |
| chicken | SNORD81 | TTGCTGGAACAAATGATGATTAAAACCTTAGCTTGAACTCTCTCACTGAACAGAGATGAAAACCTAAGGTCTGAGTTGTTCCAACAA | chr.8(+): 7734998- 7735084 |  |
| lizard | SNORD81 | GAATAGATTAATGATGATAACCTAGCCTTGAACTCTCTCTCTGACTAGATGATGGAAAATACAAGGTCTGACTATTC | scaff.169 (+): 2602651- 2602728;  scaff.5343 (-):3900- 3977 | The second copy is located in the short scaffold 5343 and is probably a fragment of gas5 pseudogene. |
| frog | SNORD81 | TGGTAAATGATGACAATCACTTTGAACTCTCTCACTGAAACTTGAGATGACAGACAAAGCACTGATTCCA | scaff.1(+): 6877973- 6878042 |  |
| mouse | SNORD82 | AGCACAAGTGATGAGTGACAAAGGGACTTAATACTGAACCATGGGGTTGAAATGAAATATGCTGATGTGCT | chr.1 (-): 88252835- 88252905 |  |
| opossum | SNORD82 | GGGCACAAGTGATGATCCACATAGGGACTTAATTCTGAAGCCTGATGCAATGCTTGCTATATATGCTGATGTGCTC | chr. 2 (-): 536435642- 536435717 |  |
| platypus | SNORD82 | AAAACAAGCACAAATGATGAGCCTAAAGGGACTTAATACTGAAACCTGATGTAACTAAATAATATATGCTGATCGTGCTGTAGTTTT | Contig 30838(-): 4968-5054 |  |
| chicken | SNORD82 | AGCACAAATGATGATTATAAGGGACTTAATACTGAAATGTGATGTGATTTTTGAGCAGAACTGATGTGCT | chr. 9 (-): 16375090 - 16375159 |  |
| lizard | SNORD82 | GATGCCAGCACAAATGATGATTTATTGGGACTTAATACTGAATTTTTTGATGTCTCTAGTATGAGCTGATGTGTTgggcatt | scaff. 852 (-): 25790- 25871 |  |
| frog | SNORD82 | gcatatgTGATGAGCATTATGGGaCTTAATTCTGAAAACTTTgatgtcttacacttgctgatatgc | scaff.285 (+): 1170473- 1170538 |  |
| zebrafish | SNORD82 | tattaaaactacacATGATGATCAACAAGGGACTTAATACTGATGTATGtgatgtcaccttattactgttctgatgcttgtttgagtg | chr.22(-): 34855326- 34855413 |  |
| fugu | SNORD82 | TTTGCCATGTAAATGATGATCTCTTAGGGACTTAATACTGATTCCGTATGATGTCATGAACAGATCTGATGCTGGTTAAG | Un (-): 102412436- 102412515 |  |
| mouse | SNORD87 | gctggcACAATGATGACTTAaGTTTTTGCCGTTTACCCAGCTGAGtGTTTCTTTGAAGAGAGAATCTTAAaaGACTGAGAtgccagc | chr.1(-): 9932544- 9932629 |  |
| rat | SNORD87 | gctggcACAATGATGACTTATGTTTTTGCCGTTTACCCAGCTGAGGGTTTCTTTGAAGAGAGAATCTTAAGACTGAGCtgccagc | chr.5(+): 8841821- 8841905 |  |
| dog | SNORD87 | actggcACAATGATGACTTAAATTATTTTTTGCCGTTTACCCAGCTGAGGGTTTCTTTGAAGAAATAATTTTAAGACTGAGAtgccagt | chr.29 (-): 19464690- 19464778 |  |
| opossum | SNORD87A | TCATTGGCACAATGATGattttttattttaaaaaaaatGCCGTTTACCCAACTGAGTGAATCATTGAGGTAATTTAAAAATCTGATGTGCCATGA | chr.3 (+): 170605006- 170605100 |  |
| opossum | SNORD87B | ACTGGTACTATGATGACTTAATTCTACCTTTTTGCCGTTTACCCAACTGAATTTGACTTTGATGTAAATCTAAAGACTGAGTACCAGT | chr.3(+): 170606325- 170606412 |  |
| opossum | SNORD87C | TTTGCTGGTTAATGATGATTTGACAAGTTTTTGCCGTTTACCCTACTGAACATTTTGATGACCTATAAATCTGAATCCCAGCAAA | chr.3 (+): 170607114- 170607198 |  |
| platypus | SNORD87A | TGGCATTAATGATGATTTTTCTTTTTGCCGTTTACCCGGCTGAGTGCTTCTTTGAAGTGGACTTAAATCTGAGATGCCA | chr.7 (+): 1217665- 1217743 |  |
| platypus | SNORD87B | ACTGGTACAGTCATGATTTTACCTTTATGCCGTTTACCCAGCTGAATGTTTCTTTGATTGAAGTTTTGAAGTCTGAGATGCCAGT | chr.7(+): 1218172- 1218256 |  |
| platypus | SNORD87C | AGGGGGCTTTGCTGGCTTAATGATGATTAAAAAAATATTCGCCGTTTACCCAACTGAAAGTTTTTGATGTGCACTTTTTAATCTGAAATCCAGCAAAAGTTTTTT | chr.7(+): 1218491- 1218595 |  |
| chicken | SNORD87A | ACCTGTCTGCTGGCGTTGTGATGATGACTCTTTGCCGTTTACCCAGCTGATTTCTGTGATGAGTAACACTGTATCTGATGTGCCAGCTCATCAGTAGGT | chr.2 (-):  119633762- 119633860 |  |
| chicken | SNORD87B | ACCTGTCTGCTGGCATTGTGATGATGACTCTTTGCCGTTTACCCAGCTGATTTCTGTGATGAGTAACACTGTATCTGATGTGCCAGCTCATCAGTAGGT | chr.2(-):  119631554- 119631652 |  |
| chicken | SNORD87C | TACTGGCATCATGATGATATCTTTGCCGTTTACCCATCTGACTGGTTGTTGATGTGTATCTTTGAATCTGAAGTGCCAGTA | chr.2(-):  119630892- 119630972 |  |
| lizard | SNORD87 | TTGCTGGCACAATGATGATTTCCTTTTGCCGTTTACCCATCTGAATAAATCCTGATGTGTTTTTTCCTAATTCTGAGATGCCAGTAA | scaff.185 (+): 684842- 684928 |  |
| frog | SNORD87A | GGCATAATGATGATTATATTTTGCCGTTTACCCAACAGAATGTATGTGGTGAAACCTTAATAAAAATCTGATATGCC | scaff.254 (+): 341143- 341219 |  |
| frog | SNORD87B | TGGCTCTATGATGATCGTGATTTTTGCCGTTTACCCATCTGAATGGCAAGTGCTGATATTCTCTTATCTGAGGAGCCAAG | scaff.254 (+): 343549- 343628 |  |
| frog | SNORD87C | GGCACAATGATGATTATATTTTGCCGTTTACCCAACAGAATGTATGTGGTGAAACCTTAATTCTAATCTGACATGCC | scaff.254 (+): 344378- 344454 |  |
| frog | SNORD87D | GGCTCTATGATGATCAATGATTTTGCCGTTTACCCATCTGAATGGCAAGTGCTGATATTATTTTATCTGAGGAGCC | scaff.254 (+): 344793- 344868 |  |
| zebrafish | SNORD87A | TTTTTGCCTAAAGCAATGATGAGTTCACATTTTTGCCGTTTACCCATCATACCAGATTGGGGTGCTGAAACTGTGACTTTCTGAAGCAAAAG | chr.24(+): 22325269- 22325360 |  |
| zebrafish | SNORD87B | tGTTGATTTTACAATGATGAATATCACACTTTCGCCGTTTACCCATCAGACCAAATTGGGTTGCTGATACCGTGATGATCTGAAGTAACAGTG | chr.24(+): 22325552- 22325644 |  |
| zebrafish | SNORD87C | TTGTTGCAATGATGAATATCACACTTTCGCCGTTTACCCATCAGACCAAATTGGGATGCTGATGCTGTGACAATCTGAAGCAACAG | chr.24(+): 22325973- 22326058 |  |
| zebrafish | SNORD87D | TGCTTGTTGCAATGATGAATATCACACTTTCGCCGTTTACCCATCAGACCAAATTGGATTGTTGATACTGTGACTATCTGAAGCATAAAGCA | chr.24(+): 22326226- 22326317 |  |
| zebrafish | SNORD87Ψ | AATTCTTGTTGCAATTATGAATATTGCACTTTCGCCGTTTATCCCATCAGACCAAATTTCATTTTTGTCTAGGTCTGCTTTCTCAGGTCCAATT | chr.24 (+): 22325797- 22325890 |  |
| fugu | SNORD87A | TGCTGCTGGAATCGTGATGATATCACATTTTCGCCGTTTACCCATCAGAATAAGATGTTGAAAAGTGACTATCTGAATCAGCAGTG | Un. (-): 168276871- 168276956 |  |
| fugu | SNORD87B | TGCAGCTGTCATGGTGATGAATACCTTTTTTGCCGTTTACCCATCAGAAAAACATTTGGTGAAAAGGAATAATCTGAGTTACCAGCTGCA | Un. (-): 168276550- 168276639 |  |
| fugu | SNORD87C | GTGATTTGCTGTCATGATGAGTTCACCTTTTCGCCGTTTACCCATCAGACCAAAATGGACTGCTGAAAATGTGACAAACTGAACCTAAAGATGT | Un. (-): 168276293- 168276386 |  |
| medaka | SNORD87A | TGCTGGAATAGTGATGATTTCACACCTTTGCCGTTTACCCATCAGACACTAAGATGGTGAATTTGTGCCTAACTGAATTTCCAGCA | chr.20 (+): 15228885- 15228970 |  |
| medaka | SNORD87B | GCTGGAATGGTGATGAGTTCACTTTTTGCCGTTTACCCGTCAGACGTTAAAGTGGTGAAATTGTGAAAACTGACCAGC | chr.20 (+): 15229296- 15229373 |  |
| mouse | SNORD88A | CCAGGACCCCTCTGATGTTCAGCACTGGGCTCTGACCATCCTGAGGACATGGTGCCCCCAGGGACCTTTGACACCCTGGGGTCTGAGGGGCCCTGG | chr.7(+): 51505219- 51505314 |  |
| mouse | SNORD88B | GGACCCCCTTGATGTCCAGCACTGGGCTCTGACCATCCTGAAGACATGGTGCCCCCAGGGACCTTTGACACCCTGGGGTCTGAGGGGTCC | chr.7 (+): 51505505- 51505594 |  |
| mouse | SNORD88C | GGCTCCTGTGATGTTTAGCACTGGGCTCCAAACACCCTTGAGGACACAGTGCCCCTCCAGGACCTTTGACACCTGGAGATCTGAGGACCT | chr.7(+): 51503461- 51503550 |  |
| mouse | SNORD88D | TGGGAACCCCCATGATGTATATAGCACTGGGCTCTGACCACCATGGTGATATGTTGTGCCTCCAGGACCTTTGACATCCAGGAGTCTGAGGGGTTTCCA | chr.7(+): 51505817- 51505915 |  |
| dog | SNORD88A | GGGGCCCCTGTGATGTCCAGCACTGGGCTCTGACCTCCCCTGAGGACACAGTGCCCCCCAGGGACCTTTGACACCCAGGGGTTCTGAGGGGCCCC | chr.1(+): 108929778- 108929872 |  |
| dog | SNORD88B | TGGGACCCCCATGATGTCCAGCACTGGGCTCTGACCTCCCCTGAGGACACAGTGCCCCCCAGGACCTTTGACACCTGGGGATCTGAGGGGCCCCA | chr.1(+): 108930243- 108930337 |  |
| dog | SNORD88C | GGGCCTCCCATGATGTTCAGCACTGGGCTCCTATCACCCCTGAGGATGCAGTGCCTCCCAGGACCTTTGACACCTGGGGATCTGAGAGGCCC | chr.1 (+): 108928093- 108928184 |  |
| opossum | SNORD88A | CCGGGTAGGGCTCCCATGATGCCAGCACTGGGCTCTGAGCCTCTGAGGACAAACACGGTGCCCCCCAGGCCTTTGACTCCTGGGGGTCTGAGGAGCCCTGGCCCCGG | Un (+): 127020518- 127020624 |  |
| opossum | SNORD88B | CAGCCCCGATGATGGCAGCACTGGGCTCTGAGCGCTGTGAAGACGTCGGTGCCCCCTGGGCCTTTGACTCCCAGGGGTCTGAGGGGCTG | Un (+): 127021011- 127021099 |  |
| platypus | SNORD88 | GGTTTCCATGATGTCTTGCACTGGGCTCTGATCTCTGAGGACAACGGTGCACCCCAGGACTTTGACCCTCGGGGAGTCTGAGGATGCC | Contig 23258 (+): 11267- 11354 |  |
| lizard | SNORD88A | CAGGGTTGCTCTCATGATGTAAGCACTGGGCACAGACAATTGCTGTGGTGATAAAAGTGCCCCCATGCCTTTGACATCATGGGGACTGAGGAGCAAAATCCCTG | scaff.306 (+): 349811- 349914 |  |
| lizard | SNORD88B | AACCCCAATGATGTCAGCACTGGGCTCCGATCACTGTGATGATTTTGGTGCCTTCTGGAGACTTGACACTCTAGGAGACTGAGGGGTT | scaff. 306 (+): 350725- 350812 |  |
| frog | SNORD88A | CTTCTGGTTCAGTGATGTTAGCACTGGGCTCTGAATTGTATGACAGACACTTGTGCCCCTCTGTCCTGACATTCAGAGGGCCTGAGGGCCAGAAG | scaff.106 (-): 439000- 439094 |  |
| frog | SNORD88B | CCTGTCCCTATGATGTCAGCACTGGGCTCTGAAGTCTGAGTGACAACTGTGCCCCACTGTGACTTGACATGCAGTGGGACTGAGGGACAGG | scaff.106 (-): 438381- 438471 |  |
| frog | SNORD88C | GTTAATCCCTATGATGTGAGCACTGGGCTCTGAAGCCTCATTTGCTGATACATGTGCCTCTCTGTGACTTTGACATGCAGAGAGACTGAGGGAATGGT | scaff.106 (-): 437405- 437502 |  |
| frog | SNORD88D | CCTGTCCCTGTGATGTCAGCACTGGGCTCTGAAATCTGAGTGACAACTGTGCCCCACTGTGACTTGACATGCAGTGGGACTGAGGGACAGG | scaff.106 (-): 436731- 436821 |  |
| zebrafish | SNORD88 | ACGCTTCAGTGATGTTTAGCACTGGGCTCTGAGTTCATGTGTAGACAAAAGTGCAGTTTTGCTCTTGACATGGCAGAACCTGAGAAGTGT | chr.3 (+): 28418650- 28418739 |  |
| tetraodon | SNORD88A | TCTCCGTTGATGACGCGCACTGGGCTCTGAGTCCACGTGGAGACAAACGTGCACGTCTGGCCCTGACAAGCCGGAGCTTCTGAGGAGA | chr.3 (-): 13850800- 13850887 |  |
| tetraodon | SNORD88B | TTGGCTCTCCCATGATGACGTGCACTGGGCTCTGAGTCCACGTGGAGACGAGCGTGCATGTCTGGCCCTGACAAGCCAGATCTTCTGAGGAGAGCTAG | chr.3 (-): 13850510- 13850607 |  |
| tetraodon | SNORD88C | CTCCCATGATGACGTGCACTGGGCTCCGAGTCAGCGTGGGGACAAAAGTGCATCTCTGGCCCTGACAAGCCAGAGCTTCTGAGGAG | chr.3 (-):  13850217- 13850302 |  |
| mouse | SNORD91A | TAGAGAAGTCAATGATGGTCATGCCCAAGATGTCTGAACCTGTCTGAAGCATCTTTTTGATGTAGTCTATGTGGTTCTGAGACTTCTCTA | chr.11(+): 74718926- 74719015 |  |
| mouse | SNORD91B | AGGAGCCAATAATGTTTTTATTCAAAATGTCTGAACCTGTCTGAAGCATCCCAGTGATGCAAAATGTTGTACTGAGGCTTCT | chr.11(+): 74719614- 74719695 |  |
| cow | SNORD91A | CAGAGAAGTCAATGATGGTTTTACCTACATGTCTGAACCTGTCTGATGCACCTCAGTGATGTCATCTCTGTATGGTTCTGAGACTTCTCTG | chr.19 (-): 23391371- 23391461 |  |
| cow | SNORD91B | TAGGAGCCAATGATGTTTCCATTCAAAATGTCTGAACCTGTCTGAAGCATCCCAGTGATGCAAACTCTGTGTGGTGCTGAGGCTCCTG | chr.19 (-): 23390076- 23390163 |  |
| opossum | SNORD91A | GAGGAGCTCATGATGTTGGCTGAAATGTCTGAACCTGACTGAGACATCTGTGTGATGCATCCCCATCATGCTCTGAAGCTTCTC | chr. 2 (-): 517793858- 517793941 |  |
| opossum | SNORD91B | GGAGAATGGAGAAGCCAATGATGGTTTTGCCAGAATGTCTGAACCTGTCTGAAGCATCTTAGTGATGCTACATCCATATGGCTCTGAGGCCTCTTTCTCT | chr. 2 (-): 517793394- 517793493 |  |
| platypus | SNORD91A | TGGAGAAGCTAATGATGAGGAAAGCTATAATGTCTGAACCTGGCTGAGGCATCTTTGTGATGCATTCTCTTTTTAGCTCTGAGGTTTCTCCA | Ultra 382 (+): 278561- 278652 |  |
| platypus | SNORD91B | AGAAGCCGATGATGACTCGAGCCAAAATGTCTGAACCTGTCTGAAGCATCGGTGACGCATTCTTTATGGCTCTGAGGCTTCT | Ultra 382 (+): 279239- 279320 |  |
| platypus | SNORD91C | ACCAGAAGCCCGTGATGTCCTAGCCGTGATGTCTGAACCTGGCTGAGGGGACTGTGAAGATCAATCCCTTTTTGGCTCTGAGGCTTCAGGGT | Ultra 382 (+): 279932- 280023 |  |
| platypus | SNORD91D | ATGGAGAAGCCAGTGATGGTTTTTGGCCAAAATGTCTGAACCTGTCTGAAGCATTTCAGTGAAGTTCCATACGGCTCTGAGGCATCTCTGT | Ultra 382 (+): 280874- 280964 |  |
| chicken | SNORD91A | GGAAGCCTGTGATGTCCTAGCCATGATGTCTGAACCTGGCTGAAGGACTCTTCTGAAGATGAACCCTCAGGCTCTGAGGCTTCC | chr.19 (-): 5586640- 5586723 |  |
| chicken | SNORD91B | GAAGCCTGTGATGTCTTAGCCACAATGTCTGAACCTGGCTGAAGGATTCTTCTGAAGACACACACTTGGGCACTGAGGCTTC | chr.19 (-): 5585503- 5585584 |  |
| mouse | SNORD92 | AGCTGTGGTGCTGTGATGATGCCTTAAAATTGTGGTTTCGACTCACTGAGAGTAACG*TGA*GGACCTACAGTTCCTTGGCTGTGTCTGAGCACCAGAGC | chr.17(+): 71980614- 71980711 |  |
| cow | SNORD92 | TTTGGTGCTATGATGATGCCTTAAAATTGTGGTTTCGACTCACTGAGAGTAACATGAAGACCTACAATTCCTTGGCTGTGTCTGAGCACCCAAG | chr.11(-): 72883537- 72883629 |  |
| opossum | SNORD92A | GTGCTGTGATGATACTAAATTGTGGTTTCAACTTAACTGATAGTTGAATGAGGACATCTAATTCCTTGGCTGTGTCTGAGCAT | chr.1 (+): 510832022- 510832104 |  |
| opossum | SNORD92B | TGTGCTATGATGACATTCCATAGTGGTTTCATCAAACTGATAATTTAATGAGAACACTAAATTCCTTGGCTGTATCTGAGCACA | chr.1 (+): 510833148- 510833231 |  |
| platypus | SNORD92A | GGGGTTACTGAGATTTGATGCTGTGATGACGTTTTCAACTGTGGTTTCTGTTCACCGACGGTTGAATGAGGACAGATAATTCCTTGGCTGTGTCTGAGCATGTCCAGAACCCC | Ultra202 (+): 1019586- 1019698 |  |
| platypus | SNORD92B | AGGGGGTTACTGCGATTTGATGCTGTGATGACATTTTCAATAGTGGTTTCTGTTCACTGACGGGTGAATGAGGACAGATAATTCCTTGGCTGTGTCTGAGCATGTCCAGAACCCCTT | Ultra202 (+): 1022813- 1022929 |  |
| platypus | SNORD92C | TGGGTAGATGCTATGATGATCACTTCAACTGTGGTTTCAACTCACCGACTGTTGAATGAGGACATGAAATTCCTTGGCTGTGTCTGAGCATAACCCA | Ultra202 (+): 1025693- 1025789 |  |
| chicken | SNORD92A | AGCTATTTCTGTGATGATACCTTCAACTGTGGTTTCAACTTCCTGACAGTTGAATGAGGACGTTGAATTCCTTGGCTGTGTCTGAGAATGCT | chr.3(-): 8531256- 8531347 |  |
| chicken | SNORD92B | CTGGAAAAATGCAGTGATGATCTCTTAAGTAGTGGTTTCAATTCACTGATTGTTGAATGAAGACCTGAATTTCCTTGGCTGTGTCTGAGCATGTTTCCAG | chr.3(-): 8530028- 8530127 |  |
| lizard | SNORD92 | CTGCTGGAAAAAATGCTGTGATGATACCTGGAAATGTGGTTTCAACTATCTGACGGTTGAGTGAGGACATAAAAttccttggctgtgtCTGAgcaCTACCAGCAG | scaff.221 (-): 1023565- 1023669 |  |
| frog | SNORD92A | CCAATACTGTGATGATGCCAAACATTGTGGTTTCAACTCACTGATTTCGGTGAAGACCCCTTTACCTTGGCTGTGTCTGAGTATTGG | scaff.372 (-): 390298- 390384 |  |
| frog | SNORD92B | CAATACTGTGATGATGCCCAACATTGTGGTTTCGACTCACTGATTTCGGTGAAGACCCCCTTACCTTGGCTGTGTCTGAGTATTG | scaff.372 (-): 389689- 389773 |  |
| frog | SNORD92C | AAGCTTTGCTGTGATGACTTTACATTGTGGTTTCCATCACTGAACGTGCAGTAGTGAGGACACTCATTTCCTTGGCTGTGTCTGAGCGAAGCTT | scaff.372 (-): 387562- 387655 |  |
| mouse | SNORD93A | TTGGCCAAGGATGAGAACTCTAACCTGACAGGATACGCTGCTGTGATGGGTTAAAGGATTTACCTGAGGCCAA | chr.5(-): 23216808- 23216880 | The gene is localized in intron of AK151523 gene. |
| mouse | SNORD93B | TTGGCCAAGGATGAGAACTCTAACCTGACtGGATACGCTGCTGTGATGGGTTAAAGGATTTACCTGAGGCCAA | chr.5(+): 23358027- 23358099 | The gene is localized in intron of AK002390 gene. |
| cow | SNORD93 | TTGGCCAAGGATGAGAACCCTAATCTGATTGGATACCCTCTGCTCTGATGGGTTAAAGGATTTACCTGAGGCCAA | chr.4(+): 32839148- 32839222 |  |
| opossum | SNORD93 | TTGGCCAAGGATGAAAACCACAGTCTGATTGGGCATGCTTTCCATGCTCTGATGGGTTAAAGGATTTACCTGAGGCCAA | chr. 8 (-): 296764861- 296764939 |  |
| platypus | SNORD93A | TTGACCGAGGATGAAAATCATAATCTGATTGGGCATATCTCACTAATATGCTCTGATGGGTTAAAGGATTTAGCTGAGGCCAA | Ultra65(-): 870996- 871078;  Contig 44612 (+):1624- 1706 |  |
| platypus | SNORD93B | TTGGCCGAGGATGAAAATCATAATCTGATTGGGCATATCTCAGTAATGTGCTCTGATGGGTTAAAGGATTTAGCTGAGGCCAA | Ultra65(-): 870747- 870829;  Contig 44612 (+):1877 - 1959 |  |
| chicken | SNORD93 | AGTATTTGGCCAGGGATGAAAACAATCATCTGATTACTTGCATACTTACAGTGTTATGCAGAAGTCTGATGGATTAAAGGATTTTCTGAGGCCAAAACT | chr. 2 (+): 30911544- 30911642 | chicken and lizard SNORD93 RNA can guide the modification of a neighboring site in rRNA. The nucleotides complementary to the SNORD93 RNA target are shown in red. |
| lizard | SNORD93 | GTGGAGTTTCTGGCCAAGGATGAAAAATATGAACTGATTGCAGATACTTCTGTAGAGTGTTATGCACAAATCTGATGTTTTAAAGGATTTTCTGAGGCCAGAATGTCAGCGTCCAC | scaff.67 (-): 1834902- 1835017 |
| frog | SNORD93A | AAGTAGCCAGTGATGAGGAATGTACTCCTGAATGTAGATAATTGTGTCTCTGATGCACTAAAGGATTTACCTGAGGCTACTT | scaff.56 (-): 3553413- 3553494 |  |
| frog | SNORD93B | TAGCCATTGATGAGGAATGTACTCCTGAATCACTTCTGTATTTGATGTGCAAAAGGATTTACCTGAGGCTA | scaff.56 (-): 3553109- 3553179 |  |
| frog | SNORD93C | GTAGCCTATGATGAGGACTGTACACCTGAATGTAGCTATAACATCTGCATTTGATGTATTAAAGGATTTACCTGAGGCTAC | scaff.56 (-): 3552617- 3552697 |  |
| frog | SNORD93D | GTAGCCAATGATGAGGAATGTACTCCTGAATAGAGATGTTACTTCTTTATTTGATGTCTCAAAGGATTTACCTGAGGCTAC | scaff.56 (-): 3551234- 3551314 |  |
| zebrafish | SNORD93 | GCTATGATAAGCAAGTATGAGCTTCGTTACCTGTATAGATAGCTAAAATTAAGTCTGTGTGCTTTAGAGGATTTACCTGAGAGT | chr.6(-): 53182834- 53182916 |  |
| mouse | SNORD94 | TATGAAGCAGGCTGTGATGATTGGCGCAGGGGTACGGACCTCAGCTGAGTCATGGGAGCTGAATGTATGTGTTTTGCCTCTGTCCTGCATGTGGCAGGCTGTTGGCATTCACATACATGAGACTGTTGCCTTAATCTGAGCCTGTTCTTCATA | chr. 6 (-): 71832542- 71832694 |  |
| opossum | SNORD94 | GAGGCTGTGATGATTGGCGCAGGGGTACGGACCTCAGCTGAATCATGGGAGCTGAATGTATGTGTGTCTCTGCCCTCCTGCATGTGGCAGGTTGATGGGGAGCACTTACATGAGACTGGTGCCTAAATCTGAGCCTC | chr. 1 (-): 717381126- 717381262 |  |
| platypus | SNORD94A | TCGGCGGCGATGATGATTGGCGCAGGGGTACGGACCTCAGCTGGATCATGGGAGCTGAATGTGAGCCGGGCCTTCCCTGTGGGGCGGACGTGTGGTCTGCCGCCGGGGAGCACGTTCACATGAGAATTTCGCCAGACACTGAGCCGCCGG | Contig 3360 (-): 17144- 17293 |  |
| platypus | SNORD94B | GTCGGCGGCGGCGATGATGATTGGCGCAGGGGTACGGACCTCAGCTGGATCATGGGAGCTGAATGTGAGCCGGGCCTTCCCTGTGGGGCGGACGTGTGGTCTGCCGCCGGGGAGCACGTTCACATGAGAATTTCGCCAGACACTGAGCCGCCGGAC | Contig3360 (-):15197 - 15352 |  |
| platypus | SNORD94C | CCGTGGCTGTGATGATCGGCGCAGGGGTACGGACCTCAGCTGCATCATGGGAGCTGAGTGTAGGTGGGTCTCTGAGTCCTGCACGTGGTAGGACGGAAGAGAGCACTTACATGAGACTGGTGCCTAAGTCTGAgcctcgg | Contig 3360 (-): 3215- 3354 |  |
| platypus | SNORD94D | CCGTGGCTGTGATGATCGGCGCAGGGGTACGGACCTCAGCTGCATCATGGGAGCTGAGTGTAGGTGGGTCTCTGAGTCCTGCACGTGGTAGGACGGAAGAGAGCACTTACATGAGACTGGTGCCTAAGTCTGAgcctcgg | Contig 3360 (-): 4300- 4439 |  |
| chicken | SNORD94A | ATCCGTGGGATGGTGAAGGTAAAATTCNNNNNNNNNNCTAGATCATGGGAGCTGAGTGCAAACTGTTGATTTCAGTGCAGCTGGCGTGTGGCTGGCTCACTGGAAACAGGTTTGCATGAGAACTTCGCCAAAGACTGAGCCAT | chr. 4 (+): 88701421- 88701563 |  |
| chicken | SNORD94B | CATGGCAGTGATGATTGGCGCAGGGGTACGGACCTCAGCTAGATCATGGGAGCTGAGTGCAAACTGTTGATTTCAGTGCAGCTGGCGTGTGGCTGGCTCACTGGAAACAGGTTTGCATGAGAACTTCGCCAAAGACTGAGCCATG | chr. 4 (+): 88705037- 88705181 |  |
| lizard | SNORD94 | GGCAGCAATGATGATTGGCGCAGGGGTACGGACCTCAGCTAGATCATGGGAGCTGAGTGTGAGTTTTCAGTGTCTGGCGTCGCTCGCATGTGGCTTGCTTCCTGGACAGTGGCTCACACGAGAATGTTGCCTTATGCTGAGCTGCC | scaff. 225 (-):889686- 889831 |  |
| frog | SNORD94A | TTGCAATGATGATTGGCGCAGGGGTACCGACCTCAGCTGGATAATGGGAGCTGAGTGCAGAGCCGTGTCTGTCTGGTTATCCGACGTGTGGTTGGTGGCCAGGAGACTCTCTGCATGATACAGCCGCCATACTGAGCAA | scaff. 280 (+): 27995-28133 |  |
| frog | SNORD94B | AAACGACTGTGATGATTGGCACAGGGGTACGGACCTCAGCTGGATCATGGGAGCTGAATGTGAACTTCCCCCTGAGGTTCCTGCATGTGGTGGGATACCCGGGGGGGCGTTCATATGAGAACAGAGCCTTAATCTGAGCCGCTTCTTT | scaff. 280 (+): 33328- 33475 |  |
| zebrafish | SNORD94A | TTATTGGGAGCAATGATGAGTGGCGCAGGGGTACGTACCTCAGCTGATAATGGGAGCTGAGTGTTGACTGCTCAAAATCAGTGGACATGTGGTCAGCTGGTTGTAGGGTGTCAGCACGAAATTATCGCCTTGACTGAGCTCCCTAA | chr.20 (+): 38011521- 38011666 |  |
| fugu | SNORD94 | ACGCCTCCCCGTGGCTCTGTGATGATGACACGCGCAGGGGTACGGACCTTGGCCAGTCATGGGGGCCGGGTGCTGCCACTGAGCCCACTGGAGGGCGAGTGGTCCTCCAGAgggggggcagcacagacaATCGCCTGTCTGAACAGAAGCTGCACGGAGGCGT | Un (+): 279167634- 279167796 |  |
| mouse | SNORD95A | GACGGGCGGAGCAATGATGACCCCAACATGCCATCTGAGTGGCTTTGCTGAAATCCAGAGGCTGTTTCTGAGCTGTTGTC | chr.11(+): 48614360- 48614439 |  |
| mouse | SNORD95B | GCCAGGCCCAACAGTGATGACCACAACATGCCATCTGAGTTGCTTTGCTGAAATCCAGAGGCTGTTTCTGAGCTGTGCCTGGT | chr.11(+): 48616634- 48616716 |  |
| dog | SNORD95 | GGGTCGGGCGGAGCGGTGATGATGCCAACATGCCATCTGAGTACCTGTGCTGAGAACCAGAGGCTGTTTCTGAGCTTACGCCCGGCCC | chr.11(-): 3432846- 3432933 |  |
| cow | SNORD95 | GGGTCGGGCGGCGCGGTGATGATCCCAACATGCCATCTGAGTGCCTATGCTGAAACCCAGAGGCTGTTTCTGAGCTGCTGCCCGGACCC | chr.7(-): 39721742- 39721830 |  |
| opossum | SNORD95A | GAGGAGAAGCAATGATGATTTTCTAACATGCCATCTGATGCCTATGCTGAATACAGAGGCTGTTTCTGAGCTTCTGCCTT | chr. 2 (-): 277760207- 277760286 |  |
| opossum | SNORD95B | GAGGTCCCGGTGATGAGACAGATGACATTGTCAGCTGACCCCCCCCTCGGGACTGAAGACAAGTCCTGCAATTCTGATGGGACCTT | chr. 2 (-): 277758071- 277758156 |  |
| platypus | SNORD95A | GGGGCTTGGGCCAGAGCCGTGATGAACTCTTAACATGCCATCTGATTGCCCTTGCTGAAACCCAGAGGCTGTTTCTGAGCATCTGGTCTTGCCCC | Ultra 696 (+):25192- 25286 |  |
| platypus | SNORD95B | GCCGGACAGAGCGCTGATGATCACTAACATGCCATCTGACTGCCCTTGCTGAAATCCAGAGGCTGTTTCTGAGCATCTGCCTGGC | Ultra 696 (+):28243- 28327 |  |
| platypus | SNORD95C | TGGGCCAGAGCGATGAAGAGTTCTAACATGCCATCTGAGCTGCCCCTGCTGAGACCAAGAGGCTGTTTCTGAGCTCTGCATCCA | Ultra 696 (+): 28997- 29080 |  |
| chicken | SNORD95 | TGCTGTGATGAAACTTCGATGCCATCTGACTGCATCCCTGTGCGGAAACCCAGAGGCTGTTTCTGAGCA | chr.16 (+): 113205- 113273 | The first antisense element is absent from the chicken, lizard, frog, zebrafish, and tetraodon SNORD95 RNA genes. This corresponds to the absence of this modification from frog and probable from other VEPM rRNA. |
| lizard | SNORD95A | TTGGCTCAGCTGTGATGAAACTTACGAGCGCCATCTGACTATTATCACCATGCTGATTCTCAGAGGCTGTTTCTGAGCTGCGCAA | scaff.274 (-): 1819110- 1819194 |
| lizard | SNORD95B | GGGCTTCTTTCCGCTGTGATGAAACTCAATATATGCCTTCTGACATTACTTCCGTGTAGAAAACCAGAGGCTGTTTCTGAGCTTGTTGCCC | scaff. 274 (-): 1817017- 1817107 |
| frog | SNORD95 | TGTTTGGCAACCTTGCAGTGATGCATTCTCTTAATGCCAGCTGATGTTTACTGCTGAATCTCAGAGGCTGTTACTGAGCAATATGTAAGTG | scaff.31 (-): 4176215- 4176305 |
| zebrafish | SNORD95A | GCGAACAAAAATGATGGAAAACATAATGCTGTCTGAATTCATGTGATTATAACAGAGGCTGTTTCTGAACGCA | chr.14(+): 55811676- 55811748 |
| zebrafish | SNORD95B | AGTGTTCAGTGATGGAACTTAATGCCTTCTGTAACTCTGTGATGATAAAAGAGGCTGTTTCTGAGAACTACT | chr.14 (+): 55813073- 55813144 |
| zebrafish | SNORD95C | gttttggagtggTGATGAAACTCAATGCCATCTGTGTCACTGTGATGATAAAGAGGCTGTTTCTGAacttaaaac | Zv7_NA179 (-): 1972- 2046 |
| tetraodon | SNORD95A | GCAGGTTTCATCAGTGATGAATCTGAAGCGTCTGTATCAGATTGATGAGAAACCAGAGGCTGTTTCTGAGAGATGCCTGC | chr. 1 (+): 918448- 918527 |
| tetraodon | SNORD95B | GGCGGCAGTGATGAAACTGTGAATGCTAACTGTACTATCTGATGATAACAGAGGCTGTTTCTGAGCTCC | chr.1 (+): 919122- 919190 |
| mouse | SNORD96 | tagaggatCCTAGTGATGACAAGACGACATTGTCAACCAATCCCCCACAAGGGAATGAGGACATGTCCTGCAATTCTGAATGggttctctg | chr.11(+): 48615529- 48615619 |  |
| dog | SNORD96 | ATCCCAGTGATGACAGATGACATTGTCAGCCAATCCCTACGTGGGAGTGAGGACATGTCCTGCAATTCTGAAGGGAT | chr.11(-): 3431716- 3431792 |  |
| cow | SNORD96 | TGGAGGATCCTGGTGATGACAGATGACATTGTCAGCCAATCCCCACATGGGAGTGAGGACATGTCCTGCAATTCTGAAGGGATTCTCTG | chr.7(-): 39720335- 39720423 |  |
| opossum | SNORD96 | GAGGTCCCGGTGATGAGACAGATGACATTGTCAGCTGACCCCCCCCTCGGGACTGAAGACAAGTCCTGCAATTCTGATGGGACCTT | chr. 2 (-): 277758071- 277758156 |  |
| platypus | SNORD96A | GAGGGTCCTGAGGATGAGACATGACACACAGCGACCGACCCCATCGGGGCTGACGACACATCCTGCAATTCTGATGGGACGCTT | Ultra 696 (+):26910- 26993 |  |
| platypus | SNORD96B | GGGTCCGGTGATGAGATGACGCATGTTGGCCGACCCCTGCTGACGACACGTCCTGCAATTCTGAAGGGACCC | Ultra 696 (+):27900- 27971 |  |
| chicken | SNORD96A | CTCTCCCAGTGATTACACCTGACACAGTCTGCTGATCCTCTGGGATCTGAGGACACGTCCTGCAATTATGATGGGAGAG | chr. 16 (+): 111283- 111361 | SNORD96 RNA from all non-mammalian spesies contains a substitution in box D. |
| chicken | SNORD96B | CTCCCAGCTCCGTCCTAATGATTAGAAGTGACACAGTGCCTGCTGACCTCTCTTTCTGGGGGGGGTCTGAGGACACGTCCTGCAATTATGAGGGGCTCAGGGAG | chr. 16 (+):112152- 112255 |
| lizard | SNORD96A | TTCTTCTCTGTCCCAATGATTAAATATGACATTGTCTACCTGATCTTCTCAATGGGATCTGAAGACAAGTCCTGCAATTATGATGGGACAGAGGAGGA | scaff.274 (-): 1828100- 1828197 |
| lizard | SNORD96B | TCCTGTCCCAATGATTAGATTTGACACACATCTACTGACTCTACTGGAGTTTGCGGAGATGTCCTGCAATTATGAAGGGACAGGA | scaff. 274 (-): 1825381- 1825465 |
| frog | SNORD96 | TTGGTCCACGTGATTAGAACAATTGTATTCTGAGTGTCTAATATAGGCACACTAATTTCAGGATGACACTCCTGCAATTATGAAGGGACCAA | scaff.31 (-): 4178192- 4178283 |
| zebrafish | SNORD96A | TTACCCTGTGATTTTAAATAAGACAAATTTCCTCTGAGCTCTTGAGCTCTGTTGACAAGTCCTGCAATTATGATGGGTAA | chr.14 (+): 55810769- 55810848 |
| zebrafish | SNORD96B | TCCCCCATTGATTATTCTCAATCACATAAACATCTGAATCCTTATGGTGACACTCCTGCAATTATGAGGGGA | chr.14(+): 55813385- 55813456 |
| tetraodon | SNORD96 | GGGACTTTACCGCTAGTTGATGCTGTGAGAACAACCCTGGTGATTATACCCATTCACTTTAAACTCTGATGACCACTGGTGACAGTCCTGCAAATATGAGGGGCCC | chr. 1 (+): 919398 - 919503 |
| human | SNORD97B | ACAGCCCAGTGATGATCACTATTCCTACTTAGAGAGAATAGGACTAACTTTCAGAAATCCAGGCATTtttctacctttcatactatctttctttcactttacttctcttttctgtcttttatcacttctttctttcttcattctttctctctttTGCCTGGATCGAGATTGTTAAGTCCCTCTCAGTGAAGGGTAAGATTATGAGATCTGAGGGCTGT | chr .12 (+): 49136620- 49136837 |  |
| mouse | SNORD98 | gaggatGAGTTATGATGTGTAAATCCTATTCCATTGCTGAAATGCAGTGTGGAACATAATGAACTGAACTCttcctt | chr.10(-): 62228318- 62228394 |  |
| opossum | SNORD98 | CAAGGATGAGTTGTGATGAGTAAAATCCTATTCCATTGCTGAAATACAGTGTGGAACATGATGAACTGAGCTCTTCCTTG | chr. 1 (-): 39002911- 39002990 |  |
| platypus | SNORD98 | GGGGGAGTTATGATGTATTTAAGTCCTATTCCATTGCTGAAATACAGTGTGGAACACGATGAACTGAACTCCTCT | Contig 917 (-):32926- 33000 |  |
| chicken | SNORD98 | CAAGAATGAGTGATGATGTTTTAAACCTATTCCATTTCTGAAATAGTCTGTGGAAAAAAATAACTGAACTCTTTCTTG | chr.6(+): 11845078- 11845155 |  |
| lizard | SNORD98 | GAATGAGTAATGATGCTTAAACCTATTCCATTACTGAAATAATCTGTGGAAAACAAAAACTGAACTCATTC | scaff. 187 (-): 800625- 800695 |  |
| zebrafish | SNORD98 | GGGAGCTGACTGTGATGTCTTTGTCCTATTCCATTGCTGAACGCTTTGCTGTGTGGAATAAGCTCATCTGATGTGGTCTCTCCC | chr.13(-): 14104551- 14104634 |  |
| mouse | SNORD99 | ACTGGTCCATGATGAAACCCATTATCAGTGGACATCTATGGATGAGAAATGCGGATATGGGACTGAGACCAGT | chr.4(+): 131866606- 131866678 |  |
| cow | SNORD99 | TGGTCTAGGATGAAACCTAATCTGAGTGGACATCTGTGGATGATAAATGCGGATATGGGACTGAGACCAG | chr.2(+): 129020686- 129020755;  Un.004.3702 (+):8975- 9044 |  |
| opossum | SNORD99 | TGACTGGTCAGTGATGAATCTCTTATCTGGTGGACATGCTTTGGATGACACTTGCGGATATGGGGCTGAGACCAGTCA | chr. 4 (-): 361011335- 361011412 |  |
| platypus | SNORD99 | GCTGGTTAAGGATGACACTCAATGGTGGACATGTATGGATGACACTTGCGGATATGGGACTGAGACCAGC | Contig 24898 (-): 6933- 7002 |  |
| chicken | SNORD99 | CAGCCTGGTCAGTGATGACACCTTTCTTTGGAGACTGCTGGATGAATCCTGCGGATATGGGGCTGAGGCCAGGCTG | chr.23(-): 2855499- 2855574 |  |
| lizard | SNORD99A | TCTGACtggtctgTGATGActcatgttTTTAATGGTGACAGCTGGATGAATCCTGCGGATATGGGGCTGAGACCAACCAGA | scaff. 1330 (+): 103554- 103634 |  |
| lizard | SNORD99B | tggtctgTGATGActcatgttTTTAATGGTGACAGCTGGATGGATACTTGCGGATATGGGGCTGAGACCA | scaff. 1330 (-):107395- 107464 |  |
| frog | SNORD99A | GGCTGCGCGGGGATGAATCTCTTATCTGGAGACGGTTGGATGAATCTTGCGGATATGGGACTGAGCGCAGCC | scaff.1546 (+):44668- 44739 |  |
| frog | SNORD99B | GAGGTTGCTCTGTGTGGGGATGAATCCCTTATCTGGAGACGGTTGGATGAATCTTGCGGATATGGGACTGAGCACAGGCGCCTC | scaff.1546 (+):45359- 45442 |  |
| frog | SNORD99C | AGGGGCTTGAGGCTGTGCGGGGATGAATCTCTTATCTGGAGACGGTTGGATGAATCTTGCGGATATGGGACTGAGCGCAGCCTTTGCCTTT | scaff.1546 (+):47992- 48082 |  |
| zebrafish | SNORD99 | GACTGATCAAGGATGATAGTATATTGATGGTGACTGCTGGATGAAACTTGCGGATATGGGACTGAGATCAGTT | chr.19(-): 22441774- 22441846 |  |
| fugu | SNORD99 | CTGATCTGTGATGACACCAAATCAATGAGGACTGCTGGATGAAGCTTGCGGATATGGGACTGAGATCAG | Un. (-): 128868227- 128868295 |  |
| mouse | SNORD100 | GGCTGTACATGATGAAAACAGTCTCCCTCTTCTGAATCTCGCTGAGGAAACTGCACGTCACCCTCCTGAAACAGCC | chr.10(-): 23505555- 23505630 |  |
| cow | SNORD100 | GGCTGTACATGATGACAACTGGCTCCCTCTACTGAACCGTGGTGAGGAAACTGCCATGTCACCCTATCTGACTACAGCC | chr.9(+): 73074132- 73074210 |  |
| opossum | SNORD100 | GCTGTACATGATGACAACTTGGCTCCCTCTACTGATAATAATGAAGAGAACAGCCATGTCACCCAACATATCTGAGCGTACAGC | chr. 2 (+): 407252030- 407252113 |  |
| platypus | SNORD100 | GCTGTACATGATGATAGCTTGGCTCCCTCTACTGAAAATGGTGAGGATAATAGCCATGTCACCCAATAGTATCTGAACGTACAGC | Ultra 520 (+): 1653849- 1653933 |  |
| chicken | SNORD100A | GCTGTACAATGATGAGACTTTGGCTCCCTCTACTGATACTTGTGAGGAAAGCAGCCATATGTTACCCAATACCTCTGAGAGTACAGC | chr.3 (-): 58701636- 58701722 |  |
| chicken | SNORD100B | CTGCTGCATCTGTGATGAAAATTTGGCTCCCTCTACTGAAACAAATGAGGAGAACTGCCTTAATCGTTACCCAAATCTTCTGAGATGTTGCAG | chr.3 (-): 58700401- 58700493 | SNORD100B RNA is encoded in the 3’ UTR of its host gene (RPS12). |
| lizard | SNORD100 | GCTGTACAGTGATGACAACATGGCTCCCTCTACTGAAATCTGTTGAGGAAAACAACCATAAATGTTACCCTTTATATCTGAGTGTACAgc | scaff. 35 (-): 5253261- 5253350 |  |
| frog | SNORD100A | TTTACCTATGATGAGAATTTGGCTCCCTCNNNNNNNNNNNNNNNNNNNNNNNNNNNNNNNNNNNNNNNNNNNNNNNNNNAACTTCCCATTGCAGATGACTGACACATAAA | scaff.172 (-): 1951026- 1951135 | The sequence of the gene contains a gap. |
| frog | SNORD100B | CCCACCTTTACCTATGATGAGAATTTGGCTCCCTCTTCTGAAACTAGTGAGGAAATTGCCAATTGTTACCCAATAATCTCTGAGGTGCATGGGGG | scaff. 172 (-): 1949544- 1949638 |  |
| frog | SNORD100C | AGTTGTATGCTATGATGATAACTTGGCTCCCTCTACTGAATGCTATGAGGAGAAAAGCCTAAATGTTACCCAATACTTCCTGAGTTTACAGCT | scaff. 172 (-): 1947854- 1947946 |  |
| zebrafish | SNORD100 | GTATCTGTGATGATAATTTGGCTCCCTCTACTGAAGTCTCTGAGGAAAACTGCCATTCGTTACCCAATTTCTTACTGAGATAC | chr.23(+): 30201813- 30201895 |  |
| tetraodon | SNORD100A | ATGTCGCTGATGATATCTTGGCTCCCTCTACTGAGTTTTAATGTGGAAGTCTGCCATTGTTACCCAATTTCTATCTGAGATGT | chr.14 (+): 5738160- 5738242 |  |
| tetraodon | SNORD100B | GCATCACTGATGATAATCTGGCTCCCTCTACTGATTATGGTGAGGAAAGTGCCACTGTTACCCAATTTTGTCTGAGATGC | chr.14 (+): 5738446- 5738525 |  |
| mouse | SNORD102 | GCTCTGGAACTTAATGATGATTGTCTGTTTGATTGCTTGATGCAATGTGAAAACCACATTTCACCGGCTCTGAGAGTTCTTGAGC | chr.5(+): 147646403- 147646487 |  |
| opossum | SNORD102A | GAGCTTGATGATGATTGTCTTATATGATTACTTTTCTAATGAGTAAAATGAAAACAACTTCACCGGCTCTGAAAGCTC | chr.4 (+): 287477229- 287477306 |  |
| opossum | SNORD102B | TTGATGTGCACAATGATGATCTTCCTAAATGATTGCCTTTCCAAAAGGAACATGAATATGTTATTTCACCGGTTCTGAGAGCACATTAA | chr. 4 (+): 287479605- 287479693 |  |
| platypus | SNORD102A | TGGTGCTCGATGATGATTGTCTCGTGTGATTGTTCTTTTGCAAGAGCAGAATGAACATACCATTTCACCGGCTCTGAGAGCTTA | Contig 59 (+): 2139256- 2139339 |  |
| platypus | SNORD102B | CTGCTCGATGATGATCTTCCTAAATGATGACTTTAGCAAAAGTCAAAAATGACTGCGTTATTTCACCGGTTCTGAGAGCAG | Contig 59 (+): 2140766- 2140846; Contig 146923 (-): 144-224 | Very short contig (contig 146923)contains a fragment of the SNORD102 RNA host gene (RPL21)including the SNORD102B sequence. Therefore, until the final assembly of the platypus genome become available it remains unclear wether SNORD102 RNA has two or more genes. |
| chicken | SNORD102A | CATTCTGGAGCTTGATGATGATTGTCTCCTATGATTGCTTGCTGAAAGCAAAGTGATCAAATCATTTCACCGGCACTGAAAGCGACTGGAATG | chr.1(-): 180618350- 180618442 | The nucleotide complementary to the SNORD102 RNA targets is shown in red. SNORD102B RNA can guide the modification of a neighboring site in rRNA.  SNORD102B RNA is encoded in the 3’ UTR of its host gene (RPL21). |
| chicken | SNORD102B | TTTGCTATTGCTCAATGATGATCATCCTCCTTTGATTACCTGGTGAGGTAATATGAGAGGACATGGAATAATTTCACCGGCGAACTGAGAGCAAATAGCAAG | chr.1(-): 180615965- 180616066 |  |
| lizard | SNORD102 | ACACtctggggcttgatgatgattgTCTCTTATGATTGCTTCTGCAAAGTGAGCAAATCATTTCACCGGCACTGAAAGCAAACCAGAGATGT | scaff. 347 (+):1009764-1009855 |  |
| frog | SNORD102 | AATGCTCCATGATGATATTTCTCCTAACTATTTGCTCTCTGAGCAGATATGAGTCTACCATTTCACCGGTTCTGAGAGCATT | scaff.795 (+):258215- 258296 |  |
| mouse | SNORD103A | TTGTCTGGCGGTGATGACCCACTTGCCCTCACTGAGGACAAAGTTCGATAATGAGAATCTTTGTTATGGACTCGTTCTGAGCCAGACAG | chr.4 (+): 130322243- 130322331 | Homologous human gene was named HBII-251. |
| mouse | SNORD103B | TTGTCTGGCGGTGATGACCCACTTGCCCTCACTGAGGACAAAGTTCGATAATGAGAATCTTTGTTATGGACTCGTTCTGAGCCAGACAG | chr.4(+): 130331653- 130331741 |
| mouse | SNORD103C | GACTGGCAGGGATGATACATACTTGCCCTCACTTAGACCAGAGGTCGATGATGAGAGCTTTGTTCTGAGCCAGTC | chr.4(+): 130305548- 130305622 |  |
| opossum | SNORD103A | TCTGGCGATGATGACACACTTGCCCTCACTGAGAATATAGTTTGGTGATGAGAACCTTTCTATATGTACTCAAAAACTGAGCCAGA | chr.4(+): 426186811- 426186896 |  |
| opossum | SNORD103B | TTGAAGACTGGCTGGGATGACACACTTGCCCTCACTGAGACAACTCATTGATGAGAGCTCTTGATTGTACTCATTGGTTCTGAGCCTGTCTTTAA | chr.4(+): 426205673- 426205767 |  |
| opossum | SNORD103C | GTCTGGCAGTGATGACACTCTTGCCCTCACTGAGAACACAGTTCGGTAATGAGAACCTTTACTATGTACTCAGAAGTGCTGAGCCAGAC | chr.4(+):  426240071- 426240159 |  |
| platypus | SNORD103A | GGGGTCTGGCAGTGATGACACTCTTGCCCTCACTGACACACTGAGGTGTGGTGATGAGAGCCAGCTCTGTGCTGAGCCCTGAGCCAGACCCC | Contig 23780(+): 3673-3764 |  |
| platypus | SNORD103B | GGCCCTGGGGGCTGGCAGGGATGACACACTTGCCCTCGCATAGATTCTGCTGATGAGAACATCTCTCCGTACTCGACTGTTCTGAGCCAGCCTCCGGGTC | Contig 23780(+): 5732- 5831 |  |
| platypus | SNORD103C | GGCCAGCGATGATGACACTCTTGCCCTCACGGAGAACTGCATTCGCTGAGGAGATCCTGTATGTGCTCAGAAGAGCTGAGCTggcc | Contig 23780 (+): 10800- 10885 |  |
| chicken | SNORD103A | CTTTATGGGTCTGGCGATGATGACACACTTGCCCTCACTGAGAACACTGTTCAGTAATGAGAGCCTCACAGTGTGCACAGCTGAGCCGGGCTCGCAGGG | chr.23(-): 463277- 463375 |  |
| chicken | SNORD103B | TTCTGGCGATGATGACTCACTTGCCCTCACTGAGATCCACTGCAGGGATTTTGTTGAGAGCTTCTTCTGGACTCATTGTGAGCTGAGCCTTGAG | chr.23(-): 456823- 456916 |  |
| chicken | SNORD103C | GAGGTCTGGCAATGATGACACACTTGCCCTCTCTGAGAACAGTGCTTGCTAATGAGAGAAGCTTTACATACTCAGCAGAGCTGAGCCAGACCTC | chr.23(-): 441274- 441367 |  |
| lizard | SNORD103 | TCAAAGGTCTGGCGATGATGACACTCTTGCCCTCACTGAGAACGCTGTTCCGTATTGATGATCCTCTTATGTGCTGAGCTAGACCTTTGA | scaff.762 (+):371579- 371668 |  |
| frog | SNORD103A | GGTCTGGTAATGATGACACACTTGCCCTCACTGAGAATGCCATTCTATAATGAGAACACCTATGTACTCAACCTGAACCAGACT | scaff.492 (+):246233- 246316 |  |
| frog | SNORD103B | TCTGGTGATGATGACACACTTGCCCTCACTGAGAATACTATTCTGTAATGAGAACACAATTGTACTCAACCTGAACCAGA | scaff.492 (+):246776- 246855 |  |
| frog | SNORD103C | GGTCTGGTGATGATGACACACTTGCCCTCACTGAGAATACTATTCTATAATGAGAACACCATTGTACTCAACTAACCTGAACCAGACC | scaff.492 (+):248200- 248287 |  |
| frog | SNORD103D | GGTCTGGTGATGATGACACACTTGCCCTCACTGAGAATACTATTCTATAATGAGAACACCATTGTACTCAACTAACCTGAACCAGACC | scaff.492 (+):248551- 248638 |  |
| frog | SNORD103E | GGTCTGGTGATGATGACACACTTGCCCTCACTGAGAATACTATTCTATAATGAGAACACCATTGTACTCAACTAACCTGAACCAGACC | scaff.492 (+):248899- 248986 |  |
| frog | SNORD103F | TTCGGCTTTGATGACACACTTGCCCTCACTGTAATCTTTGGTTCTGTGATGAGGTCAATATTGTACTCAAATTAAACTGAGCCTAA | scaff.492 (+):251036- 251121 |  |
| frog | SNORD103G | AGTCTGGTAATGATGACACACTTGCCCTCACTGAGAATACCATTCTGTAATGAGAACACCTATGTACTCAACCTGAACCAGACT | scaff.492 (+):251324- 251407 |  |
| frog | SNORD103Ψ | CCTGGCTGTGATGACACACTTGCCCTCACTGTGAATTTTATGTCTGATGAGGACAATATTGTACTCAAATTGAACTGTCTCA | scaff.492 (+):247901- 247982 |  |
| zebrafish | SNORD103 | GTTGGCTGTGATGACAAACTTGCCCTCTCTGAGAGAGCAGCTCTATTATGACAATCAAAAACTTTGCTGAGCCAAC | chr.19(-): 22442520- 22442595 |  |
| fugu | SNORD103 | AGTTCTGGCTATGATGACACACTTGCCCTCACTGAGATCAGGCTTGACTGAGACACTCGTACTCTCCTGAGCCACATT | Un. (-): 128868670- 128868747 |  |
| mouse | SNORD104 | GTGGCCGGCGATGATGACACTCCATACATAGCACGCGTTAGACTGCTGATCCGGGTGATGCGAATCGTAATCTGAGCCGACCGC | chr.11(+): 106362300- 106362383 |  |
| opossum | SNORD104 | GGGCGGCTGTGATGAACATCCATGAAAGCGTGTTAGACTACTGAGTACCATGAAGTGAATCAGAATCTGAGCCCAGCCC | chr. 2 (+): 211499287 -211499365 |  |
| platypus | SNORD104 | CTCGGCCCGCCTTGCTGTGATGACTTCCCTTGAAACGTGTTAGACTGCTGATGCGAATGATGCCACTTCCAATCTGAGCCCAGGGCGGGCCGGG | Ultra 288 (-): 3309543- 3309636 |  |
| lizard | SNORD104 | TGCTGGTTGCGGTGATGATTCTCTCAGCGTGTTAGACTGCTGATGCGGATGAGGAGACATTGGATCTGAGCCTCCAGCA | scaff.553 (+):687377- 687455 |  |
| tetraodon | SNORD104 | CCATGAATGATGTTTTTATCGCGTTAGACTTCTGATTTGAACATGAAGTCAACTCTTAATCTGACATGG | chr.3 (+): 9206278 - 9206346 |  |
| mouse | SNORD105A | GCTGCTCCTGTCTGATGATGAGCCCATTGCCCTGAGCTCTTGTGATGCTGGCTTCAAAGTAAACGCTCTGAAGTGAAGGAGC | chr.9(+): 20693926- 20694007 |  |
| mouse | SNORD105B | CGTGCACCTGATGAGAATTCTGCTGCTGAGGTTGTGTGATTGCTCAGACTCAAAGTAAACGCCCTGATGCATG | chr.9(+): 20695204- 20695276 |  |
| opossum | SNORD105A | CCCTGGTGACCATGATGATCTGATCTCTGCTGAGACCTTGTGATTGTGTCTTAAGTAAACGCTCTGAGGCCCCCGGGG | chr. 3 (-): 431356341- 431356418 |  |
| opossum | SNORD105B | AGGTTGTGGTATGTGATGAGAATGCTGATGTGATACTCCATGATCACTCAAAGTAAACGTTCTGATGCCTCTGGCCT | chr. 3 (-): 431355844- 431355920 |  |
| platypus | SNORD105A | CCTCGGGCACATGTGATGAATCCATTGTCCGCTGAGCCACGCGGTGACTTGACTAACAAAGTAAACGCTCTGATGTGCCCGGGG | Contig 29628 (+): 3940-4023 |  |
| platypus | SNORD105B | CCCCCGCTGTCATGATGACAAATTACTGGCTGAAATATCATGACTGGTTTCAGTAAACGCGCTGATGCAGCGG | Contig 29628(+): 5287-5359 |  |
| lizard | SNORD105A | TTTGATGCAAATGATGACAGTTTATTTTGCTGAGTACAACCAGTGATTCTCATTTCAAAGTAAACGCACTGATGCATCAGA | scaff. 404 (+): 1141552- 1141632 |  |
| lizard | SNORD105B | CTGATGCAAATGATGACTTGTGTGTTTTGCTGAGTTCAACCCATGATTCTCACTTCAAAGTAAACGCACTGATACATCAG | scaff. 404 (+):  1144067- 1144146 |  |
| lizard | SNORD105С | GGATGCAGTTGATGAAAAATCTTTGCACTCCTGACTCTGAGCTGTGATTTTTGCAAAGTAAACGCTCTGATGCATCC | scaff. 404 (+):  1150194- 1150270 |  |
| frog | SNORD105 | CCCCAGTTGGATGCATATGATGATGCTGTATAGTCTGCTGAGGGCTTCCCATGATCCTCTGTGAAGTAAACGCTCTGAACCATCCATCGGGG | scaff.92 (-): 428986- 429077 |  |
| zebrafish | SNORD105A | GGTGCAGATGATGAAAGTTTTACTCATGACTTTCCCCTGATTGTTTTCAAAGTAAACGCACTGATGCACT | chr.3(-): 53560273- 53560342 |  |
| zebrafish | SNORD105B | ATTAAAAAATTAAGTGCATATGATGAATTTGTATTATTATTGATGGAACGGTGATTCTTTTCAAAGTAAACGCACTGATGCACTTCTTTAAT | chr.3(-): 53557641- 53557732 |  |
| zebrafish | SNORD105C | TGGCACAAATGATGACATTTTAGAATTTTGACTGTTGAGTGATGTCTTTTCAAAGTAAACGCACTGATGTGTCCA | Zv7_NA421 (-): 3861-3935 |  |
| fugu | SNORD105A | ATGGTTTCGCTTGGATGATGACCACTTTTTGACGTGACAATTCTGTGATGTCTTCAAAGTAAACGCACTGATGAGCCTCCAT | Un. (+): 48386861- 48386942 | The nucleotide complementary to the SNORD105 RNA target is shown in red. SNORD105C and SNORD105D can guide the modification of a neighboring site in rRNA. |
| fugu | SNORD105B | TGGTCACAGTTTGGGCTCAGATGATGACATCTTGTAAATTGACTGTTCAGTGATGTTTTCAAAGTAAACGCACTGATGAGCCTGGTTGTGACCG | Un. (+): 48388208- 48388301 |  |
| fugu | SNORD105C | ACTCAAGATTTAAAGCCTCGTTGGCATGATGACAGTATAGTCTTCTGAGGTGACCTCTGAAGAGTTGTTAAAAGTAAACGCTTTCTGAATTTTAAATCTTGAGT | Un. (+): 48387628- 48387731 |  |
| fugu | SNORD105D | GGGTGTACAAGATGAAAATGCATTTATGAATTCAATGATTATTTCTAAAAAGTAAACGCTTACTGATGCACCC | Un. (+): 48388877- 48388949 |  |
| mouse | SNORD110 | CAGGCTTTGCAATGATGACTTATATATCTGTCAATCCCCTGAGAGATCACTGACGACTCCATGTGTCTGAGCAATGCCTG | chr.2(+): 130101236- 130101315 |  |
| cow | SNORD110 | GGCTTTGCAGTGATGACTTGAGTCTGTCAATCCCCTGAGTGAGTTTAATCACTGATGTCTCCATGTCTCTGAGCAAAGCC | chr.13(-): 53080740- 53080819 |
| opossum | SNORD110 | CCTGGGACAACAGCCGCTGCACTGTGATGAGTTTTTAAAATCTGTCAATCCCCTGACCATAGTGATGTGACTTACTCTCTGAGTACGGTGTCTGTCTCAGCCCCAGG | chr. 5 (+): 237926826- 237926932 |
| chicken | SNORD110 | CGCCCGGCCGTGGAGGTGATGTCTGAGTGAATCTGTCAATCCACTGAGCCGCTGTGCTGACAGCCCTCACTGCTGATCCCGGCCCGGGCG | Un_random (+): 42527563- 42527652 |
| lizard | SNORD110A | GGCACAGAGATGATGTATATTGTGAATCTGTCAATCCACTGAAGCTCTCGCTGTCGACACCCTCTCACTGCTGATCTGTGCC | scaff. 671 (+):148412- 148493 |
| lizard | SNORD110B | TGATGTGCATTGTGAATCTGTCAATCCACTGAAGCTCTCGCTGTCGACACCCTCNNN | scaff. 671 (+):154232- 154288 |
| frog | SNORD110A | GCTTCTGATGCACAATGATGTCATAATAATCTGTCAATCCACTGAGCCGTGTGATGTCAGCATTTCTACCCTGAGTGCTCAGAGGC | scaff.1626 (+):18445- 18530 |
| frog | SNORD110B | GGGACACAGACATGATGCCTCAGTGAAATCTGTCAATCCCCTGACGTCTGTGCTGATACCCTGCACTGCTGATCTGTGTCCC | scaff.1626 (+):22185- 22266 |  |
| zebrafish | SNORD110A | AGATGAAGTGATGCACACATAATCTGTCAATCCACTGAGTTATTGTGATGACAACATACCTACAAAACTGATCATCT | chr.21 (+): 3752802- 3752878 |  |
| zebrafish | SNORD110B | TCAGATGTTATGATGTATTTATAATCTGTCAATCCCCTGAAGCCTGTGATGACAGCATACCTCAATAACTGAACCTCTGG | chr.21(+): 3755994- 3756073 |  |
| tetraodon | SNORD110A | GTTGTGATGTCTTCATAATCTGTCAATCCACTGAAACCTTGTGCTGACGCTTCCTTTTGATTCTGAACAAC | chr. 11 (+):778033- 778103 |  |
| tetraodon | SNORD110B | CGGACCGATGATGTTGTACCAATATCTGTCAATCCCCTGAGTCACGGTGACGACACCAAATCCGGTGCTGATGGTCCG | chr.11 (+): 779626- 779703 |  |
| mouse | SNORD111A | GCCTGAAGTGATGATTCACAATCATGTCTCTTCTCTGAAGAATTCATGGAGATTCTGTGTATCTGATCAGGC | chr.8(-): 113366285- 113366356 |  |
| mouse | SNORD111B | CAGCCTGAAATGATGACCATTTAATTCATGTCTCTTCTCTGACATTCTCCTCTGGAGATGATTTTTGCCTTATTGATCTGATCAGGCTG | chr.8(-): 113362428- 113362516 |  |
| cow | SNORD111A | AGCCTGAAATGATGATTTACATTCATGTCTCTTCTCTGATAAATCTCTTGAAGAAAACTCTGAATATCTGATCAGGCT | chr.18 (-): 963371- 963448 |  |
| cow | SNORD111B | ACAGCCTGAAATGATGATTCTTTAAATTTCATGTCTCTTCTCTGACATTTTTCTCTGGAGATAGTTTTTGCCTTATTAATCTGATCAGGCTGT | chr.18(-): 958598- 958690 |  |
| opossum | SNORD111 | AGCCTGAAATGATGATTCACATTCATGTCTCTTCTCTGAGATTTCTTCTGGAGAAAATATGGTGTAACTGATCAGGCT | chr. 1 (+): 708253166- 708253243 |  |
| platypus | SNORD111A | GGGAAGGCCTGGAATGATGATCCAGATTCATGTCTCTTCTCTGAGATTTCCTCTGGAGAAAATCCTGTTTTTTATCTGATCGGGCCTTCCC | Ultra 55 (+):413945- 414035 | The nucleotide complementary to the SNORD111 RNA target is shown in red. SNORD111B can guide the modification of a neighboring site in rRNA. |
| platypus | SNORD111B | TGCCTGAAATGATGACTATTTCATGTCTCTTCTGAGACTCCTGAAGCTCTGGAGATAGCACTTACCTCTCTGATCAGGCA | Ultra 55 (+):414926- 415005 |
| platypus | SNORD111C | GCCTGAAATGATGAGTTCACTTCATGTCTCTTCTCTGAAATCTTCCCTGGAGACACAATTAACCTCGCTGATCTGATCAGGC | Ultra 55 (+):417203- 417284 |
| platypus | SNORD111D | GGGAAGGCCTGGAATGATGATCCAGATTCATGTCTCTTCTCTGAGATTTCCTCTGGAGAAAATCCTGTTTATCTGATCGGGCCTTCCC | Contig 268221 (-): 519 - 606 |
| chicken | SNORD111A | AGCCTGAAATGATGCTGTGAATTCATGTCTCTTCTCTGAAGTGTATGGTGGAGACAACTAGATGAATTTCTGATCAGGCT | chr.11 (-): 1724933- 1725012 |  |
| chicken | SNORD111B | GCCTGATGTGATGAGTTTGTTTCATGTCTCTTCTCTGAAAATGACACTGAAGGTACTCCAAAGACTCTGATCAGGC | chr.11 (-): 1721865- 1721940 |  |
| lizard | SNORD111 | GGCCTGAAATGATGATTAGGAATTCATGTCTCTTCTCTGAAATAAGCCATGGAGATGCTTGGATCCATTTCTGATCAGGTC | scaff.711 (-):393088- 393168 |  |
| frog | SNORD111A | GTATATACAGAACAAAATGATGAGCTTTCATGTCTCTTCTCTGACATAGACAGTGGAGAAAATGAATAACTCTGACACTTGGCTATATGT | scaff.6(-): 4385130- 4385219 |  |
| frog | SNORD111B | GGATGCTTAGAAGACAGAATGATGAGTTTTCATGTCTCTTCTCTGACCCACAGTGGAGAAAAGGAATAACTCTGACACTTGGCTTT | scaff.6(-): 4382863- 4382948 |  |
| frog | SNORD111C | TCCATGCAACAAAGTGATGAGTTTTCATGTCTCTTCTCTGAGATAAAATATGAAGAGAACAAAAAATGCATCTGATCAGCTGCTTGGA | scaff.6(-): 4382028- 4382115 |  |
| frog | SNORD111D | ATCCTTGCAAACTAGAATGATGATTTTTCATGTCTCTTCTCTGATGTAAAACATGGAGACAATAAAACATCTGATAAACTGCTTGGAT | scaff.6(-): 4380124- 4380211 |  |
| zebrafish | SNORD111A | TGTGCTGTCAAAATGATGAATATCTTCATGTCTCTTCTCTGACTGTTTGGTGGAGAGATTGTCTTGATATCTGATTTTCATGCATA | chr.18(+): 15596414- 15596499 |  |
| zebrafish | SNORD111B | AGTAATGAAATGATGAGCTTTTCATGTCTCTTCTCTGACTATTTGACTGGAGACGCATAAAGACCTCTGATCATAATATT | chr.18(+): 15596757- 15596836 |  |
| zebrafish | SNORD111C | TGTGCCAGAAATGATGATTATCTTCATGTCTCTTCTCTGACTGTTTGGTGAAGAGAGATTTGTGATATCTGATCTTCATG | chr.18(+): 15601569- 15601648 |  |
| zebrafish | SNORD111D | TGTGCCAGAAATGATGAATATCTTCATGTCTCTTCTCTGATTGTTTAGTGAAGAGAGATTTGTGATATCTGATCTTCATG | chr.18(+): 15603175- 15603254 |  |
| tetraodon | SNORD111 | GCCCTGTTGAAAAGTGATGAGTTTGTTCATGTCTCTTCTCTGAGTCAAAAGTGAAGAGTGTTGAATGTCTCTGATTTCAGGGT | Un_random (+): 108205368- 108205450 |  |
| mouse | SNORD119 | GCTGGATTCATGATGAAATAGATCCTTGACTGAAGCTGATGATGCGTCTGTGTAATTAAGCAGGATTACTCTGAGATCCAGC | chr.2 (-): 129998638- 129998719 |  |
| opossum | SNORD119A | GCTGGATTGGTGATGAAACCTAACCTTGTCTGAACCTGATGAAGAGTTTGAATTACTCAGCAGGATTACTCTGAGGTCCAGC | chr.1(+): 450204357- 450204438 |  |
| opossum | SNORD119B | CAGCTGGATTAGTGATGAAACATGGCCTTGTCTGAACTTGATGAAGAACTGGGGTCAGTTAGCAGGATTACTCTGAGGTCCAGCTG | chr.1(+): 450221466- 450221551 |  |
| lizard | SNORD119 | AGAACCTGTGATGAGACGACAGCCTCCGAACCCCATGAGGAGATGGTGTCAAAGCAGGATTACTCTGAGGTTCT | scaff.1163 (-):160421- 160494 |  |
| frog | SNORD119 | TTTTGGGATCTGTGATGAGAATTGGCTTATCTGATAATGCTGATGAGCACACATTAAGCAGGATTACCCTGAGATCAAAA | scaff.155 (-):582056- 582135 |  |
| zebrafish | SNORD119 | TAGCTGTGCTGATTCGGCAGCCGTGATGACAGCAGCATTTTCTGAAGCAGATGACGAGTCCGCTTGTGTGGTAGCAGGATTACACTGAGCTCAGAATCACATGGCTG | chr.6 (-): 58179350- 58179456 |  |
| mouse | SNORD121A | CCCGGAAACAATGATGTGATTTCCAAGCacatagctgatgattccatgtggaattttaccactgagtttcctggg | chr.4(-): 41160810- 41160884 |  |
| mouse | SNORD121B | CCaggaaacagtgatgtagtgacgtccaagcacatagctgatgattccatgtagagtttaactgctgagtttcctgg | chr.4(-): 41158537- 41158613 |  |
| mouse | SNORD121C | TGGAAAAGGCAGTGATGTTTCCAAGCACATATCTGACTTATATGTGTGGATGGTAATAATGCTGAGTCTTTTCCG | chr.4(-): 41150091- 41150165 |  |
| dog | SNORD121A | GTCTGGAAAACAATGATGTGATAATTTCCAAGCACATATCTGATGATTCCATGTGGAATTCAGCTACTCTGAGTTTTCTGGAC | chr.11(-): 53737285- 53737367 |  |
| dog | SNORD121B | GTCTCAGAAACGGTGACATGGTAATTTTTTCAAGCACATAGCTTACTCTGCTTAATGAGGTGTTGGCCATTTCTGAGAGAT | chr.11(-): 53730556- 53730636 |  |
| dog | SNORD121C | TGGAAAAGACAATGATGCTTTATTTCCAAGCACATATCTGAGTTGTATGTGTGGATAGAACTAAAACTGAGTCTTTTCTA | chr.11 (-): 53723430- 53723509 |  |
| horse | SNORD121A | GTCCAGAAGACAATGATGTGGTAATTTCCAAGCACATATCTGATGATTCCGTGTGGAATTCAACTACTCTGAGTTTTCTGGAC | chr.23(-): 40403299- 40403381 |  |
| horse | SNORD121B | TGGAAAAGACGATGATGCTTTACTTCCAAGCACATATCTGAGTTGTATGTGTGGATATCACTAAAACTGAGTCTTTTCTG | chr.23(-): 40390772- 40390851 |  |
| opossum | SNORD121A | AAGTCCAACAAACAGTGATGTACTCTTTTCCAAGCACATATCTGACAAATCTATGTGGAAAATAATGCTGCTGAGTTTTTTGGACTT | chr.6(-): 14392369- 14392455 |  |
| opossum | SNORD121B | TAGTCCAGGATACAATGATGTCATAGTTTCCAAGCACATATCTGATTATTAAATGTTGGAATACATCTGTTTCTGAGTTTTCTGGACTA | chr.6(-): 14389102- 14389190 |  |
| platypus | SNORD121A | TTTTTGTCCAGAAGACAGTGATGTGGTATTTTCCAAGCACATATCTGATTGGCATGTGTGGAAATCATTATTTCTGAGTTTTCTGGAATAAGGA | chr.3(+): 31449103- 31449196 |  |
| platypus | SNORD121B | CCAGAAGACAATGATGTGTGATTTCCAAGCACATATCCGATTTCTCCATGTGGAAATCCAACCTCGACTGAGTTTTCTGG | chr.3 (+): 31452954- 31453033 |  |
| platypus | SNORD121C | GAAAAGACAGTGATGTTTCCTTTCCAAGCACATATCCGAATTGTGTGTGTGGACAACTTGAAGACTGAGTCTTTTC | chr.3 (+): 31469066- 31469141 |  |
| platypus | SNORD121D | GAAAGACAATGATGCCTGATTTCCAAGCACATATCTGACCTAATTTGAGGAGAACTCAAGACTGAGTCTTTC | chr.3 (+): 31470346- 31470417 |  |
| chicken | SNORD121A | CCAGAAAACTGTGATGTGATACTTCCAAGCACATATCTGACAGCTTTGTGAGGAAACATGAGTATTTCTGAGTTTTCTGG | chr.Z(-): 6926190- 6926269 |  |
| chicken | SNORD121B | CAGTCCAAAATACAATGATGTTGTCTTTTCCAAGCACATATCTGATTGCGCTATGTGGAAAATGACTGTTGCTGAGTGATTTTGGATTG | chr.Z(-): 6923365- 6923453 |  |
| lizard | SNORD121A | TGGCCAGCAGACTATGATGTGATATTCTTTCCAAGCACATATCTGACTGCTGCATGAGGAAACAAACTGTTTCTGAGTCTTCTGGCCA | scaff. 453 (-):834533- 834620 |  |
| lizard | SNORD121B | GAAAAGACTATGATGTTCACCTTCCAAGCACATATCTGATACAGTATGCTGTGGAAGACCCTATGTTCTGAGTCTTTTC | scaff. 453 (-):828073- 828151 |  |
| frog | SNORD121A | TGTTTCAGAATACAATGATGCTTTATTTTCCAAGCACATAGCTGATATCAATATGCGGAAAGATGATACTGAGTGTTCTGGAACG | scaff.638 (+):387625- 387709 |  |
| frog | SNORD121B | GGAGAGACAATGATGGGAGAATTCCAAGCACATATCTGAGCTTTGTGTTGAGATTACTTGTCACTGAGTCTTTCC | scaff.638 (+):390617- 390691 |  |
| frog | SNORD121C | GGAAAGACCATGATGTGAGAGTTCCAAGCACATATCTGAAGTGAATGTGTTGACACATTTTGTCACTGAGTCTCTTCT | scaff.638 (+):391717- 391794 |  |
| mouse | SNORD123 | TGGTGAAAATGATGAATTCTGGGGCGCTGATTCATGTGACTTGAAAAAACGCCATCCATTTCCTGACTCACCG | chr.15 (-): 32171607- 32171679 |  |
| dog | SNORD123 | GGTGAAAATGATGAACCCTGGGGCGCTGATCACGTGACTTGAAAAACGCCATCCATTTCCTGATTCACC | chr.34(-): 7613230- 7613298 |  |
| opossum | SNORD123 | GGTGAAAATGATGAATTCTGGGGAACTGATTCATGTGACTTGAAAAGGCCATCCATTTCCTGATTCACC | chr.3(+): 105437598- 105437666 |  |
| platypus | SNORD123 | GGTGAAAATGATGAATATTGGGGAACTGATTTATGCGACTTGAAAAGGCCATCCATTTCCTGATTCACC | Contig 22 (+): 1352551- 1352619 |  |
| chicken | SNORD123 | GGTGAAAATGATGAATTCTGGGGTGCTGACTCATGTGATCTGAAAATGCCATCCATTTCCTGATTCACC | chr.2(-): 80732511- 80732579;  Un.random (-): 4539556- 4539624 | The second search hit lies beyond annotated genes and its chromosomal location is unknown. Therefore it is suspected to be an assembly artifact. |
| mouse | SNORD125 | GGCAGCCCCTCTTGGTGATTCCTCCTCCTGAGTGGCTCCAATGATGAGCAAACTGAGCTTCTAAGAAGTTGACTGAATGGGCAGCT | chr.11(+): 4938220- 4938305 |  |
| dog | SNORD125 | AAGCAGCCTCTCCTGATGATTCTTCTTCCTGAGTGGCTCTAGTGATGAGCAAACTGAGCCTCTAAGAAGTTGACTGAAGGGGCTGCTT | chr.26(-): 25668748- 25668835 |  |
| platypus | SNORD125 | GCCTCTCCTGATGACTCTTCTCTTCCTGACTGGCTCCAGTGATGAGTAAACCTGAGCCTCTAAGAAGTTGACTGAAGAGGC | Contig 2379 (+): 87566-87646 |  |
| chicken | SNORD125 | CCAGGGAACAGCCTCACGTGATGATCCTTCTCTCCCTGATGGCTCCGATGGTGATGGACATCAACCCAAAGCCTCTGAGAAGTTGACTGAGGAGGCTGTTTCCTGG | chr.15(+): 11473712- 11473817 |  |
| lizard | SNORD125 | AGGTGGCCTCTCGTGATGACCCCCCTCTCCTGAAGCAGATGGGTTGGTGATGATCGAACCCTCTAAGAAGTTGACTGAAGAGGCTGCTT | scaff.544 (+): 80307-80395 |  |
| rat | SNORD126 | AGTTTGCTATGATGAGATGCATGTGAAGTCCGTGTTTCAGCTGATCACACTGATTACACACATGTGCTGAGCAGACT | chr.15(-): 26781528- 26781604 |  |
| dog | SNORD126 | AGTTTGCCATGATGAAATGCATGGTGAGTCCGTGTTTCAGCCGATCACCCTGGTCAAACACATGCTCTGAGCAGACT | chr.15(-): 20693710- 20693786 |  |
| opossum | SNORD126A | GAAAAGTTTTCTGCCATGATGAGCTGCATGTTTTGTCCGTGTTTCACCTGATTGCCCTGATTATACACATGCCCTGAGCAGCCAAAACTTTT | chr.1(-): 168452248- 168452339 |  |
| opossum | SNORD126B | GTCTGCCATGATGAAATGCAATTTCAGTCCGTGTTTCAGCTGATTACCCTGATTGCATGCATACTCTGAGCAGAC | chr.1(-): 168448370- 168448444 |  |
| platypus | SNORD126A | TCTGCTGGGATGAACTGCATGTTCAGTCCGTGTTTCACCTGATTCCTGATTACACACATGCACTGAGCAGA | Contig 12565(-): 11480-11550 |  |
| platypus | SNORD126B | CCCCCTGCCTTGATGAAATGCATGTTCAGTCCGTGTTTCAGCTGACCACCCTGATTATACACATGCGCTGAGCAGGAGGG | Contig 12565(-): 11066-11145 |  |
| frog | SNORD126A | GGCTTGGCATGATGAGATTGCATAGTCCGTGTTTCACCTGAGATTACAGTGATTATGTAAATGCACTGAACAAGCT | scaff.91 (-): 2408385- 2408460 |  |
| frog | SNORD126B | TGAGCCTGATGTGATGAGATTGCATAGTCCGTGTTTCACCTGAGAATCCTATGATTATGCACATGCCCTGATCAGTCTCA | scaff.91 (-): 2407521- 2407600 |  |
| fugu | SNORD126 | TGTCAAACCTTAATGATGAGTTGCATGTGTCCGTGTTTCACCTGAAACTCGTGATTATAACTTCACTGCACTGATGGGGTTTGACA | Un (+): 322806435- 322806520 |  |
| mouse | SNORD127 | TGGCAACTGTGATGAAAGATTTGGTCTATACAGTAATATATTTATTACTACATGAGGACAACAGTCCCTCTAAACTGATGTTGCCA | chr.12(+): 66157560- 66157645 |  |
| opossum | SNORD127 | TGGCAACAGTGATGACAGGTTTGGTCTGTATGTAATATATTTTATTACTACATGAAGACACCAGTCCCTCTAAGCTGATGTTGCCA | chr.1 (-): 255634968- 255635053 |  |
| platypus | SNORD127 | GGCAACCGTGATGACAGGTTCAGTCTGTATGTGTAATGGGTTTCATTACTACATGAAGACACCAGTCCCTCTAAGCTGATGTTGCC | chr.4(+): 30550454- 30550539;  Contig 32194(-): 2732- 2817 | Very short contig (contig 32194)contains a fragment of the SNORD127 RNA host gene (PRPF39) including the SNORD127B sequence. Until the final assembly of the platypus genome become available it remains unclear wether SNORD127 RNA has one or two genes. |
| chicken | SNORD127 | ATATGGCAACAATGATGACAGGTACTGTAACTGTATGTGATATTTCATTACTGCATGGAGACACCAGTCCCTCTAAACTGATGTTGCCATAT | chr.5(-): 61395764- 61395855 |  |
| lizard | SNORD127 | ACCGGCAACGGTGATGACACTGTGACCCTGCAGGGCCCTGGCTGCTCCGTGGAGACACCAGTCCCTCTAACCTGATGTTGCTGGT | scaff.349 (+):197162- 197246 |  |
| frog | SNORD127 | GGCAACGGTGATGAACAAAAATGGTGTCTTTATGTAATTCCTGTGTTACTTCATGTAGACAACAGTCCCTCTAAACTGCTGTTACC | scaff.634 (+): 112235- 112320 | A substitution in box D. The target of SNORD127 is not methylated in frog. |
| opossum | NET1 | CCCTTGGTTTGAGTTGATGATGTGGGCATGAGCCCATTACCTGATGGACGACATGAGTCTTATGGCATTCTTTGAGTCACCAAAGCCCTGATCAACCAGAGGG | chr.5(-): 300775112 300775214 |  |
| lizard | NET1 | TAGTTTGAAATGATGACACATGTTGATGGACGACGCTAGTCTCATGGCATGAGTTACCAAAGCCCTGATCGAACTG | scaff.1488 (+): 60222-60297 |  |
| frog | NET1 | TGGTCTGAAAGGATGAATGGGTTTTACCCATTAAGTACTGGACGACAAAAGTCATATGGCATTAAGTCACCAAAGCACTGATCAGACTA | scaff.782 (+): 33592-33680 |  |
| zebrafish | NET1A | TCTGAGAGGATGAACGGTTTATTCCGTTTTATCATCTGGACGGCATTATTGCCCCATGGCATTAAAGTCACCAAAGCACTGATCAGA | chr.7(+): 15718603- 15718689 | The host gene has two copies. |
| zebrafish | NET1B | TCTGAGAGGATGAACGGTTTATTCCGTTTTATCATCTGGACGGCATTATTGCCCCATGGCATTAAAGTCACCAAAGCACTGATCAGA | chr.7(+): 15957298- 15957384 |  |
| fugu | NET1 | TGTTTGAAAGGATGAAATGGGGTTAAACCCATCAAACAACTGGACGGCTAATTGTCCCATGGCAGTTTGTCACCAAAGCCCTGATCGAACG | Un. (+): 294291976- 294292066 |  |
| frog | NET2 | GGGGTCTCCAGTGATGAATCATCCACTATACCCTGAGCTGACACATGTTGGCTATGACAAGGATCGTCGCTATACTGAAGAGATTCC | scaff.782 (+): 35979-36065 |  |
| chicken | NET3 | GTTGCAGTCTGCTGTAAATGATGTTGAATCAGGTCTCTGATCCCAGTGAGGATAAAACTTGACTAAACGCTGATACAGTATTTGACTGAGC | chr.8(+): 7731849- 7731939 |
| lizard | NET3 | ATCTGCTGTACATGATGCTGAATCAGGTCACTGATCTTAGTGAGGATAATGAATTGACTGATACAGCAGAT | scaff. 169(+): 2597036- 2597106 |  |
| frog | NET3 | ACTGTTCTGTAAAGGATGATTTATTGAATCAGGTCTCTGACTCCTCTGAGGATAACCTGACCCCACACTGATACAGACGGT | scaff.1(+): 6876805- 6876885 |  |
| zerafish | NET3 | TGGTCTCAAGCTGATGAGTGTGAATCAGGTCTCTGATCTCATTGAGGACATGGAAGCATTTCTGATTTAAGACTG | chr.8(-): 13995089- 13995163 |  |
| fugu | NET3 | GTGCTCGGATGGGATGAGTTTGAATCAGGTCGCCGATCCCGTTGAGGAAAATTAATTAATTCTGATCTGAGCAC | Un. (-): 193907276- 193907349 |  |
