# Supplementary material for: SNOntology: Myriads of novel snornas or just a mirage?
Source: BMC Genomics. 2011 Nov 3;12:543. doi: 10.1186/1471-2164-12-543 (PMC3349704; doi:10.1186/1471-2164-12-543)
Supplement: Additional file 5 — SnoRNA genes not found in the genomes of studied species by Zhang et al. [18] but found in the same species by other researchers. Gene names are listed in the same order as in Figure three in [18]. [file 1471-2164-12-543-S5.DOC]

**Additional file 5. SnoRNA genes not found in the genomes of studied species by Zhang et al. [18] but found in the same species by other researchers**. Gene names are listed in the same order as in Figure three in [18].

SnoRNA genes not found in chicken by Zhang et al. (Group 1-3).

| RNA name | | Chicken gene is present in: | | | |
| --- | --- | --- | --- | --- | --- |
| New | Old | Rfam 10.0 [21] | snoRNA Base [3] | Shao [17] | Zhang [19] |
| SNORD118 | U8 | + | - | - | - |
| SNORA62 | E2, ACA6 | + | + | + (GGgACA40) | + (GGN83) |
| SNORD94 | U94 | + | - | + (GGgCD83) | + (GGN36) |
| SNORA5 | ACA5 | + | + | + (GGgACA22, GGgACA23) | - |
| SNORA23 | ACA23 | + | + | - | + (GGN43) |
| SNORA73 | E1, U17 | + | - | - | - |

SnoRNA genes not found in medaka and zebrafish by Zhang et al. (Group 2-1).

| RNA name | | Medaka gene is present in: | | Zebrafish gene is present in: | |
| --- | --- | --- | --- | --- | --- |
| New | Old | Rfam 10.0 | snoRNA Base | Rfam 10.0 | snoRNA Base |
| SNORA8 | ACA8 | + | - | - | - |
| SNORA53 | ACA53 | + | - | + | - |
| SNORA19 | ACA19 | + | - | + | - |
| SNORD67 | HBII-166 | + | - | - | - |
| SNORA71 | U71 | + | - | + | - |

SnoRNA gene not found in chicken, medaka and zebrafish by Zhang et al. (Group 2-2).

| RNA name | | Chicken gene is present in: | | | | Medaka gene is present in: | | Zebrafish gene is present in: | |
| --- | --- | --- | --- | --- | --- | --- | --- | --- | --- |
| New | Old | Rfam 10.0 | snoRNA Base | Shao [17] | Zhang [19] | Rfam 10.0 | snoRNA Base | Rfam 10.0 | snoRNA Base |
| SNORA42 | ACA42 | - | - | - | - | + | - | - | - |

SnoRNA genes not found in platypus, chicken, medaka and zebrafish by Zhang et al. (Group 2-3).

| RNA name | | Platypus  gene is present in: | | | Chicken gene is present in: | | | | | Medaka gene is present in: | | | Zebrafish gene is present in: | | |
| --- | --- | --- | --- | --- | --- | --- | --- | --- | --- | --- | --- | --- | --- | --- | --- |
| New | Old | Rfam 10.0 | Makarova^1^ | Schmitz[15] | Rfam 10.0 | snoRNA Base | Makarova^1^ | Shao [17] | Zhang [19] | Rfam 10.0 | snoRNA Base | Makarova^1^ | Rfam 10.0 | snoRNA Base | Makarova^1^ |
| SNORD26 | U26 | + | + | + | - | - | - | - | - | - | - | + | - | - | + |
| SNORD27 | U27 | + | + | + | - | - | - | - | - | - | - | + | + | - | + |

SnoRNA genes not found in frog, medaka and zebrafish by Zhang et al. (Group 3-1).

| RNA name | | Frog gene is present in: | | Medaka gene is present in: | | Zebrafish gene is present in: | |
| --- | --- | --- | --- | --- | --- | --- | --- |
| New | Old | Rfam 10.0 | snoRNA Base | Rfam 10.0 | snoRNA Base | Rfam 10.0 | snoRNA Base |
| SNORA61 | ACA61 | - | + | - | **-** | - | - |
| SNORA24 | ACA24 | - | + | - | - | - | - |
| SNORA50 | ACA50 | - | - | + | - | + | + |
| SNORA27 | ACA27 | - | + | + | - | + | - |
| SNORA28 | ACA28 | - | + | + | - | - | - |
| SNORA66 | U66 | - | + | - | - | - | - |
| SNORA7 | ACA7 | - | + | + | - | + | + |
| SNORA72 | U72 | - | - | - | - | - | - |
| SNORD45 | U45 | - | - | - | - | - | - |

SnoRNA gene not found in platypus, frog, medaka and zebrafish by Zhang et al. (Group 3-2).

| RNA name | | Platypus gene is present in: | | Frog gene is present in: | | Medaka gene is present in: | | Zebrafish gene is present in: | |
| --- | --- | --- | --- | --- | --- | --- | --- | --- | --- |
| New | Old | Rfam 10.0 | Schmitz [15] | Rfam 10.0 | snoRNA Base | Rfam 10.0 | snoRNA Base | Rfam 10.0 | snoRNA Base |
| SNORA18 | ACA18 | + | **+** (Oa2001) | - | - | + | - | + | - |

SnoRNA genes not found in chicken, frog, medaka and zebrafish by Zhang et al. (Group 4-1).

| RNA name | | Chicken gene is present in: | | | | Frog gene is present in: | | Medaka gene is present in: | | Zebrafish gene is present in: | |
| --- | --- | --- | --- | --- | --- | --- | --- | --- | --- | --- | --- |
| New | Old | Rfam 10.0 | snoRNA Base | Shao [17] | Zhang [19] | Rfam 10.0 | snoRNA Base | Rfam 10.0 | snoRNA Base | Rfam 10.0 | snoRNA Base |
| SNORA40 | ACA40 | + | + | + (GGgACA20) | + (GGN72) | - | + | - | - | - | - |
| SNORD8 | mgU6-53 | - | - | - | - | - | - | + | - | + | - |
| SNORA25 | ACA25 | + | + | + (GGgACA11) | +(GGN141) | - | + | - | - | - | - |
| SNORA49 | ACA49 | - | - | - | - |  | + | - | - | - | - |
| SNORA41 | ACA41 | + | + | + (GGgACA28) | +(GGN101) | - | + | - | - | - | - |
| SNORA17 | ACA17 | + | + | - | + (GGN58) | - | + | + | - | + | - |
| SNORA31 | ACA31 | + | + | + (GGgACA7, GGgACA38) | + (GGN13, GGN32) | - | + | + | - | + | + |
| SNORA13 | ACA13 | + | + | + (GGgACA25) | - | - | - | + | - | + | + |
| SNORA36 | ACA36 | + | - | + (GGgACA24A) | +(GGN124) | + | + | - | - | + | + |
| SNORA70 | U70 | - | - | - | - | - | + | + | - | + | + |
| SNORA43 | ACA43 | - | - | - | - | - | + | - | - | - | - |

SnoRNA genes not found in platypus,chicken, frog, medaka and zebrafish by Zhang et al. (Group 5-1).

| RNA name | | Platypus gene is present in: | | Chicken gene is present in: | | | | Frog gene is present in: | | Medaka gene is present in: | | Zebrafish gene is present in: | |
| --- | --- | --- | --- | --- | --- | --- | --- | --- | --- | --- | --- | --- | --- |
| New | Old | Rfam 10.0 | Schmitz [15] | Rfam 10.0 | snoRNA Base | Shao [17] | Zhang [19] | Rfam 10.0 | snoRNA Base | Rfam 10.0 | snoRNA Base | Rfam 10.0 | snoRNA Base |
| SNORD87 | HBII-276, U87 | + | + (Oa1892) | + | + | + (GGgCD46) | + (GGN34) | - | + | + | - | - | + |
| SNORA76 | ACA62 | + | - | + | - | + (GGgACA3) | - | - | - | - | - | - | - |
| SNORD116 | HBII-85 | - | - | - | - | - | - | - | - | - | - | - | - |
| SNORD17 | HBI-43 | + | - | + | - | + (GGgCD50) | + (GGN47) | - | - | - | - | - | - |
| SNORD46 | U46 | - | - | - | - | + (GGgCD47) | + (GGN108) | - | - | + | - | + | - |
| SNORA14 | ACA14 | + | + (Oa1849) | + | + | - | - | - | - | + | - | + | + |
| SNORA20 | ACA20 | - | - | + | + | + (GGgACA10) | - | - | - | - | - | - | + |
| SNORA58 | ACA58 | - | - | + | + | + (GGgACA6) | - | - | - | - | - | - | - |
| SNORA74 | U19 | + | - | + | + | - | + (GGN152) | - | + | + | - | + | - |
| SNORA64 | U64 | - | - | - | - | + (GGgACA47) | + (GGN74) | - | - | - | - | - | - |
| SNORA68 | U68 | + | - | + | - | - | - | - | - | + | - | + | - |
| SNORA11 | U107 | - | - | - | - | - | - | - | - | - | - | - | - |
| SNORA4 | ACA4 | + | - | + | + | + (GGgACA26) | + (GGN123) | - | + | - | - | - | - |

SnoRNA gene not found in mouse, platypus, chicken, frog, medaka and zebrafish by Zhang et al. (Group 6-1).

| RNA name | | Mouse gene is present in: | | Platypus gene is present in: | | Chicken gene is present in: | | | | Frog gene is present in: | | Medaka gene is present in: | | Zebrafish gene is present in: | |
| --- | --- | --- | --- | --- | --- | --- | --- | --- | --- | --- | --- | --- | --- | --- | --- |
| New | Old | Rfam 10.0 | snoRNA Base | Rfam 10.0 | Schmitz [15] | Rfam 10.0 | snoRNA Base | Shao [17] | Zhang [19] | Rfam 10.0 | snoRNA Base | Rfam 10.0 | snoRNA Base | Rfam 10.0 | snoRNA Base |
| SNORA15 | ACA15 | + | + | - | - | + | - | - | + (GGN56) | - | - | + | - | - | - |

^1^ Additional file 4 in this article.
