# Supplementary material for: SNOntology: Myriads of novel snornas or just a mirage?
Source: BMC Genomics. 2011 Nov 3;12:543. doi: 10.1186/1471-2164-12-543 (PMC3349704; doi:10.1186/1471-2164-12-543)

Additional file 6. Nearly all examples of independent transcription of snoRNA genes in Li et al. [20] are erroneous. Screenshots of UCSC Genome Browser (March 2006, NCBI Build 36.1) and nucleotide sequence alignments of snoRNA genes and pseudogenes are shown. The antisense elements are boxed; H, ACA, C, and D/D' sequences are underlined. The nucleotides whose modification is guided by snoRNA are indicated in some cases. SnoRNA genes and pseudogenes (designated as pseudo or Ψ) are listed in the same order as in Tables 3, 4, and 5 of Li et al. [20]. The secondary structures were predicted by mfold [43, 44].

SNORD104 and **SNORA76 genes** are localized in introns of host gene**.**

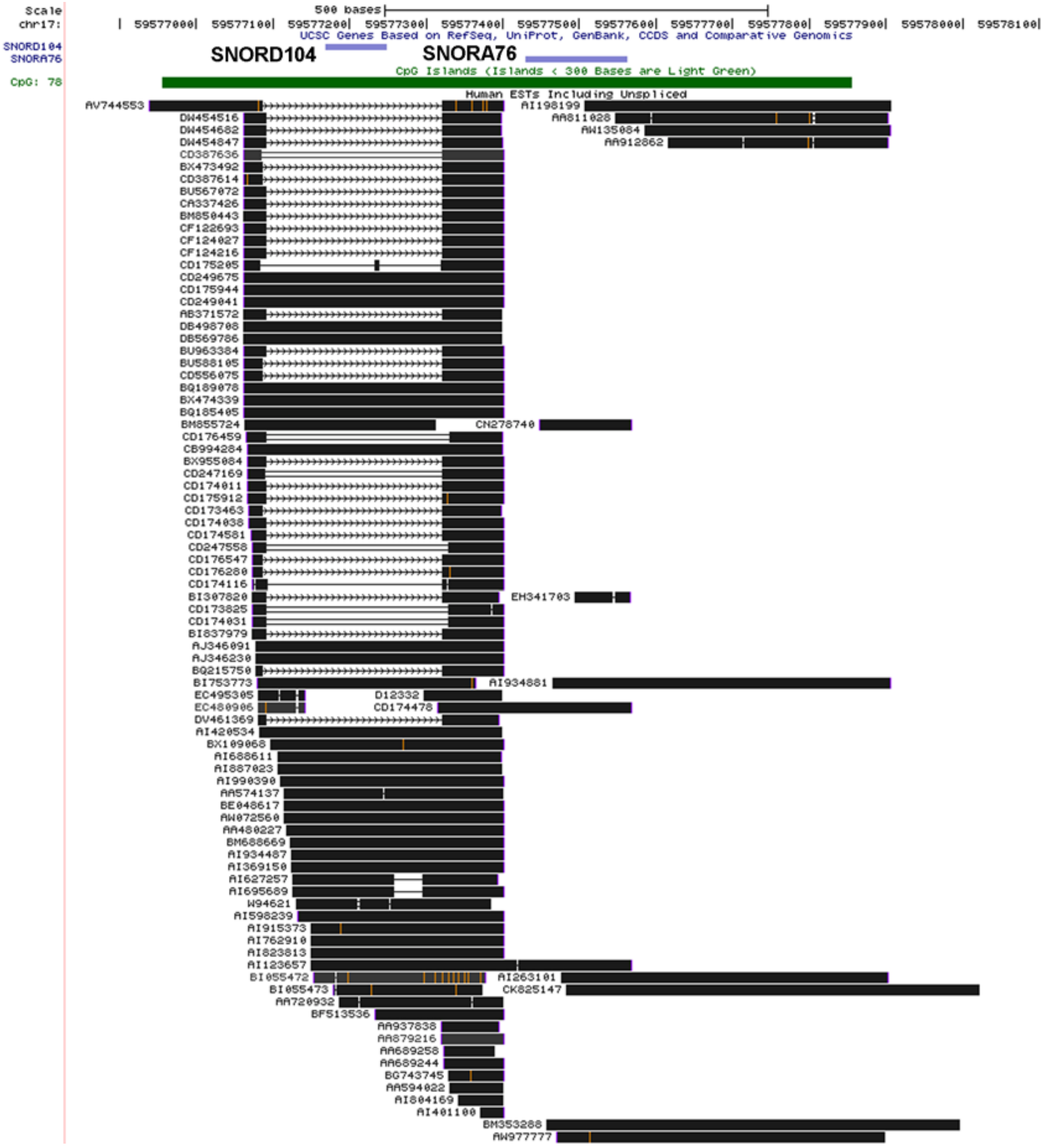

SNORD123 gene is localized in intron of host gene.

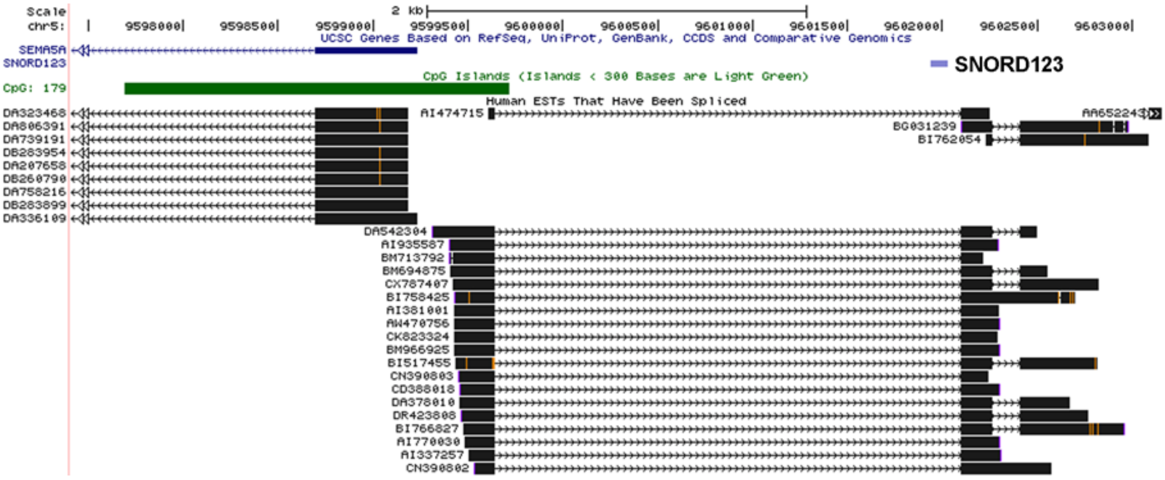

SNORA69 pseudogene on chr17(+):8173626-8173762. The pseudogene secondary structure differs from that of SNORA69 and does not correspond to the canonical H/ACA RNA structure. In particular, the pseudogene box H is within the hairpin, the first antisense element has substitutions. These are indications of a pseudogene. Double-stranded regions are marked with different colors.

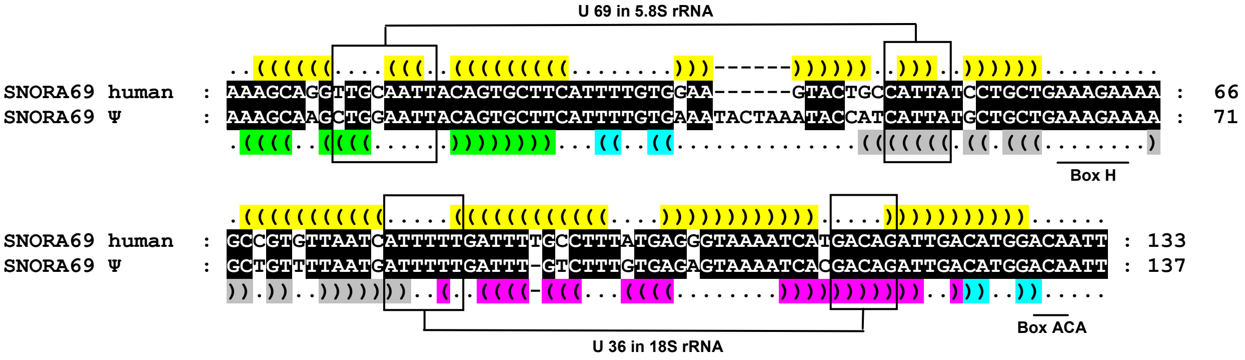

SNORA3 pseudogene on chr16(-):2786410-2786534. The antisense elements have substitutions.

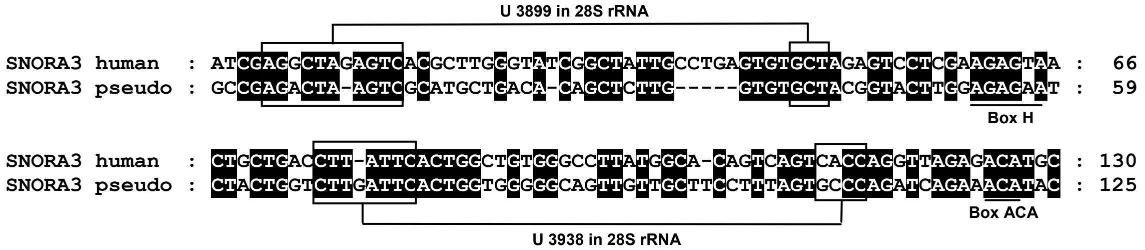

SNORA51 pseudogene on chr7(-):137523284-137523415. Secondary structure of the pseudogene differs from SNORA51 structure and does not correspond to the canonical H/ACA RNA folding. In addition, box H of the pseudogene is located in hairpin, and contains nucleotide substitutions. Double-stranded regions are marked with different colors. Target RNA unknown.

```
                  .(((((((....((....((((...((((....))))...))))..))....)))))))).......
SNORA51 human  : GGCCTCCTGGTGCTTACCACAGGCTGTGTTCTTACACTGACTGTATAGAAAGAGGAGGTAGAGTAA :  66
SNORA51 pseudo : AGCCTCCTGGTGCTCAGCACAGGATGTGTTCTTACCCTAACCAAATAGAAAGAGTAGGCTGAATTA :  66
                  ....(((((.(((...)))))))).((((((..((((....((((((((..((((.(((.......
                                                                            Box H

                  .....((((((.((((...(((..(((((.....)))))..)))...)))).))))))).........
SNORA51 human  : ACCTACCCCATATACACCTCAGCTCAGGCCCTGTGCCTGGTCTGTATTGTGAATGGGGGAACATAG : 132
SNORA51 pseudo : ACCTATGCCATATACACCTCAACCCAGGCCCTGCAACTCCTCTGTATTGTCAATGGGTGGACATGG : 132
                  ......))).......(((......)))........)))).))))).))).....))))))))))..
                                                                          Box ACA
```

SNORD3 (U3) pseudogenes on chr17(-):54064002-54064196 and chr1(+):218202617-218202829. Alignment of human SNORD3 genes and two pseudogenes is shown. Conserved elements are underlined (after Granneman S, Vogelzangs J, Lührmann R, van Venrooij WJ, Pruijn GJ, Watkins NJ: **Role of Pre-rRNA Base Pairing and 80S Complex Formation in Subnucleolar Localization of the U3 snoRNP.** *Mol Cell Biol* 2004, **24:**8600-8610). 5′-hinge and 3′-hinge are SNORD3 fragments complementary to the regions of 5′-ETS of pre-rRNA. The pseudogenes have numerous substitutions and indels.

```
                   GAC box  Box A'       Box A              5' hinge              3' hinge
U3             : AAGACTATACTTTCAGGGATCATTTCTATAGTGTGTTACTAGAGAAGTTTCTCTGAACGTGTAGAGCACCGAAA :  74
U3-2           : AAGACTATACTTTCAGGGATCATTTCTATAGTGTGTTACTAGAGAAGTTTCTCTGAACGTGTAGAGCACCGAAA :  74
U3-2b          : AAGACTATACTTTCAGGGATCATTTCTATAGTGTGTTACTAGAGAAGTTTCTCTGAACGTGTAGAGCACCGAAA :  74
U3-3           : AAGACTATACTTTCAGGGATCATTTCTATAGTGTGTTACTAGAGAAGTTTCTCTGAACGTGTAGAGCACCGAAA :  74
U3-4           : AAGGCTATACTTTCAGGGATCATTTCTATAGTGTGTTACTAGAGAAGTTTCTTTGAACGTGTAGAGCACCGAAA :  74
U3 Ψ on chr.17 : AAGACTATATTTTGAGGGATAATTTCTATAGTGTGTTTCTCGAGGAGTATATCTGAACGTGTAGAACGCTGGAA :  74
U3 Ψ on chr.1  : AAGACTTTATCCCC-----CCACCCCGCAAAGAAAAGGAAGAAGAAGAGTTTTTAAATG-GCGCAGCCCTGGAA :  68

                        Box C'                    Box B
U3             : ACCACGAGGAAGAGAGGTA-GCGTTTTCTCCTGAGCGTGAAGCCGGCTTTCTGGCGTTGCTTGGCTGCAACTGC : 147
U3-2           : ACCCCGAGGAAGAGAGGTA-GCGTTTTCTCCTGAGCGTGAAGCCGGCTTTCTGGCGTTGCTTGGCTGCAACTGC : 147
U3-2b          : ACCCCGAGGAAGAGAGGTA-GCGTTTTCTCCTGAGCGTGAAGCCGGCTTTCTGGCGTTGCTTGGCTGCAACTGC : 147
U3-3           : ACCACGAGGAAGAGAGGTA-GCGTTTTCTCCTGAACGTGAAGCCGGCTTTCTGGCGTTGCTTGGCTGCAACTGC : 147
U3-4           : ACCCCGAGGAAGAGAGGTA-GCGTTTTCTCCTGAGCGTGAAGCCGGCTTTCTGGCGTTGCTTGGCTGCAACTGC : 147
U3 Ψ on chr.17 : ACCGCGAGAGGGAGATATTTGTGTTCTCTCACGAGTGGATAGCAG-TGTTTGGTGTTGCGTTATTCTAACTGC : 147
U3 Ψ on chr.1  : TCCACAAGGAGGAGGTTCA-GTATTCTCTCCTGAGCGTGAAGTTGG-TTCTTGGCATTGCTTCACTGCAACTGC : 140

                           Box C                                          Box D
U3             : CGTCAGCCATTGATGATCGTTCTTCTCTCCGTATTGGGGAGTGAGAGGGAGAGA----ACGCGGTCTGAGTGGT : 217
U3-2           : CGTCAGCCATTGATGATCGTTCTTCTCTCCGTATTGGGGAGTGAGAGGGAGAGA----ACGCGGTCTGAGTGGT : 217
U3-2b          : CGTCAGCCATTGATGATCGTTCTTCTCTCCGTATTGGGGAGTGAGAGGGAGAGA----ACGCGGTCTGAGTGGT : 217
U3-3           : CGTCAGCCATTGATGATCGTTCTTCTCTCCGTATTGGGGAGTGAGAGGGAGAGA----ACGCGGTCTGAGTGGT : 217
U3-4           : CGTCAGCCATTGATGATCGTTCTTCTCTCCGTATTGGGGAGTGAGAGGGAGAGA----ACGCGGTCTGAGTGGT : 217
U3 Ψ on chr.17 : TGTTTGCTGTTGGTGACCGTTCTTCTCT---------------------AGAGA----AGGCAGTCTGAGTAG- : 195
U3 Ψ on chr.1  : TATTTGCCATTGATGATTGTTCTTCTCTTCCTCTGAGAGAGTAAGAGGGAGAGGATGCATGCAGTCTGAGTGG- : 213
```

SNORA70 pseudogene on chr19(+):9791649-9791770. The pseudogene has no 5'-terminal region of SNORA70

```
SNORA70A human : -CCGCAGCCAATTAAGCCGACTGAGTTCCTTTCCTCATGGGGA--CCCAGTGTGCGATGGCTGCACACAGCAGCT :  72
SNORA70B human : --TGCAGCCAATTAAGCTGACTGAATTCCTTTCCTTATGGGGG--TCCAGTGTGCAATGGCTGTAAACAGCAGCT :  71
SNORA70C human : TCTGCAGCCAATTAAGCCAACTGGGTTCCTTTCCTCATGGGGG--CCCATTGTGCAATGGCTGCAAACAGCAGCT :  73
SNORA70D human : -CTGCAGACAGTTAAGCCAACTGAGTTCCTTTCCTCAGGGAGG--CCCAGTGTACAATGGCTGCCCACAGCAGCT :  72
SNORA70E human : -CTGCAACCAATTAAGCCGACCTAGTTCCTTTCCTCTTTGGGG--CCTGGTGTTCAATAGCTGCAAACAGCAGCT :  72
SNORA70F human : -CTGCAGTCAATTAAGTGTACTGAGTTCCTTTCCTTATGGGGG--CCCAGTGTGCAATGGCTGCAAACAGCAGCT :  72
SNORA70G human : -CTGCAGCCTATTAAGCCAACTGAGTTCCTTTCCTCATGGGGGGCCCAGTGTGCAATGGCTGCAAACAGCAGCT :  74
SNORA70 Ψ     : ---------TGGCAAGTCCACCAAGAAACTATAAAAATGTGTG-------TGTGCAATGGCTGCACACAGCAGCT :  59
                                                                               Box H

SNORA70A human : TCCTTGGTAGTGTACGCAGCCTGTTGGTTGTAT-----GGGTTGCTCTAAGGGACCTTGGAGACAGGC : 135
SNORA70B human : TCCTTGGTAGTGTATGCGGCCTGTTTGTTGTAT-----AGGTTGCTCTAAGGGACCTTGGAGACAGGC : 134
SNORA70C human : TTCTTGGTAGTGTATGCAGCCTGTTTGTTGTAT-----GGGTTGCTTTAAGGGACCATGGAAACAGGC : 136
SNORA70D human : TCCTTGGTAGCGTACTCAGCCTGTTTCTTGTAT-----GGGTTGCTCTAAGGGACCTTAGAGACAGGC : 135
SNORA70E human : TCCTTGGTAGTGTATGCAGCCTGTTTCTTGTAT-----GGGTTGCTCTAAAGGACCTTGGAGACAGCC : 135
SNORA70F human : TCCTTGGTGGTGTATGCAGCCTGTTTCCTCTAT-----AGGTTGCTCTAAGGGACCTTGATAATAGGC : 135
SNORA70G human : TCCTTGGTAGTGTATGCAGCCTGTGTGTTGTATTGTATGGGTTGCTCTAAGGGACCCTGGAGACAGTC : 142
SNORA70 Ψ     : TCCTTGGTAGTGTACGCAGCCTGTTGGTTGTAT-----GGGTTGCTCTAAGGGACCTTGGAGACAGGC
                                                                   Box ACA
                          U 1692 in 18S rRNA
```

SNORA48 pseudogene on chr16(-):11275471-11275594. The pseudogene is missing a part of the antisense element.

```
SNORA48 human : TGTCCCTGACCTGGGTAGAGTGGCATCTGGTTGGTGATGCCCATCTCATATCAGCCAGGGACAAAGCAACTCCTTG : 76
SNORA48 pseudo: CCACCCTGACCTGGGTAGAGTGGCCTCTGGTTGGTGGTGTCCATCTTGCATCAGTCAGGGACAAAGCAACCCCTTG : 76
                                                                      Box H

                                                                 Box ACA
SNORA48 human : TTCATCCCAGCTTGGCTTTTGATCCGTGCCCATGCCTGGTTCATGCCTTGGACACATAG : 135
SNORA48 pseudo: TTCATCCCAGCTTGGTTTCTGGCCTGTTCCCATGCCT-----------TGGACAAACAA : 124
                                                                  Box ACA
                      U 3797 in 28S rRNA
```

SNORA75 pseudogenes on chr12(+):9488933-9489068 (pseudo 1) and chr12(-):9330536-9330660 (pseudo 2). The pseudogenes have no 5'-terminal region of SNORA75.

```
                              U 93  in 18S rRNA
SNORA75 human    : ----GTCTTCTCATTGAGCTCCTTTCTGTCTATCAGTGGCAGTTTATGGATTCGCACGAGAAGAAGAGAGAATTCA :  72
SNORA75 pseudo 1 : TTCGGTTTAGGAGCAATTAAAATGACTACAGATTGGTGGCAGTTTATGGATTTGCAC---AAGAAGAG--AACTCA :  71
SNORA75 pseudo 2 : -------------CAATTAAAATGACTACAGATTGGTGGCAGTTTATGGATTTGCAC---AAGAAGAGAGAACTCA :  60
                                                                                Box H

SNORA75 human    : CAGAACTAGCATTATTTTACCTTCTGTCTTTACAGAGGTATATTTAGCTGTATTGTGAGACATTC : 137
SNORA75 pseudo 1 : CAGAACTAGCATTATTTTACCCTCTGTCTTTACAGAGGTGTATTTGGCTGTTTTGTTAGACATTC : 136
SNORA75 pseudo 2 : CAGAACTAGCATTATTTTACCCTCTGTCTTTACAGAGGTATATTTGGCTGTTTTGTTAGACATTC : 125
                                                                   Box ACA
```

SNORA36 pseudogene on chr2(-):27718414-27718540. Antisense elements are located in different hairpins (blue and green) and contain substitusions.

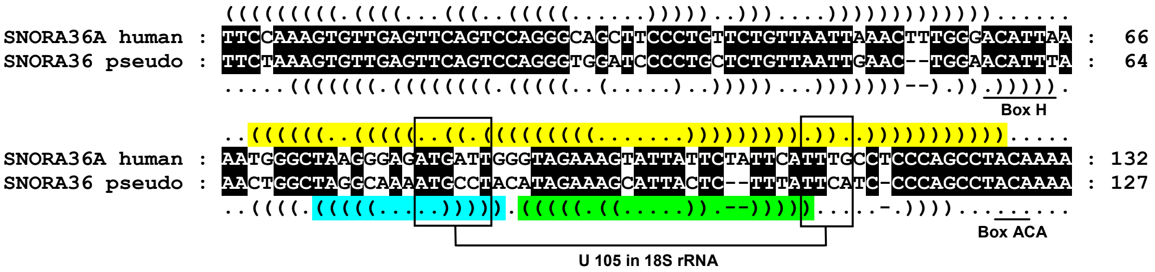

SNORD51 pseudogene on chr14(-):103333362-103333438. The pseudogene has substitutions in the antisense element and boxes C and D′. The nucleotide complementary to the modification site is indicated by the red arrowhead.

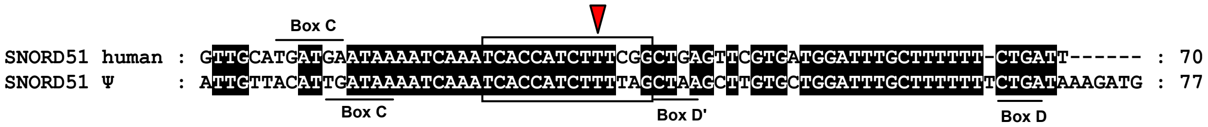

Supplement: Additional file 6 — Nearly all examples of independent transcription of snoRNA genes in Li et al. [20] are erroneous. Screenshots of UCSC Genome Browser (March 2006, NCBI Build 36.1) and nucleotide sequence alignments of snoRNA genes and pseudogenes are shown. The antisense elements are boxed; H, ACA, C, and D/D' sequences are underlined. The nucleotides whose modification is guided by snoRNA are indicated in some cases. SnoRNA genes and pseudogenes (designated as pseudo or Ψ) are listed in the same order as in Tables three, four, and five of Li et al. [20]. The secondary structures were predicted by mfold [42,43]. [file 1471-2164-12-543-S6.PDF]
